# Feasibility and Efficacy of a Resiliency Intervention for the Prevention of Chronic Emotional Distress Among Survivor-Caregiver Dyads Admitted to the Neuroscience Intensive Care Unit: A Randomized Clinical Trial

**DOI:** 10.1001/jamanetworkopen.2020.20807

**Published:** 2020-10-14

**Authors:** Ana-Maria Vranceanu, Sarah Bannon, Ryan Mace, Ethan Lester, Emma Meyers, Melissa Gates, Paula Popok, Ann Lin, Danielle Salgueiro, Tara Tehan, Eric Macklin, Jonathan Rosand

**Affiliations:** 1 Integrated Brain Health Clinical and Research Program, Department of Psychiatry, Massachusetts General Hospital, Boston, Massachusetts; 2 Henry and Allison McCance Center for Brain Health, Massachusetts General Hospital, Boston; 3 Harvard Medical School, Boston, Massachusetts; 4 Neuroscience Intensive Care Unit, Massachusetts General Hospital, Boston; 5 Biostatistics Center, Massachusetts General Hospital, Boston

## Abstract

**Importance:**

To our knowledge, there are no evidence-based interventions to prevent chronic emotional distress (ie, depression, anxiety, and posttraumatic stress [PTS]) in critical care survivors and their informal caregivers.

**Objective:**

To determine the feasibility and preliminary effect of the novel dyadic resiliency intervention Recovering Together (RT) on reducing symptoms of depression, anxiety, and PTS among hospitalized patients and their informal caregivers.

**Design, Setting, and Participants:**

This single-blind, pilot randomized clinical trial of RT vs an educational control was conducted among 58 dyads in which either the survivor or caregiver endorsed clinically significant symptoms of depression, anxiety, or PTS. The study was conducted in the neuroscience intensive care unit at Massachusetts General Hospital. Data were collected from September 2019 to March 2020.

**Interventions:**

Both RT and control programs had 6 sessions (2 at bedside and 4 via live video after discharge), and both survivor and caregiver participated together.

**Main Outcomes and Measures:**

The primary outcomes were feasibility of recruitment and intervention delivery, credibility, and satisfaction. The secondary outcomes included depression and anxiety (measured by the Hospital Depression and Anxiety Scale), PTS (measured by the PTSD Checklist–Civilian Version), and intervention targets (ie, mindfulness, measured by the Cognitive and Affective Mindfulness Scale–Revised; coping, measured by the Measure of Current Status–Part A; and dyadic interpersonal interactions, measured by the Dyadic Relationship Scale). Main outcomes and targets were assessed at baseline, 6 weeks, and 12 weeks.

**Results:**

The 58 dyads were randomized to RT (29 dyads [50.0%]; survivors: mean [SD] age, 49.3 [16.7] years; 9 [31.0%] women; caregivers: mean [SD] age, 52.4 [14.3] years; 22 [75.9%] women) or control (29 dyads [50.0%]; survivors: mean [SD] age, 50.3 [16.4] years; 12 [41.3%] women; caregivers, mean [SD] age, 52.1 [14.9], 17 [58.6%] women). Feasibility (recruitment [76%], randomization [100%], and data collection [83%-100%]), adherence (86%), fidelity (100%; κ = 0.98), satisfaction (RT: 57 of 58 [98%] with scores >6; control: 58 of 58 [100%] with scores >6), credibility (RT: 47 of 58 [81%] with scores >6; control: 46 of 58 [80%] with scores >6), and expectancy (RT: 49 of 58 [85%] with scores >13.5; 51 of 58 [87%] with scores >13.5) exceeded benchmarks set a priori. Participation in RT was associated with statistically and clinically significant improvement between baseline and postintervention in symptoms of depression (among survivors: −4.0 vs −0.6; difference, −3.4; 95% CI, −5.6 to −1.3; *P* = .002; among caregivers: −3.8 vs 0.6; difference, −4.5; 95% CI, −6.7 to −2.3; *P* < .001), anxiety (among survivors: −6.0 vs 0.3; difference, −6.3; 95% CI, −8.8 to −3.8; *P* < .001; among caregivers: −5.0 vs −0.9; difference, −4.1; 95% CI, −6.7 to −1.5, *P* = .002), and PTS (among survivors: −11.3 vs 1.0; difference, −12.3; 95% CI, −18.1 to −6.5, *P* < .001; among caregivers, −11.4 vs 5.0; difference, −16.4, 95% CI, −21.8 to −10.9; *P* < .001). Improvements sustained through the 12-week follow-up visit. We also observed RT-dependent improvement in dyadic interpersonal interactions for survivors (0.2 vs −0.2; difference, 0.4; 95% CI, 0.0 to 0.8; *P* = .04).

**Conclusions and Relevance:**

In this pilot randomized clinical trial, RT was feasible and potentially efficacious in preventing chronic emotional distress in dyads of survivors of the neuroscience intensive care unit and their informal caregivers.

**Trial Registration:**

ClinicalTrials.gov Identifier: NCT03694678

## Key Points

**Question:**

Is participation in a dyadic resiliency intervention associated with a measurable reduction in symptoms of depression, anxiety, and posttraumatic stress (PTS) compared with participation in an educational control?

**Findings:**

In this pilot, single-blind, randomized clinical trial of 58 dyads of survivors of the neuroscience intensive care unit and their informal caregivers, survivors and caregivers who received the active intervention experienced a significant reduction in symptoms of depression, anxiety, and PTS.

**Meaning:**

In this pilot randomized clinical trial, the dyadic resiliency intervention was feasible, and the findings suggest that it may prevent chronic emotional distress in survivors of acute brain injury and their caregivers.

## Teaser

This pilot randomized clinical trial evaluates the feasibility and preliminary effect of the novel dyadic resiliency intervention Recovering Together in reducing symptoms of depression, anxiety, and posttraumatic stress among patients hospitalized in the neuroscience intensive care unit and their informal caregivers.

## Introduction

Advanced technologies have transformed the quality of care for patients with critical neurologic illness admitted to the intensive care unit (ICU), contributing to increased survival and more favorable discharge dispositions.^1^ However, as more and more patients survive their neuroscience ICU stays, the long-term psychiatric sequelae associated with the sudden, life-threatening, and often traumatic onset of acute neurological injury (ANI) are becoming increasingly recognized. These psychiatric sequelae affect not only the survivors but also the informal caregivers^2^ who support and attend to them through their hospitalizations and beyond.^3,4,5,6,7^ Rates of clinically significant anxiety, depression, and posttraumatic stress (PTS) are high for both patients (43%, 24%, and 21%, respectively) and caregivers (46%, 8%-24%, and 17%, respectively) during hospitalization and remain high 3 and 6 months later.^3,4,5,6,7^ This trajectory of emotional distress is concerning given the deleterious effect that long-term distress has on patients’ physical recovery as well as patients’ and caregivers’ morbidity and mortality.^8,9,10,11,12^ Early interventions that prevent chronic psychiatric illness in at-risk patients and caregivers are therefore an urgent priority.

The critical care community has responded to this need, but to our knowledge, no interventions have been successful in addressing this problem. A recent systematic review and meta-analysis^13^ showed potential for ICU diaries to reduce the risk for depression and anxiety in ICU survivors but not in informal caregivers and not for PTS. Unfortunately, only 3 randomized clinical trials (RCTs) with very small samples were included in the review, diminishing generalizability. Recently, Wade et al^14^ reported on a large, multicenter, cluster RCT of a nurse-led cognitive behavioral therapy intervention among at-risk ICU survivors that showed no significant reduction in PTS symptom severity 6 months after discharge. White and colleagues^15^ reported on a large stepped-wedge cluster RCT of a nurse-delivered family support intervention for surrogates of critically ill patients from 5 ICUs and found no effect on improvement in emotional distress when compared with control.

For the past 5 years, our multidisciplinary team of psychologists, nurses, and neurointensivists built on limitations of prior research and used a sequential approach to psychosocial intervention development to create Recovering Together (RT),^16,17^ the first dyadic intervention (treating survivors and caregivers together) aimed at preventing chronic emotional distress in this population. We began with qualitative interviews to understand population needs and perceptions of the nursing team and a feasibility study to refine the intervention and protocols.^16,17,18^ This preliminary work supported 5 key decisions regarding intervention content, format, and methods. First, we started the intervention in person at bedside and offered support throughout transition to rehabilitation facilities or home through secure video, while prior studies included support only in or out of the hospital. Second, as symptoms of depression, anxiety, and PTS are interrelated and prevalent during hospitalization,^4^ we used a transdiagnostic approach focused on addressing the construct of emotional distress by providing in vivo coping skills, rather than specifically targeting 1 particular psychiatric condition. Third, because psychiatric distress and resiliency factors are interdependent between survivors and caregivers,^5,6,7,19^ to fully account for the nuanced presentation of distress, we targeted dyads rather than survivors or caregivers independently. This approach is supported by research from psycho-oncology and recommendations from the American Heart Association.^20^ Fourth, we used simplified mindfulness-based and dialectical behavioral therapy skills^21^ rather than cognitive skills, which may be too taxing for patients with ANIs to address initial stress response to critical illness, uncertainty surrounding prognosis, and concerns regarding each other’s well-being, which were the key challenges reported by dyads during hospitalization.^16,17^ We used skills with a more cognitive focus in the postdischarge sessions. Fifth, noting that dyads have heterogeneous needs after hospitalization,^17,22^ we designed RT as a tailored intervention that includes flexible, modular posthospitalization sessions that meet the unique needs of each dyad.

Here we present results from a pilot, single-blind RCT of RT vs an educational control. We hypothesized that the study would meet a priori feasibility benchmarks and that participation in RT would be associated with sustained clinically and statistically significant improvements in depression, anxiety, and PTS. We also explored improvement in intervention targets (ie, coping, mindfulness, and dyadic interpersonal relationships).

## Methods

### Study Design, Setting, and Participants

We conducted a single-site, single blind, RCT in the neuroscience ICU at Massachusetts General Hospital from September 2019 to March 2020. The complete protocol appears as Supplement 1. We aimed to randomize approximately 30 dyads per group.^23,24,25^ We followed the Consolidated Standards of Reporting Trials (CONSORT) reporting guideline.^26^ The Massachusetts General Hospital institutional review board approved this study, and all dyads provided written informed consent prior to participation.

Although the primary aim of the pilot trial was feasibility, this sample provided 80% power to detect a moderate to large effect size. Participants were recruited through direct referrals from the nursing team. Study staff met every morning with the nurse champion to review admissions and identify caregivers. When possible, the bedside nurse helped introduce the study. An institutional review board–approved recruitment video, which included 2 dyads who successfully completed a prior feasibility study,^18^ was used as needed. Inclusion criteria for patients were as follows: (1) aged at least 18 years; (2) cleared medically and cognitively for participation; (3) Mini-Mental State Examination score of at least 24; (4) access to a smartphone, laptop, or computer; (5) informal caregiver willing to participate; and (6) English fluency. Inclusion criteria for caregivers were equivalent. Within each dyad, either the patient, caregiver, or both needed to screen in for clinically significant depression or anxiety (Hospital Anxiety and Depression Scale–Depression [HADS-D] or HADS-Anxiety [HADS-A] score, >7) or PTS (*Diagnostic and Statistical Manual of Mental Disorders* (Fifth Edition) criteria determined by PTSD Checklist–Civilian Version [PCL-C]). We excluded patients unable to participate because of severity of the ANI, Glasgow Coma Scale (GCS)^27^ score of less than 10, premorbid cognitive impairment, aphasia, or who were judged by the medical team as unlikely to be able to participate due to predicted permanent impairment.

A total of 220 dyads were referred; 22 were found ineligible before screening; 28 declined screening; 16 were discharged prior to screening; and contact was discontinued for 7 (Figure).

**Figure.**
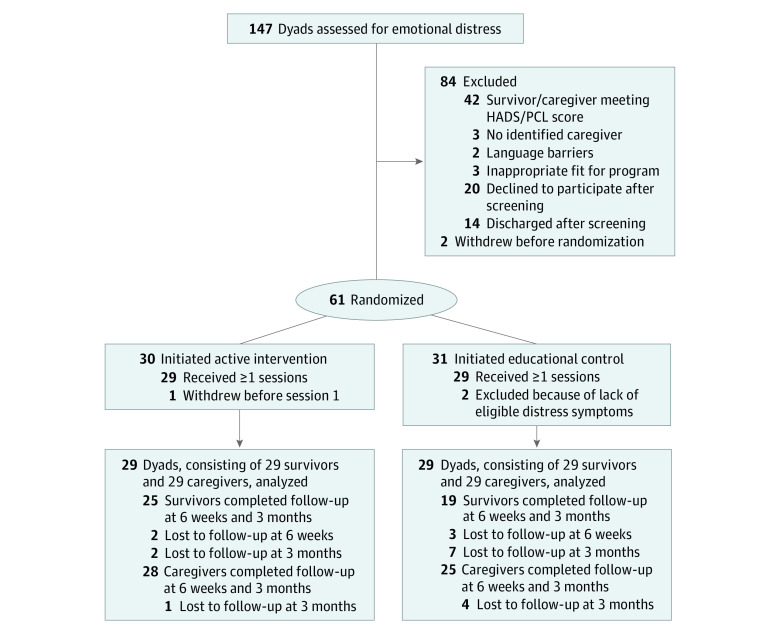
Study Flowchart HADS indicates Hospital Anxiety and Depression Scale; and PCL, PTSD Checklist–Civilian Version.

### Randomization, Allocation Concealment, and Follow-up

We used a computer-generated randomization sequence with permuted blocks of size 2 and 4 and 1:1 allocation to RT or control. Treatment assignments were implemented using REDCap.^28^ All study staff besides the statistician were blind to the allocation algorithm. Manuals were labeled *Recovering Together 1* (active) and *Recovering Together 2* (control) to keep dyads blinded. Self-reported measures were collected at baseline (in person), postintervention, and 12 weeks later (REDcap link). Study staff assisted participants with questionnaire completion as needed.

### Treatment Conditions

#### RT Active Intervention

RT content was based on formative qualitative and quantitative research with neuroscience ICU dyads and nursing teams; theoretical frameworks^29,30,31,32,33^; strategies drawn systematically from mindfulness, cognitive-behavioral, and positive psychology principles; and feedback from nursing and medical staff.^13,14^ The first 2 sessions taught concrete skills focused on helping dyads get through the trauma of the hospitalization and focus on self-care. The subsequent 4 sessions were tailored to the specific needs of each dyad based on specific challenges, sequelae, or concerns identified collaboratively by the therapist and dyad, from a total of 6 available modules (Table 1). At recruitment, study staff installed a web-based application on participants’ smartphones with all session content and recordings to facilitate practice. The development of the intervention and description and case study have been previously published.^16,17,18^

**Table 1.** RT Session Content

Module	Location	Purpose	Skills
General	NICU	Staying in the here and now	Education on emotional distress post-ANI tailored to dyad answers on HADS and PCL Diaphragmatic breathing—immediate relief from intense emotions Mindfulness: observe, describe, participate, stay within a 24-hour block Self-care: sleep, diet, movement, pleasant activities, engaging support Skill practice planning tailored to HADS and PCL answers and dyad examples; individually, together, or both using RT mobile app
General	NICU	Coping with uncertainty	Dialectics, ie, how to let more than 1 thought or feeling be true at the same time Coping with worries, ie, deciding between change and acceptance Understanding active vs emotional coping Skill practice planning; individually, together, or both using RT mobile app
Specific	After NICU	Adjusting to life after NICU	Review and identify challenges for both patient and caregiver together Identify the interrelation of thoughts, behaviors, emotions, and physical sensations and learn to make changes at all these levels and support each other in making these changes Skill practice planning; individually, together, or both using RT mobile app
Specific	After NICU	Navigating interpersonal relationships	Review and identify changes in roles, gender roles, and personal identity Review and identify changes in self-image Interpersonal effectiveness skills Skill practice planning; individually, together, or both using RT mobile app
Specific	After NICU	Adherence to rehabilitation and self-care	Strategies to adhere to self-care and rehabilitation Setting specific, measurable, attainable, realistic, and time-bound (SMART) goals to achieve them Assisting each other in making these goals Skill practice planning; individually, together, or both using RT mobile app
Specific	After NICU	Fear of recurrence	Decision coping tree to differentiate things that you can control from those you cannot Using mindfulness and dialectics to cope with fear of recurrence; learning if it is time for acceptance or change Skill practice planning; individually, together, or both using RT mobile app
Specific	After NICU	Making meaning	Exploring the ANI experience for both patient and caregiver Identifying things that changed and things that are the same post-ANI for both patient and caregiver Using dialectics and developing posttraumatic growth Skill practice planning; individually, together, or both using RT mobile app
Specific	After NICU	Adjusting to ANI sequelae	Adjust values and priorities consistent with where you are now Acceptance and self-appreciation; positive psychology skills, ie, humor, optimism Continue skill practice; individually, together, or both using RT mobile app

Abbreviations: ANI, acute neurological injury; HADS, Hospital Depression and Anxiety Scale; NICU, neuroscience intensive care unit; PCL, PTSD Checklist–Civilian Version; RT, Recovering Together.

#### Health Education Control

The educational program mimicked the dose and duration of RT without teaching RT skills. The topics included education regarding the stress of the acute neurologic injury on the patient and caregiver (session 1); the importance of self-care (session 2); the stress associated with discharge and home adjustment (session 3); the importance of following up with medical recommendations (session 4); interpersonal stress (Session 5); and self-care (session 6).

### Study Outcomes

The primary outcomes were feasibility of recruitment and intervention delivery, credibility, and satisfaction. The secondary outcomes included depression and anxiety (measured by the Hospital Depression and Anxiety Scale), PTS (measured by the PTSD Checklist–Civilian Version), and intervention targets (ie, mindfulness, measured by the Cognitive and Affective Mindfulness Scale–Revised; coping, measured by the Measure of Current Status–Part A; and dyadic interpersonal interactions, measured by the Dyadic Relationship Scale). Main outcomes and targets were assessed at baseline, 6 weeks, and 12 weeks.

### Study Instruments

#### Demographic and Clinical Characteristics

Demographic data (Table 2) were collected at baseline. Patients’ ANI diagnosis, intubation history, and GCS score were collected from electronic medical records. Study staff assessed Mini-Mental State Examination and degree of functional disability (Barthel Index and Modified Rankin Scale).

**Table 2.** Baseline Demographic and Clinical Characteristics of Randomized Dyads, by Patients and Caregivers

Characteristic	No. (%)	
	RT (n = 29)	Control (n = 29)
**Baseline patient characteristics**		
Age, mean (SD) [range]	49.3 (16.7) [21-83]	50.1 (16.4) [24-81]
Sex		
Women	9 (31.0)	12 (41.4)
Men	20 (69.0)	17 (58.6)
Race		
White	25 (86.3)	24 (82.9)
Black or African American	2 (6.9)	2 (6.9)
Asian	1 (3.4)	1 (3.4)
>1 Race	1 (3.4)	1 (3.4)
Choose not to answer	0 (0.0)	1 (3.4)
Marital status		
Married or civil union	17 (58.7)	20 (69.0)
Single, never married	5 (17.3)	5 (17.2)
Divorced or separated	3 (10.3)	0
Living with partner	1 (3.4)	2 (7.0)
Widowed	1 (3.4)	1 (3.4)
Other	2 (6.9)	1 (3.4)
Employment status		
Employed full-time	18 (62.2)	12 (41.6)
Retired	5 (17.2)	8 (27.6)
Full-time or part-time student	1 (3.4)	1 (3.4)
Employed part-time	1 (3.4)	1 (3.4)
Unemployed	0 (0.0)	1 (3.4)
Homemaker	0 (0.0)	1 (3.4)
Other	4 (13.8)	4 (13.8)
Missing	0	1 (3.4)
Education		
Some high school, <grade 12	0	2 (6.9)
High school diploma, grade 12	5 (17.2)	6 (20.7)
Some college or associate degree	9 (31.0)	8 (27.6)
4-y college degree	3 (10.4)	4 (13.8)
Graduate or professional degree	12 (41.4)	8 (27.6)
Missing value	0	1 (3.4)
Diagnosis		
Vascular	11 (38.0)	13 (44.8)
Neoplasm	8 (27.6)	7 (24.1)
Spine	1 (3.4)	2 (7.0)
TBI	2 (6.9)	0 (0.0)
Seizure	2 (6.9)	0 (0.0)
Myasthenia gravis	0 (0.0)	2 (6.9)
Other	5 (17.2)	5 (17.2)
Cognitive status, mean (SD) [range]		
Mini-Mental State Examination	26.8 (2.0) [23-30]	25.9 (3.1) [13-29]
Barthel Index	60.0 (31.7) [0-100]	65.0 (30.0) [5-100]
Modified Rankin Scale	3.0 (0.91) [1-5]	3.0 (1.22) [1-5]
Glasgow Coma Scale score		
15	25 (86.2)	25 (86.2)
14	3 (10.3)	4 (13.8)
13	1 (3.4)	0
Intubated, other than postoperatively	5 (17.2)	1 (3.4)
**Caregiver baseline characteristics**		
Age, mean (SD) [range], y	52.4 (14.3) [20-75]	52.1 (14.9) [25-77]
Relationship to survivor		
Spouse or partner	22 (75.9)	24 (82.8)
Parent	4 (13.8)	3 (10.3)
Sibling	2 (6.9)	0 (0.0)
Other	1 (3.4)	2 (6.9)
Sex		
Women	22 (75.9)	17 (58.6)
Men	7 (24.1)	12 (41.4)
Race		
White	25 (86.2)	20 (69.0)
Black or African American	4 (13.8)	3 (10.4)
Asian	0	1 (3.4)
>1 Race	0	1 (3.4)
Choose not to answer	0	4 (13.8)
Employment status		
Employed full-time	14 (48.3)	18 (62.1)
Retired	6 (20.7)	4 (13.8)
Employed part-time	3 (10.3)	2 (6.9)
Homemaker	2 (7.0)	2 (6.9)
Full-time or part-time student	1 (3.4)	1 (3.4)
Other	3 (10.3)	2 (6.9)
Education		
Some high school, ie, <grade 12	1 (3.5)	1 (3.4)
High school diploma, ie, grade 12	4 (13.8)	5 (17.2)
Some college or associates degree	9 (31.0)	9 (31.0)
4-y college degree	6 (20.7)	7 (24.2)
Graduate or professional degree	9 (31.0)	7 (24.2)

Abbreviation: RT, Recovering Together; TBI, traumatic brain injury.

#### Depression and Anxiety

The HADS^34^ assesses symptoms of depression (7 items) and anxiety (7 items), with a range of 0 to 28 and a score greater than 7 depicting clinically significant symptoms.^35^ Lower scores represent negligible symptoms. Minimally important clinical differences (MCIDs) for each are 1.5 to 1.7.^36^

#### Posttraumatic Stress

The PCL-C^37^ assesses PTS symptom severity linked to ANI (17 items; range 17-85). Clinically significant symptoms are calculated based on the DSM *Diagnostic and Statistical Manual of Mental Disorders* algorithm, and the MCID is 10.^38^

#### Mindfulness, Coping Skills, and Dyadic Interactions

The Cognitive and Affective Mindfulness Scale-Revised (CAMS-R)^39^ assesses mindfulness (12 items; range, 12-48). The Measure of Current Status–Part A (MOCS-A)^40^ assesses coping (13 items; range, 0-65; 4 subscales: relaxation, awareness of tension, getting needs met, and coping confidence). The Dyadic Relationship Scale (DRS)^41^ has 2 subscales that assess positive (range, 6-24) and negative (range, 4-16 or 5-20) dyadic interactions.

#### Feasibility Markers

Feasibility Markers appear in Table 3. They include the feasibility of recruitment, ie, the percentage of dyads who screened in and agreed to participate among those who were approached in the neuroscience ICU; feasibility of randomization (acceptability), ie, the percentage of patients who were started within 1 group (ie, intervention or control) and completed posttreatment assessment among those randomized; feasibility of data collection, ie, the percentage of dyads who provided posttreatment and 3-month follow-up among those randomized; and adherence to session (adherence), ie, the percentage of dyads who completed 4 of 6 sessions among those randomized. Fidelity to condition was defined in 2 ways. First, it was defined as the percentage of clinicians who self-reported adherence to each study component using a predetermined session checklist among all clinicians. Second, it was defined as the percentage of clinicians whom 2 independent raters judged were adherent among a randomly selected 20% of the recorded sessions across all clinicians. Satisfaction was assessed at postintervention with the 3-item Client Satisfaction Questionnaire^42,43^ (CSQ-3; range, 0-12). Treatment expectancy and rationale credibility were assessed with the 6-item Credibility and Expectancy Questionnaire (CEQ),^44^ which has 2 subscales: cognitively based credibility and affectively based expectancy (range, 3-27).

**Table 3.** Feasibility, Acceptability, Fidelity, Satisfaction and Credibility

Marker	Proposed benchmark	Observed benchmark
Feasibility of recruitment	>70% dyads who screened in agree to participate	76%; 83 dyads screened in; of these, 20 declined consent after screening
Feasibility of randomization	>70% participants who start within 1 group complete post-test	RT: 100%; of 29 dyads who started RT, 29 had at least 1 member who completed posttreatment assessment (27 survivors and 29 caregivers)
		Control: 100%; of 29 dyads who started, 29 had at least 1 member complete posttreatment assessment (26 survivors and 29 caregivers)
Feasibility of data collection	>70% participants will provide posttreatment assessment; >70% participants will provide 3-mo follow-up data	Of 58 dyads who started the intervention, 53 survivors (91%) and 58 caregivers (100%) completed at least HADS and PCL posttreatment assessment
		Of 58 dyads who reached the 3-mo follow-up, 48 survivors (83%) and 51 caregivers (88%) completed at least HADS and PCL 3-mo follow-up
Adherence to sessions, ie, acceptability	>70% participants will complete 4 of 6 sessions.	50 participants (86%) completed 4 of 6 sessions
Fidelity to condition	>70% clinician-rated adherence checklists collected	100% clinician rated adherence checklists collected
	>70% sessions with 100% adherence (per clinician)	100% sessions with 100% adherence (per clinician)
	κ > 0.80 for 20% of the recorded sessions randomly selected and checked for adherence	κ = 0.98 agreement (2 independent raters) for 20% of recorded sessions randomly selected and checked for adherence
Satisfaction with treatment group	>70% participants endorse scores higher than the Client Satisfaction scale midpoint for both groups	RT: mean (SD), 10.65 (1.18); 57 of 58 (98%) endorsed scores >6
		Control: mean (SD), 9.87 (2.70); 58 of 58 (100%) endorsed scores >6
Credibility	>70% participants endorse scores higher than the Credibility Scale midpoint for both groups	RT: mean (SD), 22.0 (4.4); 47 of 58 (81%) endorsed scores >13.5
		Control: mean (SD), 20.3 (4.8); 46 of 58 (80%) endorsed scores >13.5
Expectancy	>70% participants endorse scores higher than the Expectancy Scale midpoint for both groups	RT: mean (SD), 19.0 (5.2); 49 of 58 (85%) endorsed scores >13.5
		Control: mean (SD), 19.5 (5.6) 51 of 58 (87%) endorsed scores >13.5

Abbreviations: HADS, Hospital Depression and Anxiety Scale; PCL, PTSD Checklist; RT, Recovering Together.

### Statistical Analysis

Analyses included randomized dyads who were eligible and completed at least 1 intervention session. We calculated standard univariate statistics to characterize the sample. Efficacy outcomes were analyzed in separate shared-baseline, mixed-model repeated-measure analysis of variance estimated by restricted maximum likelihood.^45^ The shared-baseline assumption reflects the data-generating process and, in combination with unstructured person-level covariance among repeated measures, makes a linear adjustment for the chance difference in baseline levels of the given outcome measure. Item nonresponse for instruments was mean imputed from other responses on the same assessment if at least 75% of items were nonmissing. Missing scores were accommodated in the mixed-model repeated-measure analyses of variance implicitly. Model estimates are unbiased, assuming that unobserved scores were missing at random conditional on nonmissing scores. Analyses were performed using SAS version 9.4 (SAS Institute). Inference was based on 2-tailed tests at *P* < .05 without correction for multiple comparisons, given the focus on feasibility and proof of concept in this pilot trial. Analyses were conducted separately for patients and caregivers.

## Results

A total of 58 dyads were randomized to RT (29 dyads [50.0%]; survivors: mean [SD] age, 49.3[16.7] years; 9 [31.0%] women; caregivers, mean [SD] age, 52.4 [14.3] years; 22 [75.9%] women) or control (29 dyads [50.0%]; survivors: mean [SD] age, 50.3 [16.4] years; 12 [41.3%] women; caregivers: mean [SD] age, 52.1 [14,9], 17 [58.6%] women) (Table 2). We found no clinically meaningful between-group differences (RT vs control) in demographic characteristics. We met or exceeded our a priori benchmarks for feasibility, data collection, acceptability, fidelity, credibility, expectancy, and satisfaction (Table 3). Of 83 dyads screened in, 20 declined consent after screening (76% recruitment). Of 29 dyads who started RT, 29 had at least 1 member complete the posttreatment assessment (100% randomization); of 29 dyads who started control, 29 had at least 1 member complete the posttreatment assessment (100% randomization). Regarding data collection, of 58 dyads who started the intervention, 53 survivors (91%) and 58 caregivers (100%) completed at least HADS and PCL after the intervention. A total of 50 participants completed 4 of 6 sessions (86% acceptability). Overall, all clinicians rated adherence (100% fidelity; κ = 0.98). In the intervention group, the mean (SD) satisfaction score was 10.65 (1.18), with 57 of 58 RT participants (98%) and 58 of 58 control participants [100%] with scores greater than 6; the mean (SD) credibility score was 20.3 (4.4), with 47 of 58 RT participants (81%) and 46 of 58 control participants [80%] with scores greater than 6; and the mean (SD) expectancy score was 19.0 (5.2), with 49 of 58 RT participants (85%) and 51 of 58 control participants (87%) with scores greater than 13.5.

By chance, patients and caregivers randomized to RT had substantially higher mean (SD) baseline PTS scores (among survivors: 41.6 [12.9] vs 29.3 [12.1]; difference, 12.3; among caregivers: 43.2 [10.7] vs 29.4 [12.0]; difference, 13.8) and anxiety scores (among survivors: 11.1 [4.7] vs 6.3 [4.0]; difference, 4.8; among caregivers: 13.2 [3.9] vs 7.0 [4.7]; difference, 6.2) than control participants. Caregivers randomized to RT also had substantially higher depression scores (7.8 [2.6] vs 4.8 [3.8]; difference, 3.0) than those in the control group.

Participation in RT vs control was associated with statistically significant improvement from baseline to postintervention in depression (among survivors: −4.0 vs −0.6; difference, −3.4; 95% CI, −5.6 to −1.3; *P* = .002; among caregivers: −3.8 vs 0.6; difference, −4.5; 95% CI, −6.7 to −2.3; *P* < .001) and anxiety (among survivors: −6.0 vs 0.3; difference, −6.3; 95% CI, −8.8 to −3.8; *P* < .001; among caregivers: −5.0 vs −0.9; difference, −4.1; 95% CI, −6.7 to −1.5; *P* = .002). Table 4 presents the unadjusted means, SDs, and ranges for patients and caregivers by condition across the study period. Treatment-dependent improvements in depression and anxiety exceeded the MCID for the HADS (ie, ≥1.5) for both patients and caregivers. Similarly, participation in RT vs control was associated with statistically significant improvement from baseline to postintervention in PTS (among survivors: −11.3 vs 1.0; difference, −12.3; 95% CI, −18.1 to −6.5; *P* < .001; among caregivers: −11.4 vs 5.0, difference, −16.4; 95% CI, −21.8 to −10.9; *P* < .001). Treatment-dependent improvements in PTS exceeded the MCID for the PCL (ie, ≥10) for both patients and caregivers. Improvements in depression, anxiety, and PTS sustained through the 12-week follow-up visit. Notably, patients in the control group had significantly worse PTS at 12 weeks (difference, 8.3; 95% CI, 2.8 to 13.7; *P* = .003) while those in RT remained stable (difference, 0.6; 95% CI, −4.6 to 5.8; *P* = .82). For caregivers, the control group had stable PTS at 12 weeks (difference, 1.1; 95% CI, −2.6 to 4.8; *P* = .55) while RT continued to improve (difference, −3.7; 95% CI, −7.3 to 0.0; *P* = .02) (eFigure in Supplement 2).

**Table 4.** Unadjusted Scores at Baseline, 6 Weeks, and 3 Months, Separately by Survivors, Caregivers, and Group

Outcome	Baseline		Posttreatment		3-mo Follow-up	
	Survivors	Caregivers	Survivors	Caregivers	Survivors	Caregivers
Depression, HADS						
RT						
No. (%)	29 (100)	29 (100)	27 (93.1)	29 (100)	26 (89.7)	26 (100)
Mean (SD) [range]	8.7 (4.2) [2.0-16.0]	7.8 (2.7) [3.0-15.0]	4.0 (4.1) [0-16.0]	3.28 (3.37) [0-12.0]	3.7 (3.3) [0-11.0]	2.42 (2.58) [0-11.0]
MEUC						
No. (%)	29 (100)	29 (100)	25 (86.2)	29 (100)	23 (79.3)	26 (100)
Mean (SD) [range]	7.1 (4.0) [1.0-18.0]	4.8 (3.8) [0-12.0]	7.2 (3.7) [0-14.0]	6.1 (5.4) [0-16.0]	7.7 (5.0) [0-16.0]	7.27 (5.28) [0-21.0]
Anxiety, HADS						
RT						
No. (%)	29 (100)	29 (100)	27 (93.1)	29 (100)	26 (89.7)	26 (89.7)
Mean (SD) [range]	11.1 (4.7) [3.0-20.0]	13.2 (3.9) [4.0-20.0]	3.3 (4.1) [0-19.0]	6.0 (3.8) 0-16.0]	4.7 (4.8) [0-17.0]	5.3 (3.8) [0-16.0]
MEUC						
No. (%)	29 (100)	29 (100)	26 (89.7)	29 (100)	23 (79.3)	26 (89.7)
Mean (SD) [range]	6.3 (4.0) [0-17.0]	7.0 (4.7) [0-17.0]	8.5 (5.2) [0-20.0]	8.1 (6.1) [0-21.0]	8.9 (6.0) [0-21.0]	9.62 (5.02) [0-17.0]
Posttraumatic stress, PCL-C						
RT						
No. (%)	29 (100)	29 (100)	27 (93.1)	29 (100)	26 (89.7)	26 (89.7)
Mean (SD) [range]	41.6 (12.9) [18.0-64.0]	43.2 (10.7) [26.0-61.0]	26.4 (10.8) [17.0-66.0]	30.3 (9.8) [24.0-65.0]	28.2 (10.1) [17.0-52.0]	27.3 (7.7) [17.0-47.0]
MEUC						
No. (%)	29 (100)	29 (100)	26 (89.7)	29 (100)	23 (79.3)	26 (89.7)
Mean (SD) [range]	29.3 (12.1) [18.0-63.0]	29.4 (12.0) [17.0-62.0]	34.6 (12.1) [17.0-58.0]	35.9 (16.6) [17.0-77.0]	41.7 (16.0) [17.0-70.0]	37.7 (16.1) [17.0-67.0]
Coping skills, MOCS-A						
RT						
No. (%)	29 (100)	28 (96.6)	24 (82.8)	25 (86.2)	25 (86.2)	24 (82.8)
Mean (SD) [range]	2.2 (0.8) [0.2-4.0]	2.3 (0.6) [0.6-3.2]	2.7 (0.8) [1.2-4.0]	2.4 (0.8) [1-3.9]	2.5 (0.7) [1.2-3.8]	2.5 (0.9) [0.78-3.9]
MEUC						
No. (%)	28 (96.6)	28 (96.6)	22 (75.9)	25 (86.2)	16 (55.2)	18 (62.1)
Mean (SD) [range]	2.6 (0.9) [0.8-3.8]	2.5 (0.9) [0.7-3.8]	2.6 (1.1) [0.7-4.0]	2.4 (1.2) [0-4.0]	2.2 (1.0) [0.8-3.8]	2.3 (1.0) [0.9-3.9]
Mindfulness, CAMS						
RT						
No. (%)	29 (100)	29 (100)	23 (79.3)	26 (89.7)	26 (89.7)	25 (86.2)
Mean (SD) [range]	34.2 (7.0) [17.5-48.0]	34.6 (5.8) [23.0-46.0]	36.7 (7.7) [23.0-48.0]	36.3 (6.5) [25.0-46.0]	35.5 (7.9) [23.0-48.0]	36.3 (7.5) [230-48.0]
MEUC						
No. (%)	29 (100)	29 (100)	21 (72.4)	26 (89.7)	18 (62.1)	19 (65.5)
Mean (SD) [range]	35.1 (7.8) [21.0-47.0]	36.4 (7.6) [20.0-47.0]	35.0 (10.7) [14.0-48.0]	36.7 (8.5) [17.0-48.0]	32.3 (9.0) [15.0-46.0]	33.3 (9.4) [15.0-48.0]
Dyadic strain, DRS						
RT						
No. (%)	28 (96.6)	28 (96.6)	21 (72.4)	26 (89.7)	25 (86.2)	23 (79.3)
Mean (SD) [range]	2.0 (0.8) [1.0-4.0]	2.0 (0.7) [1.0-3.4]	1.6 (0.6) [1.0-3.0]	2.01 (0.8) [1.0-3.8]	1.9 (0.8) [1.0-4.0]	2.2 (0.8) [1.0-4.0]
MEUC						
No. (%)	29 (100)	28 (96.6)	21 (72.4)	26 (89.7)	17 (58.6)	19 (65.5)
Mean (SD) [range]	2.0 (0.78) [1.0-3.8]	1.8 (0.7) [1.0-3.3]	2.0 (0.9) [1.0-3.5]	2.1 (0.9) [1.0-3.8]	2.0 (0.9) [1.0-4.0]	2.2 (0.8) [1.0-3.6]
Positive interactions, DRS						
RT						
No. (%)	28 (96.6)	28 (96.6)	21 (72.4)	25 (86.2)	24 (82.8)	23 (79.3)
Mean (SD) [range]	3.1 (0.5) [1.6-4.0]	3.0 (0.5) [2.2-4.0]	3.4 (0.6) [2.3-4.0]	3.3 (0.5) [2.3-4.0]	3.2 (0.6) [1.8-4.0]	3.2 (0.6) [1.8-4.0]
MEUC						
No. (%)	29 (100)	26 (89.7)	21 (72.4)	26 (89.7)	16 (55.2)	19 (65.5)
Mean (SD) [range]	3.2 (0.6) [2.0-4.0]	3.3 (0.4) [2.7-4.0]	2.97 (0.77) [1.2-4.0]	3.2 (0.7) [1.83-4]	2.82 (0.67) [1.8-4.0]	3.0 (0.7) [1.5-4.0]

Abbreviations: CAMS, Cognitive and Affective Mindfulness Scale–Revised; DRS, Dyadic Relationship Scale; HADS, Hospital Depression and Anxiety Scale; MOCS-A, Measure of Current Status–Part A; PCL-C, PTSD Checklist–Civilian Version; RT, Recovering Together.

Participation in RT was also associated with statistically significant improvement in positive dyadic interactions for survivors (0.2 vs −0.2; difference, 0.4; 95% CI, 0.0 to 0.8; *P* = .04) compared with the control group. It was not statistically significantly different for caregivers (0.2 vs 0; difference, 0.3; 95% CI, 0.0 to 0.6; *P* = .09). Participation in RT was associated with statistically significant increases from baseline to posttreatment in the use of positive coping skills for survivors (eg, assertiveness: difference, 0.6; 95% CI, 0.2 to 1.0; *P* = .002) and caregivers (eg, relaxation: difference, 0.6; 95% CI, 0.2 to 1.0; *P* = .008). We did not observe any between-group or within-group differences in other outcomes.

## Discussion

We observed excellent feasibility as evidenced by the ability to recruit participants quickly and randomize and retain them with low attrition rates. Participants believed RT would help improve their recovery trajectory and experienced high satisfaction with the program at both posttreatment and 12-week follow-up. Both intervention and control were delivered with high fidelity, and most survivors and caregivers attended at least 4 sessions. These findings suggest that RT content and delivery (in-person and video) is appropriate for this population.

Across the main outcomes of symptoms of depression, anxiety, and PTS, those randomized to RT showed better outcomes at posttreatment assessment than those randomized to control. Improvement in these outcomes were clinically meaningful and maintained at the 3-month follow-up visit. Improvements were observed for both survivors and caregivers. Of note, survivors randomized to RT continued to show significant improvement in PTS symptoms after the intervention ended. These results suggest that RT has durable effects over and above the time and attention from a clinician and general education information. This pilot trial successfully demonstrated the usefulness of this type of resilience intervention for patients with ANI and their caregivers. Across the intervention target outcomes, we observed improvement in positive dyadic interpersonal interaction for survivors. These improvements remained stable during the 3-month follow up. The observed improvement in dyadic interpersonal interaction supports RT content of teaching interpersonal communication skills.

Significant strengths of the study include strong measures of feasibility, including recruitment, retention, and fidelity, and significant improvement in outcomes. RT was developed to directly address limitations of prior negative trials in similar settings and with similar populations.^46,47^

### Limitations

This study has limitations. First, it was a pilot study, and the sample size was not large enough to detect small to moderate effect sizes. Lack of significant between-group differences in mindfulness and coping may reflect lack of power. Although the large and statistically significant effects for our emotional distress outcomes, despite the small sample size, suggest the strong potential of RT to benefit survivors and caregivers, findings require replication. Although the shared baseline model adjusts for these chance differences, future studies should use stratified randomization to ensure balanced samples. Second, although 4 therapists delivered RT and control, we did not assess for therapist effects. Third, we instructed participants to practice skills through the web-based application, but this was not formally collected. Fourth, within each dyad, at least 1 participant needed to endorse emotional distress to participate. While this approach is economical, we acknowledge that a small percentage of individuals who do not endorse emotional distress at hospitalization may develop it later. It is possible that we missed dyads who could have benefitted from RT. Several biomarkers more sensitively detect risk of chronic emotional distress among veterans^48^ and might be useful in accurately identifying dyads for interventions like RT. Fifth, we used a low cutoff score for clinically significant symptoms for depression and anxiety as an inclusionary criteria. The optimal cutoff score is not currently known, and researchers must balance specificity with sensitivity. Prior research is mixed, with some studies supporting a low cutoff score^4,6,7,18,35,49,50,51^ and others a higher cut-off score.^3,52^ Given the acute nature of neurological illness and the detrimental consequences of depression and anxiety symptoms that go unaddressed, we chose to use a lower score that allows for higher sensitivity so that we would not miss any dyads that might benefit from our program. Sixth, our sample mostly consisted of white individuals with higher educational attainment. While this reflects the sample composition of the neuroscience ICU at our institution, future studies should include racially, ethnically, and socioeconomically diverse samples of patients and care-partners in neuroscience ICUs with diverse resources and models of care.

## Conclusions

Findings from this pilot study support a fully powered RCT of RT vs control. Given that, to our knowledge, there are no evidence-based interventions to prevent chronic emotional distress in this population, there is a tremendous opportunity to reduce suffering and implement RT in the care of dyads in neuroscience ICUs across the country.

## Notes

Supplement 1. **Trial Protocol**

Supplement 2. **eFigure. **Adjusted Mean Scores for Depression (Hospital Anxiety and Depression Scale), Anxiety (Hospital Anxiety and Depression Scale), and PTS (PTSD Checklist–Civilian Version) by Randomization from the Shared Baseline

Supplement 3. **Data Sharing Statement**

## References

[1] Busl KM, Bleck TP, Varelas PN Neurocritical care outcomes, research, and technology: a review. JAMA Neurol. 2019;76(5):612-618. doi:10.1001/jamaneurol.2018.4407 30667464

[2] National Alliance for Caregiving Caregiving in America. Published 2015 Accessed March 30, 2020. https://www.caregiving.org/research/caregivingusa/

[3] Fumis RRL, Ranzani OT, Martins PS, Schettino G Emotional disorders in pairs of patients and their family members during and after ICU stay. PLoS One. 2015;10(1):e0115332. doi:10.1371/journal.pone.0115332 25616059 PMC4304779

[4] Shaffer KM, Riklin E, Jacobs JM, Rosand J, Vranceanu A-M Mindfulness and coping are inversely related to psychiatric symptoms in patients and informal caregivers in the neuroscience ICU: implications for clinical care. Crit Care Med. 2016;44(11):2028-2036. doi:10.1097/CCM.0000000000001855 27513536 PMC5069080

[5] Meyers EE, Shaffer KM, Gates M, Lin A, Rosand J, Vranceanu A-M Baseline resilience and posttraumatic symptoms in dyads of neurocritical patients and their informal caregivers: a prospective dyadic analysis. Psychosomatics. 2020;61(2):135-144. doi:10.1016/j.psym.2019.11.007 31928783 PMC7035969

[6] Meyers EE, Presciutti A, Shaffer KM, The impact of resilience factors and anxiety during hospital admission on longitudinal anxiety among dyads of neurocritical care patients without major cognitive impairment and their family caregivers. Neurocrit Care. Published online January 29, 2020s. doi:10.1007/s12028-020-00913-7 31997141 PMC12054369

[7] Meyers E, Lin A, Lester E, Shaffer K, Rosand J, Vranceanu A-M Baseline resilience and depression symptoms predict trajectory of depression in dyads of patients and their informal caregivers following discharge from the neuro-ICU. Gen Hosp Psychiatry. 2020;62:87-92. doi:10.1016/j.genhosppsych.2019.12.003 31887641 PMC6948176

[8] Lee S, Colditz GA, Berkman LF, Kawachi I Caregiving and risk of coronary heart disease in U.S. women: a prospective study. Am J Prev Med. 2003;24(2):113-119. doi:10.1016/S0749-3797(02)00582-2 12568816

[9] Ji J, Zöller B, Sundquist K, Sundquist J Increased risks of coronary heart disease and stroke among spousal caregivers of cancer patients. Circulation. 2012;125(14):1742-1747. doi:10.1161/CIRCULATIONAHA.111.057018 22415143

[10] Schulz R, Beach SR Caregiving as a risk factor for mortality: the Caregiver Health Effects Study. JAMA. 1999;282(23):2215-2219. doi:10.1001/jama.282.23.2215 10605972

[11] Beach SR, Schulz R, Williamson GM, Miller LS, Weiner MF, Lance CE Risk factors for potentially harmful informal caregiver behavior. J Am Geriatr Soc. 2005;53(2):255-261. doi:10.1111/j.1532-5415.2005.53111.x 15673349

[12] Turner-Stokes L, Hassan N Depression after stroke: a review of the evidence base to inform the development of an integrated care pathway—part 1: diagnosis, frequency and impact. Clin Rehabil. 2002;16(3):231-247. doi:10.1191/0269215502cr487oa 12017511

[13] Barreto BB, Luz M, Rios MNO, Lopes AA, Gusmao-Flores D The impact of intensive care unit diaries on patients’ and relatives’ outcomes: a systematic review and meta-analysis. Crit Care. 2019;23(1):411. doi:10.1186/s13054-019-2678-0 31842929 PMC6916011

[14] Wade DM, Mouncey PR, Richards-Belle A, ; POPPI Trial Investigators Effect of a nurse-led preventive psychological intervention on symptoms of posttraumatic stress disorder among critically ill patients: a randomized clinical trial. JAMA. 2019;321(7):665-675. doi:10.1001/jama.2019.0073 30776295 PMC6439605

[15] White DB, Cua SM, Walk R, Nurse-led intervention to improve surrogate decision making for patients with advanced critical illness. Am J Crit Care. 2012;21(6):396-409. doi:10.4037/ajcc2012223 23117903 PMC3547494

[16] Meyers EE, McCurley J, Lester E, Jacobo M, Rosand J, Vranceanu A-M Building resiliency in dyads of patients admitted to the neuroscience intensive care unit and their family caregivers: lessons learned from William and Laura. Cogn Behav Pract. 2020;27(3):321-335. doi:10.1016/j.cbpra.2020.02.001 32863700 PMC7454168

[17] McCurley JL, Funes CJ, Zale EL, Preventing chronic emotional distress in stroke survivors and their informal caregivers. Neurocrit Care. 2019;30(3):581-589. doi:10.1007/s12028-018-0641-6 30421266 PMC6958510

[18] Bannon S, Lester EG, Gates MV, Recovering together: building resiliency in dyads of stroke patients and their caregivers at risk for chronic emotional distress; a feasibility study. Pilot Feasibility Stud. 2020;6:75. doi:10.1186/s40814-020-00615-z 32509320 PMC7249683

[19] Streck BP, Wardell DW, LoBiondo-Wood G, Beauchamp JES Interdependence of physical and psychological morbidity among patients with cancer and family caregivers: review of the literature. Psychooncology. 2020;29(6):974-989. doi:10.1002/pon.5382 32227401

[20] Bakas T, Clark PC, Kelly-Hayes M, King RB, Lutz BJ, Miller EL; American Heart Association Council on Cardiovascular and Stroke Nursing and the Stroke Council Evidence for stroke family caregiver and dyad interventions: a statement for healthcare professionals from the American Heart Association and American Stroke Association. Stroke. 2014;45(9):2836-2852. doi:10.1161/STR.0000000000000033 25034718

[21] Dimeff L, Koerner K Dialectical Behavior Therapy in Clinical Practice: Applications Across Disorders and Settings. Guilford Press; 2007 Accessed April 8, 2020. https://www.guilford.com/books/Dialectical-Behavior-Therapy-in-Clinical-Practice/Dimeff-Koerner/9781572309746/reviews

[22] Lin A, Vranceanu A-M, Guanci M, Salgueiro D, Rosand J, Zale EL Gender differences in longitudinal associations between intimate care, resiliency, and depression among informal caregivers of patients surviving the neuroscience intensive care unit. Neurocrit Care. 2020;32(2):512-521. doi:10.1007/s12028-019-00772-x 31270671

[23] Browne RH On the use of a pilot sample for sample size determination. Stat Med. 1995;14(17):1933-1940. doi:10.1002/sim.4780141709 8532986

[24] Lancaster GA, Dodd S, Williamson PR Design and analysis of pilot studies: recommendations for good practice. J Eval Clin Pract. 2004;10(2):307-312. doi:10.1111/j..2002.384.doc.x 15189396

[25] Rounsaville B, Carroll K. A stage model of behavioral therapies research: getting started and moving on from stage I. Clin Psychol. 2001;8(2):133-142. doi:10.1093/clipsy.8.2.133

[26] Schulz KF, Altman DG, Moher D; CONSORT Group CONSORT 2010 statement: updated guidelines for reporting parallel group randomised trials. PLoS Med. 2010;7(3):e1000251. doi:10.1371/journal.pmed.1000251 20352064 PMC2844794

[27] Jain S, Iverson LM Glasgow Coma Scale. In: StatPearls. StatPearls Publishing; 2020 Accessed April 6, 2020. https://www.ncbi.nlm.nih.gov/books/NBK513298/30020670

[28] Harris PA, Taylor R, Thielke R, Payne J, Gonzalez N, Conde JG Research electronic data capture (REDCap)—a metadata-driven methodology and workflow process for providing translational research informatics support. J Biomed Inform. 2009;42(2):377-381. doi:10.1016/j.jbi.2008.08.010 18929686 PMC2700030

[29] Barclay-Goddard R, King J, Dubouloz C-J, Schwartz CE; Response Shift Think Tank Working Group Building on transformative learning and response shift theory to investigate health-related quality of life changes over time in individuals with chronic health conditions and disability. Arch Phys Med Rehabil. 2012;93(2):214-220. doi:10.1016/j.apmr.2011.09.010 22289229

[30] Cook WL, Kenny DA The actor-partner interdependence model: a model of bidirectional effects in developmental studies. Int J Behav Dev. 2005;29(2):101-109. doi:10.1080/01650250444000405

[31] Shields CG, King DA, Wynne LC Interventions with later life families. In: Mikesell RH, Lusterman D-D, McDaniel SH, eds. Integrating Family Therapy: Handbook of Family Psychology and Systems Theory. American Psychological Association; 1995:141-158. doi:10.1037/10172-008

[32] Savini S, Buck HG, Dickson VV, Quality of life in stroke survivor-caregiver dyads: a new conceptual framework and longitudinal study protocol. J Adv Nurs. 2015;71(3):676-687. doi:10.1111/jan.12524 25186274

[33] Bonanno GA, Galea S, Bucciarelli A, Vlahov D What predicts psychological resilience after disaster? the role of demographics, resources, and life stress. J Consult Clin Psychol. 2007;75(5):671-682. doi:10.1037/0022-006X.75.5.671 17907849

[34] Bjelland I, Dahl AA, Haug TT, Neckelmann D The validity of the Hospital Anxiety and Depression Scale—an updated literature review. J Psychosom Res. 2002;52(2):69-77. doi:10.1016/S0022-3999(01)00296-3 11832252

[35] Hansson M, Chotai J, Nordstöm A, Bodlund O Comparison of two self-rating scales to detect depression: HADS and PHQ-9. Br J Gen Pract. 2009;59(566):e283-e288. doi:10.3399/bjgp09X454070 19761655 PMC2734374

[36] Puhan MA, Frey M, Büchi S, Schünemann HJ The minimal important difference of the Hospital Anxiety and Depression Scale in patients with chronic obstructive pulmonary disease. Health Qual Life Outcomes. 2008;6:46. doi:10.1186/1477-7525-6-46 18597689 PMC2459149

[37] Weathers FW, Lits BT, Herman DS, Huska JA, Keane TM The PTSD Checklist (PCL): Reliability, validity, and diagnostic utility. Annual convention of the International Society for Traumatic Stress Studies; San Antonio, TX; 1993.

[38] Blanchard EB, Jones-Alexander J, Buckley TC, Forneris CA Psychometric properties of the PTSD Checklist (PCL). Behav Res Ther. 1996;34(8):669-673. doi:10.1016/0005-7967(96)00033-2 8870294

[39] Feldman G, Hayes A, Kumar S, Greeson J, Laurenceau J-P Mindfulness and emotion regulation: the development and initial validation of the Cognitive and Affective Mindfulness Scale-Revised (CAMS-R). J Psychopathol Behav Assess. 2006;29(3):177. doi:10.1007/s10862-006-9035-8

[40] Carver S Measure of Current Status (MOCS). Accessed September 11, 2020. https://www.midss.org/content/measure-current-status-mocs

[41] Sebern MD, Whitlatch CJ Dyadic relationship scale: a measure of the impact of the provision and receipt of family care. Gerontologist. 2007;47(6):741-751. doi:10.1093/geront/47.6.741 18192628

[42] Attkisson CC, Greenfield T The Client Satisfaction Questionnaire (CSQ) Scales: Outcome Assessment in Clinical Practice. Williams & Wilkins; 1995:2-10.

[43] Larsen DL, Attkisson CC, Hargreaves WA, Nguyen TD Assessment of client/patient satisfaction: development of a general scale. Eval Program Plann. 1979;2(3):197-207. doi:10.1016/0149-7189(79)90094-6 10245370

[44] Devilly GJ, Borkovec TD Psychometric properties of the credibility/expectancy questionnaire. J Behav Ther Exp Psychiatry. 2000;31(2):73-86. doi:10.1016/S0005-7916(00)00012-4 11132119

[45] Liang K-Y, Zeger SL Longitudinal data analysis of continuous and discrete responses for pre-post designs. Sankhyā: Indian J Stat, Ser B. 2000;62(1):134-148. doi:10.2307/25053123

[46] White DB, Angus DC, Shields A-M, ; PARTNER Investigators A randomized trial of a family-support intervention in intensive care units. N Engl J Med. 2018;378(25):2365-2375. doi:10.1056/NEJMoa1802637 29791247

[47] Glass TA, Berkman LF, Hiltunen EF, The Families In Recovery From Stroke Trial (FIRST): primary study results. Psychosom Med. 2004;66(6):889-897. doi:10.1097/01.psy.0000146326.01642.ca 15564354

[48] Dean KR, Hammamieh R, Mellon SH, ; PTSD Systems Biology Consortium Multi-omic biomarker identification and validation for diagnosing warzone-related post-traumatic stress disorder. Mol Psychiatry. 2019. Published online September 10, 2019. doi:10.1038/s41380-019-0496-z 31501510 PMC7714692

[49] Olssøn I, Mykletun A, Dahl AA The Hospital Anxiety and Depression Rating Scale: a cross-sectional study of psychometrics and case finding abilities in general practice. BMC Psychiatry. 2005;5:46. doi:10.1186/1471-244X-5-46 16351733 PMC1343544

[50] Lester EG, Silverman IH, Gates MV, Lin A, Vranceanu A-M Associations between gender, resiliency factors, and anxiety in neuro-ICU caregivers: a prospective study. Int J Behav Med. 2020. doi:10.1007/s12529-020-09907-3 32488793

[51] Singer S, Kuhnt S, Götze H, Hospital Anxiety and Depression Scale cutoff scores for cancer patients in acute care. Br J Cancer. 2009;100(6):908-912. doi:10.1038/sj.bjc.6604952 19240713 PMC2661775

[52] Pochard F, Darmon M, Fassier T, ; French FAMIREA study group Symptoms of anxiety and depression in family members of intensive care unit patients before discharge or death: a prospective multicenter study. J Crit Care. 2005;20(1):90-96. doi:10.1016/j.jcrc.2004.11.004 16015522

