# Supplementary material for: Feasibility and Efficacy of a Resiliency Intervention for the Prevention of Chronic Emotional Distress Among Survivor-Caregiver Dyads Admitted to the Neuroscience Intensive Care Unit: A Randomized Clinical Trial
Source: JAMA Netw Open. 2020 Oct 14;3(10):e2020807. doi: 10.1001/jamanetworkopen.2020.20807 (PMC7557506; doi:10.1001/jamanetworkopen.2020.20807)
Supplement: Supplement 1. — Trial Protocol [file jamanetwopen-e2020807-s001.pdf]

Partners Human Research Committee
Partners HealthCare
399 Revolution Drive, Suite 710
Somerville, MA 02145
Tel: 857-282-1900
Fax: 857-282-5693

Notification of IRB Review

Protocol #: 2018P002187

Date:	October 26, 2018
To:	Vranceanu, Ana-Maria,<br>MGH<br>Partners > MGH > Psychiatry
From:	Partners Human Research Committee<br>399 Revolution Drive, Suite 710<br>Somerville, MA 02145
Title of Protocol:	Recovering Together: Building resiliency in dyads in patients admitted to the Neuroscience Intensive Care Unit (NICU) and their caregivers
Version/Number:	1
Version Date:	09/24/2018
IRB Review Type:	Expedited
Expedited Category/ies:	(4) Collection of data through noninvasive procedures (not involving general anesthesia or sedation) routinely employed in clinical practice, excluding procedures involving x-rays or microwaves. Where medical devices are employed, they must be cleared/approved for marketing. (Studies intended to evaluate the safety and effectiveness of the medical device are not generally eligible for expedited review, including studies of cleared medical devices for new indications.) Examples...<br><br>(5) Research involving materials (data, documents, records, or specimens) that have been collected, or will be collected solely for nonresearch purposes (such as medical treatment or diagnosis). (Note: Some research in this category may be exempt from the HHS regulations for the protection of human subjects. 45 CFR 46.101 (b)(4). This listing refers only to research that is not exempt.)<br><br>(7) Research on individual or group characteristics or behavior (including, but not limited to, research on perception, cognition, motivation, identity, language, communication, cultural beliefs or practices, and social behavior) or research employing survey, interview, oral history, focus group, program evaluation, human factors evaluation, or quality assurance methodologies. (Note: Some research in this category may be exempt from the HHS regulations for the protection of human subjects. 45 CFR 46.101 (b)(2) and (b)(3). This listing refers only to research that is not exempt.)
IRB Approval Date:	10/26/2018

Partners Human Research Committee
Partners HealthCare
399 Revolution Drive, Suite 710
Somerville, MA 02145
Tel: 857-282-1900
Fax: 857-282-5693

Approval/Activation Date:	10/26/2018
**IRB Expiration Date:**	**10/26/2019**

This project has been reviewed and approved by the **PHS IRB**. During the review of this project, the IRB specifically considered (i) the risks and anticipated benefits, if any, to subjects; (ii) the selection of subjects; (iii) the procedures for obtaining and documenting informed consent; (iv) the safety of subjects; and (v) the privacy of subjects and confidentiality of the data.

Please note that if an IRB member had a conflict of interest with regard to the review of this project, consistent with IRB policies and procedures, the member was required to recuse him/herself and, if applicable, leave the room during the discussion and vote on this project except to provide information requested by the IRB.

The following protocol documents have been approved and supporting documents noted by the IRB:

Protocol summary (v. 10/17/18)

Detailed protocol (v. 10/11/18)

Schema

Consent form

Instruments/questionnaires (12)

Subject reimbursement form

<u>**ANCILLARY COMMITTEES**</u>

1. **Nursing (MGH) : Approved**

As Principal Investigator, you are responsible for ensuring that this project is conducted in compliance with all applicable federal, state and local laws and regulations, institutional policies, and requirements of the IRB, which include, but are not limited to, the following:

1. Submission of any and all proposed changes to this project (e.g., protocol, recruitment materials, consent form, status of the study, etc.) to the IRB for review and approval prior to initiation of the change(s), <u>except</u> where necessary to eliminate apparent immediate hazards to the subject(s). Changes made to eliminate apparent immediate hazards to subjects must be reported to the IRB as an unanticipated problem.
2. Submission of continuing review submissions for re-approval of the project prior to expiration of IRB approval and a final continuing review submission when the project has been completed.

Partners Human Research Committee
Partners HealthCare
399 Revolution Drive, Suite 710
Somerville, MA 02145
Tel: 857-282-1900
Fax: 857-282-5693

3. Submission of any and all unanticipated problems, including adverse event(s) in accordance with the IRB's policy on reporting unanticipated problems including adverse events.
4. Obtaining informed consent from subjects or their legally authorized representative prior to initiation of research procedures when and as required by the IRB and, when applicable, documenting informed consent current IRB approved consent form(s) with the IRB-approval stamp in the document footer.
5. Informing all investigators and study staff listed on the project of changes and unanticipated problems, including adverse events, involving risks to subjects or others.
6. When investigator financial disclosure forms are required, submitting updated financial disclosure forms for yourself and for informing all site responsible investigators, co-investigators and any other members of the study staff identified by you as being responsible for the design, conduct, or reporting of this research study of their obligation to submit updated Investigator Financial Disclosure Forms for this protocol to the IRB if (a) they have acquired new financial interests related to the study and/or (b) any of their previously reported financial interests related to the study have changed.

IMPORTANT REMINDER: THE IRB HAS THE AUTHORITY TO TERMINATE PROJECTS THAT ARE NOT IN COMPLIANCE WITH THESE REQUIREMENTS.

Questions related to this project may be directed to **Ednice, Monteiro | Tel: 282-1916 | Email: EEMONTEIRO@PARTNERS.ORG**

cc:

Ana-Maria, Vranceanu, , Psychiatry, Psychiatry, Principal Investigator

Ann, Lin, , Psychiatry, Psychiatry, Research Coordinator/Manager

Melissa, Gates, , Psychiatry, Psychiatry

PARTNERS HUMAN RESEARCH COMMITTEE
PROTOCOL SUMMARY

Answer all questions accurately and completely in order to provide the PHRC with the relevant information to assess the risk-benefit ratio for the study. Do not leave sections blank.

PRINCIPAL/OVERALL INVESTIGATOR

Ana-Maria Vranceanu, Ph.D.

PROTOCOL TITLE

Recovering Together: Building resiliency in dyads in patients admitted to the Neuroscience Intensive Care Unit (NICU) and their caregivers

FUNDING

National Institute of Nursing Research

VERSION DATE

October 17th, 2018

SPECIFIC AIMS

Concisely state the objectives of the study and the hypothesis being tested.

The current study has the following specific aims:

1) To determine the feasibility of recruitment, feasibility of program delivery, program credibility, and program satisfaction using evidence-based benchmarks.

   Hypothesis 1: We hypothesize that > 75% of the dyads approached will agree to participate.
   Hypothesis 2: We hypothesize that > 75% of dyads who start the intervention will complete at least 4 sessions.
   Hypothesis 3: We hypothesize that > 75% of participants will report average credibility (Credibility and Expectancy Questionnaire) scores greater than the scale's midpoint.
   Hypothesis 4: We hypothesize that > 75% participants will report average satisfaction (Client Satisfaction Scale) scores greater than the scale's midpoint.

2) To demonstrate a proof of concept that the Recovering Together program can sustainably improve emotional distress [Hospital Anxiety and Depression Scale; HADS], Post-Traumatic Symptoms (PTS) [PCL-S], resiliency variables (mindfulness, coping, social support and self-efficacy) and interpersonal factors (interpersonal bond).

   Hypothesis 1: We hypothesize that participation in the Recovering Together Program will be associated with a more potent decrease in emotional distress and PTS compared to participation in the educational program (control), and that these improvements will maintain at 3 month follow up.
   Hypothesis 2: We hypothesize that Recovering Together Program will be associated with a more potent increase in resiliency variables (mindfulness, coping, social support, self-

efficacy) and interpersonal factors (interpersonal bond) compared to participation in the educational program, and that these improvements will maintain at 3 month follow up.

BACKGROUND AND SIGNIFICANCE

Provide a brief paragraph summarizing prior experience important for understanding the proposed study and procedures.

Acute neurological illnesses (ANIs) are common, costly and often lead to long-term disability. ANIs are biologically distinct injuries that disrupt the normal function of the brain. The most common ANIs in Neuroscience Intensive Care Units (NICU) include cerebrovascular (stroke/hemorrhage and brain aneurysm), structural (tumors and lesions/brain masses), and traumatic (TBI) brain injuries. NICU admissions for ANIs are prevalent (e.g., 795,000 acute stroke/year; 275,000 acute TBI/year) and costly; post NICU prolonged rehabilitation is common.
ANIs are associated with chronic emotional distress in both patients (pts) and caregivers (cgs). Although biologically heterogeneous, ANIs are unified by sudden onset, and substantial emotional distress in both pts (e.g., 12-43% anxiety; 10-58% depression; 20-29% post-traumatic stress PTS) and family cgs (27-60% depression, anxiety or PTS). These symptoms often become chronic and treatment resistant.
Pt and cg factors interact and influence physical and emotional outcomes in both pts and cgs. Post ANI emotional distress is associated with pts' poor medical adherence, slower recovery, higher mortality, and need of more caregiving assistance, which further increase cgs' distress and own risk for morbidity and mortality; in turn cgs' emotional distress interferes with ability to provide high-quality care to pts and negatively impacts pts' outcomes.
Current management of ANIs does not meet the psychological needs of pts and cgs for 3 reasons. First, although recognition of the emotional burden associated with NICU admission has increased, and some NICUs have social workers available to assist pts and cgs, there are no formal screening methods for emotional distress routinely integrated in practice during hospitalization, when the primary focus is on medical care and survival; further, there are no formal evidence-based treatments integrated within the medical care. When social workers are included to help pts and cgs, the care is brief and occurs only during hospitalization. When referrals to mental health services are provided to families at discharge, few will access additional treatment due to burden associated with traveling outside of home. Second, psychosocial interventions available for ANI pts or cgs are limited in that they are delivered when symptoms are already chronic, address only one emotional illness (e.g., depression or anxiety or PTS), and/or are focused on a *single member* of the pt-cg dyad. Even interventions labeled as "dyadic," which include pts and cgs, typically address only the pts' needs and do not focus on cg outcomes or on the dyad's interpersonal communication and bond/relationship. These interventions are not consistent with the *dyadic framework* which specifies that dyadic interventions should account for the interdependence between pt and cg psychosocial factors including their interpersonal bond by ensuring that both pts and cgs attend each session together, and by targeting improvement in outcomes for both pts and cgs. Third, most interventions are delivered using uniform protocols. However, the needs of ANI dyads are heterogeneous due to varying levels of post ANI impairment, identity of the cg, context and stage of life. A recent systematic review urged for the development of dyadic interventions that address the needs of *both* pts and cgs and are tailored to the specific needs of each ANI dyad.

We developed the first dyadic skills-based intervention – Recovering Together - to prevent chronic heightened emotional distress in at risk ANI pt-cg dyads. The “Recovering Together” program is informed by the theoretical response-shift framework of adaptation to acute illness (successful adaptation implies recalibration of values and life goals), the family strength vulnerability model (within dyads relational systems have strengths and weaknesses in how they cope with life events), the dyadic longitudinal model (distress travel from one member of the dyad to the other across time), the APIM model, and the resiliency framework**.** The program is in line with recent recommendations for skill-based interventions for critical care patients, and uses preliminary data collected by our team for the past 3 years. The intervention teaches pts and cgs resiliency factors that are associated with well-being after trauma for both pts and cgs: *mindfulness* – the ability to stay present and defer judgment in the face of adversity; *coping*– the arsenal and application of one’s behavioral, cognitive, and emotional strategies to manage stress; *social support* –empathetic interpersonal interactions that meet one’s emotional and functional needs; *self-efficacy* – perceived ability to adapt under adversity and *positive dyadic interpersonal communication to increase interpersonal bond*. Informed by the aforementioned theoretical models, our conceptual model hypothesizes that by teaching both members of the dyad resiliency and interpersonal communication skills (e.g., Recovering Together) we will be able to sustainably decrease emotional distress in both members of the dyad.

RESEARCH DESIGN AND METHODS

Briefly describe study design and anticipated enrollment, i.e., number of subjects to be enrolled by researchers study-wide and by Partners researchers. Provide a brief summary of the eligibility criteria (for example, age range, gender, medical condition). Include any local site restrictions, for example, “Enrollment at Partners will be limited to adults although the sponsor’s protocol is open to both children and adults.”

The current study will be a two arm, feasibility pilot RCT.

We will randomize a total of 80 dyads (40 dyads in each arm) to either a psychosocial intervention or to an educational program in the Neuro ICU.

Eligible dyads (pts and cgs) must meet the following inclusion criteria:

1) Age 18 or older
2) English fluency and literacy
3) Access to high speed internet for video sessions
4) Pt with an informal cg (family or friend who provides unpaid care) available and willing to participate
5) Hospitalized with an ANI within 1-2 weeks (pt) OR primary cg of a pt currently admitted with an ANI
6) Either pt <u>or</u> cg have clinically significant symptoms of depression, anxiety, and/or PTS

One or more of the following exclusion criteria will render a pt ineligible:

1) Permanent or severe cognitive impairment severe enough to impede participation – This will be determined by nurses through an assessment conducted as part of usual

care and that includes the MMSE (score of <23) and GCS (score of <10). Nurses are trained and use these measures as part of NICU care.

2) Dyads where the pt is anticipated to die or to never be able to participate due to medical sequelae. This will be determined by nurses.

This exclusionary criterion is already used in our IRB approved prospective study with this population.

Briefly describe study procedures. Include any local site restrictions, for example, “Subjects enrolled at Partners will not participate in the pharmacokinetic portion of the study.” Describe study endpoints.

Study procedures:

Participants will be referred to the study by the nursing team who will ensure that patients are medically and cognitively able to participate (see above). Eligible dyads will next be screened, consented, and enrolled by the research assistant.

After enrollment, subjects will be randomized to either the newly developed psychosocial intervention or to the educational program (control). Dyads will be randomly assigned by using a random number sequence generator to ensure comparability between groups. Randomization will be developed by the statistician, without any input from the rest of the team. All subjects will be given baseline psychological and behavioral assessments that will assess depression, anxiety, PTSD symptoms, and other psychological constructs. All assessments will be administered via computer using the REDCap secure data collection system.

In the skills-based intervention group, sessions focus on developing skills to cope and manage ANI related stressors. The intervention will be tailored consistent with AHA recommendations for ANI skills-based interventions and will include 2 general and 4 specific modules. It is anticipated that the intervention will have 6 sessions with 2 general sessions delivered, in person if possible, within the NICU, or through live video using Vidyo if patients leave the hospital before sessions occur, and 4 tailored specific sessions (chosen from by the dyads from 6 available modules) to be delivered via live video using Vidyo.

Those in the educational program will receive general health information that mimics the Recovering Together Program, but without teaching any of the resiliency or interpersonal communication skills that are hypothesized to be responsible for improvement in emotional distress. There will also be 6 sessions, 2 in-person dyadic visits in the NICU and 4 dyadic virtual visits following discharge. The educational program group will not have the opportunity to specify which modules they would like to take; the modules will be predetermined. All participants will receive medical care as determined by their medical team.

Both groups will continue with their current care with addition to the educational programs provided.

Both groups will complete post-treatment psychological and behavioral assessments (identical to those administered at baseline) to be administered immediately after completion of the course

and again at three months after completion of the course in order to measure long term outcomes. They will also be asked questions about the perceived efficacy of the intervention.

Study Endpoints:

1) Is the psychosocial skills-based intervention feasible, usable, and accepted by pts and cgs in the ICU?

2) Is the intervention associated with improvement in depression, anxiety and PTSD?

3) Is the effect of the skills-based intervention for pts and cgs in the ICU durable at 3 month follow up?

We will assess feasibility, usability, and acceptability by the enrolment numbers, participant completion in at least 4 out of 6 sessions, and the questionnaires, the Credibility and Expectancy and Client Satisfaction Scale. These will be the primary outcomes. The PCL-S, HADS, and the resiliency measures will serve as the secondary outcomes.

For studies involving treatment or diagnosis, provide information about standard of care at Partners (e.g., BWH, MGH) and indicate how the study procedures differ from standard care. Provide information on available alternative treatments, procedures, or methods of diagnosis.

NA

Describe how risks to subjects are minimized, for example, by using procedures which are consistent with sound research design and which do not unnecessarily expose subjects to risk or by using procedures already being performed on the subject for diagnostic or treatment purposes.

All study staff will complete required Partners human subjects trainings prior to the start of study procedures. In order to preserve confidentiality of participants, study data will not be linked to any identifying information; rather, study ID numbers will be assigned and used to identify participants. All study forms will be stored in locked storage spaces, to which only study staff will have access. All interventionists and assessors will have advanced training in clinical interviewing and assessment. Participants will be informed that they may refuse to answer questions that make them feel uncomfortable.

Describe explicitly the methods for ensuring the safety of subjects. Provide objective criteria for removing a subject from the study, for example, objective criteria for worsening disease/lack of improvement and/or unacceptable adverse events. The inclusion of objective drop criteria is especially important in studies designed with placebo control groups.

There is no risk of physical injury to participants. If a participant is judged to be suicidal at any time during participation, the interventionist will refer him or her to appropriate services, including the Acute Psychiatry Service at MGH if deemed appropriate.

FORESEEABLE RISKS AND DISCOMFORTS

> Provide a brief description of any foreseeable risks and discomforts to subjects. Include those related to drugs/devices/procedures being studied and/or administered/performed solely for research purposes. In addition, include psychosocial risks, and risks related to privacy and confidentiality. When applicable, describe risks to a developing fetus or nursing infant.

Participants may feel uncomfortable completing various psychological questionnaires. As in any research study, there is a small risk that confidentiality may be breached; all efforts to minimize this risk will be taken, as outlined above. In the unlikely event that participants will become suicidal during the duration of the study, the research assistant will contact the PI and appropriate clinical intervention will be executed.

EXPECTED BENEFITS

> Describe both the expected benefits to individual subjects participating in the research and the importance of the knowledge that may reasonably be expected to result from the study. Provide a brief, realistic summary of potential benefits to subjects, for example, “It is hoped that the treatment will result in a partial reduction in tumor size in at least 25% of the enrolled subjects.” Indicate how the results of the study will benefit future patients with the disease/condition being studied and/or society, e.g., through increased knowledge of human physiology or behavior, improved safety, or technological advances.

Participants may not benefit from the study directly. However, knowledge from this research study may benefit others by enhancing our understanding of the role of psychosocial skills-based interventions in treating future pts and cgs within the NICU. All participants will receive $20 for the completion of each of the 3 assessment points.

EQUITABLE SELECTION OF SUBJECTS

> The risks and benefits of the research must be fairly distributed among the populations that stand to benefit from it. No group of persons, for example, men, women, pregnant women, children, and minorities, should be categorically excluded from the research without a good scientific or ethical reason to do so. Please provide the basis for concluding that the study population is representative of the population that stands to potentially benefit from this research.

All subjects who satisfy the inclusion/exclusion criteria are eligible for enrollment in this study regardless of sex, race or ethnicity.

> When people who do not speak English are excluded from participation in the research, provide the scientific rationale for doing so. Individuals who do not speak English should not be denied participation in research simply because it is inconvenient to translate the consent form in different languages and to have an interpreter present.

Only participants who can read and speak English will be included in the current study as not all assessment measures have been validated for use in non-English speaking populations.

For guidance, refer to the following Partners policy:
Obtaining and Documenting Informed Consent of Subjects who do not Speak English
https://partnershealthcare-public.sharepoint.com/ClinicalResearch/Non-English_Speaking_Subjects.1.10.pdf

RECRUITMENT PROCEDURES

Explain in detail the specific methodology that will be used to recruit subjects. Specifically address how, when, where and by whom subjects will be identified and approached about participation. Include any specific recruitment methods used to enhance recruitment of women and minorities.

Potential participants will be referred for study participation by their medical teams (nurses). The medical team will assess whether the ANI patients are able to consent, consistent with medical presentation. Nurses will be administering the MMSE and GSC to all patients and will refer only dyads where the patients' scores are higher than the established cut off scores on these measures. Pts and their respective cgs who are able to consent and who express interest in the study will speak with a study team member to learn more about the study and be screened for eligibility; those who wish to participate will complete consent. If cgs cannot be reached in person in the hospital, they will be contacted via phone for screening and completed of informed consent. Cgs will be given a copy of the consent form to review while discussing the study over the telephone. We will fax or securely e-mail the consent form prior to obtaining consent. Cgs will return a signed copy of the consent form.

Patients and caregivers will be considered enrolled when they sign the consent form with the study staff. These procedures will be completed in a private medical space.

Participants will be explicitly informed that this intervention is a research study that does not constitute individualized, personal care. The intervention is a broad-based method of training that is not tailored to any particular individual. Should any participant seek formal mental healthcare, study staff will refer them to either MGH Psychiatry, as appropriate.

All dyads will be recruited within 1-2 weeks of pt's hospitalization. The pt's medical team will also alert the research assistant whether they anticipate that the patient might be able to participate in the study at a future point during the hospital stay. In situations where patients are unable to consent due to the severity of the stroke, we will enroll the informal cg and return to enroll the cg as soon as their mental capacity improves. The research assistant will not approach patients who are not mentally or physically capable to participate.

Eligible cases may also be identified by daily screening of Epic admission reports.

We will not include patients who do not have a caregiver. Informal caregivers will be designated by the health care proxy and verbally confirmed by the patient. Nurses will assist the study team with identifying the patient's health care proxy.

Provide details of remuneration, when applicable. Even when subjects may derive medical benefit from participation, it is often the case that extra hospital visits, meals at the hospital, parking fees or other inconveniences will result in additional out-of-pocket expenses related to study participation. Investigators may wish to consider providing reimbursement for such expenses when funding is available

All participants will receive $20 compensation for completing each of the 3 assessment points.

For guidance, refer to the following Partners policies:

Recruitment of Research Subjects
https://partnershealthcare-public.sharepoint.com/ClinicalResearch/Recruitment_Of_Research_Subjects.pdf

Guidelines for Advertisements for Recruiting Subjects
https://partnershealthcare-public.sharepoint.com/ClinicalResearch/Guidelines_For_Advertisements.1.11.pdf

Remuneration for Research Subjects
https://partnershealthcare-public.sharepoint.com/ClinicalResearch/Remuneration_for_Research_Subjects.pdf

CONSENT PROCEDURES

Explain in detail how, when, where, and by whom consent is obtained, and the timing of consent (i.e., how long subjects will be given to consider participation). For most studies involving more than minimal risk and all studies involving investigational drugs/devices, a licensed physician investigator must obtain informed consent. When subjects are to be enrolled from among the investigators' own patients, describe how the potential for coercion will be avoided.

After determining eligibility study staff will meet with potential dyads to review the informed consent document. After the document has been reviewed, study staff will answer any and all questions the pt or cg may have. Once all questions have been addressed, each member of the dyad will sign informed consent form, which will include a description of all study procedures, information about potential risks and benefits of participation, and study contact information (including that of the IRB) in case questions arise at a later time. The consent form will also explicitly state that study participation is voluntary, and that participants may refuse to answer any questions that make them uncomfortable, and may discontinue participation at any time. In addition, participants will be assured that withdrawal from the study will not compromise their medical care in any way.

As informed consent is a continuous process, participants will be given a copy of the signed informed consent document, and will be invited to ask questions about their participation at any point over the course of the study.

NOTE: When subjects are unable to give consent due to age (minors) or impaired decision-making capacity, complete the forms for Research Involving Children as Subjects of Research

and/or Research Involving Individuals with Impaired Decision-making Capacity, available on the New Submissions page on the PHRC website:
https://partnershealthcare.sharepoint.com/sites/phrmApply/aieipa/irb

For guidance, refer to the following Partners policy:
Informed Consent of Research Subjects:
https://partnershealthcare-public.sharepoint.com/ClinicalResearch/Informed_Consent_of_Research_Subjects.pdf

DATA AND SAFETY MONITORING

Describe the plan for monitoring the data to ensure the safety of subjects. The plan should include a brief description of (1) the safety and/or efficacy data that will be reviewed; (2) the planned frequency of review; and (3) who will be responsible for this review and for determining whether the research should be altered or stopped. Include a brief description of any stopping rules for the study, when appropriate. Depending upon the risk, size and complexity of the study, the investigator, an expert group, an independent Data and Safety Monitoring Board (DSMB) or others might be assigned primary responsibility for this monitoring activity.

NOTE: Regardless of data and safety monitoring plans by the sponsor or others, the principal investigator is ultimately responsible for protecting the rights, safety, and welfare of subjects under his/her care.

Risks to participants are minimal. In the unlikely event that a participant is determined to be actively suicidal and at risk for self-harm during any study procedures, the research assistant will contact the PI (Vranceanu) and appropriate clinical intervention will be executed. Dr. Vranceanu may start a psychiatric consult depending on the severity of the situation. All study staff have been trained in responsible research conduct through a CITI course at MGH. The research assistant has also been trained on the importance of maintaining confidentiality, and the assignment of ID numbers. All data will be kept confidential, under lock-and-key, accessible only to trained study staff. Participants' data will be identified by ID number only, and a link between names and ID numbers will be kept separately under lock and key.

Describe the plan to be followed by the Principal Investigator/study staff for review of adverse events experienced by subjects under his/her care, and when applicable, for review of sponsor safety reports and DSMB reports. Describe the plan for reporting adverse events to the sponsor and the Partners' IRB and, when applicable, for submitting sponsor safety reports and DSMB reports to the Partners' IRBs. When the investigator is also the sponsor of the IND/IDE, include the plan for reporting of adverse events to the FDA and, when applicable, to investigators at other sites.

NOTE: In addition to the adverse event reporting requirements of the sponsor, the principal investigator must follow the Partners Human Research Committee guidelines for Adverse Event Reporting

Adverse events may be discovered in the event that a patient spontaneously reports an adverse event, or an adverse event is discovered during the assessment process. All adverse events will be reported by the PI to the Office of Research Compliance within 24 hours.

MONITORING AND QUALITY ASSURANCE

Describe the plan to be followed by the principal investigator/study staff to monitor and assure the validity and integrity of the data and adherence to the IRB-approved protocol. Specify who will be responsible for monitoring, and the planned frequency of monitoring. For example, specify who will review the accuracy and completeness of case report form entries, source documents, and informed consent.<br><br>NOTE: Regardless of monitoring plans by the sponsor or others, the principal investigator is ultimately responsible for ensuring that the study is conducted at his/her investigative site in accordance with the IRB-approved protocol, and applicable regulations and requirements of the IRB.

Once completed, a member of study staff will verify that all items on all questionnaires have been addressed. Data will be checked for out of range values using frequency distributions prior to analyzing the data. The Principal Investigator will be responsible for ensuring compliance with IRB procedures.

For guidance, refer to the following Partners policies:<br>Data and Safety Monitoring Plans and Quality Assurance<br>**https://partnershealthcare-public.sharepoint.com/ClinicalResearch/DSMP_in_Human_Subjects_Research.pdf**<br><br>Reporting Unanticipated Problems (including Adverse Events)<br>**https://partnershealthcare-public.sharepoint.com/ClinicalResearch/Reporting_Unanticipated_Problems_including_Adverse_Events.pdf**

PRIVACY AND CONFIDENTIALITY

Describe methods used to protect the privacy of subjects and maintain confidentiality of data collected. This typically includes such practices as substituting codes for names and/or medical record numbers; removing face sheets or other identifiers from completed surveys/questionnaires; proper disposal of printed computer data; limited access to study data; use of password-protected computer databases; training for research staff on the importance of confidentiality of data, and storing research records in a secure location.<br><br>NOTE: Additional measures, such as obtaining a Certificate of Confidentiality, should be considered and are strongly encouraged when the research involves the collection of sensitive data, such as sexual, criminal or illegal behaviors.

As noted above, study data will not be linked to any identifying information; rather, study ID numbers will be assigned and used to identify participants. All study forms will be stored in

locked storage spaces, to which only study staff will have access. All study staff will complete required Partners human subjects trainings prior to the start of study procedures.

SENDING SPECIMENS/DATA TO RESEARCH COLLABORATORS OUTSIDE PARTNERS

Specimens or data collected by Partners investigators will be sent to research collaborators outside Partners, indicate to whom specimens/data will be sent, what information will be sent, and whether the specimens/data will contain identifiers that could be used by the outside collaborators to link the specimens/data to individual subjects.

Data collected from the current study will not be sent to research collaborators outside of Partners.

Specifically address whether specimens/data will be stored at collaborating sites outside Partners for future use not described in the protocol. Include whether subjects can withdraw their specimens/data, and how they would do so. When appropriate, submit documentation of IRB approval from the recipient institution.

Specimens / data will not be stored at collaborating sites outside of Partners for future use not described in this protocol.

RECEIVING SPECIMENS/DATA FROM RESEARCH COLLABORATORS OUTSIDE PARTNERS

When specimens or data collected by research collaborators outside Partners will be sent to Partners investigators, indicate from where the specimens/data will be obtained and whether the specimens/data will contain identifiers that could be used by Partners investigators to link the specimens/data to individual subjects. When appropriate, submit documentation of IRB approval and a copy of the IRB-approved consent form from the institution where the specimens/data were collected.

Specimens and data will not be collected by research collaborators outside of Partners.

Partners HealthCare System
Research Consent Form

Certificate of Confidentiality Template
Version Date: January 2018

Subject Identification

Protocol Title: Recovering Together: Building resiliency in dyads in patients admitted to the Neuroscience Intensive Care Unit (NICU) and their caregivers

Principal Investigator: Ana-Maria Vranceanu, PhD

Site Principal Investigator: N/A

Description of Subject Population: Acute neurological illnesses (ANIs) patient - caregiver dyads admitted to the NICU

About this consent form

Please read this form carefully. It tells you important information about a research study. A member of our research team will also talk to you about taking part in this research study. People who agree to take part in research studies are called "subjects." This term will be used throughout this consent form.

Partners HealthCare System is made up of Partners hospitals, health care providers, and researchers. In the rest of this consent form, we refer to the Partners system simply as "Partners."

If you have any questions about the research or about this form, please ask us. Taking part in this research study is up to you. If you decide to take part in this research study, you must sign this form to show that you want to take part. We will give you a signed copy of this form to keep.

A description of this clinical trial will be available on *https://www.ClinicalTrials.gov*, as required by the U.S. Law. This website will not include information that can identify you. At most, the website will include a summary of the results. You can search this website at any time.

Subject Identification

Why is this research study being done?

This research study is being done to understand the experiences of patients admitted to the neuroscience intensive care unit (Neuro-ICU) and their primary caregivers. The purpose of this study is to compare two dyadic (patient and caregiver) programs to improve emotional distress in patients and caregivers.

We are asking you to take part in this research study because you are at least 18 years of age, an English-speaking patient with an acute neurological illness (ANI) admitted to the intensive care unit, or a caregiver of a patient with an ANI.

About 80 dyads (160 people) will take part in this research study.

This research study is being conducted by the Neuroscience Intensive Care Unit at Massachusetts General Hospital. The National Institute of Nursing Research is paying for this study to be done.

How long will I take part in this research study?

It will take you about 4-5 months to complete this research study.

What will happen in this research study?

The training program we are testing was developed based on information from the nursing care team and patients and caregivers like yourself. It has 6 sessions and it teaches behavioral, and psychosocial skills. Two sessions will take place during hospitalization and 4 sessions will take place through a secure, live-videoconferencing program called Vidyo.

If you choose to participate in this study, we will ask you to sign this consent form before we do any study procedures.

Once you sign this consent form and agree to participate in this study, you and your partner will be randomly assigned together to one of two groups. You will be assigned by chance (like flipping a coin) to one of the two dyadic training programs. There is no way to predict which of the two groups you and your partner you will be in. Both groups will participate in a dyadic program with a study therapist and a program manual. We do not know right now which intervention will be more effective. You will not know what the other intervention is.

Below is an outline of the study schedule:

Study Intake (1/2 hour)

During this portion of the study, you will fill out several questionnaires online or in person, through a secure system. The survey questions will ask you about your stress levels, mental health, behavior, medical symptoms, and quality of life. You can complete these questionnaires in-person during your intake visit, on paper at home, or on a personal computer at home.

For your safety, we will also ask you to provide contact information for a family member or friend that we may contact on your behalf in case of emergency.

Weekly Sessions 1-6

The intervention will have 6 sessions with 2 general sessions delivered in-person within the Neuro-ICU and 4 tailored specific sessions to be delivered via live video using Vidyo, when you return home or at rehab. Vidyo is a HIPAA approved, secure online videoconferencing software program. We will help you set up for Vidyo sessions. We will help you install Vidyo on your computer, tablet or smartphone and will teach you how to use it before we start the groups. Once you log in from a webcam-equipped computer, the Vidyo program will allow you to see and hear the entire group in real-time, while participating from your home or another independent location. Study staff will schedule one brief Vidyo meeting with you to ensure that you are comfortable with using the software.

Post Program Assessment (1/2 hour)

This portion of the study will occur after you have completed the 6-week program. You will fill out several questionnaires. The survey questions will ask you about your stress levels, mental health, behavior, medical symptoms and quality of life. You will complete these on a personal computer at home.

Follow-Up Assessments (1/2 hour each)

This portion of the study will occur 3 months after you have completed the 6-week program. You will fill out several questionnaires. The survey questions will ask you about your stress levels, mental health, behavior, medical symptoms and quality of life. You will complete these questionnaires on a computer from home.

Place of Visits: You can attend the online group sessions from your home or any other private place with a personal computer. The personal computer must be equipped with a webcam and Vidyo videoconferencing software.

Confidentiality: Your research study information will remain confidential, stored without identifying information, and be accessible only to study staff. Confidentiality will only be suspended in the case of a psychological emergency. In the unlikely event that a participant is determined to be actively suicidal and at risk for self-harm during any study procedures, the research assistant will contact the Principal Investigator (Vranceanu) and appropriate clinical intervention will be executed. Dr. Vranceanu may start a psychiatric consult depending on the severity of the situation.

OPTION TO CHOOSE:
The study investigator may wish to re-contact you in the future about related research studies. Do you agree to let us contact you in the future?
YES: ________ NO: _________

What are the risks and possible discomforts from being in this research study?

There are no foreseeable physical risks from this research study. Responding to questions about your recent feelings, emotions, and thoughts may cause you to feel discomfort. If you experience any of these symptoms, you may choose not to answer any question that makes you feel uncomfortable. You may also find it time consuming to participate in the 6 visits.

What are the possible benefits from being in this research study?

You will not benefit from this study directly. You may enjoy the opportunity to talk about your ANI experience and to share your story. However, knowledge from this research study may benefit others by enhancing our understanding of the role of skills-based interventions in treating future ANI patients or caregivers of ANI patients.

What other treatments or procedures are available for my condition?

The program offered in this research study does not constitute individualized, personal care. These interventions are broad-based training methods that are not tailored to any individual. If you would like formal mental healthcare or personalized instruction in mind body methods, we can give you a referral for psychological treatment that is suitable for you. For example, you may seek psychotherapy or medications outside of this research study or you may participate in other research studies for which you may qualify.

Participation in this research study does not mean that you cannot seek other forms of treatment for psychological distress, including medications or other forms of psychotherapy. In fact, we ask that you continue your regular medical treatment with your physician in addition to taking part in this research study.

Can I still get medical care within Partners if I don't take part in this research study, or if I stop taking part?

Yes. Your decision won't change the medical care you get within Partners now or in the future. There will be no penalty, and you won't lose any benefits you receive now or have a right to receive.

Taking part in this research study is up to you. You can decide not to take part. If you decide to take part now, you can change your mind and drop out later. We will tell you if we learn new information that could make you change your mind about taking part in this research study.

What should I do if I want to stop taking part in the study?

If you take part in this research study, and want to drop out, you should tell us. We will make sure that you stop the study safely. We will also talk to you about follow-up care, if needed.

Also, it is possible that we will have to ask you to drop out of the study before you finish it. If this happens, we will tell you why. We will also help arrange other care for you, if needed.

Will I be paid to take part in this research study?

All participants will receive $20 for the completion of each of the 3 assessment points. Participants can receive up to $60 for study participation.

What will I have to pay for if I take part in this research study?

There will be no cost to you for any study visits. All of the group sessions and study assessments will be paid for by study funds. However, you will be required to have access to a personal computer equipped with a webcam or other video/audio capture device. The study will not provide you with such a computer. In addition, you will be required to download and install Vidyo videoconferencing software in order to participate in the online group sessions. This software is available for a free download, and study staff will give you specific instruction on how to locate and install it onto your computer.

Subject Identification

What happens if I am injured as a result of taking part in this research study?

We will offer you the care needed to treat any injury that directly results from taking part in this research study. We reserve the right to bill your insurance company or other third parties, if appropriate, for the care you get for the injury. We will try to have these costs paid for, but you may be responsible for some of them. For example, if the care is billed to your insurer, you will be responsible for payment of any deductibles and co-payments required by your insurer.

Injuries sometimes happen in research even when no one is at fault. There are no plans to pay you or give you other compensation for an injury, should one occur. However, you are not giving up any of your legal rights by signing this form.

If you think you have been injured or have experienced a medical problem as a result of taking part in this research study, tell the person in charge of this study as soon as possible. The researcher's name and phone number are listed in the next section of this consent form.

If I have questions or concerns about this research study, whom can I call?

You can call us with your questions or concerns. Our telephone numbers are listed below. Ask questions as often as you want.

Ana-Maria Vranceanu, PhD, is the person in charge of this research study. You can call her at 617-724-4977, Monday through Friday, 9:00 am to 5:00 pm. You can also call Dr. Jonathan Rosand, MD, at 617-724-2698, Melissa Gates, at 617-643-9406, or Ann Lin, at 617-724-0051, Monday through Friday, 9:00 am to 5:00 pm, with questions about this research study.

If you have questions about the scheduling of appointments or study visits, please call Melissa Gates, at 617-643-9406, or Ann Lin, at 617-724-0051.

If you want to speak with someone **not** directly involved in this research study, please contact the Partners Human Research Committee office. You can call them at 857-282-1900.

You can talk to them about:

- Your rights as a research subject
- Your concerns about the research

- A complaint about the research

Also, if you feel pressured to take part in this research study, or to continue with it, they want to know and can help.

If I take part in this research study, how will you protect my privacy?

Federal law requires Partners to protect the privacy of health information and related information that identifies you. We refer to this information as "identifiable information."

In this study, we may collect identifiable information about you from:

- Past, present, and future medical records
- Research procedures, including research office visits, tests, interviews, and questionnaires

Who may see, use, and share your identifiable information and why:

- Partners researchers and staff involved in this study
- The sponsor(s) of the study, and people or groups it hires to help perform this research or to audit the research
- Other researchers and medical centers that are part of this study
- The Partners ethics board or an ethics board outside Partners that oversees the research
- A group that oversees the data (study information) and safety of this study
- Non-research staff within Partners who need identifiable information to do their jobs, such as for treatment, payment (billing), or hospital operations (such as assessing the quality of care or research)
- People or groups that we hire to do certain work for us, such as data storage companies, accreditors, insurers, and lawyers
- Federal agencies (such as the U.S. Department of Health and Human Services (DHHS) and agencies within DHHS like the Food and Drug Administration, the National Institutes of Health, and the Office for Human Research Protections) state agencies, and foreign government bodies that oversee, evaluate, and audit research, which may include inspection of your records
- Public health and safety authorities, if we learn information that could mean harm to you or others (such as to make required reports about communicable diseases or about child or elder abuse)

- Other researchers within or outside Partners, for use in other research as allowed by law.

Certificate of Confidentiality

A federal Certificate of Confidentiality (Certificate) has been issued for this research to add special protection for information and specimens that may identify you. With a Certificate, unless you give permission (such as in this form) and except as described above, the researchers are not allowed to share your identifiable information or identifiable specimens, including for a court order or subpoena.

Certain information from the research will be put into your medical record and will not be covered by the Certificate. This includes records of medical tests or procedures done at the hospitals and clinics, and information that treating health care providers may need to care for you. Please ask your study doctor if you have any questions about what information will be included in your medical record. Other researchers receiving your identifiable information or specimens are expected to comply with the privacy protections of the Certificate. The Certificate does not stop you from voluntarily releasing information about yourself or your participation in this study.

Even with these measures to protect your privacy, once your identifiable information is shared outside Partners, we cannot control all the ways that others use or share it and cannot promise that it will remain completely private.

Because research is an ongoing process, we cannot give you an exact date when we will either destroy or stop using or sharing your identifiable information. Your permission to use and share your information does not expire.

The results of this research may be published in a medical book or journal, or used to teach others. However, your name or other identifiable information **will not** be used for these purposes without your specific permission.

Your Privacy Rights

You have the right **not** to sign this form that allows us to use and share your identifiable information for research; however, if you don't sign it, you can't take part in this research study.

You have the right to withdraw your permission for us to use or share your identifiable information for this research study. If you want to withdraw your permission, you must notify the person in charge of this research study in writing. Once permission is withdrawn, you cannot continue to take part in the study.

If you withdraw your permission, we will not be able to take back information that has already been used or shared with others, and such information may continue to be used for certain purposes, such as to comply with law or maintain the reliability of the study.

You have the right to see and get a copy of your identifiable information that is used or shared for treatment or for payment. To ask for this information, please contact the person in charge of this research study. You may only get such information after the research is finished.

Informed Consent and Authorization

Statement of Person Giving Informed Consent and Authorization

- I have read this consent form.
- This research study has been explained to me, including risks and possible benefits (if any), other possible treatments or procedures, and other important things about the study.
- I have had the opportunity to ask questions.
- I understand the information given to me.

Signature of Subject:

I give my consent to take part in this research study and agree to allow my identifiable information to be used and shared as described above.

______________________________	____________	____________
Subject	Date	Time (optional)

Signature of Study Doctor or Person Obtaining Consent:

Statement of Study Doctor or Person Obtaining Consent

- I have explained the research to the study subject.
- I have answered all questions about this research study to the best of my ability.

_______________________________ _______________ _______________
Study Doctor or Person Obtaining Consent | Date | Time (optional)

Consent Form Version Date: 10/11/2018

PARTNERS HUMAN RESEARCH COMMITTEE DETAILED PROTOCOL

Principal Investigator: Ana-Maria Vranceanu, PhD

Protocol Title: Recovering Together: Building resiliency in dyads in patients admitted to the Neuroscience Intensive Care Unit (NICU) and their caregivers

Funding: National Institute of Nursing Research

Version Date: 10/11/18

I. BACKGROUND AND SIGNIFICANCE

Acute neurological illnesses (ANIs) are common, costly and often lead to long-term disability. ANIs are biologically distinct injuries that disrupt the normal function of the brain. The most common ANIs in Neuroscience Intensive Care Units (NICU) include cerebrovascular (stroke/hemorrhage and brain aneurysm), structural (tumors and lesions/brain masses), and traumatic (TBI) brain injuries. NICU admissions for ANIs are prevalent (e.g., 795,000 acute stroke/year; 275,000 acute TBI/year) and costly[1,2]; post NICU prolonged rehabilitation is common[3].

ANIs are associated with chronic emotional distress in both patients (pts) and caregivers (cgs). Although biologically heterogeneous, ANIs are unified by sudden onset, and substantial emotional distress in both pts (e.g., 12-43% anxiety[4-7]; 10-58% depression[4,7,8]; 20-29% post-traumatic stress PTS[5,9]) and family cgs (27-60% depression, anxiety or PTS [4,10,11]). These symptoms often become chronic and treatment resistant[26,27].

Pt and cg factors interact and influence physical and emotional outcomes in both pts and cgs. Post ANI emotional distress is associated with pts' poor medical adherence[28], slower recovery[28-30], higher mortality[29-31], and need of more caregiving assistance[32], which further increase cgs' distress[30,33,34] and own risk for morbidity[35,36] and mortality[37]; in turn cgs' emotional distress interferes with ability to provide high-quality care to pts[38,39] and negatively impacts pts' outcomes.

Current management of ANIs does not meet the psychological needs of pts and cgs for 3 reasons[40-51]. First, although recognition of the emotional burden associated with NICU admission has increased, and some NICUs have social workers available to assist pts and cgs, there are no formal screening methods for emotional distress routinely integrated in practice during hospitalization, when the primary focus is on medical care and survival; further, there are no formal evidence-based treatments integrated within the medical care. When social workers are included to help pts and cgs, the care is brief and occurs only during hospitalization. When referrals to mental health services are provided to families at discharge, few will access additional treatment due to burden associated with traveling outside of home. Second, psychosocial interventions available for ANI pts or cgs are limited in that they are delivered when symptoms are already chronic, address only one emotional illness (e.g., depression or anxiety or PTS), and/or are focused on a *single member* of the pt-cg dyad. Even interventions labeled as "dyadic," which include pts and cgs, typically address only the pts' needs and do not

focus on cg outcomes or on the dyad's interpersonal communication and bond/relationship[12,52]. These interventions are not consistent with the *dyadic framework*[22] which specifies that dyadic interventions should account for the interdependence between pt and cg psychosocial factors including their interpersonal bond by ensuring that both pts and cgs attend each session together, and by targeting improvement in outcomes for both pts and cgs. Third, most interventions are delivered using uniform protocols. However, the needs of ANI dyads are heterogeneous due to varying levels of post ANI impairment, identity of the cg, context and stage of life. A recent systematic review[52] urged for the development of dyadic interventions that address the needs of *both* pts and cgs and are tailored to the specific needs of each ANI dyad.

We developed the first dyadic skills-based intervention – Recovering Together - to prevent chronic heightened emotional distress in at risk ANI pt-cg dyads. The "Recovering Together" program is informed by the theoretical response-shift framework of adaptation to acute illness[53] (successful adaptation implies recalibration of values and life goals), the family strength vulnerability model[24] (within dyads relational systems have strengths and weaknesses in how they cope with life events), the dyadic longitudinal model[22] (distress travel from one member of the dyad to the other across time), the APIM model[54], and the resiliency framework[17]**.** The program is in line with recent recommendations for skill-based interventions for critical care patients, and uses preliminary data collected by our team for the past 3 years[4, 13-15]. The intervention teaches pts and cgs resiliency factors that are associated with well-being after trauma for both pts and cgs: *mindfulness* – the ability to stay present and defer judgment in the face of adversity[18]; *coping*– the arsenal and application of one's behavioral, cognitive, and emotional strategies to manage stress[19]; *social support* –empathetic interpersonal interactions that meet one's emotional and functional needs[20]; *self-efficacy* – perceived ability to adapt under adversity[21] and *positive dyadic interpersonal communication to increase interpersonal bond*[12]. Informed by the aforementioned theoretical models, our conceptual model hypothesizes that by teaching both members of the dyad resiliency and interpersonal communication skills (e.g., Recovering Together) we will be able to sustainably decrease emotional distress in both members of the dyad

In addition, our team has an established record of collaboration on published or ongoing investigations. **Emotional distress is prevalent in dyads, interdependent between pt and cg, and negatively associated with resiliency factors[4].** Our team conducted a cross-sectional study of pt-cg dyads in the NICU (40% stroke, 30% tumor). 75% pts and 84% cgs approached agreed to participate. 74% pts had been intubated at one time during NICU admission, and 2/3 were discharged home. Rates of clinically significant symptoms of depression, anxiety and PTS did not differ between pts (24%, 43%, 21%) and cgs (24%, 46%, 17%), or by any demographic or medical characteristic. Dyadic modeling showed that for both pts and cgs, mindfulness and coping impacted both self and partner's emotional distress symptoms. We showed: 1) feasibility of recruitment; 2) high emotional distress in dyads; and that 3) modifiable resiliency factors (mindfulness and coping) are intervention targets interdependently associated with distress in pts and cgs, regardless of the identity of cg (e.g., spouse, friend, etc).

Resiliency factors are associated with lower emotional distress in NICU dyads[13]. Our team found that resiliency factors of mindfulness, coping, self-efficacy and patient-caregiver interactions were associated with decreased emotional distress in dyads of ANI. This study confirms mindfulness, coping as intervention targets and provides novel evidence on self-efficacy and patient-caregiver interaction as additional important intervention targets.

ANI pts have greater anxiety than cancer patients at early diagnoses[14]. We led the first cross-comparison study of emotional distress among dyads with ANI and cancer. This study supports the priority of addressing emotional distress in ANI dyads as has been emphasized for cancer dyads.

Clinically significant emotional distress in one member of the dyad at hospitalization predicts chronic emotional distress in at least 1 of the dyad members 3 and 6 months later[55]. Our team has an ongoing prospective study of dyads with ANI. Retention rates for dyads due for assessments at 3 and 6 months thus far are 84% and 91% for pts and 87.7% and 95.7% for cgs, confirming our ability to retain post-ANI participants. Within each dyad, if one member screens in for clinically significant symptoms for any diagnosis (i.e., depression, anxiety or PTSD) at hospitalization there is good sensitivity and specificity that one member of the dyad will endorse clinically significant symptoms 3 months later. This study shows a reliable method for identifying dyads of patients at risk for chronic heightened emotional distress by identifying dyads in which either the pt or cg screens in for heightened emotional distress (symptoms of depression, anxiety or PTSD).

Caregiver gender moderates the prospective association of resiliency factors to emotional distress[87]. This study found that at the time of admission resiliency factors have main effects on emotional distress, with no differences by cg gender. However, significant interaction effects emerged prospectively such that male cgs with high mindfulness at baseline demonstrated lower levels of emotional distress at 3 and 6 months later than did males with low mindfulness ($p = 0.026$ and $p <0.013$). Similarly, women cgs with high intimate bond at baseline reported the lowest levels of depression symptoms 3 and 6 months later ($p < 0.020$). This study confirmed the need to assess and address resiliency factors early in the recovery process, and identified important gender differences to be accounted for in intervention development.

Recovering Together; Developing a novel dyadic resiliency skills program for ANI pt-cg dyads at risk for chronic emotional distress[15]. With funding from American Heart Association, we conducted 20 qualitative interviews with pt-cg stroke dyads at risk for chronic emotional distress during NICU hospitalization. We also conducted additional clinical interviews with 10 pt-cg dyads representative of other ANI diagnoses. 83% dyads approached agreed to participate. Pts (23) and cgs (25) were mostly women. Dyads were mostly spouses and mothers-daughter. Data was analyzed with Nvivo10. Main themes did not differ by medical diagnoses: 1) most challenging and distressing experiences: uncertainty about future, anxiety, depression, sleep difficulties, worries about the future, guilt, managing job with caretaking, making treatment decisions, lack of predictability; 2) concerns about interpersonal relationships (self-image, role changes, role fulfillment); 3) fear of recurrence; 4) adjusting to sequelae. Dyads noted interest in a resiliency program (30/30) and preferred a combination of in person and live video sessions (30/30). Dyads learned about resiliency skills (e.g., name, description and goal) and agreed they would be helpful to them. They also noted interest in learning about: survivorship plans, adaptation to deficits (present or anticipated), and return to normal living. We found no thematic differences between stroke dyads and other ANI dyads. Challenges associated with embracing the caregiver role emerged as a theme while differences by the identity of caregiver (e.g., spouse vs. friend, vs parent) did not. Themes associated with the gender of the cg emerged and have been incorporated in the intervention.

Nurses perception of the needs for and feasibility of "Recovering Together for ANI families[25]. We conducted 2 focus groups (N =15) with NICU nurses who provided feedback on the qualitative findings from our 30 ANI dyads and shared own experiences and opinions on

implementation and scalability of the intervention including nurse involvement. Nurses concurred with pts experiences, and suggested strategies to recruit and retain dyads for the study, which are now included in the methodology section of the current grant proposal. Studies 3.6 and 3.7 represent the building blocks for the development of our Recovering Together program and manual. Nurses provided edits and contributed to the iterative development of the manual. These studies also confirm feasibility of conducting the pilot RCT proposed through this R21.

II. SPECIFIC AIMS

The current study has the following objectives:

Aim 1: **To determine the feasibility of recruitment, feasibility of program delivery, program credibility, and program satisfaction using evidence-based benchmarks.**

Hypothesis 1: We hypothesize that > 75% of the dyads approached will agree to participate.
Hypothesis 2: We hypothesize that > 75% of dyads who start the intervention will complete at least 4 sessions.
Hypothesis 3: We hypothesize that > 75% of participants will report average credibility (Credibility and Expectancy Questionnaire) scores greater than the scale's midpoint.
Hypothesis 4: We hypothesize that > 75% participants will report average satisfaction (Client Satisfaction Scale) scores greater than the scale's midpoint.

Aim 2: **To demonstrate a proof of concept that the Recovering Together program can sustainably improve emotional distress [Hospital Anxiety and Depression Scale; HADS], Post Traumatic Symptoms (PTS) [PCL-S], resiliency variables (mindfulness, coping, social support and self-efficacy) and interpersonal factors (interpersonal bond).**

Hypothesis 1: We hypothesize that participation in the Recovering Together Program will be associated with a more potent decrease in emotional distress and PTS compared to participation in the educational program (control), and that these improvements will maintain at 3 month follow up.
Hypothesis 2: We hypothesize that Recovering Together Program will be associated with a more potent increase in resiliency variables (mindfulness, coping, social support, self-efficacy) and interpersonal factors (interpersonal bond) compared to participation in the educational program, and that these improvements will maintain at 3 month follow up.

For this feasibility pilot RCT, our primary outcomes (feasibility, credibility and satisfaction) will be assessed in Aim 1. Our secondary outcomes in this trial are: emotional distress, PTS, mindfulness, coping, social support, self-efficacy, and interpersonal bond, and will be assessed in Aim 2.

III. SUBJECT SELECTION

All participants will be recruited from the Massachusetts General Hospital Neuroscience ICU, using IRB approved recruitment materials.

Inclusion/Exclusion Criteria

Eligible dyads (Pts and Cgs) must meet the following inclusion criteria:

1) Age 18 or older
2) English fluency and literacy
3) Access to high speed internet for video sessions
4) Pt with an informal cg (family or friend who provides unpaid care) available and willing to participate
5) Hospitalized with an ANI within 1-2 weeks (pt) OR primary cg of a pt currently admitted with an ANI
6) Either pt or cg have clinically significant symptoms of depression, anxiety, and/or PTS

One or more of the following exclusion criteria will render a pt ineligible:

1) Permanent and severe cognitive impairment severe enough to impede participation – This will be determined by nurses through an assessment conducted as part of usual care and that includes the MMSE (score of <23) and GCS (score of <10). Nurses are trained and use these measures as part of NICU care.
2) Dyads where the pt is anticipated to die or to never be able to participate due to medical sequelae. This will be determined by nurses.

All adult patients and family caregivers, satisfying all inclusion criteria, are eligible for enrollment in this study regardless of sex, race, or ethnicity. Vulnerable populations will not be recruited.

Recruitment

Patients will be recruited from the NICU at MGH from the medical team (nurses). Recruitment will be facilitated by the nursing team who will introduce the study to eligible dyads and who will also assess whether pts are able to consent, consistent with medical presentation, and cognitive status. Nurses will be administering two cognitive measures, MMSE and GSC, to all patients and will refer only dyads where the patients scores are higher than the established cut off scores on these measures. Consistent with NICU practice, they will administer these measures. They will refer participants to the study only if they are cleared medically and cognitively. The team will page the RA using the secure Voalte system used by our team and nursing staff, when both the pt and cg are present and able to hear more about the study. The RA will only approach pts identified and cleared by the medical team. The RA will ensure eligibility of both pts and cgs based on the additional inclusionary and exclusionary criteria depicted above. The RA will finalize the screening and conduct informed consent**.** All participants will receive a physical copy of the informed consent form which has the contact information for the PI. If cgs cannot be reached in person in the hospital, they will be contacted via phone for screening and completion of informed consent. Cgs will be given a copy of the consent form to review while discussing the study over the telephone. We will fax or securely e-mail the consent form prior to obtaining consent. Cg will return a signed copy of the consent form.

Patients and caregivers will be considered enrolled when they sign the consent form with the study staff. Please see details on the informed consent process in the next section, Subject Enrollment (Section IV). These procedures will be completed in a private medical space.

Participants will be explicitly informed that this intervention is a research study that does not constitute individualized, personal care. The intervention is a broad-based method of training that is not tailored to any particular individual. Should any participant seek formal mental healthcare, study staff will refer them to either MGH Psychiatry, as appropriate.

We will recruit pts with any type of ANI and their cgs, ensuring representation of all NICU diagnoses. All cgs will be recruited within the first week of the pts' hospitalization. Pt's medical team will also alert the research assistant whether they anticipate that the patient might be able to participate in the study at a future point during the hospital stay. In situations where patients are unable to consent due to the severity of the ANI, we will enroll the informal cg and return to enroll the pt as soon as their mental capacity improves. The research assistant will not approach patients who are not mentally or physically capable to participate. This study does not include participants with impaired decision making.

We will not include patients who do not have a caregiver. Informal caregivers will be designated by the health care proxy and verbally confirmed by the patient. Nurses will assist the study team with identifying the patient's health care proxy.

Eligible cases may also be identified by daily screening of Epic admission reports.

The study team will keep track of all pts and cgs approached who refuse to participate (along with reasons for refusal), as well as those who were not approached and reasons why.

The recruitment process was developed and refined through prior research for the past 2 years.

IV. SUBJECT ENROLLMENT

Participants will be referred to the study by the nursing team who will ensure that patients are medically and cognitively able to participate. Eligible dyads will next be screened, consented, and enrolled by the research assistant.

After determining eligibility, study staff will meet with potential dyads to review the informed consent document. If cgs cannot be reached in person in the hospital, they will be contacted via phone to complete informed consent. Cgs will be given a copy of the consent form to review while discussing the study over the telephone. We will fax or securely e-mail the consent form prior to obtaining consent. Cgs will return a signed copy of the consent form. After the document has been reviewed, study staff will answer any and all questions the pt or cg may have. Once all questions have been addressed, each member of the dyad will sign the consent form, which will include a description of all study procedures, information about potential risks and benefits of participation, and study contact information (including that of the IRB) in case questions arise at a later time. The consent form will also explicitly state that study participation is voluntary, and

that participants may refuse to answer any questions that make them uncomfortable, and may discontinue participation at any time. In addition, participants will be assured that withdrawal from the study will not compromise their medical care in any way.

As informed consent is a continuous process, participants will be given a copy of the signed informed consent document, and will be invited to ask questions about their participation at any point over the course of the study.

Following a study enrollment and baseline assessment, dyads will be randomly assigned to either the newly developed psychosocial intervention or to the educational program (control) using a random number sequence generator to ensure comparability between groups. We will document time between assessment and intervention initiation, and analyze as a predictor of study outcomes as needed. Randomization will be developed by the statistician, without any input from the rest of the team.

V. STUDY PROCEDURES

After enrollment, participants will complete study assessments. All subjects will be given baseline psychological and behavioral assessments that will assess depression, anxiety, PTS symptoms, and other psychological constructs. Assessments will be administered online, using the REDCap system. Subjects may choose to fill out these questionnaires on-site, or to fill them out at home on a personal computer or other Internet-equipped device. This assessment includes demographic information and a battery of psychological questionnaires. We will also collect information about important clinical variables including duration of acute hospitalization. All questionnaires are itemized below:

Administered at Baseline Only:
Demographics
Prior Mental Health History questions
Credibility Questionnaire

Administered at Baseline, Post-Intervention, and Follow-up:
Medical history information from LMR (i.e., prior ANI status, current psychotropic meds, comorbid medical conditions, etc.)
Post-Traumatic Stress Disorder Checklist (PCL-S)[29]
Hospital Depression and Anxiety Scale (HADS)[30]
Measure of Current Status Part A (MOCS-A)[31]
The Cognitive and Affective Mindfulness Scale (CAMS)[32]
World Health Organization Quality of Life (WHOQOL-BREF)[33]

Administered Post-Intervention and Follow-up Only:
Client Satisfaction Questionnaire (CSQ-8)[34] [intervention group only]

The medical team will complete the following questionnaires to assess stroke severity for each patient:
Modified Rankin Scale (mRS)[35] [Pts only]

Barthel Index[36] [Pts only]

After completing the baseline assessments, dyads will be randomized to either the newly developed psychosocial intervention or to treatment as usual.

The psychosocial intervention entails 6 sessions, each 30 minutes. Both the pt and cg participate in each session. The intervention is manualized, and teaches dyads resiliency (mindfulness, social support, self-efficacy, coping skills) and interpersonal communications (interpersonal bond) skills. The first 2 sessions will occur in person, during hospitalization. The next 4 sessions are chosen from 5 available, depending on each dyad's preference, and are delivered after discharge via secure live video. For the video sessions, dyads can participate from the same or different locations. During the in hospital sessions, dyads learn diaphragmatic breathing, mindfulness, self-care, and dialectics. During the video sessions, dyads learn how to identify negative thinking patterns and replace them with adaptive thoughts, how to communicate effectively and openly, how to engage in self-care, and how to accept things that can't be changed. Participants in the intervention group will also receive treatment as usual. This may include meeting with nurses, physical therapists, medical doctors, and other members of the pt's medical team. Treatment as usual may also involve administration of SSRI to those patients with motor problems.

Those in the educational program (control) will receive general health information that mimics the Recovering Together Program, but without teaching any of the resiliency or interpersonal communication skills that are hypothesized to be responsible for improvement in emotional distress. The educational program will also have 6 sessions, 2 in-person dyadic visits in the NICU and 4 dyadic virtual visits following discharge. It controls for dose and support from clinician. Both members of the dyads participate in all sessions. The topics of each session include: education about the stress of the ANI on patient and caregiver; education on the importance of self-care; education on stress associated with discharge and home adjustment; education on the importance of following up with medical recommendations; education on interpersonal stress as part of adjustment to ANI; education on self-care. The educational program condition will ensure that patients will remain blind to intervention or control and increase confidence that improvement in outcomes are due to the active ingredients of the intervention and not confounds. Participants in the educational program will continue with their current care. This may include meeting with nurses, physical therapists, medical doctors, and other members of the pt's medical team. Treatment as usual may also involve administration of SSRI to those patients with motor problems.

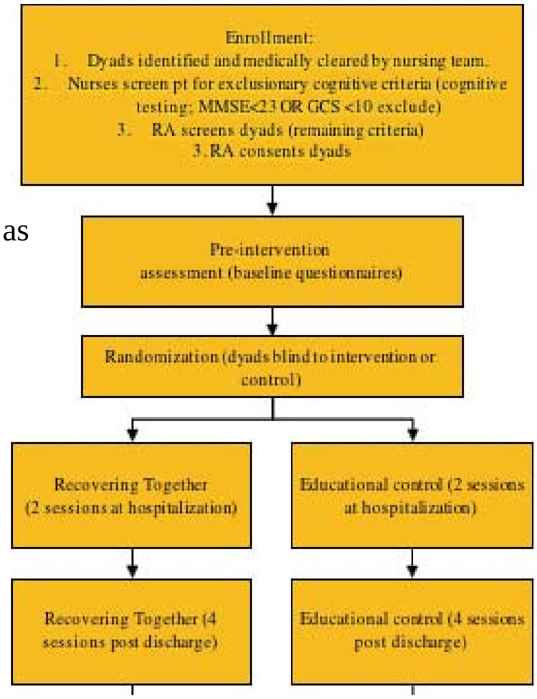

Participants in both groups will be given post-treatment psychological and behavioral assessments identical to those administered at baseline, in addition to the CSQ-8. As a baseline, participants will be given the option to complete post-treatment questionnaires on site or at home. Participants will be asked to complete questionnaires immediately after

completion of the group intervention (T2), and 3 months (T3) after completion of the group intervention, in order to measure long term outcomes. Study staff will email these questionnaires to the participants via the REDCap system.

VI. BIOSTATISTIC ANALYSIS

We plan to conduct a RCT with patients from the Neuro-ICU. The main intervention goal is to provide dyads with resiliency and interpersonal communication skills necessary to optimize recovery. Dyads will be medically cleared by a member of the nursing staff. Nurses will then screen dyads for exclusionary cognitive criteria. Dyads will complete additional screening and baseline questionnaires with a trained research assistant. Dyads will be randomized to one of two groups 1) Psychosocial skills based intervention (Recovering Together), or 2) Educational Control.

We chose our study measures based on our theoretical frameworks, strong psychometric properties in studies of patients with ANI, and feasibility in our prior work with ANI dyads. Measures will be collected at baseline, post-intervention, and at 3 month follow up. Data collection and management will be conducted with Research Electronic Data Capture. Dyads will be given the option to complete measures electronically, by paper and pencil, or telephone.

1) Is the psychosocial skills-based intervention feasible, credible usable, and accepted by pts and cgs in the ICU?

Our primary aim focuses on trial feasibility, acceptability, credibility and preliminary effect. In pilot studies, ≥30 participants are recommended per group to establish feasibility and detect larger effect sizes for ≥1 outcome. We plan to recruit 80 dyads (160 participants), 40 dyads (80 participants) per arm to establish feasibility, acceptability, credibility and estimate effect size for emotional distress variables (primary quantitative outcomes). Assuming attrition of over 25% (in excess of what we experienced in our preliminary studies), we will have the necessary 30 dyads (60 participants) per arm. This size is considered to yield stable estimates of M/SDs based on prior behavioral trial recommendations. Effect sizes from this study may overestimate power in future sample calculations. We chose study measures based on our theoretical frameworks, strong psychometric properties in studies of patients with ANI, and feasibility work in our prior work with ANI dyads. Feasibility will be reported as the percentage of patients enrolled in the study who complete at least 75% of the intervention sessions. Demonstration of feasibility will be assessed by the number of individuals who drop out of the study prior to completing the post- intervention assessment and the rate of missed sessions. If drop-out rate or missed-session rate exceeds 25%, revisions to the intervention may be needed. We will also report number of patients approached, enrolled, randomized and who completed time 2 and time 3 to determine feasibility.

2) Is the psychosocial skills-based intervention effective for pts and cgs in the ICU?

Dr. Vranceanu developed the proposed study design in collaboration with the NICU study team (nurses, physicians, clinical interns) and an MGH psychologist who specializes in using mindfulness and emotion regulation in both chronic illness and medically healthy populations. With funding from the American Heart Association, we conducted 20 qualitative interviews with pt-cg stroke dyads at risk for chronic emotional distress during NICU hospitalization. We also conducted additional clinical interviews with 10 pt-cg dyads representative of other ANI diagnoses. 83% of dyads approached agreed to participate. Pts (23) and cgs (25) were mostly women. Dyads were mostly spouses and mothers-daughter. Dyads noted interest in a resiliency program (30/30) and preferred a combination of in person and live video sessions (30/30). Dyads learned about resiliency skills (name, description, and goal) and agreed they would be helpful to them. They also noted interest in learning about: survivorship plans, adaptation to deficits (present or anticipated), and return to normal living. This led to the development and subsequent refinement (through feedback from the nursing team) of the Recovering Together Program active intervention.

3) Is the effect of the skills-based intervention for pts and cgs in the ICU durable?

We will assess feasibility, usability, and acceptability by the enrollment numbers, participants completion in at least 4 out of 6 sessions, and the questionnaires, The Credibility and Expectancy and Client Satisfaction Scale. These will be primary outcomes. The PCL-S, HADS, and resiliency measures will serve as the secondary outcomes.

We will use student's t-test to assess within-group differences in long-term outcomes. We will compare measures at the 3 month follow-up assessment to measures at the post-intervention assessment for both study arms.

We will also use t tests and chi squared tests to assess differences at the 3 month follow up.

VII. RISKS AND DISCOMFORTS

There is a risk that some participants may feel uncomfortable completing various psychological questionnaires or parts of the skills-based on intervention. Participants are free to withdraw from the study at any time, as the study is completely voluntary.

As in any research study, there is a small risk that confidentiality may be breached; all efforts to minimize this risk will be taken. In the unlikely event that participants will become suicidal during the duration of the study, the research assistant will contact the PI and the appropriate clinical intervention will be executed.

VIII. POTENTIAL BENEFITS

Participants in this study may observe a reduction in depression, anxiety, and/or psychological and physiological markers of stress, as well as an improvement in perceived qualify of life. It is hoped that the intervention will result in improvements across these domains. Participants may learn new techniques for managing distress and lifestyle factors that may enhance wellbeing, both in disease-specific domains as well as in their general lives. In addition, all participants will receive $20 for the completion of each of the 3 assessment points.

IX. MONITORING AND QUALITY ASSURANCE

Electronic information will be stored in REDCap (Research Electronic Data Capture), a free, secure, and HIPAA-compliant web-based application hosted by the Partners HealthCare Research Computing Enterprise Research Infrastructure & Services (ERIS) group (based at the PHS Needham corporate datacenter). Data will be stored on password protected computers that will be stored in secure locations at all times. Paper data files (with coded subject identification) will be stored in a locked filing cabinet. Only research staff will have access to these data locations.

A unique anonymous identifier will be assigned to each subject; subsequently, all data collected will be associated exclusively with this identifier. This includes all questionnaires administered over the course of the study, as well as home practice logs.

Data from this study will be stored for three years after the publication of all study results, at which time all paper data files will be shredded, and computer files will be deleted.

Data Management and Quality Control Procedures

To maximize accuracy and security, all survey data will be collected and stored on REDCap. Research staff will ensure that proper consent has been obtained before sending the REDCap survey to each participant.

REDCap (Research Electronic Data Capture) is a free, secure, HIPAA compliant web-based application hosted by the Partners HealthCare Research Computing Enterprise Research Infrastructure & Services (ERIS) group. Vanderbilt University, with collaboration from a consortium of academic and non-profit institutional partners, has developed this software toolset and workflow methodology for electronic collection and management of research and clinical study data. Data collection projects rely on a study-specific data dictionary defined by members of the research team with planning assistance from Harvard Catalyst, The Harvard Clinical and Translational Science Center EDC Support Staff. This iterative development and testing process results in a well-planned data collection strategy for individual studies. Using REDCap, the research team can also design web-based surveys and engage potential respondents using a variety of notification methods. REDCap provides flexible features that can be used for a variety of research projects and provides an intuitive interface to enter data with real time validation (automated data type and range checks). The system offers easy data manipulation with audit trails, reports for monitoring and querying participant records, and an automated export mechanism to common statistical packages (SPSS, SAS, Stata, R/S-Plus).

Since consistency of application of the study protocol is critical to acquiring high quality data, all research personnel have undergone or will undergo a competency-based training program prior to enrolling subjects.

Data and Safety Monitoring Plan

Adverse Event Monitoring: Throughout the study subjects will be monitored for the occurrence of events defined as any undesirable experience or unanticipated risk. Lack of effect of treatment is not considered an event. All adverse events will be reported on an adverse event form. The Principle Investigator has the responsibility of reporting serious adverse events (death, life threatening illness or injury, serious injury, or permanent disability) to PHRC within 24-72 hours of notification.

X. REFERENCES

1. Center for Disease Control and Prevention. (2015). Stroke Facts: Stroke in the United States. *CDC*. Retrieved from http://www.cdc.gov/stroke/facts.htm.
2. Center for Disease Control and Prevention. (2015). *Report to Congress on Traumatic Brain Injury in the United States: Epidemiology and Rehabilitation.* National Center for Injury Prevention and Control; Division of Unintentional Injury Prevention. Atlanta, GA.
3. The Society of Critical Care Medicine. (2015). What is critical care? *SCCM*. Retrieved from www.myicucare.org.
4. Shaffer KM, Riklin E, Stagl JM, Rosand J, Vranceanu AM. (2016). Mindfulness and coping are inversely related to psychiatric symptoms in patients and informal caregivers in the Neuroscience ICU: Implications for Clinical Care. Critical *Care Medicine*, 44 2036-2038. PMID: 27513536
5. Jackson, J.C., Mitchell, N., & Hopkins, R.O. (2011). Cognitive functioning, mental health, and quality of life in ICU survivors: an overview. *Anesthesiology Clinics, 29,* 751-764. PMID: 22078921
6. Kress, J.P., Gehlbach, B., Lacy, M., Pliskin, N., Pohlman, A.S., & Hall, J.B. (2003). The long-term psychological effects of daily sedative interruption on critically ill patients. *American Journal of Respiratory and Critical Care Medicine, 168,* 1457-1461. PMID: 14525802
7. Cheung, A.M., Tansey, C.M., Tomlinson, G., Diaz-Granados, N., Matté, A., Barr, A., ... Herridge, M.S. (2006). Two-year outcomes, health care use, and costs of survivors of acute respiratory distress syndrome. *American Journal of Respiratory and Critical Care Medicine, 174,* 538-544. PMID: 16763220
8. Daydow, D.S., Gifford, J.M., Desai, S.V., Bienvenu, O.J., & Needham, D.M. (2009). Depression in general intensive care unit survivors: a systematic review. *Intensive Care Medicine, 35,* 796-809. PMID: 19165464
9. Jackson, J.C., Hart, R.P., Gordon, S.M., Hopkins, R.O., Girard, T.D., & Ely, W.E. (2007). Post-traumatic stress disorder and post-traumatic stress symptoms following critical illness in medical intensive care unit patients: assessing the magnitude of the problem. *Critical Care, 11,* R27. PMCID: PMC2151890
10. Choi, J., Hoffman, J.A., Schulz, R., Ren, D., Donahoe, M.P., Given, B., & Sherwood, P.R. (2013). Health risk behaviors in family caregivers during patients' stay in intensive care units: a pilot analysis. *American Journal of Critical Care, 22,* 41-45. PMCID: PMC4109809

11. McAdam, J.L., Fontaine, D.K., White, D.B., Dracup, K.A., & Puntillo, K.A. (2012). Psychological symptoms of family members of high-risk intensive care unit patients. *American Journal of Critical Care, 21,* 386-393. PMID:23117902
12. McCarthy, M.J., Lyons, S.L., Powers, L.E. (2012). Relational factors associated with depressive symptoms among stroke survivor-spouse dyads. *Journal of Family Social Work* 15: 303-320.
13. Shaffer KM, Riklin E, Stagl JM, Rosand J, Vranceanu AM. (2016) Psychosocial resiliency is associated with lower psychological distress among dyads of patients and their informal caregivers in the neuroscience intensive care unit. J Critical Care 2016 Jul 16; 36:154-159. PMID: 27546765
14. Schaffer KM, Jacobs JM, Coleman JN, Rosand J, Temel, J, Greer JA. Vranceanu AM**.** Anxiety and depressive symptoms among two seriously ill medical populations and their family caregivers. Neurocritical Care 2016; ahead of print.
15. Zale EL, Piere-Louis C, Tehan T, Henhuis T, Rosand J., Vranceanu, A.M. Improving resiliency and brain health after acute neurological illness; Perceptions of patients and families. Accepted for presentation at Brain Health Conference, Columbus, OH April 28-30, 2017.
16. Martire LM, Lustig AP, Schulz R, Miller GE, Helgeson VS. Is it beneficial to involve a family member? A meta-analysis of psychosocial interventions for chronic illness. *Health Psychol* 2004; 23(6):599-611
17. Bonanno, G. A., Galea, S., Bucciarelli, A., & Vlahov, D. (2007). What predicts psychological resilience after disaster? The role of demographics, resources, and life stress. *Journal of Consulting and Clinical Psychology*, *75*,671–682. http://dx.doi.org/10.1037/0022-006X.75.5.671
18. Brown KW, Ryan RM (2003) The benefits of being present: mindfulness and its role in psychological well-being.J Pers Soc Psychol 84(4):822
19. Donnellan C, Hevey D, Hickey A, O'Neil D (2006) Defining and quantifying coping strategies after stroke: a review. J Neurol Neurosurg Psychiatry 77:1208-1218
20. Southwick SM, Vythilingam M, Charney DS (2005) The psychobiology of depression and resilience to stress:implications for prevention and treatment. Annu Rev Clin Psychol 1:255-291
21. Korpershoek C, van der Bijl J, Hafsteinsdóttir TB (2011) Self-efficacy and its influence on recovery of patients with stroke: a systematic review. J Adv Nurs 67(9):1876-1894
22. Savini, S., Buck, H.G., Dickson, V.V., Simeone, S., Pucciarelli, G., Fida, R., ... Vellone, E. (2015). Quality of life in stroke survivor-caregiver dyads: a new conceptual framework and longitudinal study protocol. *Journal of Advanced Nursing, 71*(3), 676-687. PMID: 25186274
23. Barclay-Goddard, R., King, J., Dubouloz, C.J., Schwartz, C.E., & Response Shift Think Tank Working Group. (2012). Building on transformative learning and response shift theory to investigate health-related quality of life changes over time in individuals with chronic health conditions and disability. *Archives of Physical Medicine and Rehabilitation, 93*, 214-220. PMID: 22289229
24. Shields, C.G., King, D.A., & Wynne, L.C. (1995). Interventions with later life families. In R.H. Mikesell, D.D. Lustermann, & S.H. McDaniel (Eds.), *Integrating Family Therapy: Handbook of Family Psychology and Systems Theory* (pp. 141-158). Washington, DC: American Psychological Association.
25. Tehan T., Zale E., Rosand J, Vranceanu AM. Perceptions of needs and recommendations for implementation of a resiliency intervention for patients with stroke and their families; Nurses speak. Accepted for presentation at Brain Health Conference, Columbus, OH April 28-30, 2017
26. http://www.who.int/mental_health/media/en/545.pdf. Accessed March 3rd, 2017.

27. Collins PY, Patel V, Joestl SS, March D, Insel TR, Daar A, on behalf of the Grand Challenges in Global Mental Health Scientific Advisory Board and Executive Committee. Grand Challenges in Global Mental Health. *Nature.* 2011 July 7. 474(7354):27-30. PMID 21734685
28. Edmondson, D., Richardson, S., Fausett, J.K., Falzon, L., Howard, V.J., Kronish, I.M. (2013). Prevalence of PTSD in survivors of stroke and transient ischemic attack: a meta-analytic review. *PLoS One, 8,* e66435. PMCID: PMC3686746
29. Ayerbe, L., Ayis, S., Wolfe, C.D., Rudd, A.G. (2013). Natural history, predictors and outcomes of depression after stroke: systematic review and meta-analysis. *The British Journal of Psychiatry, 202,* 14-21. PMID:23284148
30. Carod-Artal, F.J., Egido, J.A. (2009). Quality of life after stroke: the importance of a good recovery. *Cerebrovascular Diseases, 27,* 204-214. PMID: 19342853
31. Bartoli, F., Lillia, N., Lax, A., Crocamo, C., Mantero, V., Carrà, G., Agostoni, E. Clerici, M. (2013). Depression after stroke and risk of mortality: a systematic review and meta-analysis. *Stroke Research and Treatment, 2013,* 862978. PMCID: PMC3606772
32. Denno, M.S., Gillard, P.J., Graham, G.D., DiBonaventura, M.D., Goren, A., Varon, S.F., Zorowitz, R. (2013). Anxiety and depression associated with caregiver burden in caregivers of stroke survivors with spasticity. *Archives of Physical Medicine and Rehabilitation, 94,*1731-1736. PMID: 23548544
33. Bakas, T., Burgener, S.C. (2002). Predictors of emotional distress, general health, and caregiving outcomes in family caregivers of stroke survivors. *Topics in Stroke Rehabilitation, 9,* 34-45. PMID: 14523721
34. Monin, J., Doyle, M., Levy, B., Schulz, R., Fried, T., Kershaw, T. (2016). Spousal associations between frailty and depressive symptoms: longitudinal findings from the cardiovascular health study. *Journal of the American Geriatrics Society, 64,* 824-830. PMID: 27100578
35. Lee, S,, Colditz, G.A., Berkman, L.F., Kawachi, I. (2003). Caregiving and risk of coronary heart disease in US women: a prospective study. *American Journal of Preventative Medicine, 24,*113-119. PMID: 12568816
36. Ji, J., Zöller, B., Sundquist, K., Sundquist, J. (2012). Increased risks of coronary heart disease and stroke among spousal caregivers of cancer patients. *Circulation, 125,* 1742-1747. PMID: 22415143
37. Schulz, R., Beach, S.R. (1999). Caregiving as a risk factor for mortality: the Caregiver Health Effects Study. *JAMA, 282*, 2215-2219. PMID: 10605972
38. Beach, S.R., Schulz, R., Williamson, G.M., Miller, L.S., Weiner, M.F., Lance, C.E. (2005). Risk factors for potentially harmful informal caregiver behavior. *Journal of the American Geriatrics Society, 53,* 255-261. PMID:15673349
39. Turner-Stokes, L., Hassan, N. (2002). Depression after stroke: a review of the evidence base to inform the development of an integrated care pathway. Part 1: Diagnosis, frequency and impact. *Clinical Rehabilitation, 16,* 231-247. PMID: 12017511
40. Schubart, J.R., Kinzie, M.B., & Farace, E. (2008). Caring for the brain tumor patient: family caregiver burden and unmet needs. *Neuro-oncology, 10,* 61-72. PMCID: PMC2600839
41. Palmer, S., & Glass, T.A. (2003). Family function and stroke recovery: a review. *Rehabilitation Psychology, 48,* 255-265.
42. Perrin, P.B., Heesacker, M., Hinojosa, M.S., Uthe, C.E., & Rittman, M.R. (2009). Identifying at-risk, ethnically diverse stroke caregivers for counseling: a longitudinal study of mental health. *Rehabilitation Psychology, 54,* 138-149. PMID: 19469603

43. Bienvenu, O.J., Colantuoni, E., Mendez-Tellez, P.A., Dinglas, V.D., Shanholtz, C., Husain, N., ... Needham,
D.M. (2012). Depressive symptoms and impaired physical function after acute lung injury. *American Journal of Respiratory and Critical Care Medicine, 185,* 517-524. PMCID: PMC3297105
44. Martin, L.R., Williams, S.L., Haskard, K.B., & DiMatteo, M.R. (2005). The challenge of patient adherence. *Therapeutics and Clinical Risk Management, 1,* 189-199. PMCID: PMC1661624
45. Rees, J., O'Boyle, C., & MacDonagh, R. (2001). Quality of life: impact of chronic illness on the partner. *Journal of the Royal Society of Medicine, 94,* 563-566. PMCID: PMC1282240
46. Azoulay, E., Pochard, F., Kentish-Barnes, N., Chevret, S., Aboab, J., Adrie, C., ... FAMIREA Study Group. (2005). Risk of post-traumatic stress symptoms in family members of intensive care unit patients. *American Journal of Respiratory and Critical Care Medicine*, *171,* 987-994. PMID: 15665319
47. Jones, C., Skirrow, P., Griffiths, R.D., Humphris, G., Ingleby, S., Eddleston, J., ... Gager, M. (2004). Posttraumatic stress disorder-related symptoms in relatives of patients following intensive care. *Intensive Care Medicine, 30,* 456-460. PMID: 14767589
48. Jones, C., & Griffiths, R.D. (2007). Patient and caregiver counselling after the intensive care unit: what are the needs and how should they be met? *Current Opinion in Critical Care, 13,* 503-507. PMID: 17762226
49. Im, K., Belle, S.H., Shulz, R., Mendelsohn, A.B., Chelluri, L., & QOL-MV Investigators. (2004). Prevalence and outcomes of caregiving after prolonged (> or =48 hours) mechanical ventilation in the ICU. *Chest*, *125,* 597-606. PMID: 14769744
50. Douglas, S.L., Daly, B.J., Kelley, C.G., O'Toole, E., & Montenegro, H. (2005). Impact of a disease management
program upon caregivers of chronically ill patients. *Chest, 128,* 3925-3936. PMID: 16354865
51. Martire LM, Lustig AP, Schulz R, Miller GE, Helgeson VS. Is it beneficial to involve a family member? A metaanalysis of psychosocial interventions for chronic illness. *Health Psychol* 2004; 23(6):599-611
52. Bakas, T., Clark, P.C., Kelly-Hayes, M., King, R.B., Lutz, B.J., Miller, E.L., & American Heart Association Council on Cardiovascular and Stroke Nursing and the Stroke Council. (2014). Evidence for stroke family caregiver and dyad interventions: a statement for healthcare professionals from the American Heart Association and American Stroke Association. *Stroke, 45*, 2836-2852. PMID: 25034718
53. Barclay-Goddard, R., King, J., Dubouloz, C.J., Schwartz, C.E., & Response Shift Think Tank Working Group. (2012). Building on transformative learning and response shift theory to investigate health-related quality of life changes over time in individuals with chronic health conditions and disability. *Archives of Physical Medicine and Rehabilitation, 93*, 214-220. PMID: 22289229
54. Cook, W.L. & Kenny, D.A. (2005). The Actor-Partner Interdependence Model: A model of bidirectional effects in developmental studies. *Journal of Behavioral Development*, *29*, 102-109.
55. Zale EL, McCurley JL, Lin A, Funes C, Tehan T, Henhuis T, Rosand J., Vranceanu, A.M. Early psychological distress is crossectionally and prospectively interdependent between patients admitted to the Neuro-ICU and their family caregivers. Submitted for presentation at Society of Behavioral Medicine, New Orleans, April 28-30, 2018.
56. Richards, K.C., Enderlin, C.A., Beck, C., McSweeney, J.C., Jones, T.C., & Roberson, P.K. (2007). Tailored biobehavioral interventions: a literature review and synthesis. *Research and Theory for Nursing Practices, 21*, 271-285. PMID: 18236771

57. Vranceanu AM, Ricklin E, Merker V, Park E, Plotkin SR. (2016) Mind body therapy for patients with
neurofibromatosis via live video; An RCT. Neurology 87 (8):806-14.
58. Zale EL, Piere-Louis C, Riklin E, Macklin E, Vranceanu AM. The impact of a mind body program on multiple dimensions of resiliency in geographically diverse patients with neurofibromatosis. JCCP accepted.
59. Bellg AJ, Borrelli B, Resnick B, et al. (2004). Enhancing treatment fidelity in health behavior change studies: Best practices and recommendations from the NIH Behavior Change Consortium. *Health Psychol.* 23:443-451. PMID:15367063.
60. Vranceanu AM**,** Merker VL, Plotkin SR, Park ER. The Relaxation Response Resiliency Program (3RP) in patients with neurofibromatosis 1, neurofibromatosis 2, and schwannomatosis: results from a pilot study. J Neurooncol. 2014; 120(1): 103-109.
61. Rounsavile, B.J., Carroll, K.M., & Onken, L.S. (2001). A stage model of behavioral therapies research: getting started and moving on from stage I. *Clinical Psychology: Science and Practice, 8*, 133-142.
62. Larsen, D.L., Attkisson, C.C., Hargreaves, W.A., & Nguyen, T.D. (1979). Assessment of client/patient satisfaction: development of a general scale. *Evaluation and Program Planning, 2,* 197-207. PMID: 10245370
63. Devilly, G.J., & Borkovec, T.D. (2000). Psychometric properties of the credibility/expectancy questionnaire. *Journal of Behavior Therapy and Experimental Psychiatry, 31,* 73-86. PMID: 11132119
64. Zigmond, A.S. & Snaith, R.P. (1983). The hospital anxiety and depression scale. *Acta Psychiatrica Scandinavica, 67,* 361-370. PMID: 6880820
65. Bhandari, N.J., Jain, T., Marolda, C., & ZuWallack, R.L. (2013). Comprehensive pulmonary rehabilitation results in clinically meaningful improvements in anxiety and depression in patients with chronic obstructive pulmonary disease. *Journal of Cardiopulmonary Rehabilitation and Prevention, 33,* 123-127. PMID: 23399845
66. Blanchard, E.B., Jones-Alexander, J., Buckley, T.C., & Forneris, C.A. (1996). Psychometric properties of the PTSD Checklist (PCL). *Behaviour Research and Therapy, 34,* 669-673. PMID: 8870294
67. Monson, C.M., Gradus, J.L., Young-Xu, Y., Schnurr, P.P., Price, J.L., & Schumm, J.A. (2008). Change in posttraumatic stress disorder symptoms: do clinicians and patients agree? *Psychological Assessment, 20,* 131-138. PMID: 18557690
68. Feldman, G., Hates, A., Kumar, S., Greeson, J., & Laurenceau, J.P. (2007). Mindfulness and emotion regulation: The development and initial validation of the Cognitive and Affective Mindfulness Scale Revised (CAMS-R). *Journal of Psychopathology and Behavioral Assessment, 29,* 177-190.
69. Schwarzer, R. & Jerusalem, M. (1995). Generalized Self-Efficacy scale. In J. Weinman, S. Wright, & M. Johnston (Eds.), *Measures in health psychology: A user's portfolio. Causal and control beliefs* (pp. 35-37). Windsor, UK: NFER-NELSON.
70. Cohen, S., Mermelstein, R., Kamarck, T., & Hoberman, H.M. (1985). Measuring the functional components of social support. In I.G. Sarason & B.R. Sarason (Eds.), *Social Support: Theory, Research and Applications* (pp.73-94). Netherlands: Springer.
71. Carver CS. (2006). Measure of Current Status. http://www.psy.miami.edu/faculty/ccarver/sclMOCS.html

72. Merz, EL, Roesch, SC, Malcarne, VL, Penedo, FJ, Llabre, MM, Weitzman, OB, ... Johnson, TP. (2013). Validation of interpersonal support evaluation list-12 (ISEL-12) scores among English- and Spanish-speaking Hispanics/Latinos from the HCHS/SOL sociocultural ancillary study. *Psychological Assessment* 26(2): 384-394.
73. Wilhelm, K. & Parker, G. (1988). The development of a measure of intimate bonds. *Psychological Medicine, 18*, 225-234. PMID: 3363041
74. Guest, G., Bunce, A., & Johnson, L. (2006). How many interviews are enough? An experiment with data saturation and variability. *Field Methods*, *18*(1), 24. doi: 10.1177/1525822X05279903
75. Browne RH. On the use of a pilot sample for sample size determination. *Stat Med*.1995;14: 1933-40. PMID:8532986.
76. Lancaster GA, Dodd S, Williamson PR. (2004). Design and analysis of pilot studies: recommendations for good practice. *J Eval Clin Pract*.;10:307-12. PMID: 15189396.
77. Rounsaville BJ, Caroll KM, Onken LS. (2001)A stage model of behavioral therapies research: getting started and moving on from stage I. *Clin Psychol Sci Pract*. 8:133–142
78. Shih WJ, Ohman-Strickland PA, Lin Y. (2004) Analysis of pilot and early phase studies with small sample sizes. *Stat Med.* 23:1827–1842 PMID: 15195318.
79. Whitehead AL, Julious SA, Cooper CL, Campbell MJ (2016). Estimating the sample size for a pilot randomized trial to minimise the overall trial sample size for the external pilot and main trial for a continuous outcome variable. *Stat Methods Med Res* 25:1057-1073. PMID: 26092476.
80. Miles, H.B., Huberman, A.M.(1994). *Qualitative Data Analysis.* Sage Publication, Thousand Oaks California.
81. Bowen DJ, Kreuter M, Spring B, et al. (2009) How we design feasibility studies. *Am J Prev Med.* May;36(5):452-7. PMID: 19362699.
82. Schafer JL, Graham JW. (2002). Missing data: our view of the state of the art. *Psychol Methods* 7(2):147-77. PMID: 12090408.
83. National Alliance for Caregiving (NAC). (2015). *Caregiving in the U.S. 2015.* Bethesda, MD: AARP Public Policy Institute.
84. Ruskin, P.E., Silver-Aylaian, M., Kling, M.A., Reed, S.A., Bradham, D.D., Hebel, J.R., ... Hauser, P. (2004). Treatment outcomes in depression: comparison of remote treatment through telepsychiatry to in-person treatment. *The American Journal of Psychiatry, 161,* 1471-1476. PMID: 15285975
85. Carlbring, P., & Andersson, G. (2006). Internet and psychological treatment. How well can they be combined? *Computers in Human Behavior, 22*, 545-553.
86. Clough, B.A., & Casey, L.M. (2011). Technological adjuncts to increase adherence to therapy: a review. *Clinical Psychology Review, 31,* 697-710. PMID: 21497153
87. Lin A, Jacobo M, Jacobs J, Tehan T, Salgueiro D, Rosand J, Vranceanu AM, Zale E. Gender differences in emotional distress among caregivers of patients admitted to the Neuroscience-Intensive Care. Submitted to the Society of Behavioral Medicine Annual Meeting, New Orleans, April 2018
88. Blake H, McKinney K, Treece E, Lee NB. (2002) An evaluation of screening measures for cognitive functioning after stroke. Age and Ageing, 31, pp.451-456.

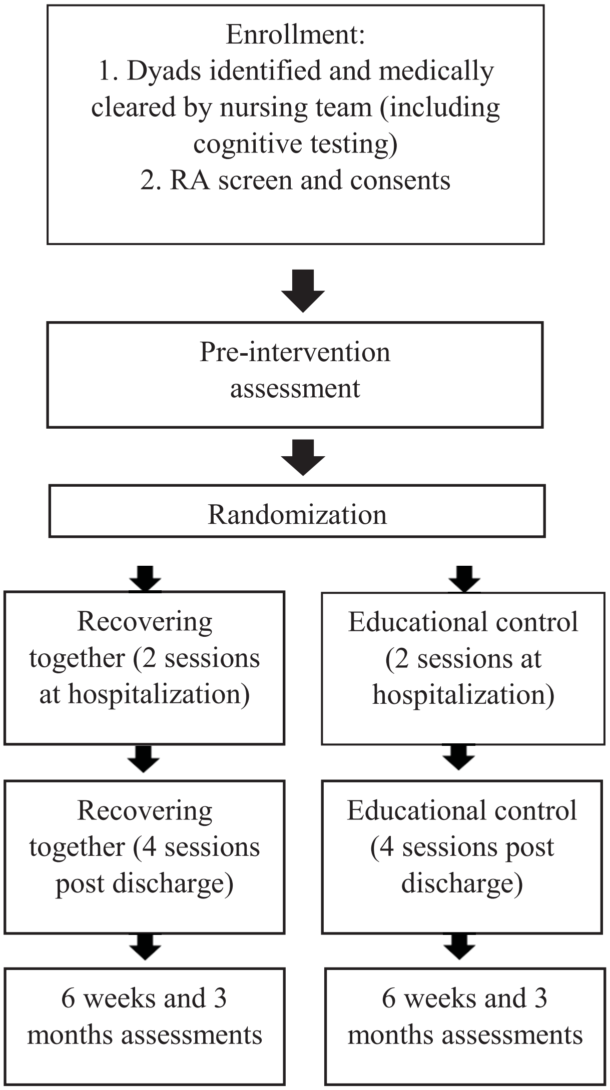
Enrollment:
1. Dyads identified and medically cleared by nursing team (including cognitive testing)
2. RA screen and consents
Pre-intervention assessment
Randomization
Recovering together (2 sessions at hospitalization)
Educational control (2 sessions at hospitalization)
Recovering together (4 sessions post discharge)
Educational control (4 sessions post discharge)
6 weeks and 3 months assessments
6 weeks and 3 months assessments

Partners Human Research
Partners HealthCare
399 Revolution Drive, Suite 710
Somerville, MA 02145
Tel: 857-282-1900
Fax: 857-282-5693

Notification of IRB Review

Protocol #: 2018P002187

Date:	December 31, 2018
To:	Vranceanu, Ana-Maria,<br>MGH<br>Partners > MGH > Psychiatry
From:	Partners Human Research<br>399 Revolution Drive, Suite 710<br>Somerville, MA 02145
Title of Protocol:	Recovering Together: Building resiliency in dyads in patients admitted to the Neuroscience Intensive Care Unit (NICU) and their caregivers
Version/Number:	1
Version Date:	09/24/2018
IRB Amendment #:	1
IRB Review Type:	Expedited
IRB Approval Date:	12/28/2018
Approval/Activation Date:	12/31/2018

This project has been reviewed and approved by the **PHS IRB**. During the review of this project, the IRB specifically considered (i) the risks and anticipated benefits, if any, to subjects; (ii) the selection of subjects; (iii) the procedures for obtaining and documenting informed consent; (iv) the safety of subjects; and (v) the privacy of subjects and confidentiality of the data.

Please note that if an IRB member had a conflict of interest with regard to the review of this project, consistent with IRB policies and procedures, the member was required to recuse him/herself and, if applicable, leave the room during the discussion and vote on this project except to provide information requested by the IRB.

GENERAL REVIEW COMMENTS

- This approval covers an amendment to add additional measures; adding recruitment video; adding option for patients to opt into text message reminders; and changing MMSE administrator from nurses to trained study staff.

ANCILLARY COMMITTEES

1. **RISO (PHS) : Approved**

Partners Human Research
Partners HealthCare
399 Revolution Drive, Suite 710
Somerville, MA 02145
Tel: 857-282-1900
Fax: 857-282-5693

As Principal Investigator, you are responsible for ensuring that this project is conducted in compliance with all applicable federal, state and local laws and regulations, institutional policies, and requirements of the IRB, which include, but are not limited to, the following:

1. Submission of any and all proposed changes to this project (e.g., protocol, recruitment materials, consent form, status of the study, etc.) to the IRB for review and approval prior to initiation of the change(s), except where necessary to eliminate apparent immediate hazards to the subject(s). Changes made to eliminate apparent immediate hazards to subjects must be reported to the IRB as an unanticipated problem.
2. Submission of continuing review submissions for re-approval of the project prior to expiration of IRB approval and a final continuing review submission when the project has been completed.
3. Submission of any and all unanticipated problems, including adverse event(s) in accordance with the IRB's policy on reporting unanticipated problems including adverse events.
4. Obtaining informed consent from subjects or their legally authorized representative prior to initiation of research procedures when and as required by the IRB and, when applicable, documenting informed consent current IRB approved consent form(s) with the IRB-approval stamp in the document footer.
5. Informing all investigators and study staff listed on the project of changes and unanticipated problems, including adverse events, involving risks to subjects or others.
6. When investigator financial disclosure forms are required, submitting updated financial disclosure forms for yourself and for informing all site responsible investigators, co-investigators and any other members of the study staff identified by you as being responsible for the design, conduct, or reporting of this research study of their obligation to submit updated Investigator Financial Disclosure Forms for this protocol to the IRB if (a) they have acquired new financial interests related to the study and/or (b) any of their previously reported financial interests related to the study have changed.

IMPORTANT REMINDER: THE IRB HAS THE AUTHORITY TO TERMINATE PROJECTS THAT ARE NOT IN COMPLIANCE WITH THESE REQUIREMENTS.

Questions related to this project may be directed to **Ednice, Monteiro | Tel: 282-1916 | Email: EEMONTEIRO@PARTNERS.ORG**

cc:

Ana-Maria, Vranceanu, , Psychiatry, Psychiatry, Principal Investigator

Sofia, Distefano, BS, Neurology, Neurology, Research Coordinator/Manager

Ann, Lin, , Psychiatry, Psychiatry, Research Coordinator/Manager

Melissa, Gates, , Psychiatry, Psychiatry, Research Coordinator/Manager

PARTNERS HUMAN RESEARCH COMMITTEE DETAILED PROTOCOL

Principal Investigator: Ana-Maria Vranceanu, PhD

Protocol Title: Recovering Together: Building resiliency in dyads in patients admitted to the Neuroscience Intensive Care Unit (NICU) and their caregivers

Funding: National Institute of Nursing Research

Version Date: 10/31/2018

I. BACKGROUND AND SIGNIFICANCE

Acute neurological illnesses (ANIs) are common, costly and often lead to long-term disability. ANIs are biologically distinct injuries that disrupt the normal function of the brain. The most common ANIs in Neuroscience Intensive Care Units (NICU) include cerebrovascular (stroke/hemorrhage and brain aneurysm), structural (tumors and lesions/brain masses), and traumatic (TBI) brain injuries. NICU admissions for ANIs are prevalent (e.g., 795,000 acute stroke/year; 275,000 acute TBI/year) and costly[1,2]; post NICU prolonged rehabilitation is common[3].

ANIs are associated with chronic emotional distress in both patients (pts) and caregivers (cgs). Although biologically heterogeneous, ANIs are unified by sudden onset, and substantial emotional distress in both pts (e.g., 12-43% anxiety[4-7]; 10-58% depression[4,7,8]; 20-29% post-traumatic stress PTS[5,9]) and family cgs (27-60% depression, anxiety or PTS [4,10,11]).
These symptoms often become chronic and treatment resistant[26,27].

Pt and cg factors interact and influence physical and emotional outcomes in both pts and cgs. Post ANI emotional distress is associated with pts' poor medical adherence[28], slower recovery[28-30], higher mortality[29-31], and need of more caregiving assistance[32], which further increase cgs' distress[30,33,34] and own risk for morbidity[35,36] and mortality[37]; in turn cgs' emotional distress interferes with ability to provide high-quality care to pts[38,39] and negatively impacts pts' outcomes.

Current management of ANIs does not meet the psychological needs of pts and cgs for 3 reasons[40-51]. First, although recognition of the emotional burden associated with NICU admission has increased, and some NICUs have social workers available to assist pts and cgs, there are no formal screening methods for emotional distress routinely integrated in practice during hospitalization, when the primary focus is on medical care and survival; further, there are no formal evidence-based treatments integrated within the medical care. When social workers are included to help pts and cgs, the care is brief and occurs only during hospitalization. When referrals to mental health services are provided to families at discharge, few will access additional treatment due to burden associated with traveling outside of home. Second, psychosocial interventions available for ANI pts or cgs are limited in that they are delivered when symptoms are already chronic, address only one emotional illness (e.g., depression or anxiety or PTS), and/or are focused on a *single member* of the pt-cg dyad. Even interventions labeled as "dyadic," which include pts and cgs, typically address only the pts' needs and do not

focus on cg outcomes or on the dyad's interpersonal communication and bond/relationship[12,52]. These interventions are not consistent with the *dyadic framework*[22] which specifies that dyadic interventions should account for the interdependence between pt and cg psychosocial factors including their interpersonal bond by ensuring that both pts and cgs attend each session together, and by targeting improvement in outcomes for both pts and cgs. Third, most interventions are delivered using uniform protocols. However, the needs of ANI dyads are heterogeneous due to varying levels of post ANI impairment, identity of the cg, context and stage of life. A recent systematic review[52] urged for the development of dyadic interventions that address the needs of *both* pts and cgs and are tailored to the specific needs of each ANI dyad.

We developed the first dyadic skills-based intervention – Recovering Together - to prevent chronic heightened emotional distress in at risk ANI pt-cg dyads. The "Recovering Together" program is informed by the theoretical response-shift framework of adaptation to acute illness[53] (successful adaptation implies recalibration of values and life goals), the family strength vulnerability model[24] (within dyads relational systems have strengths and weaknesses in how they cope with life events), the dyadic longitudinal model[22] (distress travel from one member of the dyad to the other across time), the APIM model[54], and the resiliency framework[17]**.** The program is in line with recent recommendations for skill-based interventions for critical care patients, and uses preliminary data collected by our team for the past 3 years[4, 13-15]. The intervention teaches pts and cgs resiliency factors that are associated with well-being after trauma for both pts and cgs: *mindfulness* – the ability to stay present and defer judgment in the face of adversity[18]; *coping*– the arsenal and application of one's behavioral, cognitive, and emotional strategies to manage stress[19]; *social support* –empathetic interpersonal interactions that meet one's emotional and functional needs[20]; *self-efficacy* – perceived ability to adapt under adversity[21] and *positive dyadic interpersonal communication to increase interpersonal bond*[12]. Informed by the aforementioned theoretical models, our conceptual model hypothesizes that by teaching both members of the dyad resiliency and interpersonal communication skills (e.g., Recovering Together) we will be able to sustainably decrease emotional distress in both members of the dyad

In addition, our team has an established record of collaboration on published or ongoing investigations. **Emotional distress is prevalent in dyads, interdependent between pt and cg, and negatively associated with resiliency factors[4].** Our team conducted a cross-sectional study of pt-cg dyads in the NICU (40% stroke, 30% tumor). 75% pts and 84% cgs approached agreed to participate. 74% pts had been intubated at one time during NICU admission, and 2/3 were discharged home. Rates of clinically significant symptoms of depression, anxiety and PTS did not differ between pts (24%, 43%, 21%) and cgs (24%, 46%, 17%), or by any demographic or medical characteristic. Dyadic modeling showed that for both pts and cgs, mindfulness and coping impacted both self and partner's emotional distress symptoms. We showed: 1) feasibility of recruitment; 2) high emotional distress in dyads; and that 3) modifiable resiliency factors (mindfulness and coping) are intervention targets interdependently associated with distress in pts and cgs, regardless of the identity of cg (e.g., spouse, friend, etc).

Resiliency factors are associated with lower emotional distress in NICU dyads[13]. Our team found that resiliency factors of mindfulness, coping, self-efficacy and patient-caregiver interactions were associated with decreased emotional distress in dyads of ANI. This study confirms mindfulness, coping as intervention targets and provides novel evidence on self-efficacy and patient-caregiver interaction as additional important intervention targets.

ANI pts have greater anxiety than cancer patients at early diagnoses[14]. We led the first cross-comparison study of emotional distress among dyads with ANI and cancer. This study supports the priority of addressing emotional distress in ANI dyads as has been emphasized for cancer dyads.

Clinically significant emotional distress in one member of the dyad at hospitalization predicts chronic emotional distress in at least 1 of the dyad members 3 and 6 months later[55]. Our team has an ongoing prospective study of dyads with ANI. Retention rates for dyads due for assessments at 3 and 6 months thus far are 84% and 91% for pts and 87.7% and 95.7% for cgs, confirming our ability to retain post-ANI participants. Within each dyad, if one member screens in for clinically significant symptoms for any diagnosis (i.e., depression, anxiety or PTSD) at hospitalization there is good sensitivity and specificity that one member of the dyad will endorse clinically significant symptoms 3 months later. This study shows a reliable method for identifying dyads of patients at risk for chronic heightened emotional distress by identifying dyads in which either the pt or cg screens in for heightened emotional distress (symptoms of depression, anxiety or PTSD).

Caregiver gender moderates the prospective association of resiliency factors to emotional distress[87]. This study found that at the time of admission resiliency factors have main effects on emotional distress, with no differences by cg gender. However, significant interaction effects emerged prospectively such that male cgs with high mindfulness at baseline demonstrated lower levels of emotional distress at 3 and 6 months later than did males with low mindfulness ($p = 0.026$ and $p <0.013$). Similarly, women cgs with high intimate bond at baseline reported the lowest levels of depression symptoms 3 and 6 months later ($p < 0.020$). This study confirmed the need to assess and address resiliency factors early in the recovery process, and identified important gender differences to be accounted for in intervention development.

Recovering Together; Developing a novel dyadic resiliency skills program for ANI pt-cg dyads at risk for chronic emotional distress[15]. With funding from American Heart Association, we conducted 20 qualitative interviews with pt-cg stroke dyads at risk for chronic emotional distress during NICU hospitalization. We also conducted additional clinical interviews with 10 pt-cg dyads representative of other ANI diagnoses. 83% dyads approached agreed to participate. Pts (23) and cgs (25) were mostly women. Dyads were mostly spouses and mothers-daughter. Data was analyzed with Nvivo10. Main themes did not differ by medical diagnoses: 1) most challenging and distressing experiences: uncertainty about future, anxiety, depression, sleep difficulties, worries about the future, guilt, managing job with caretaking, making treatment decisions, lack of predictability; 2) concerns about interpersonal relationships (self-image, role changes, role fulfillment); 3) fear of recurrence; 4) adjusting to sequelae. Dyads noted interest in a resiliency program (30/30) and preferred a combination of in person and live video sessions (30/30). Dyads learned about resiliency skills (e.g., name, description and goal) and agreed they would be helpful to them. They also noted interest in learning about: survivorship plans, adaptation to deficits (present or anticipated), and return to normal living. We found no thematic differences between stroke dyads and other ANI dyads. Challenges associated with embracing the caregiver role emerged as a theme while differences by the identity of caregiver (e.g., spouse vs. friend, vs parent) did not. Themes associated with the gender of the cg emerged and have been incorporated in the intervention.

Nurses perception of the needs for and feasibility of "Recovering Together for ANI families[25]. We conducted 2 focus groups (N =15) with NICU nurses who provided feedback on the qualitative findings from our 30 ANI dyads and shared own experiences and opinions on

implementation and scalability of the intervention including nurse involvement. Nurses concurred with pts experiences, and suggested strategies to recruit and retain dyads for the study, which are now included in the methodology section of the current grant proposal. Studies 3.6 and 3.7 represent the building blocks for the development of our Recovering Together program and manual. Nurses provided edits and contributed to the iterative development of the manual. These studies also confirm feasibility of conducting the pilot RCT proposed through this R21.

II. SPECIFIC AIMS

The current study has the following objectives:

Aim 1: **To determine the feasibility of recruitment, feasibility of program delivery, program credibility, and program satisfaction using evidence-based benchmarks.**

Hypothesis 1: We hypothesize that > 75% of the dyads approached will agree to participate.
Hypothesis 2: We hypothesize that > 75% of dyads who start the intervention will complete at least 4 sessions.
Hypothesis 3: We hypothesize that > 75% of participants will report average credibility (Credibility and Expectancy Questionnaire) scores greater than the scale's midpoint.
Hypothesis 4: We hypothesize that > 75% participants will report average satisfaction (Client Satisfaction Scale) scores greater than the scale's midpoint.

Aim 2: **To demonstrate a proof of concept that the Recovering Together program can sustainably improve emotional distress [Hospital Anxiety and Depression Scale; HADS], Post Traumatic Symptoms (PTS) [PCL-S], resiliency variables (mindfulness, coping, social support and self-efficacy) and interpersonal factors (interpersonal bond).**

Hypothesis 1: We hypothesize that participation in the Recovering Together Program will be associated with a more potent decrease in emotional distress and PTS compared to participation in the educational program (control), and that these improvements will maintain at 3 month follow up.
Hypothesis 2: We hypothesize that Recovering Together Program will be associated with a more potent increase in resiliency variables (mindfulness, coping, social support, self-efficacy) and interpersonal factors (interpersonal bond) compared to participation in the educational program, and that these improvements will maintain at 3 month follow up.

For this feasibility pilot RCT, our primary outcomes (feasibility, credibility and satisfaction) will be assessed in Aim 1. Our secondary outcomes in this trial are: emotional distress, PTS, mindfulness, coping, social support, self-efficacy, and interpersonal bond, and will be assessed in Aim 2.

III. SUBJECT SELECTION

All participants will be recruited from the Massachusetts General Hospital Neuroscience ICU, using IRB approved recruitment materials.

Inclusion/Exclusion Criteria

Eligible dyads (Pts and Cgs) must meet the following inclusion criteria:

1) Age 18 or older
2) English fluency and literacy
3) Access to high speed internet for video sessions
4) Pt with an informal cg (family or friend who provides unpaid care) available and willing to participate
5) Hospitalized with an ANI within 1-2 weeks (pt) OR primary cg of a pt currently admitted with an ANI
6) Either pt <u>or</u> cg have clinically significant symptoms of depression, anxiety, and/or PTS

One or more of the following exclusion criteria will render a pt ineligible:

1) Permanent and severe cognitive impairment severe enough to impede participation – This will be determined by trained study staff through an assessment conducted as part of usual care and that includes the MMSE (score of <23) and GCS (score of <10). Nurses and study staff are trained and use these measures as part of NICU care.
2) Dyads where the pt is anticipated to die or to never be able to participate due to medical sequelae. This will be determined by nurses.

All adult patients and family caregivers, satisfying all inclusion criteria, are eligible for enrollment in this study regardless of sex, race, or ethnicity. Vulnerable populations will not be recruited.

Recruitment

Patients will be recruited from the NICU at MGH from the medical team (nurses). Recruitment will be facilitated by the nursing team who will introduce the study to eligible dyads and who will also assess whether pts are able to consent, consistent with medical presentation, and cognitive status. Trained study staff will be administering the MMSE and nurses will administer the GSC, to all patients and will refer only dyads where the patients scores are higher than the established cut off scores on these measures. Consistent with NICU practice, nurses will administer the GSC. They will refer participants to the study only if they are cleared medically and cognitively. The team will page the RA using the secure Voalte system used by our team and nursing staff, when both the pt and cg are present and able to hear more about the study. The RA will only approach pts identified and cleared by the medical team. The RA will ensure eligibility of both pts and cgs based on the additional inclusionary and exclusionary criteria depicted above. The RA will finalize the screening and conduct informed consent**.** All participants will receive a physical copy of the informed consent form which has the contact information for the PI. If cg~~s~~ cannot be reached in person in the hospital, they will be contacted via phone for screening and completion of informed consent. Cgs will be given a copy of the consent form to review while discussing the study over the telephone. We will fax or securely e-mail the consent form prior to obtaining consent. Cg will return a signed copy of the consent form.

Patients and caregivers will be considered enrolled when they sign the consent form with the study staff. Please see details on the informed consent process in the next section, Subject Enrollment (Section IV). These procedures will be completed in a private medical space.

Participants will be explicitly informed that this intervention is a research study that does not constitute individualized, personal care. The intervention is a broad-based method of training that is not tailored to any particular individual. Should any participant seek formal mental healthcare, study staff will refer them to either MGH Psychiatry, as appropriate.

We will recruit pts with any type of ANI and their cgs, ensuring representation of all NICU diagnoses. All cgs will be recruited within the first week of the pts' hospitalization. Pt's medical team will also alert the research assistant whether they anticipate that the patient might be able to participate in the study at a future point during the hospital stay. In situations where patients are unable to consent due to the severity of the ANI, we will enroll the informal cg and return to enroll the pt as soon as their mental capacity improves. The research assistant will not approach patients who are not mentally or physically capable to participate. This study does not include participants with impaired decision making.

We will not include patients who do not have a caregiver. Informal caregivers will be designated by the health care proxy and verbally confirmed by the patient. Nurses will assist the study team with identifying the patient's health care proxy.

Eligible cases may also be identified by daily screening of Epic admission reports.

The study team will keep track of all pts and cgs approached who refuse to participate (along with reasons for refusal), as well as those who were not approached and reasons why.

When the RA approaches a patient, patients and caregivers will be given the choice of watching a short recruitment video that contains testimonials from previous patients.

The recruitment process was developed and refined through prior research for the past 2 years.

IV. SUBJECT ENROLLMENT

Participants will be referred to the study by the nursing team who will ensure that patients are medically and cognitively able to participate. Eligible dyads will next be screened, consented, and enrolled by the research assistant.

After determining eligibility, study staff will meet with potential dyads to review the informed consent document. If cgs cannot be reached in person in the hospital, they will be contacted via phone to complete informed consent. Cgs will be given a copy of the consent form to review while discussing the study over the telephone. We will fax or securely e-mail the consent form prior to obtaining consent. Cgs will return a signed copy of the consent form. After the document has been reviewed, study staff will answer any and all questions the pt or cg may have. Once all questions have been addressed, each member of the dyad will sign the consent form, which will

include a description of all study procedures, the option to receive text message reminders, information about potential risks and benefits of participation, and study contact information (including that of the IRB) in case questions arise at a later time. The consent form will also explicitly state that study participation is voluntary, and that participants may refuse to answer any questions that make them uncomfortable, and may discontinue participation at any time. In addition, participants will be assured that withdrawal from the study will not compromise their medical care in any way.

As informed consent is a continuous process, participants will be given a copy of the signed informed consent document, and will be invited to ask questions about their participation at any point over the course of the study.

Following a study enrollment and baseline assessment, dyads will be randomly assigned to either the newly developed psychosocial intervention or to the educational program (control) using a random number sequence generator to ensure comparability between groups. We will document time between assessment and intervention initiation, and analyze as a predictor of study outcomes as needed. Randomization will be developed by the statistician, without any input from the rest of the team.

V. STUDY PROCEDURES

After enrollment, participants will complete study assessments. All subjects will be given baseline psychological and behavioral assessments that will assess depression, anxiety, PTS symptoms, and other psychological constructs. Assessments will be administered online, using the REDCap system. Subjects may choose to fill out these questionnaires on-site, or to fill them out at home on a personal computer or other Internet-equipped device. This assessment includes demographic information and a battery of psychological questionnaires. We will also collect information about important clinical variables including duration of acute hospitalization. All questionnaires are itemized below:

Administered at Baseline Only:
Demographics
Prior Mental Health History questions
Credibility Questionnaire

Administered at Baseline, Post-Intervention, and Follow-up:
Medical history information from LMR (i.e., prior ANI status, current psychotropic meds, comorbid medical conditions, etc.)
Post-Traumatic Stress Disorder Checklist (PCL-S)[29]
Hospital Depression and Anxiety Scale (HADS)[30]
Measure of Current Status Part A (MOCS-A)[31]
The Cognitive and Affective Mindfulness Scale (CAMS)[32]
World Health Organization Quality of Life (WHOQOL-BREF)[33]
Dyadic Relationship Scale (DRS)
Experience in Close Relationships – Relationship Structure (ECR-RS)

Administered Post-Intervention and Follow-up Only:
Client Satisfaction Questionnaire (CSQ-8)[34] [intervention group only]

The medical team will complete the following questionnaires to assess stroke severity for each patient:
Modified Rankin Scale (mRS)[35] [Pts only]
Barthel Index[36] [Pts only]

After completing the baseline assessments, dyads will be randomized to either the newly developed psychosocial intervention or to treatment as usual.

The psychosocial intervention entails 6 sessions, each 30 minutes. Both the pt and cg participate in each session. The intervention is manualized, and teaches dyads resiliency (mindfulness, social support, self-efficacy, coping skills) and interpersonal communications (interpersonal bond) skills. The first 2 sessions will occur in person, during hospitalization. The next 4 sessions are chosen from 5 available, depending on each dyad's preference, and are delivered after discharge via secure live video. For the video sessions, dyads can participate from the same or different locations. During the in hospital sessions, dyads learn diaphragmatic breathing, mindfulness, self-care, and dialectics. During the video sessions, dyads learn how to identify negative thinking patterns and replace them with adaptive thoughts, how to communicate effectively and openly, how to engage in self-care, and how to accept things that can't be changed. Participants in the intervention group will also receive treatment as usual. This may include meeting with nurses, physical therapist**s**, medical doctors, and other members of the pt's medical team. Treatment as usual may also involve administration of SSRI to those patients with motor problems.

Those in the educational program (control) will receive general health information that mimics the Recovering Together Program, but without teaching any of the resiliency or interpersonal communication skills that are hypothesized to be responsible for improvement in emotional distress. The educational program will also have 6 sessions, 2 in-person dyadic visits in the NICU and 4 dyadic virtual visits following discharge. It controls for dose and support from clinician. Both members of the dyads participate in all sessions. The topics of each session include: education about the stress of the ANI on patient and caregiver; education on the importance of self-care; education on stress associated with discharge and home adjustment; education on the importance of following up with medical recommendations; education on interpersonal stress as part of adjustment to ANI; education on self-care. The educational program condition will ensure that patients will remain blind to intervention or control and increase confidence that improvement in outcomes are due to the active ingredients of the intervention and not confounds. Participants in the educational program will continue with their current care. This may include meeting with nurses, physical therapist**s**, medical doctors, and other members of the pt's medical team. Treatment as usual may also involve administration of SSRI to those patients with motor problems.

Participants in both groups will be given post-treatment psychological and behavioral assessments identical to those administered at baseline, in addition to the CSQ-8. As a baseline, participants will be given the option to complete post-treatment questionnaires on site or at home. Participants will be asked to complete questionnaires immediately after completion of the group intervention (T2), and 3 months (T3) after completion of the group intervention, in order to measure long term outcomes. Study staff will email these questionnaires to the participants via the REDCap system.

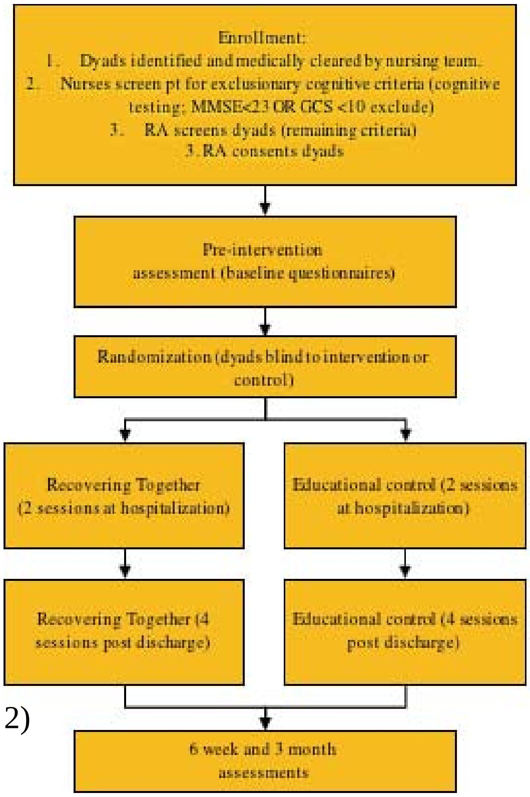

VI. BIOSTATISTIC ANALYSIS

We plan to conduct a RCT with patients from the Neuro-ICU. The main intervention goal is to provide dyads with resiliency and interpersonal communication skills necessary to optimize recovery. Dyads will be medically cleared by a member of the nursing staff. Nurses will then screen dyads for exclusionary cognitive criteria. Dyads will complete additional screening and baseline questionnaires with a trained research assistant. Dyads will be randomized to one of two groups 1) Psychosocial skills based intervention (Recovering Together), or 2) Educational Control.

We chose our study measures based on our theoretical frameworks, strong psychometric properties in studies of patients with ANI, and feasibility in our prior work with ANI dyads. Measures will be collected at baseline, post-intervention, and at 3 month follow up. Data collection and management will be conducted with Research Electronic Data Capture. Dyads will be given the option to complete measures electronically, by paper and pencil, or telephone.

1) Is the psychosocial skills-based intervention feasible, credible usable, and accepted by pts and cgs in the ICU?

Our primary aim focuses on trial feasibility, acceptability, credibility and preliminary effect. In pilot studies, ≥30 participants are recommended per group to establish feasibility and detect larger effect sizes for ≥1 outcome. We plan to recruit 80 dyads (160 participants), 40 dyads (80 participants) per arm to establish feasibility, acceptability, credibility and estimate effect size for emotional distress variables (primary quantitative outcomes). Assuming attrition of over 25% (in excess of what we experienced in our preliminary studies), we will have the necessary 30 dyads (60 participants) per arm. This size is considered to yield stable estimates of M/SDs based on prior behavioral trial recommendations. Effect sizes from this study may overestimate power in future sample calculations. We chose study measures based on our theoretical frameworks, strong psychometric properties in studies of patients with ANI, and feasibility work in our prior work with ANI dyads. Feasibility will be reported as the percentage of patients enrolled

in the study who complete at least 75% of the intervention sessions. Demonstration of feasibility will be assessed by the number of individuals who drop out of the study prior to completing the post- intervention assessment and the rate of missed sessions. If drop-out rate or missed-session rate exceeds 25%, revisions to the intervention may be needed. We will also report number of patients approached, enrolled, randomized and who completed time 2 and time 3 to determine feasibility.

2) Is the psychosocial skills-based intervention effective for pts and cgs in the ICU?

Dr. Vranceanu developed the proposed study design in collaboration with the NICU study team (nurses, physicians, clinical interns) and an MGH psychologist who specializes in using mindfulness and emotion regulation in both chronic illness and medically healthy populations. With funding from the American Heart Association, we conducted 20 qualitative interviews with pt-cg stroke dyads at risk for chronic emotional distress during NICU hospitalization. We also conducted additional clinical interviews with 10 pt-cg dyads representative of other ANI diagnoses. 83% of dyads approached agreed to participate. Pts (23) and cgs (25) were mostly women. Dyads were mostly spouses and mothers-daughter. Dyads noted interest in a resiliency program (30/30) and preferred a combination of in person and live video sessions (30/30). Dyads learned about resiliency skills (name, description, and goal) and agreed they would be helpful to them. They also noted interest in learning about: survivorship plans, adaptation to deficits (present or anticipated), and return to normal living. This led to the development and subsequent refinement (through feedback from the nursing team) of the Recovering Together Program active intervention.

3) Is the effect of the skills-based intervention for pts and cgs in the ICU durable?

We will assess feasibility, usability, and acceptability by the enrollment numbers, participants completion in at least 4 out of 6 sessions, and the questionnaires, The Credibility and Expectancy and Client Satisfaction Scale. These will be primary outcomes. The PCL-S, HADS, and resiliency measures will serve as the secondary outcomes.

We will use student’s t-test to assess within-group differences in long-term outcomes. We will compare measures at the 3 month follow-up assessment to measures at the post-intervention assessment for both study arms.

We will also use t tests and chi squared tests to assess differences at the 3 month follow up.

VII. RISKS AND DISCOMFORTS

There is a risk that some participants may feel uncomfortable completing various psychological questionnaires or parts of the skills-based on intervention. Participants are free to withdraw from the study at any time, as the study is completely voluntary.

As in any research study, there is a small risk that confidentiality may be breached; all efforts to minimize this risk will be taken. In the unlikely event that participants will become suicidal during the duration of the study, the research assistant will contact the PI and the appropriate clinical intervention will be executed.

VIII. POTENTIAL BENEFITS

Participants in this study may observe a reduction in depression, anxiety, and/or psychological and physiological markers of stress, as well as an improvement in perceived qualify of life. It is hoped that the intervention will result in improvements across these domains. Participants may learn new techniques for managing distress and lifestyle factors that may enhance wellbeing, both in disease-specific domains as well as in their general lives. In addition, all participants will receive $20 for the completion of each of the 3 assessment points.

IX. MONITORING AND QUALITY ASSURANCE

Electronic information will be stored in REDCap (Research Electronic Data Capture), a free, secure, and HIPAA-compliant web-based application hosted by the Partners HealthCare Research Computing Enterprise Research Infrastructure & Services (ERIS) group (based at the PHS Needham corporate datacenter). Data will be stored on password protected computers that will be stored in secure locations at all times. Paper data files (with coded subject identification) will be stored in a locked filing cabinet. Only research staff will have access to these data locations.

A unique anonymous identifier will be assigned to each subject; subsequently, all data collected will be associated exclusively with this identifier. This includes all questionnaires administered over the course of the study, as well as home practice logs.

Data from this study will be stored for three years after the publication of all study results, at which time all paper data files will be shredded, and computer files will be deleted.

Data Management and Quality Control Procedures

To maximize accuracy and security, all survey data will be collected and stored on REDCap. Research staff will ensure that proper consent has been obtained before sending the REDCap survey to each participant.

REDCap (Research Electronic Data Capture) is a free, secure, HIPAA compliant web-based application hosted by the Partners HealthCare Research Computing Enterprise Research Infrastructure & Services (ERIS) group. Vanderbilt University, with collaboration from a consortium of academic and non-profit institutional partners, has developed this software toolset and workflow methodology for electronic collection and management of research and clinical study data. Data collection projects rely on a study-specific data dictionary defined by members of the research team with planning assistance from Harvard Catalyst, The Harvard Clinical and Translational Science Center EDC Support Staff. This iterative development and testing process

results in a well-planned data collection strategy for individual studies. Using REDCap, the research team can also design web-based surveys and engage potential respondents using a variety of notification methods. REDCap provides flexible features that can be used for a variety of research projects and provides an intuitive interface to enter data with real time validation (automated data type and range checks). The system offers easy data manipulation with audit trails, reports for monitoring and querying participant records, and an automated export mechanism to common statistical packages (SPSS, SAS, Stata, R/S-Plus).

Since consistency of application of the study protocol is critical to acquiring high quality data, all research personnel have undergone or will undergo a competency-based training program prior to enrolling subjects.

Data and Safety Monitoring Plan

Adverse Event Monitoring: Throughout the study subjects will be monitored for the occurrence of events defined as any undesirable experience or unanticipated risk. Lack of effect of treatment is not considered an event. All adverse events will be reported on an adverse event form. The Principle Investigator has the responsibility of reporting serious adverse events (death, life threatening illness or injury, serious injury, or permanent disability) to PHRC within 24-72 hours of notification.

X. REFERENCES

1. Center for Disease Control and Prevention. (2015). Stroke Facts: Stroke in the United States. *CDC*. Retrieved from http://www.cdc.gov/stroke/facts.htm.
2. Center for Disease Control and Prevention. (2015). *Report to Congress on Traumatic Brain Injury in the United States: Epidemiology and Rehabilitation.* National Center for Injury Prevention and Control; Division of Unintentional Injury Prevention. Atlanta, GA.
3. The Society of Critical Care Medicine. (2015). What is critical care? *SCCM*. Retrieved from www.myicucare.org.
4. Shaffer KM, Riklin E, Stagl JM, Rosand J, Vranceanu AM. (2016). Mindfulness and coping are inversely related to psychiatric symptoms in patients and informal caregivers in the Neuroscience ICU: Implications for Clinical Care. Critical *Care Medicine*, 44 2036-2038. PMID: 27513536
5. Jackson, J.C., Mitchell, N., & Hopkins, R.O. (2011). Cognitive functioning, mental health, and quality of life in ICU survivors: an overview. *Anesthesiology Clinics, 29,* 751-764. PMID: 22078921
6. Kress, J.P., Gehlbach, B., Lacy, M., Pliskin, N., Pohlman, A.S., & Hall, J.B. (2003). The long-term psychological effects of daily sedative interruption on critically ill patients. *American Journal of Respiratory and Critical Care Medicine, 168,* 1457-1461. PMID: 14525802
7. Cheung, A.M., Tansey, C.M., Tomlinson, G., Diaz-Granados, N., Matté, A., Barr, A., ... Herridge, M.S. (2006). Two-year outcomes, health care use, and costs of survivors of acute respiratory distress syndrome. *American Journal of Respiratory and Critical Care Medicine, 174,* 538-544. PMID: 16763220
8. Daydow, D.S., Gifford, J.M., Desai, S.V., Bienvenu, O.J., & Needham, D.M. (2009). Depression in general intensive care unit survivors: a systematic review. *Intensive Care Medicine, 35,* 796-809. PMID: 19165464

9. Jackson, J.C., Hart, R.P., Gordon, S.M., Hopkins, R.O., Girard, T.D., & Ely, W.E. (2007). Post-traumatic stress disorder and post-traumatic stress symptoms following critical illness in medical intensive care unit patients: assessing the magnitude of the problem. *Critical Care, 11,* R27. PMCID: PMC2151890
10. Choi, J., Hoffman, J.A., Schulz, R., Ren, D., Donahoe, M.P., Given, B., & Sherwood, P.R. (2013). Health risk behaviors in family caregivers during patients' stay in intensive care units: a pilot analysis. *American Journal of Critical Care, 22,* 41-45. PMCID: PMC4109809
11. McAdam, J.L., Fontaine, D.K., White, D.B., Dracup, K.A., & Puntillo, K.A. (2012). Psychological symptoms of family members of high-risk intensive care unit patients. *American Journal of Critical Care, 21,* 386-393. PMID:23117902
12. McCarthy, M.J., Lyons, S.L., Powers, L.E. (2012). Relational factors associated with depressive symptoms among stroke survivor-spouse dyads. *Journal of Family Social Work* 15: 303-320.
13. Shaffer KM, Riklin E, Stagl JM, Rosand J, Vranceanu AM. (2016) Psychosocial resiliency is associated with lower psychological distress among dyads of patients and their informal caregivers in the neuroscience intensive care unit. J Critical Care 2016 Jul 16; 36:154-159. PMID: 27546765
14. Schaffer KM, Jacobs JM, Coleman JN, Rosand J, Temel, J, Greer JA. Vranceanu AM**.** Anxiety and depressive symptoms among two seriously ill medical populations and their family caregivers. Neurocritical Care 2016; ahead of print.
15. Zale EL, Piere-Louis C, Tehan T, Henhuis T, Rosand J., Vranceanu, A.M. Improving resiliency and brain health after acute neurological illness; Perceptions of patients and families. Accepted for presentation at Brain Health Conference, Columbus, OH April 28-30, 2017.
16. Martire LM, Lustig AP, Schulz R, Miller GE, Helgeson VS. Is it beneficial to involve a family member? A meta-analysis of psychosocial interventions for chronic illness. *Health Psychol* 2004; 23(6):599-611
17. Bonanno, G. A., Galea, S., Bucciarelli, A., & Vlahov, D. (2007). What predicts psychological resilience after disaster? The role of demographics, resources, and life stress. *Journal of Consulting and Clinical Psychology*, *75*,671–682. http://dx.doi.org/10.1037/0022-006X.75.5.671
18. Brown KW, Ryan RM (2003) The benefits of being present: mindfulness and its role in psychological well-being.J Pers Soc Psychol 84(4):822
19. Donnellan C, Hevey D, Hickey A, O'Neil D (2006) Defining and quantifying coping strategies after stroke: a review. J Neurol Neurosurg Psychiatry 77:1208-1218
20. Southwick SM, Vythilingam M, Charney DS (2005) The psychobiology of depression and resilience to stress:implications for prevention and treatment. Annu Rev Clin Psychol 1:255-291
21. Korpershoek C, van der Bijl J, Hafsteinsdóttir TB (2011) Self-efficacy and its influence on recovery of patients with stroke: a systematic review. J Adv Nurs 67(9):1876-1894
22. Savini, S., Buck, H.G., Dickson, V.V., Simeone, S., Pucciarelli, G., Fida, R., ... Vellone, E. (2015). Quality of life in stroke survivor-caregiver dyads: a new conceptual framework and longitudinal study protocol. *Journal of Advanced Nursing, 71*(3), 676-687. PMID: 25186274
23. Barclay-Goddard, R., King, J., Dubouloz, C.J., Schwartz, C.E., & Response Shift Think Tank Working Group. (2012). Building on transformative learning and response shift theory to investigate health-related quality of life changes over time in individuals with chronic health conditions and disability. *Archives of Physical Medicine and Rehabilitation, 93*, 214-220. PMID: 22289229
24. Shields, C.G., King, D.A., & Wynne, L.C. (1995). Interventions with later life families. In R.H. Mikesell, D.D. Lustermann, & S.H. McDaniel (Eds.), *Integrating Family Therapy: Handbook of*

Family Psychology and Systems Theory (pp. 141-158). Washington, DC: American Psychological Association.

25. Tehan T., Zale E., Rosand J, Vranceanu AM. Perceptions of needs and recommendations for implementation of a resiliency intervention for patients with stroke and their families; Nurses speak. Accepted for presentation at Brain Health Conference, Columbus, OH April 28-30, 2017
26. http://www.who.int/mental_health/media/en/545.pdf. Accessed March 3rd, 2017.
27. Collins PY, Patel V, Joestl SS, March D, Insel TR, Daar A, on behalf of the Grand Challenges in Global Mental Health Scientific Advisory Board and Executive Committee. Grand Challenges in Global Mental Health. *Nature.* 2011 July 7. 474(7354):27-30. PMID 21734685
28. Edmondson, D., Richardson, S., Fausett, J.K., Falzon, L., Howard, V.J., Kronish, I.M. (2013). Prevalence of PTSD in survivors of stroke and transient ischemic attack: a meta-analytic review. *PLoS One, 8,* e66435. PMCID: PMC3686746
29. Ayerbe, L., Ayis, S., Wolfe, C.D., Rudd, A.G. (2013). Natural history, predictors and outcomes of depression after stroke: systematic review and meta-analysis. *The British Journal of Psychiatry, 202,* 14-21. PMID:23284148
30. Carod-Artal, F.J., Egido, J.A. (2009). Quality of life after stroke: the importance of a good recovery. *Cerebrovascular Diseases, 27,* 204-214. PMID: 19342853
31. Bartoli, F., Lillia, N., Lax, A., Crocamo, C., Mantero, V., Carrà, G., Agostoni, E. Clerici, M. (2013). Depression after stroke and risk of mortality: a systematic review and meta-analysis. *Stroke Research and Treatment, 2013,* 862978. PMCID: PMC3606772
32. Denno, M.S., Gillard, P.J., Graham, G.D., DiBonaventura, M.D., Goren, A., Varon, S.F., Zorowitz, R. (2013). Anxiety and depression associated with caregiver burden in caregivers of stroke survivors with spasticity. *Archives of Physical Medicine and Rehabilitation, 94,*1731-1736. PMID: 23548544
33. Bakas, T., Burgener, S.C. (2002). Predictors of emotional distress, general health, and caregiving outcomes in family caregivers of stroke survivors. *Topics in Stroke Rehabilitation, 9,* 34-45. PMID: 14523721
34. Monin, J., Doyle, M., Levy, B., Schulz, R., Fried, T., Kershaw, T. (2016). Spousal associations between frailty and depressive symptoms: longitudinal findings from the cardiovascular health study. *Journal of the American Geriatrics Society, 64,* 824-830. PMID: 27100578
35. Lee, S,, Colditz, G.A., Berkman, L.F., Kawachi, I. (2003). Caregiving and risk of coronary heart disease in US women: a prospective study. *American Journal of Preventative Medicine, 24,*113-119. PMID: 12568816
36. Ji, J., Zöller, B., Sundquist, K., Sundquist, J. (2012). Increased risks of coronary heart disease and stroke among spousal caregivers of cancer patients. *Circulation, 125,* 1742-1747. PMID: 22415143
37. Schulz, R., Beach, S.R. (1999). Caregiving as a risk factor for mortality: the Caregiver Health Effects Study. *JAMA, 282,* 2215-2219. PMID: 10605972
38. Beach, S.R., Schulz, R., Williamson, G.M., Miller, L.S., Weiner, M.F., Lance, C.E. (2005). Risk factors for potentially harmful informal caregiver behavior. *Journal of the American Geriatrics Society, 53,* 255-261. PMID:15673349
39. Turner-Stokes, L., Hassan, N. (2002). Depression after stroke: a review of the evidence base to inform the development of an integrated care pathway. Part 1: Diagnosis, frequency and impact. *Clinical Rehabilitation, 16,* 231-247. PMID: 12017511
40. Schubart, J.R., Kinzie, M.B., & Farace, E. (2008). Caring for the brain tumor patient: family caregiver burden and unmet needs. *Neuro-oncology, 10,* 61-72. PMCID: PMC2600839

41. Palmer, S., & Glass, T.A. (2003). Family function and stroke recovery: a review. *Rehabilitation Psychology, 48,* 255-265.
42. Perrin, P.B., Heesacker, M., Hinojosa, M.S., Uthe, C.E., & Rittman, M.R. (2009). Identifying at-risk, ethnically diverse stroke caregivers for counseling: a longitudinal study of mental health. *Rehabilitation Psychology, 54,* 138-149. PMID: 19469603
43. Bienvenu, O.J., Colantuoni, E., Mendez-Tellez, P.A., Dinglas, V.D., Shanholtz, C., Husain, N., ... Needham,
D.M. (2012). Depressive symptoms and impaired physical function after acute lung injury. *American Journal of Respiratory and Critical Care Medicine, 185,* 517-524. PMCID: PMC3297105
44. Martin, L.R., Williams, S.L., Haskard, K.B., & DiMatteo, M.R. (2005). The challenge of patient adherence. *Therapeutics and Clinical Risk Management, 1,* 189-199. PMCID: PMC1661624
45. Rees, J., O'Boyle, C., & MacDonagh, R. (2001). Quality of life: impact of chronic illness on the partner. *Journal of the Royal Society of Medicine, 94,* 563-566. PMCID: PMC1282240
46. Azoulay, E., Pochard, F., Kentish-Barnes, N., Chevret, S., Aboab, J., Adrie, C., ... FAMIREA Study Group. (2005). Risk of post-traumatic stress symptoms in family members of intensive care unit patients. *American Journal of Respiratory and Critical Care Medicine, 171,* 987-994. PMID: 15665319
47. Jones, C., Skirrow, P., Griffiths, R.D., Humphris, G., Ingleby, S., Eddleston, J., ... Gager, M. (2004). Posttraumatic stress disorder-related symptoms in relatives of patients following intensive care. *Intensive Care Medicine, 30,* 456-460. PMID: 14767589
48. Jones, C., & Griffiths, R.D. (2007). Patient and caregiver counselling after the intensive care unit: what are the needs and how should they be met? *Current Opinion in Critical Care, 13,* 503-507. PMID: 17762226
49. Im, K., Belle, S.H., Shulz, R., Mendelsohn, A.B., Chelluri, L., & QOL-MV Investigators. (2004). Prevalence and outcomes of caregiving after prolonged (> or =48 hours) mechanical ventilation in the ICU. *Chest, 125,* 597-606. PMID: 14769744
50. Douglas, S.L., Daly, B.J., Kelley, C.G., O'Toole, E., & Montenegro, H. (2005). Impact of a disease management
program upon caregivers of chronically ill patients. *Chest, 128,* 3925-3936. PMID: 16354865
51. Martire LM, Lustig AP, Schulz R, Miller GE, Helgeson VS. Is it beneficial to involve a family member? A metaanalysis of psychosocial interventions for chronic illness. *Health Psychol* 2004; 23(6):599-611
52. Bakas, T., Clark, P.C., Kelly-Hayes, M., King, R.B., Lutz, B.J., Miller, E.L., & American Heart Association Council on Cardiovascular and Stroke Nursing and the Stroke Council. (2014). Evidence for stroke family caregiver and dyad interventions: a statement for healthcare professionals from the American Heart Association and American Stroke Association. *Stroke, 45,* 2836-2852. PMID: 25034718
53. Barclay-Goddard, R., King, J., Dubouloz, C.J., Schwartz, C.E., & Response Shift Think Tank Working Group. (2012). Building on transformative learning and response shift theory to investigate health-related quality of life changes over time in individuals with chronic health conditions and disability. *Archives of Physical Medicine and Rehabilitation, 93,* 214-220. PMID: 22289229
54. Cook, W.L. & Kenny, D.A. (2005). The Actor-Partner Interdependence Model: A model of bidirectional effects in developmental studies. *Journal of Behavioral Development, 29,* 102-109.
55. Zale EL, McCurley JL, Lin A, Funes C, Tehan T, Henhuis T, Rosand J., Vranceanu, A.M. Early psychological distress is crossectionally and prospectively interdependent between patients

admitted to the Neuro-ICU and their family caregivers. Submitted for presentation at Society of Behavioral Medicine, New Orleans, April 28-30, 2018.

56. Richards, K.C., Enderlin, C.A., Beck, C., McSweeney, J.C., Jones, T.C., & Roberson, P.K. (2007). Tailored biobehavioral interventions: a literature review and synthesis. *Research and Theory for Nursing Practices, 21,* 271-285. PMID: 18236771
57. Vranceanu AM, Ricklin E, Merker V, Park E, Plotkin SR. (2016) Mind body therapy for patients with
neurofibromatosis via live video; An RCT. Neurology 87 (8):806-14.
58. Zale EL, Piere-Louis C, Riklin E, Macklin E, Vranceanu AM. The impact of a mind body program on multiple dimensions of resiliency in geographically diverse patients with neurofibromatosis. JCCP accepted.
59. Bellg AJ, Borrelli B, Resnick B, et al. (2004). Enhancing treatment fidelity in health behavior change studies: Best practices and recommendations from the NIH Behavior Change Consortium. *Health Psychol.* 23:443-451. PMID:15367063.
60. Vranceanu AM**,** Merker VL, Plotkin SR, Park ER. The Relaxation Response Resiliency Program (3RP) in patients with neurofibromatosis 1, neurofibromatosis 2, and schwannomatosis: results from a pilot study. J Neurooncol. 2014; 120(1): 103-109.
61. Rounsavile, B.J., Carroll, K.M., & Onken, L.S. (2001). A stage model of behavioral therapies research: getting started and moving on from stage I. *Clinical Psychology: Science and Practice, 8*, 133-142.
62. Larsen, D.L., Attkisson, C.C., Hargreaves, W.A., & Nguyen, T.D. (1979). Assessment of client/patient satisfaction: development of a general scale. *Evaluation and Program Planning, 2,* 197-207. PMID: 10245370
63. Devilly, G.J., & Borkovec, T.D. (2000). Psychometric properties of the credibility/expectancy questionnaire. *Journal of Behavior Therapy and Experimental Psychiatry, 31,* 73-86. PMID: 11132119
64. Zigmond, A.S. & Snaith, R.P. (1983). The hospital anxiety and depression scale. *Acta Psychiatrica Scandinavica, 67,* 361-370. PMID: 6880820
65. Bhandari, N.J., Jain, T., Marolda, C., & ZuWallack, R.L. (2013). Comprehensive pulmonary rehabilitation results in clinically meaningful improvements in anxiety and depression in patients with chronic obstructive pulmonary disease. *Journal of Cardiopulmonary Rehabilitation and Prevention, 33,* 123-127. PMID: 23399845
66. Blanchard, E.B., Jones-Alexander, J., Buckley, T.C., & Forneris, C.A. (1996). Psychometric properties of the PTSD Checklist (PCL). *Behaviour Research and Therapy, 34,* 669-673. PMID: 8870294
67. Monson, C.M., Gradus, J.L., Young-Xu, Y., Schnurr, P.P., Price, J.L., & Schumm, J.A. (2008). Change in posttraumatic stress disorder symptoms: do clinicians and patients agree? *Psychological Assessment, 20,* 131-138. PMID: 18557690
68. Feldman, G., Hates, A., Kumar, S., Greeson, J., & Laurenceau, J.P. (2007). Mindfulness and emotion regulation: The development and initial validation of the Cognitive and Affective Mindfulness Scale Revised (CAMS-R). *Journal of Psychopathology and Behavioral Assessment, 29*, 177-190.
69. Schwarzer, R. & Jerusalem, M. (1995). Generalized Self-Efficacy scale. In J. Weinman, S. Wright, & M. Johnston (Eds.), *Measures in health psychology: A user's portfolio. Causal and control beliefs* (pp. 35-37). Windsor, UK: NFER-NELSON.

70. Cohen, S., Mermelstein, R., Kamarck, T., & Hoberman, H.M. (1985). Measuring the functional components of social support. In I.G. Sarason & B.R. Sarason (Eds.), *Social Support: Theory, Research and Applications* (pp.73-94). Netherlands: Springer.
71. Carver CS. (2006). Measure of Current Status. http://www.psy.miami.edu/faculty/ccarver/sclMOCS.html
72. Merz, EL, Roesch, SC, Malcarne, VL, Penedo, FJ, Llabre, MM, Weitzman, OB, ... Johnson, TP. (2013). Validation of interpersonal support evaluation list-12 (ISEL-12) scores among English- and Spanish-speaking Hispanics/Latinos from the HCHS/SOL sociocultural ancillary study. *Psychological Assessment* 26(2): 384-394.
73. Wilhelm, K. & Parker, G. (1988). The development of a measure of intimate bonds. *Psychological Medicine*, *18*, 225-234. PMID: 3363041
74. Guest, G., Bunce, A., & Johnson, L. (2006). How many interviews are enough? An experiment with data saturation and variability. *Field Methods, 18*(1), 24. doi: 10.1177/1525822X05279903
75. Browne RH. On the use of a pilot sample for sample size determination. *Stat Med*.1995;14: 1933-40. PMID:8532986.
76. Lancaster GA, Dodd S, Williamson PR. (2004). Design and analysis of pilot studies: recommendations for good practice. *J Eval Clin Pract*.;10:307-12. PMID: 15189396.
77. Rounsaville BJ, Caroll KM, Onken LS. (2001)A stage model of behavioral therapies research: getting started and moving on from stage I. *Clin Psychol Sci Pract*. 8:133–142
78. Shih WJ, Ohman-Strickland PA, Lin Y. (2004) Analysis of pilot and early phase studies with small sample sizes. *Stat Med.* 23:1827–1842 PMID: 15195318.
79. Whitehead AL, Julious SA, Cooper CL, Campbell MJ (2016). Estimating the sample size for a pilot randomized trial to minimise the overall trial sample size for the external pilot and main trial for a continuous outcome variable. *Stat Methods Med Res* 25:1057-1073. PMID: 26092476.
80. Miles, H.B., Huberman, A.M.(1994). *Qualitative Data Analysis.* Sage Publication, Thousand Oaks California.
81. Bowen DJ, Kreuter M, Spring B, et al. (2009) How we design feasibility studies. *Am J Prev Med.* May;36(5):452-7. PMID: 19362699.
82. Schafer JL, Graham JW. (2002). Missing data: our view of the state of the art. *Psychol Methods* 7(2):147-77. PMID: 12090408.
83. National Alliance for Caregiving (NAC). (2015). *Caregiving in the U.S. 2015.* Bethesda, MD: AARP Public Policy Institute.
84. Ruskin, P.E., Silver-Aylaian, M., Kling, M.A., Reed, S.A., Bradham, D.D., Hebel, J.R., ... Hauser, P. (2004). Treatment outcomes in depression: comparison of remote treatment through telepsychiatry to in-person treatment. *The American Journal of Psychiatry, 161,* 1471-1476. PMID: 15285975
85. Carlbring, P., & Andersson, G. (2006). Internet and psychological treatment. How well can they be combined? *Computers in Human Behavior, 22,* 545-553.
86. Clough, B.A., & Casey, L.M. (2011). Technological adjuncts to increase adherence to therapy: a review. *Clinical Psychology Review, 31,* 697-710. PMID: 21497153
87. Lin A, Jacobo M, Jacobs J, Tehan T, Salgueiro D, Rosand J, Vranceanu AM, Zale E. Gender differences in emotional distress among caregivers of patients admitted to the Neuroscience-Intensive Care. Submitted to the Society of Behavioral Medicine Annual Meeting, New Orleans, April 2018
88. Blake H, McKinney K, Treece E, Lee NB. (2002) An evaluation of screening measures for cognitive functioning after stroke. Age and Ageing, 31, pp.451-456.

PARTNERS HUMAN RESEARCH COMMITTEE
PROTOCOL SUMMARY

Answer all questions accurately and completely in order to provide the PHRC with the relevant information to assess the risk-benefit ratio for the study. Do not leave sections blank.

PRINCIPAL/OVERALL INVESTIGATOR
Ana-Maria Vranceanu, Ph.D.

PROTOCOL TITLE
Recovering Together: Building resiliency in dyads in patients admitted to the Neuroscience Intensive Care Unit (NICU) and their caregivers

FUNDING
National Institute of Nursing Research

VERSION DATE
October 31, 2018

SPECIFIC AIMS
Concisely state the objectives of the study and the hypothesis being tested.

The current study has the following specific aims:

1) To determine the feasibility of recruitment, feasibility of program delivery, program credibility, and program satisfaction using evidence-based benchmarks.

   Hypothesis 1: We hypothesize that > 75% of the dyads approached will agree to participate.
   Hypothesis 2: We hypothesize that > 75% of dyads who start the intervention will complete at least 4 sessions.
   Hypothesis 3: We hypothesize that > 75% of participants will report average credibility (Credibility and Expectancy Questionnaire) scores greater than the scale's midpoint.
   Hypothesis 4: We hypothesize that > 75% participants will report average satisfaction (Client Satisfaction Scale) scores greater than the scale's midpoint.

2) To demonstrate a proof of concept that the Recovering Together program can sustainably improve emotional distress [Hospital Anxiety and Depression Scale; HADS], Post-Traumatic Symptoms (PTS) [PCL-S], resiliency variables (mindfulness, coping, social support and self-efficacy) and interpersonal factors (interpersonal bond).

   Hypothesis 1: We hypothesize that participation in the Recovering Together Program will be associated with a more potent decrease in emotional distress and PTS compared to participation in the educational program (control), and that these improvements will maintain at 3 month follow up.
   Hypothesis 2: We hypothesize that Recovering Together Program will be associated with a more potent increase in resiliency variables (mindfulness, coping, social support, self-efficacy) and interpersonal factors (interpersonal bond) compared to participation in the educational program, and that these improvements will maintain at 3 month follow up.

BACKGROUND AND SIGNIFICANCE

Provide a brief paragraph summarizing prior experience important for understanding the proposed study and procedures.

Acute neurological illnesses (ANIs) are common, costly and often lead to long-term disability. ANIs are biologically distinct injuries that disrupt the normal function of the brain. The most common ANIs in Neuroscience Intensive Care Units (NICU) include cerebrovascular (stroke/hemorrhage and brain aneurysm), structural (tumors and lesions/brain masses), and traumatic (TBI) brain injuries. NICU admissions for ANIs are prevalent (e.g., 795,000 acute stroke/year; 275,000 acute TBI/year) and costly; post NICU prolonged rehabilitation is common.
ANIs are associated with chronic emotional distress in both patients (pts) and caregivers (cgs). Although biologically heterogeneous, ANIs are unified by sudden onset, and substantial emotional distress in both pts (e.g., 12-43% anxiety; 10-58% depression; 20-29% post-traumatic stress PTS) and family cgs (27-60% depression, anxiety or PTS). These symptoms often become chronic and treatment resistant.
Pt and cg factors interact and influence physical and emotional outcomes in both pts and cgs. Post ANI emotional distress is associated with pts' poor medical adherence, slower recovery, higher mortality, and need of more caregiving assistance, which further increase cgs' distress and own risk for morbidity and mortality; in turn cgs' emotional distress interferes with ability to provide high-quality care to pts and negatively impacts pts' outcomes.
Current management of ANIs does not meet the psychological needs of pts and cgs for 3 reasons. First, although recognition of the emotional burden associated with NICU admission has increased, and some NICUs have social workers available to assist pts and cgs, there are no formal screening methods for emotional distress routinely integrated in practice during hospitalization, when the primary focus is on medical care and survival; further, there are no formal evidence-based treatments integrated within the medical care. When social workers are included to help pts and cgs, the care is brief and occurs only during hospitalization. When referrals to mental health services are provided to families at discharge, few will access additional treatment due to burden associated with traveling outside of home. Second, psychosocial interventions available for ANI pts or cgs are limited in that they are delivered when symptoms are already chronic, address only one emotional illness (e.g., depression or anxiety or PTS), and/or are focused on a *single member* of the pt-cg dyad. Even interventions labeled as "dyadic," which include pts and cgs, typically address only the pts' needs and do not focus on cg outcomes or on the dyad's interpersonal communication and bond/relationship. These interventions are not consistent with the *dyadic framework* which specifies that dyadic interventions should account for the interdependence between pt and cg psychosocial factors including their interpersonal bond by ensuring that both pts and cgs attend each session together, and by targeting improvement in outcomes for both pts and cgs. Third, most interventions are delivered using uniform protocols. However, the needs of ANI dyads are heterogeneous due to varying levels of post ANI impairment, identity of the cg, context and stage of life. A recent systematic review urged for the development of dyadic interventions that address the needs of *both* pts and cgs and are tailored to the specific needs of each ANI dyad.
We developed the first dyadic skills-based intervention – Recovering Together - to prevent chronic heightened emotional distress in at risk ANI pt-cg dyads. The "Recovering

Together” program is informed by the theoretical response-shift framework of adaptation to acute illness (successful adaptation implies recalibration of values and life goals), the family strength vulnerability model (within dyads relational systems have strengths and weaknesses in how they cope with life events), the dyadic longitudinal model (distress travel from one member of the dyad to the other across time), the APIM model, and the resiliency framework**.** The program is in line with recent recommendations for skill-based interventions for critical care patients, and uses preliminary data collected by our team for the past 3 years. The intervention teaches pts and cgs resiliency factors that are associated with well-being after trauma for both pts and cgs: *mindfulness* – the ability to stay present and defer judgment in the face of adversity; *coping*– the arsenal and application of one’s behavioral, cognitive, and emotional strategies to manage stress; *social support* –empathetic interpersonal interactions that meet one’s emotional and functional needs; *self-efficacy* – perceived ability to adapt under adversity and *positive dyadic interpersonal communication to increase interpersonal bond*. Informed by the aforementioned theoretical models, our conceptual model hypothesizes that by teaching both members of the dyad resiliency and interpersonal communication skills (e.g., Recovering Together) we will be able to sustainably decrease emotional distress in both members of the dyad.

RESEARCH DESIGN AND METHODS

Briefly describe study design and anticipated enrollment, i.e., number of subjects to be enrolled by researchers study-wide and by Partners researchers. Provide a brief summary of the eligibility criteria (for example, age range, gender, medical condition). Include any local site restrictions, for example, “Enrollment at Partners will be limited to adults although the sponsor’s protocol is open to both children and adults.”

The current study will be a two arm, feasibility pilot RCT.

We will randomize a total of 80 dyads (40 dyads in each arm) to either a psychosocial intervention or to an educational program in the Neuro ICU.

Eligible dyads (pts and cgs) must meet the following inclusion criteria:

1) Age 18 or older
2) English fluency and literacy
3) Access to high speed internet for video sessions
4) Pt with an informal cg (family or friend who provides unpaid care) available and willing to participate
5) Hospitalized with an ANI within 1-2 weeks (pt) OR primary cg of a pt currently admitted with an ANI
6) Either pt <u>or</u> cg have clinically significant symptoms of depression, anxiety, and/or PTS

One or more of the following exclusion criteria will render a pt ineligible:

1) Permanent or severe cognitive impairment severe enough to impede participation – This will be determined by nurses through an assessment conducted as part of usual care and that includes the MMSE (score of <23) and GCS (score of <10). Nurses are trained and use these measures as part of NICU care.

2) Dyads where the pt is anticipated to die or to never be able to participate due to medical sequelae. This will be determined by nurses.

   This exclusionary criterion is already used in our IRB approved prospective study with this population.

Briefly describe study procedures. Include any local site restrictions, for example, "Subjects enrolled at Partners will not participate in the pharmacokinetic portion of the study." Describe study endpoints.

Study procedures:

Participants will be referred to the study by the nursing team who will ensure that patients are medically and cognitively able to participate (see above). Eligible dyads will next be screened, consented, and enrolled by the research assistant.

After enrollment, subjects will be randomized to either the newly developed psychosocial intervention or to the educational program (control). Dyads will be randomly assigned by using a random number sequence generator to ensure comparability between groups. Randomization will be developed by the statistician, without any input from the rest of the team. All subjects will be given baseline psychological and behavioral assessments that will assess depression, anxiety, PTSD symptoms, and other psychological constructs. All assessments will be administered via computer using the REDCap secure data collection system.

In the skills-based intervention group, sessions focus on developing skills to cope and manage ANI related stressors. The intervention will be tailored consistent with AHA recommendations for ANI skills-based interventions and will include 2 general and 4 specific modules. It is anticipated that the intervention will have 6 sessions with 2 general sessions delivered, in person if possible, within the NICU, or through live video using Vidyo if patients leave the hospital before sessions occur, and 4 tailored specific sessions (chosen from by the dyads from 6 available modules) to be delivered via live video using Vidyo.

Those in the educational program will receive general health information that mimics the Recovering Together Program, but without teaching any of the resiliency or interpersonal communication skills that are hypothesized to be responsible for improvement in emotional distress. There will also be 6 sessions, 2 in-person dyadic visits in the NICU and 4 dyadic virtual visits following discharge. The educational program group will not have the opportunity to specify which modules they would like to take; the modules will be predetermined. All participants will receive medical care as determined by their medical team.

Both groups will continue with their current care with addition to the educational programs provided.

Both groups will complete post-treatment psychological and behavioral assessments (identical to those administered at baseline) to be administered immediately after completion of the course and again at three months after completion of the course in order to measure long term outcomes. They will also be asked questions about the perceived efficacy of the intervention.

Study Endpoints:

1) Is the psychosocial skills-based intervention feasible, usable, and accepted by pts and cgs in the ICU?

2) Is the intervention associated with improvement in depression, anxiety and PTSD?

3) Is the effect of the skills-based intervention for pts and cgs in the ICU durable at 3 month follow up?

We will assess feasibility, usability, and acceptability by the enrolment numbers, participant completion in at least 4 out of 6 sessions, and the questionnaires, the Credibility and Expectancy and Client Satisfaction Scale. These will be the primary outcomes. The PCL-S, HADS, and the resiliency measures will serve as the secondary outcomes.

For studies involving treatment or diagnosis, provide information about standard of care at Partners (e.g., BWH, MGH) and indicate how the study procedures differ from standard care. Provide information on available alternative treatments, procedures, or methods of diagnosis.

NA

Describe how risks to subjects are minimized, for example, by using procedures which are consistent with sound research design and which do not unnecessarily expose subjects to risk or by using procedures already being performed on the subject for diagnostic or treatment purposes.

All study staff will complete required Partners human subjects trainings prior to the start of study procedures. In order to preserve confidentiality of participants, study data will not be linked to any identifying information; rather, study ID numbers will be assigned and used to identify participants. All study forms will be stored in locked storage spaces, to which only study staff will have access. All interventionists and assessors will have advanced training in clinical interviewing and assessment. Participants will be informed that they may refuse to answer questions that make them feel uncomfortable.

Describe explicitly the methods for ensuring the safety of subjects. Provide objective criteria for removing a subject from the study, for example, objective criteria for worsening disease/lack of improvement and/or unacceptable adverse events. The inclusion of objective drop criteria is especially important in studies designed with placebo control groups.

There is no risk of physical injury to participants. If a participant is judged to be suicidal at any time during participation, the interventionist will refer him or her to appropriate services, including the Acute Psychiatry Service at MGH if deemed appropriate.

FORESEEABLE RISKS AND DISCOMFORTS

Provide a brief description of any foreseeable risks and discomforts to subjects. Include those related to drugs/devices/procedures being studied and/or administered/performed solely for research purposes. In addition, include psychosocial risks, and risks related to privacy and confidentiality. When applicable, describe risks to a developing fetus or nursing infant.

Participants may feel uncomfortable completing various psychological questionnaires. As in any research study, there is a small risk that confidentiality may be breached; all efforts to minimize this risk will be taken, as outlined above. In the unlikely event that participants will become suicidal during the duration of the study, the research assistant will contact the PI and appropriate clinical intervention will be executed.

EXPECTED BENEFITS

Describe both the expected benefits to individual subjects participating in the research and the importance of the knowledge that may reasonably be expected to result from the study. Provide a brief, realistic summary of potential benefits to subjects, for example, “It is hoped that the treatment will result in a partial reduction in tumor size in at least 25% of the enrolled subjects.” Indicate how the results of the study will benefit future patients with the disease/condition being studied and/or society, e.g., through increased knowledge of human physiology or behavior, improved safety, or technological advances.

Participants may not benefit from the study directly. However, knowledge from this research study may benefit others by enhancing our understanding of the role of psychosocial skills-based interventions in treating future pts and cgs within the NICU. All participants will receive $20 for the completion of each of the 3 assessment points.

EQUITABLE SELECTION OF SUBJECTS

The risks and benefits of the research must be fairly distributed among the populations that stand to benefit from it. No group of persons, for example, men, women, pregnant women, children, and minorities, should be categorically excluded from the research without a good scientific or ethical reason to do so. Please provide the basis for concluding that the study population is representative of the population that stands to potentially benefit from this research.

All subjects who satisfy the inclusion/exclusion criteria are eligible for enrollment in this study regardless of sex, race or ethnicity.

When people who do not speak English are excluded from participation in the research, provide the scientific rationale for doing so. Individuals who do not speak English should not be denied participation in research simply because it is inconvenient to translate the consent form in different languages and to have an interpreter present.

Only participants who can read and speak English will be included in the current study as not all assessment measures have been validated for use in non-English speaking populations.

For guidance, refer to the following Partners policy:

Obtaining and Documenting Informed Consent of Subjects who do not Speak English
https://partnershealthcare-public.sharepoint.com/ClinicalResearch/Non-English_Speaking_Subjects.1.10.pdf

RECRUITMENT PROCEDURES

Explain in detail the specific methodology that will be used to recruit subjects. Specifically address how, when, where and by whom subjects will be identified and approached about participation. Include any specific recruitment methods used to enhance recruitment of women and minorities.

Potential participants will be referred for study participation by their medical teams (nurses). The medical team will assess whether the ANI patients are able to consent, consistent with medical presentation. Trained study staff will be administering the MMSE and nurses will be administering the GSC to all patients and will refer only dyads where the patients' scores are higher than the established cut off scores on these measures. Pts and their respective cgs who are able to consent and who express interest in the study will speak with a study team member to learn more about the study and be screened for eligibility; those who wish to participate will complete consent. If cgs cannot be reached in person in the hospital, they will be contacted via phone for screening and completed of informed consent. Cgs will be given a copy of the consent form to review while discussing the study over the telephone. We will fax or securely e-mail the consent form prior to obtaining consent. Cgs will return a signed copy of the consent form.

Patients and caregivers will be considered enrolled when they sign the consent form with the study staff. These procedures will be completed in a private medical space.

Participants will be explicitly informed that this intervention is a research study that does not constitute individualized, personal care. The intervention is a broad-based method of training that is not tailored to any particular individual. Should any participant seek formal mental healthcare, study staff will refer them to either MGH Psychiatry, as appropriate.

All dyads will be recruited within 1-2 weeks of pt's hospitalization. The pt's medical team will also alert the research assistant whether they anticipate that the patient might be able to participate in the study at a future point during the hospital stay. In situations where patients are unable to consent due to the severity of the stroke, we will enroll the informal cg and return to enroll the cg as soon as their mental capacity improves. The research assistant will not approach patients who are not mentally or physically capable to participate.

Eligible cases may also be identified by daily screening of Epic admission reports.

We will not include patients who do not have a caregiver. Informal caregivers will be designated by the health care proxy and verbally confirmed by the patient. Nurses will assist the study team with identifying the patient's health care proxy.

Provide details of remuneration, when applicable. Even when subjects may derive medical benefit from participation, it is often the case that extra hospital visits, meals at the hospital, parking fees or other inconveniences will result in additional out-of-pocket expenses related to

study participation. Investigators may wish to consider providing reimbursement for such expenses when funding is available

All participants will receive $20 compensation for completing each of the 3 assessment points.

For guidance, refer to the following Partners policies:

Recruitment of Research Subjects
https://partnershealthcare-public.sharepoint.com/ClinicalResearch/Recruitment_Of_Research_Subjects.pdf

Guidelines for Advertisements for Recruiting Subjects
https://partnershealthcare-public.sharepoint.com/ClinicalResearch/Guidelines_For_Advertisements.1.11.pdf

Remuneration for Research Subjects
https://partnershealthcare-public.sharepoint.com/ClinicalResearch/Remuneration_for_Research_Subjects.pdf

CONSENT PROCEDURES

Explain in detail how, when, where, and by whom consent is obtained, and the timing of consent (i.e., how long subjects will be given to consider participation). For most studies involving more than minimal risk and all studies involving investigational drugs/devices, a licensed physician investigator must obtain informed consent. When subjects are to be enrolled from among the investigators' own patients, describe how the potential for coercion will be avoided.

After determining eligibility study staff will meet with potential dyads to review the informed consent document. After the document has been reviewed, study staff will answer any and all questions the pt or cg may have. Once all questions have been addressed, each member of the dyad will sign informed consent form, which will include a description of all study procedures, information about potential risks and benefits of participation, the option to receive text message reminders and study contact information (including that of the IRB) in case questions arise at a later time. The consent form will also explicitly state that study participation is voluntary, and that participants may refuse to answer any questions that make them uncomfortable, and may discontinue participation at any time. In addition, participants will be assured that withdrawal from the study will not compromise their medical care in any way.

As informed consent is a continuous process, participants will be given a copy of the signed informed consent document, and will be invited to ask questions about their participation at any point over the course of the study.

NOTE: When subjects are unable to give consent due to age (minors) or impaired decision-making capacity, complete the forms for Research Involving Children as Subjects of Research and/or Research Involving Individuals with Impaired Decision-making Capacity, available on the New Submissions page on the PHRC website:
https://partnershealthcare.sharepoint.com/sites/phrmApply/aieipa/irb

For guidance, refer to the following Partners policy:
Informed Consent of Research Subjects:
https://partnershealthcare-public.sharepoint.com/ClinicalResearch/Informed_Consent_of_Research_Subjects.pdf

DATA AND SAFETY MONITORING

Describe the plan for monitoring the data to ensure the safety of subjects. The plan should include a brief description of (1) the safety and/or efficacy data that will be reviewed; (2) the planned frequency of review; and (3) who will be responsible for this review and for determining whether the research should be altered or stopped. Include a brief description of any stopping rules for the study, when appropriate. Depending upon the risk, size and complexity of the study, the investigator, an expert group, an independent Data and Safety Monitoring Board (DSMB) or others might be assigned primary responsibility for this monitoring activity.

NOTE: Regardless of data and safety monitoring plans by the sponsor or others, the principal investigator is ultimately responsible for protecting the rights, safety, and welfare of subjects under his/her care.

Risks to participants are minimal. In the unlikely event that a participant is determined to be actively suicidal and at risk for self-harm during any study procedures, the research assistant will contact the PI (Vranceanu) and appropriate clinical intervention will be executed. Dr. Vranceanu may start a psychiatric consult depending on the severity of the situation. All study staff have been trained in responsible research conduct through a CITI course at MGH. The research assistant has also been trained on the importance of maintaining confidentiality, and the assignment of ID numbers. All data will be kept confidential, under lock-and-key, accessible only to trained study staff. Participants' data will be identified by ID number only, and a link between names and ID numbers will be kept separately under lock and key.

Describe the plan to be followed by the Principal Investigator/study staff for review of adverse events experienced by subjects under his/her care, and when applicable, for review of sponsor safety reports and DSMB reports. Describe the plan for reporting adverse events to the sponsor and the Partners' IRB and, when applicable, for submitting sponsor safety reports and DSMB reports to the Partners' IRBs. When the investigator is also the sponsor of the IND/IDE, include the plan for reporting of adverse events to the FDA and, when applicable, to investigators at other sites.

NOTE: In addition to the adverse event reporting requirements of the sponsor, the principal investigator must follow the Partners Human Research Committee guidelines for Adverse Event Reporting

Adverse events may be discovered in the event that a patient spontaneously reports an adverse event, or an adverse event is discovered during the assessment process. All adverse events will be reported by the PI to the Office of Research Compliance within 24 hours.

MONITORING AND QUALITY ASSURANCE

Describe the plan to be followed by the principal investigator/study staff to monitor and assure the validity and integrity of the data and adherence to the IRB-approved protocol. Specify who will be responsible for monitoring, and the planned frequency of monitoring. For example, specify who will review the accuracy and completeness of case report form entries, source documents, and informed consent.

NOTE: Regardless of monitoring plans by the sponsor or others, the principal investigator is ultimately responsible for ensuring that the study is conducted at his/her investigative site in accordance with the IRB-approved protocol, and applicable regulations and requirements of the IRB.

Once completed, a member of study staff will verify that all items on all questionnaires have been addressed. Data will be checked for out of range values using frequency distributions prior to analyzing the data. The Principal Investigator will be responsible for ensuring compliance with IRB procedures.

For guidance, refer to the following Partners policies:

Data and Safety Monitoring Plans and Quality Assurance
https://partnershealthcare-public.sharepoint.com/ClinicalResearch/DSMP_in_Human_Subjects_Research.pdf

Reporting Unanticipated Problems (including Adverse Events)
https://partnershealthcare-public.sharepoint.com/ClinicalResearch/Reporting_Unanticipated_Problems_including_Adverse_Events.pdf

PRIVACY AND CONFIDENTIALITY

Describe methods used to protect the privacy of subjects and maintain confidentiality of data collected. This typically includes such practices as substituting codes for names and/or medical record numbers; removing face sheets or other identifiers from completed surveys/questionnaires; proper disposal of printed computer data; limited access to study data; use of password-protected computer databases; training for research staff on the importance of confidentiality of data, and storing research records in a secure location.

NOTE: Additional measures, such as obtaining a Certificate of Confidentiality, should be considered and are strongly encouraged when the research involves the collection of sensitive data, such as sexual, criminal or illegal behaviors.

As noted above, study data will not be linked to any identifying information; rather, study ID numbers will be assigned and used to identify participants. All study forms will be stored in locked storage spaces, to which only study staff will have access. All study staff will complete required Partners human subjects trainings prior to the start of study procedures.

SENDING SPECIMENS/DATA TO RESEARCH COLLABORATORS OUTSIDE PARTNERS

Specimens or data collected by Partners investigators will be sent to research collaborators outside Partners, indicate to whom specimens/data will be sent, what information will be sent, and whether the specimens/data will contain identifiers that could be used by the outside collaborators to link the specimens/data to individual subjects.

Data collected from the current study will not be sent to research collaborators outside of Partners.

Specifically address whether specimens/data will be stored at collaborating sites outside Partners for future use not described in the protocol. Include whether subjects can withdraw their specimens/data, and how they would do so. When appropriate, submit documentation of IRB approval from the recipient institution.

Specimens / data will not be stored at collaborating sites outside of Partners for future use not described in this protocol.

RECEIVING SPECIMENS/DATA FROM RESEARCH COLLABORATORS OUTSIDE PARTNERS

When specimens or data collected by research collaborators outside Partners will be sent to Partners investigators, indicate from where the specimens/data will be obtained and whether the specimens/data will contain identifiers that could be used by Partners investigators to link the specimens/data to individual subjects. When appropriate, submit documentation of IRB approval and a copy of the IRB-approved consent form from the institution where the specimens/data were collected.

Specimens and data will not be collected by research collaborators outside of Partners.

Partners Human Research
Partners HealthCare
399 Revolution Drive, Suite 710
Somerville, MA 02145
Tel: 857-282-1900
Fax: 857-282-5693

Notification of IRB Review

Protocol #: 2018P002187

Date:	February 25, 2019
To:	Vranceanu, Ana-Maria,<br>MGH<br>Partners > MGH > Psychiatry
From:	Partners Human Research<br>399 Revolution Drive, Suite 710<br>Somerville, MA 02145
Title of Protocol:	Recovering Together: Building resiliency in dyads in patients admitted to the Neuroscience Intensive Care Unit (NICU) and their caregivers
Version/Number:	1
Version Date:	09/24/2018
Sponsor/Funding/Support:	Proposal Title: Recovering together: Building resiliency in dyads of patients admitted to the Neuroscience Intensive Care Unit (NICU) and their caregivers<br>Principal Investigator: Vranceanu, Ana-Maria<br>Immediate Sponsor: NIH<br>A# ard Number: 1R21NR017979-01A1<br>Fund w. 233585

IRB Amendment w.	8
IRB Revie# Type:	Expedited
IRB Approval Date:	02/25/2019
Approval/Activation Date:	02/25/2019
**IRB Expiration Date:**	**10/26/2019**

This project has been revie# ed and approved by the **PHS IRB**. During the revie# of this project, the IRB specifically considered (i) the risks and anticipated benefits, if any, to subjects; (ii) the selection of subjects; (iii) the procedures for obtaining and documenting informed consent; (iv) the safety of subjects; and (v) the privacy of subjects and confidentiality of the data.

Please note that if an IRB member had a conflict of interest # ith regard to the revie# of this project, consistent # ith IRB policies and procedures, the member # as required to recuse him/herself and, if applicable, leave the room during the discussion and vote on this project except to provide information requested by the IRB.

Partners Human Research
Partners HealthCare
399 Revolution Drive, Suite 710
Somerville, MA 02145
Tel: 857-282-1900
Fax: 857-282-5693

GENERAL REVIEW COMMENTS

This approval covers an amendment to use a shorter client satisfaction questionnaire, the CSz -3 and removing the CSz -8; and adding to the detailed protocol that this measure # ill be sent to both the intervention and control groups.

As Principal Investigator, you are responsible for ensuring that this project is conducted in compliance # ith all applicable federal, state and local la# s and regulations, institutional policies, and requirements of the IRB, # hich include, but are not limited to, the follo# ing:

1. Submission of any and all proposed changes to this project (e.g., protocol, recruitment materials, consent form, status of the study, etc.) to the IRB for revie# and approval prior to initiation of the change(s), except # here necessary to eliminate apparent immediate ha' ards to the subject(s). Changes made to eliminate apparent immediate ha' ards to subjects must be reported to the IRB as an unanticipated problem.
2. Submission of a continuing revie# submission or institutional status report as required by the IRB and/or institution to continue the research, and submission of a final report # hen the project has been closed or completed.
3. Submission of any and all unanticipated problems, including adverse event(s) in accordance # ith the IRBWs policy on reporting unanticipated problems including adverse events.
4. Obtaining informed consent from subjects or their legally authori' ed representative prior to initiation of research procedures # hen and as required by the IRB and, # hen applicable, documenting informed consent current IRB approved consent form(s) # ith the IRB-approval stamp in the document footer.
5. Informing all investigators and study staff listed on the project of changes and unanticipated problems, including adverse events, involving risks to subjects or others.
6. Q hen investigator financial disclosure forms are required, submitting updated financial disclosure forms for yourself and for informing all site responsible investigators, co-investigators and any other members of the study staff identified by you as being responsible for the design, conduct, or reporting of this research study of their obligation to submit updated Investigator Financial Disclosure Forms for this protocol to the IRB if (a) they have acquired ne# financial interests related to the study and/or (b) any of their previously reported financial interests related to the study have changed.

IMPORTANT REMINDER: THE IRB HAS THE AUTHORITY TO TERMINATE PROJECTS THAT ARE NOT IN COMPLIANCE WITH THESE REQUIREMENTS.

z uestions related to this project may be directed to **Ednice, Monteiro | Tel: 282-1916 | Email: EEMONTEIRO@PARTNERS.ORG**

cc:

Ana-Maria, Vranceanu, , Psychiatry, Psychiatry, Principal Investigator

Sofia, Distefano, BS, Neurology, Neurology, Research Coordinator/Manager

Partners Human Research
Partners HealthCare
399 Revolution Drive, Suite 710
Somerville, MA 02145
Tel: 857-282-1900
Fax: 857-282-5693

Ann, Lin, , Psychiatry, Psychiatry, Research Coordinator/Manager

Melissa, Gates, , Psychiatry, Psychiatry, Research Coordinator/Manager

PARTNERS HUMAN RESEARCH COMMITTEE DETAILED PROTOCOL

Principal Investigator: Ana-Maria Vranceanu, PhD

Protocol Title: Recovering Together: Building resiliency in dyads in patients admitted to the Neuroscience Intensive Care Unit (NICU) and their caregivers

Funding: National Institute of Nursing Research

Version Date: 2/7/2019

I. BACKGROUND AND SIGNIFICANCE

Acute neurological illnesses (ANIs) are common, costly and often lead to long-term disability. ANIs are biologically distinct injuries that disrupt the normal function of the brain. The most common ANIs in Neuroscience Intensive Care Units (NICU) include cerebrovascular (stroke/hemorrhage and brain aneurysm), structural (tumors and lesions/brain masses), and traumatic (TBI) brain injuries. NICU admissions for ANIs are prevalent (e.g., 795,000 acute stroke/year; 275,000 acute TBI/year) and costly[1,2]; post NICU prolonged rehabilitation is common[3].

ANIs are associated with chronic emotional distress in both patients (pts) and caregivers (cgs). Although biologically heterogeneous, ANIs are unified by sudden onset, and substantial emotional distress in both pts (e.g., 12-43% anxiety[4-7]; 10-58% depression[4,7,8]; 20-29% post-traumatic stress PTS[5,9]) and family cgs (27-60% depression, anxiety or PTS [4,10,11]). These symptoms often become chronic and treatment resistant[26,27].

Pt and cg factors interact and influence physical and emotional outcomes in both pts and cgs. Post ANI emotional distress is associated with pts' poor medical adherence[28], slower recovery[28-30], higher mortality[29-31], and need of more caregiving assistance[32], which further increase cgs' distress[30,33,34] and own risk for morbidity[35,36] and mortality[37]; in turn cgs' emotional distress interferes with ability to provide high-quality care to pts[38,39] and negatively impacts pts' outcomes.

Current management of ANIs does not meet the psychological needs of pts and cgs for 3 reasons[40-51]. First, although recognition of the emotional burden associated with NICU admission has increased, and some NICUs have social workers available to assist pts and cgs, there are no formal screening methods for emotional distress routinely integrated in practice during hospitalization, when the primary focus is on medical care and survival; further, there are no formal evidence-based treatments integrated within the medical care. When social workers are included to help pts and cgs, the care is brief and occurs only during hospitalization. When referrals to mental health services are provided to families at discharge, few will access additional treatment due to burden associated with traveling outside of home. Second, psychosocial interventions available for ANI pts or cgs are limited in that they are delivered when symptoms are already chronic, address only one emotional illness (e.g., depression or anxiety or PTS), and/or are focused on a *single member* of the pt-cg dyad. Even interventions labeled as "dyadic," which include pts and cgs, typically address only the pts' needs and do not

focus on cg outcomes or on the dyad's interpersonal communication and bond/relationship[12,52]. These interventions are not consistent with the *dyadic framework*[22] which specifies that dyadic interventions should account for the interdependence between pt and cg psychosocial factors including their interpersonal bond by ensuring that both pts and cgs attend each session together, and by targeting improvement in outcomes for both pts and cgs. Third, most interventions are delivered using uniform protocols. However, the needs of ANI dyads are heterogeneous due to varying levels of post ANI impairment, identity of the cg, context and stage of life. A recent systematic review[52] urged for the development of dyadic interventions that address the needs of *both* pts and cgs and are tailored to the specific needs of each ANI dyad.

We developed the first dyadic skills-based intervention – Recovering Together - to prevent chronic heightened emotional distress in at risk ANI pt-cg dyads. The "Recovering Together" program is informed by the theoretical response-shift framework of adaptation to acute illness[53] (successful adaptation implies recalibration of values and life goals), the family strength vulnerability model[24] (within dyads relational systems have strengths and weaknesses in how they cope with life events), the dyadic longitudinal model[22] (distress travel from one member of the dyad to the other across time), the APIM model[54], and the resiliency framework[17]**.** The program is in line with recent recommendations for skill-based interventions for critical care patients, and uses preliminary data collected by our team for the past 3 years[4, 13-15]. The intervention teaches pts and cgs resiliency factors that are associated with well-being after trauma for both pts and cgs: *mindfulness* – the ability to stay present and defer judgment in the face of adversity[18]; *coping*– the arsenal and application of one's behavioral, cognitive, and emotional strategies to manage stress[19]; *social support* –empathetic interpersonal interactions that meet one's emotional and functional needs[20]; *self-efficacy* – perceived ability to adapt under adversity[21] and *positive dyadic interpersonal communication to increase interpersonal bond*[12]. Informed by the aforementioned theoretical models, our conceptual model hypothesizes that by teaching both members of the dyad resiliency and interpersonal communication skills (e.g., Recovering Together) we will be able to sustainably decrease emotional distress in both members of the dyad

In addition, our team has an established record of collaboration on published or ongoing investigations. **Emotional distress is prevalent in dyads, interdependent between pt and cg, and negatively associated with resiliency factors[4].** Our team conducted a cross-sectional study of pt-cg dyads in the NICU (40% stroke, 30% tumor). 75% pts and 84% cgs approached agreed to participate. 74% pts had been intubated at one time during NICU admission, and 2/3 were discharged home. Rates of clinically significant symptoms of depression, anxiety and PTS did not differ between pts (24%, 43%, 21%) and cgs (24%, 46%, 17%), or by any demographic or medical characteristic. Dyadic modeling showed that for both pts and cgs, mindfulness and coping impacted both self and partner's emotional distress symptoms. We showed: 1) feasibility of recruitment; 2) high emotional distress in dyads; and that 3) modifiable resiliency factors (mindfulness and coping) are intervention targets interdependently associated with distress in pts and cgs, regardless of the identity of cg (e.g., spouse, friend, etc).

Resiliency factors are associated with lower emotional distress in NICU dyads[13]. Our team found that resiliency factors of mindfulness, coping, self-efficacy and patient-caregiver interactions were associated with decreased emotional distress in dyads of ANI. This study confirms mindfulness, coping as intervention targets and provides novel evidence on self-efficacy and patient-caregiver interaction as additional important intervention targets.

ANI pts have greater anxiety than cancer patients at early diagnoses[14]. We led the first cross-comparison study of emotional distress among dyads with ANI and cancer. This study supports the priority of addressing emotional distress in ANI dyads as has been emphasized for cancer dyads.

Clinically significant emotional distress in one member of the dyad at hospitalization predicts chronic emotional distress in at least 1 of the dyad members 3 and 6 months later[55]. Our team has an ongoing prospective study of dyads with ANI. Retention rates for dyads due for assessments at 3 and 6 months thus far are 84% and 91% for pts and 87.7% and 95.7% for cgs, confirming our ability to retain post-ANI participants. Within each dyad, if one member screens in for clinically significant symptoms for any diagnosis (i.e., depression, anxiety or PTSD) at hospitalization there is good sensitivity and specificity that one member of the dyad will endorse clinically significant symptoms 3 months later. This study shows a reliable method for identifying dyads of patients at risk for chronic heightened emotional distress by identifying dyads in which either the pt or cg screens in for heightened emotional distress (symptoms of depression, anxiety or PTSD).

Caregiver gender moderates the prospective association of resiliency factors to emotional distress[87]. This study found that at the time of admission resiliency factors have main effects on emotional distress, with no differences by cg gender. However, significant interaction effects emerged prospectively such that male cgs with high mindfulness at baseline demonstrated lower levels of emotional distress at 3 and 6 months later than did males with low mindfulness ($p = 0.026$ and $p <0.013$). Similarly, women cgs with high intimate bond at baseline reported the lowest levels of depression symptoms 3 and 6 months later ($p < 0.020$). This study confirmed the need to assess and address resiliency factors early in the recovery process, and identified important gender differences to be accounted for in intervention development.

Recovering Together; Developing a novel dyadic resiliency skills program for ANI pt-cg dyads at risk for chronic emotional distress[15]. With funding from American Heart Association, we conducted 20 qualitative interviews with pt-cg stroke dyads at risk for chronic emotional distress during NICU hospitalization. We also conducted additional clinical interviews with 10 pt-cg dyads representative of other ANI diagnoses. 83% dyads approached agreed to participate. Pts (23) and cgs (25) were mostly women. Dyads were mostly spouses and mothers-daughter. Data was analyzed with Nvivo10. Main themes did not differ by medical diagnoses: 1) most challenging and distressing experiences: uncertainty about future, anxiety, depression, sleep difficulties, worries about the future, guilt, managing job with caretaking, making treatment decisions, lack of predictability; 2) concerns about interpersonal relationships (self-image, role changes, role fulfillment); 3) fear of recurrence; 4) adjusting to sequelae. Dyads noted interest in a resiliency program (30/30) and preferred a combination of in person and live video sessions (30/30). Dyads learned about resiliency skills (e.g., name, description and goal) and agreed they would be helpful to them. They also noted interest in learning about: survivorship plans, adaptation to deficits (present or anticipated), and return to normal living. We found no thematic differences between stroke dyads and other ANI dyads. Challenges associated with embracing the caregiver role emerged as a theme while differences by the identity of caregiver (e.g., spouse vs. friend, vs parent) did not. Themes associated with the gender of the cg emerged and have been incorporated in the intervention.

Nurses perception of the needs for and feasibility of "Recovering Together for ANI families[25]. We conducted 2 focus groups (N =15) with NICU nurses who provided feedback on the qualitative findings from our 30 ANI dyads and shared own experiences and opinions on

implementation and scalability of the intervention including nurse involvement. Nurses concurred with pts experiences, and suggested strategies to recruit and retain dyads for the study, which are now included in the methodology section of the current grant proposal. Studies 3.6 and 3.7 represent the building blocks for the development of our Recovering Together program and manual. Nurses provided edits and contributed to the iterative development of the manual. These studies also confirm feasibility of conducting the pilot RCT proposed through this R21.

II. SPECIFIC AIMS

The current study has the following objectives:

Aim 1: **To determine the feasibility of recruitment, feasibility of program delivery, program credibility, and program satisfaction using evidence-based benchmarks.**

Hypothesis 1: We hypothesize that > 75% of the dyads approached will agree to participate.
Hypothesis 2: We hypothesize that > 75% of dyads who start the intervention will complete at least 4 sessions.
Hypothesis 3: We hypothesize that > 75% of participants will report average credibility (Credibility and Expectancy Questionnaire) scores greater than the scale's midpoint.
Hypothesis 4: We hypothesize that > 75% participants will report average satisfaction (Client Satisfaction Scale) scores greater than the scale's midpoint.

Aim 2: **To demonstrate a proof of concept that the Recovering Together program can sustainably improve emotional distress [Hospital Anxiety and Depression Scale; HADS], Post Traumatic Symptoms (PTS) [PCL-S], resiliency variables (mindfulness, coping, social support and self-efficacy) and interpersonal factors (interpersonal bond).**

Hypothesis 1: We hypothesize that participation in the Recovering Together Program will be associated with a more potent decrease in emotional distress and PTS compared to participation in the educational program (control), and that these improvements will maintain at 3 month follow up.
Hypothesis 2: We hypothesize that Recovering Together Program will be associated with a more potent increase in resiliency variables (mindfulness, coping, social support, self-efficacy) and interpersonal factors (interpersonal bond) compared to participation in the educational program, and that these improvements will maintain at 3 month follow up.

For this feasibility pilot RCT, our primary outcomes (feasibility, credibility and satisfaction) will be assessed in Aim 1. Our secondary outcomes in this trial are: emotional distress, PTS, mindfulness, coping, social support, self-efficacy, and interpersonal bond, and will be assessed in Aim 2.

III. SUBJECT SELECTION

All participants will be recruited from the Massachusetts General Hospital Neuroscience ICU, using IRB approved recruitment materials.

Inclusion/Exclusion Criteria

Eligible dyads (Pts and Cgs) must meet the following inclusion criteria:

1) Age 18 or older
2) English fluency and literacy
3) Access to high speed internet for video sessions
4) Pt with an informal cg (family or friend who provides unpaid care) available and willing to participate
5) Hospitalized with an ANI within 1-2 weeks (pt) OR primary cg of a pt currently admitted with an ANI
6) Either pt or cg have clinically significant symptoms of depression, anxiety, and/or PTS

One or more of the following exclusion criteria will render a pt ineligible:

1) Permanent and severe cognitive impairment severe enough to impede participation – This will be determined by trained study staff through an assessment conducted as part of usual care and that includes the MMSE (score of <23) and GCS (score of <10). Nurses and study staff are trained and use these measures as part of NICU care.
2) Dyads where the pt is anticipated to die or to never be able to participate due to medical sequelae. This will be determined by nurses.

All adult patients and family caregivers, satisfying all inclusion criteria, are eligible for enrollment in this study regardless of sex, race, or ethnicity. Vulnerable populations will not be recruited.

Recruitment

Patients will be recruited from the NICU at MGH from the medical team (nurses). Recruitment will be facilitated by the nursing team who will introduce the study to eligible dyads and who will also assess whether pts are able to consent, consistent with medical presentation, and cognitive status. Trained study staff will be administering the MMSE and nurses will administer the GSC, to all patients and will refer only dyads where the patients scores are higher than the established cut off scores on these measures. Consistent with NICU practice, nurses will administer the GSC. They will refer participants to the study only if they are cleared medically and cognitively. The team will page the RA using the secure Voalte system used by our team and nursing staff, when both the pt and cg are present and able to hear more about the study. The RA will only approach pts identified and cleared by the medical team. The RA will ensure eligibility of both pts and cgs based on the additional inclusionary and exclusionary criteria depicted above. The RA will finalize the screening and conduct informed consent**.** All participants will receive a physical copy of the informed consent form which has the contact information for the PI. If cg~~s~~ cannot be reached in person in the hospital, they will be contacted via phone for screening and completion of informed consent. Cgs will be given a copy of the consent form to review while discussing the study over the telephone. We will fax or securely e-mail the consent form prior to obtaining consent. Cg will return a signed copy of the consent form.

Patients and caregivers will be considered enrolled when they sign the consent form with the study staff. Please see details on the informed consent process in the next section, Subject Enrollment (Section IV). These procedures will be completed in a private medical space.

Participants will be explicitly informed that this intervention is a research study that does not constitute individualized, personal care. The intervention is a broad-based method of training that is not tailored to any particular individual. Should any participant seek formal mental healthcare, study staff will refer them to either MGH Psychiatry, as appropriate.

We will recruit pts with any type of ANI and their cgs, ensuring representation of all NICU diagnoses. All cgs will be recruited within the first week of the pts' hospitalization. Pt's medical team will also alert the research assistant whether they anticipate that the patient might be able to participate in the study at a future point during the hospital stay. In situations where patients are unable to consent due to the severity of the ANI, we will enroll the informal cg and return to enroll the pt as soon as their mental capacity improves. The research assistant will not approach patients who are not mentally or physically capable to participate. This study does not include participants with impaired decision making.

We will not include patients who do not have a caregiver. Informal caregivers will be designated by the health care proxy and verbally confirmed by the patient. Nurses will assist the study team with identifying the patient's health care proxy.

Eligible cases may also be identified by daily screening of Epic admission reports.

The study team will keep track of all pts and cgs approached who refuse to participate (along with reasons for refusal), as well as those who were not approached and reasons why.

When the RA approaches a patient, patients and caregivers will be given the choice of watching a short recruitment video that contains testimonials from previous patients.

The recruitment process was developed and refined through prior research for the past 2 years.

IV. SUBJECT ENROLLMENT

Participants will be referred to the study by the nursing team who will ensure that patients are medically and cognitively able to participate. Eligible dyads will next be screened, consented, and enrolled by the research assistant.

After determining eligibility, study staff will meet with potential dyads to review the informed consent document. If cgs cannot be reached in person in the hospital, they will be contacted via phone to complete informed consent. Cgs will be given a copy of the consent form to review while discussing the study over the telephone. We will fax or securely e-mail the consent form prior to obtaining consent. Cgs will return a signed copy of the consent form. After the document has been reviewed, study staff will answer any and all questions the pt or cg may have. Once all questions have been addressed, each member of the dyad will sign the consent form, which will

include a description of all study procedures, the option to receive text message reminders, information about potential risks and benefits of participation, and study contact information (including that of the IRB) in case questions arise at a later time. The consent form will also explicitly state that study participation is voluntary, and that participants may refuse to answer any questions that make them uncomfortable, and may discontinue participation at any time. In addition, participants will be assured that withdrawal from the study will not compromise their medical care in any way.

As informed consent is a continuous process, participants will be given a copy of the signed informed consent document, and will be invited to ask questions about their participation at any point over the course of the study.

Following a study enrollment and baseline assessment, dyads will be randomly assigned to either the newly developed psychosocial intervention or to the educational program (control) using a random number sequence generator to ensure comparability between groups. We will document time between assessment and intervention initiation, and analyze as a predictor of study outcomes as needed. Randomization will be developed by the statistician, without any input from the rest of the team.

V. STUDY PROCEDURES

After enrollment, participants will complete study assessments. All subjects will be given baseline psychological and behavioral assessments that will assess depression, anxiety, PTS symptoms, and other psychological constructs. Assessments will be administered online, using the REDCap system. Subjects may choose to fill out these questionnaires on-site, or to fill them out at home on a personal computer or other Internet-equipped device. This assessment includes demographic information and a battery of psychological questionnaires. We will also collect information about important clinical variables including duration of acute hospitalization. All questionnaires are itemized below:

Administered at Baseline Only:
Demographics
Prior Mental Health History questions
Credibility Questionnaire

Administered at Baseline, Post-Intervention, and Follow-up:
Medical history information from LMR (i.e., prior ANI status, current psychotropic meds, comorbid medical conditions, etc.)
Post-Traumatic Stress Disorder Checklist (PCL-S)[29]
Hospital Depression and Anxiety Scale (HADS)[30]
Measure of Current Status Part A (MOCS-A)[31]
The Cognitive and Affective Mindfulness Scale (CAMS)[32]
World Health Organization Quality of Life (WHOQOL-BREF)[33]
Dyadic Relationship Scale (DRS)
Experience in Close Relationships – Relationship Structure (ECR-RS)

Administered Post-Intervention and Follow-up Only:
Client Satisfaction Questionnaire (CSQ-3)[34]

The medical team will complete the following questionnaires to assess stroke severity for each patient:
Modified Rankin Scale (mRS)[35] [Pts only]
Barthel Index[36] [Pts only]

After completing the baseline assessments, dyads will be randomized to either the newly developed psychosocial intervention or to treatment as usual.

The psychosocial intervention entails 6 sessions, each 30 minutes. Both the pt and cg participate in each session. The intervention is manualized, and teaches dyads resiliency (mindfulness, social support, self-efficacy, coping skills) and interpersonal communications (interpersonal bond) skills. The first 2 sessions will occur in person, during hospitalization. The next 4 sessions are chosen from 5 available, depending on each dyad's preference, and are delivered after discharge via secure live video. For the video sessions, dyads can participate from the same or different locations. During the in hospital sessions, dyads learn diaphragmatic breathing, mindfulness, self-care, and dialectics. During the video sessions, dyads learn how to identify negative thinking patterns and replace them with adaptive thoughts, how to communicate effectively and openly, how to engage in self-care, and how to accept things that can't be changed. Participants in the intervention group will also receive treatment as usual. This may include meeting with nurses, physical therapists, medical doctors, and other members of the pt's medical team. Treatment as usual may also involve administration of SSRI to those patients with motor problems.

Those in the educational program (control) will receive general health information that mimics the Recovering Together Program, but without teaching any of the resiliency or interpersonal communication skills that are hypothesized to be responsible for improvement in emotional distress. The educational program will also have 6 sessions, 2 in-person dyadic visits in the NICU and 4 dyadic virtual visits following discharge. It controls for dose and support from clinician. Both members of the dyads participate in all sessions. The topics of each session include: education about the stress of the ANI on patient and caregiver; education on the importance of self-care; education on stress associated with discharge and home adjustment; education on the importance of following up with medical recommendations; education on interpersonal stress as part of adjustment to ANI; education on self-care. The educational program condition will ensure that patients will remain blind to intervention or control and increase confidence that improvement in outcomes are due to the active ingredients of the intervention and not confounds. Participants in the educational program will continue with their current care. This may include meeting with nurses, physical therapists, medical doctors, and other members of the pt's medical team. Treatment as usual may also involve administration of SSRI to those patients with motor problems.

Participants in both groups will be given post-treatment psychological and behavioral assessments identical to those administered at baseline, in addition to the CSQ-3. As a baseline, participants will be given the option to complete post-treatment questionnaires on site or at home. Participants will be asked to complete questionnaires immediately after completion of the group intervention (T2), and 3 months (T3) after completion of the group intervention, in order to measure long term outcomes. Study staff will email these questionnaires to the participants via the REDCap system.

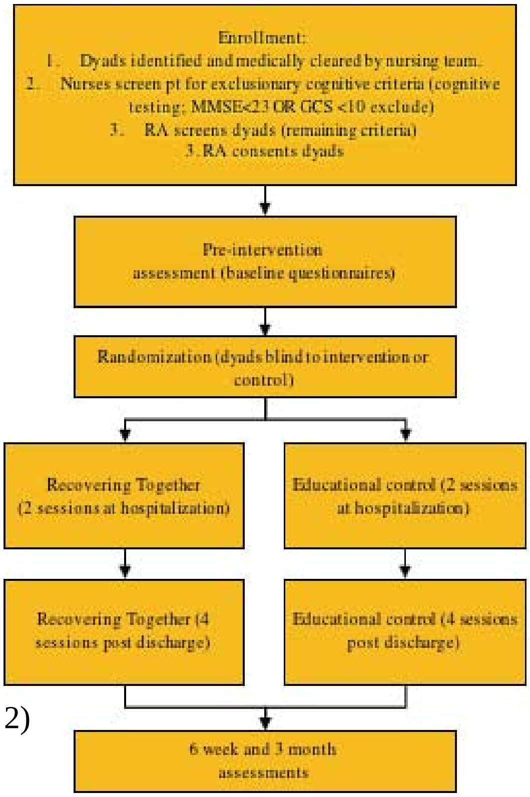

VI. BIOSTATISTIC ANALYSIS

We plan to conduct a RCT with patients from the Neuro-ICU. The main intervention goal is to provide dyads with resiliency and interpersonal communication skills necessary to optimize recovery. Dyads will be medically cleared by a member of the nursing staff. Nurses will then screen dyads for exclusionary cognitive criteria. Dyads will complete additional screening and baseline questionnaires with a trained research assistant. Dyads will be randomized to one of two groups 1) Psychosocial skills based intervention (Recovering Together), or 2) Educational Control.

We chose our study measures based on our theoretical frameworks, strong psychometric properties in studies of patients with ANI, and feasibility in our prior work with ANI dyads. Measures will be collected at baseline, post-intervention, and at 3 month follow up. Data collection and management will be conducted with Research Electronic Data Capture. Dyads will be given the option to complete measures electronically, by paper and pencil, or telephone.

1) Is the psychosocial skills-based intervention feasible, credible usable, and accepted by pts and cgs in the ICU?

Our primary aim focuses on trial feasibility, acceptability, credibility and preliminary effect. In pilot studies, ≥30 participants are recommended per group to establish feasibility and detect larger effect sizes for ≥1 outcome. We plan to recruit 80 dyads (160 participants), 40 dyads (80 participants) per arm to establish feasibility, acceptability, credibility and estimate effect size for emotional distress variables (primary quantitative outcomes). Assuming attrition of over 25% (in excess of what we experienced in our preliminary studies), we will have the necessary 30 dyads (60 participants) per arm. This size is considered to yield stable estimates of M/SDs based on prior behavioral trial recommendations. Effect sizes from this study may overestimate power in future sample calculations. We chose study measures based on our theoretical frameworks, strong psychometric properties in studies of patients with ANI, and feasibility work in our prior work with ANI dyads. Feasibility will be reported as the percentage of patients enrolled

in the study who complete at least 75% of the intervention sessions. Demonstration of feasibility will be assessed by the number of individuals who drop out of the study prior to completing the post- intervention assessment and the rate of missed sessions. If drop-out rate or missed-session rate exceeds 25%, revisions to the intervention may be needed. We will also report number of patients approached, enrolled, randomized and who completed time 2 and time 3 to determine feasibility.

2) Is the psychosocial skills-based intervention effective for pts and cgs in the ICU?

Dr. Vranceanu developed the proposed study design in collaboration with the NICU study team (nurses, physicians, clinical interns) and an MGH psychologist who specializes in using mindfulness and emotion regulation in both chronic illness and medically healthy populations. With funding from the American Heart Association, we conducted 20 qualitative interviews with pt-cg stroke dyads at risk for chronic emotional distress during NICU hospitalization. We also conducted additional clinical interviews with 10 pt-cg dyads representative of other ANI diagnoses. 83% of dyads approached agreed to participate. Pts (23) and cgs (25) were mostly women. Dyads were mostly spouses and mothers-daughter. Dyads noted interest in a resiliency program (30/30) and preferred a combination of in person and live video sessions (30/30). Dyads learned about resiliency skills (name, description, and goal) and agreed they would be helpful to them. They also noted interest in learning about: survivorship plans, adaptation to deficits (present or anticipated), and return to normal living. This led to the development and subsequent refinement (through feedback from the nursing team) of the Recovering Together Program active intervention.

3) Is the effect of the skills-based intervention for pts and cgs in the ICU durable?

We will assess feasibility, usability, and acceptability by the enrollment numbers, participants completion in at least 4 out of 6 sessions, and the questionnaires, The Credibility and Expectancy and Client Satisfaction Scale. These will be primary outcomes. The PCL-S, HADS, and resiliency measures will serve as the secondary outcomes.

We will use student's t-test to assess within-group differences in long-term outcomes. We will compare measures at the 3 month follow-up assessment to measures at the post-intervention assessment for both study arms.

We will also use t tests and chi squared tests to assess differences at the 3 month follow up.

VII. RISKS AND DISCOMFORTS

There is a risk that some participants may feel uncomfortable completing various psychological questionnaires or parts of the skills-based on intervention. Participants are free to withdraw from the study at any time, as the study is completely voluntary.

As in any research study, there is a small risk that confidentiality may be breached; all efforts to minimize this risk will be taken. In the unlikely event that participants will become suicidal during the duration of the study, the research assistant will contact the PI and the appropriate clinical intervention will be executed.

VIII. POTENTIAL BENEFITS

Participants in this study may observe a reduction in depression, anxiety, and/or psychological and physiological markers of stress, as well as an improvement in perceived qualify of life. It is hoped that the intervention will result in improvements across these domains. Participants may learn new techniques for managing distress and lifestyle factors that may enhance wellbeing, both in disease-specific domains as well as in their general lives. In addition, all participants will receive $20 for the completion of each of the 3 assessment points.

IX. MONITORING AND QUALITY ASSURANCE

Electronic information will be stored in REDCap (Research Electronic Data Capture), a free, secure, and HIPAA-compliant web-based application hosted by the Partners HealthCare Research Computing Enterprise Research Infrastructure & Services (ERIS) group (based at the PHS Needham corporate datacenter). Data will be stored on password protected computers that will be stored in secure locations at all times. Paper data files (with coded subject identification) will be stored in a locked filing cabinet. Only research staff will have access to these data locations.

A unique anonymous identifier will be assigned to each subject; subsequently, all data collected will be associated exclusively with this identifier. This includes all questionnaires administered over the course of the study, as well as home practice logs.

Data from this study will be stored for three years after the publication of all study results, at which time all paper data files will be shredded, and computer files will be deleted.

Data Management and Quality Control Procedures

To maximize accuracy and security, all survey data will be collected and stored on REDCap. Research staff will ensure that proper consent has been obtained before sending the REDCap survey to each participant.

REDCap (Research Electronic Data Capture) is a free, secure, HIPAA compliant web-based application hosted by the Partners HealthCare Research Computing Enterprise Research Infrastructure & Services (ERIS) group. Vanderbilt University, with collaboration from a consortium of academic and non-profit institutional partners, has developed this software toolset and workflow methodology for electronic collection and management of research and clinical study data. Data collection projects rely on a study-specific data dictionary defined by members of the research team with planning assistance from Harvard Catalyst, The Harvard Clinical and Translational Science Center EDC Support Staff. This iterative development and testing process

results in a well-planned data collection strategy for individual studies. Using REDCap, the research team can also design web-based surveys and engage potential respondents using a variety of notification methods. REDCap provides flexible features that can be used for a variety of research projects and provides an intuitive interface to enter data with real time validation (automated data type and range checks). The system offers easy data manipulation with audit trails, reports for monitoring and querying participant records, and an automated export mechanism to common statistical packages (SPSS, SAS, Stata, R/S-Plus).

Since consistency of application of the study protocol is critical to acquiring high quality data, all research personnel have undergone or will undergo a competency-based training program prior to enrolling subjects.

Data and Safety Monitoring Plan

Adverse Event Monitoring: Throughout the study subjects will be monitored for the occurrence of events defined as any undesirable experience or unanticipated risk. Lack of effect of treatment is not considered an event. All adverse events will be reported on an adverse event form. The Principle Investigator has the responsibility of reporting serious adverse events (death, life threatening illness or injury, serious injury, or permanent disability) to PHRC within 24-72 hours of notification.

X. REFERENCES

1. Center for Disease Control and Prevention. (2015). Stroke Facts: Stroke in the United States. *CDC*. Retrieved from http://www.cdc.gov/stroke/facts.htm.
2. Center for Disease Control and Prevention. (2015). *Report to Congress on Traumatic Brain Injury in the United States: Epidemiology and Rehabilitation.* National Center for Injury Prevention and Control; Division of Unintentional Injury Prevention. Atlanta, GA.
3. The Society of Critical Care Medicine. (2015). What is critical care? *SCCM*. Retrieved from www.myicucare.org.
4. Shaffer KM, Riklin E, Stagl JM, Rosand J, Vranceanu AM. (2016). Mindfulness and coping are inversely related to psychiatric symptoms in patients and informal caregivers in the Neuroscience ICU: Implications for Clinical Care. Critical *Care Medicine*, 44 2036-2038. PMID: 27513536
5. Jackson, J.C., Mitchell, N., & Hopkins, R.O. (2011). Cognitive functioning, mental health, and quality of life in ICU survivors: an overview. *Anesthesiology Clinics, 29,* 751-764. PMID: 22078921
6. Kress, J.P., Gehlbach, B., Lacy, M., Pliskin, N., Pohlman, A.S., & Hall, J.B. (2003). The long-term psychological effects of daily sedative interruption on critically ill patients. *American Journal of Respiratory and Critical Care Medicine, 168,* 1457-1461. PMID: 14525802
7. Cheung, A.M., Tansey, C.M., Tomlinson, G., Diaz-Granados, N., Matté, A., Barr, A., ... Herridge, M.S. (2006). Two-year outcomes, health care use, and costs of survivors of acute respiratory distress syndrome. *American Journal of Respiratory and Critical Care Medicine, 174,* 538-544. PMID: 16763220
8. Daydow, D.S., Gifford, J.M., Desai, S.V., Bienvenu, O.J., & Needham, D.M. (2009). Depression in general intensive care unit survivors: a systematic review. *Intensive Care Medicine, 35,* 796-809. PMID: 19165464

9. Jackson, J.C., Hart, R.P., Gordon, S.M., Hopkins, R.O., Girard, T.D., & Ely, W.E. (2007). Post-traumatic stress disorder and post-traumatic stress symptoms following critical illness in medical intensive care unit patients: assessing the magnitude of the problem. *Critical Care, 11,* R27. PMCID: PMC2151890
10. Choi, J., Hoffman, J.A., Schulz, R., Ren, D., Donahoe, M.P., Given, B., & Sherwood, P.R. (2013). Health risk behaviors in family caregivers during patients' stay in intensive care units: a pilot analysis. *American Journal of Critical Care, 22,* 41-45. PMCID: PMC4109809
11. McAdam, J.L., Fontaine, D.K., White, D.B., Dracup, K.A., & Puntillo, K.A. (2012). Psychological symptoms of family members of high-risk intensive care unit patients. *American Journal of Critical Care, 21,* 386-393. PMID:23117902
12. McCarthy, M.J., Lyons, S.L., Powers, L.E. (2012). Relational factors associated with depressive symptoms among stroke survivor-spouse dyads. *Journal of Family Social Work* 15: 303-320.
13. Shaffer KM, Riklin E, Stagl JM, Rosand J, Vranceanu AM. (2016) Psychosocial resiliency is associated with lower psychological distress among dyads of patients and their informal caregivers in the neuroscience intensive care unit. J Critical Care 2016 Jul 16; 36:154-159. PMID: 27546765
14. Schaffer KM, Jacobs JM, Coleman JN, Rosand J, Temel, J, Greer JA. Vranceanu AM**.** Anxiety and depressive symptoms among two seriously ill medical populations and their family caregivers. Neurocritical Care 2016; ahead of print.
15. Zale EL, Piere-Louis C, Tehan T, Henhuis T, Rosand J., Vranceanu, A.M. Improving resiliency and brain health after acute neurological illness; Perceptions of patients and families. Accepted for presentation at Brain Health Conference, Columbus, OH April 28-30, 2017.
16. Martire LM, Lustig AP, Schulz R, Miller GE, Helgeson VS. Is it beneficial to involve a family member? A meta-analysis of psychosocial interventions for chronic illness. *Health Psychol* 2004; 23(6):599-611
17. Bonanno, G. A., Galea, S., Bucciarelli, A., & Vlahov, D. (2007). What predicts psychological resilience after disaster? The role of demographics, resources, and life stress. *Journal of Consulting and Clinical Psychology*, *75*,671–682. http://dx.doi.org/10.1037/0022-006X.75.5.671
18. Brown KW, Ryan RM (2003) The benefits of being present: mindfulness and its role in psychological well-being.J Pers Soc Psychol 84(4):822
19. Donnellan C, Hevey D, Hickey A, O'Neil D (2006) Defining and quantifying coping strategies after stroke: a review. J Neurol Neurosurg Psychiatry 77:1208-1218
20. Southwick SM, Vythilingam M, Charney DS (2005) The psychobiology of depression and resilience to stress:implications for prevention and treatment. Annu Rev Clin Psychol 1:255-291
21. Korpershoek C, van der Bijl J, Hafsteinsdóttir TB (2011) Self-efficacy and its influence on recovery of patients with stroke: a systematic review. J Adv Nurs 67(9):1876-1894
22. Savini, S., Buck, H.G., Dickson, V.V., Simeone, S., Pucciarelli, G., Fida, R., ... Vellone, E. (2015). Quality of life in stroke survivor-caregiver dyads: a new conceptual framework and longitudinal study protocol. *Journal of Advanced Nursing, 71*(3), 676-687. PMID: 25186274
23. Barclay-Goddard, R., King, J., Dubouloz, C.J., Schwartz, C.E., & Response Shift Think Tank Working Group. (2012). Building on transformative learning and response shift theory to investigate health-related quality of life changes over time in individuals with chronic health conditions and disability. *Archives of Physical Medicine and Rehabilitation, 93*, 214-220. PMID: 22289229
24. Shields, C.G., King, D.A., & Wynne, L.C. (1995). Interventions with later life families. In R.H. Mikesell, D.D. Lustermann, & S.H. McDaniel (Eds.), *Integrating Family Therapy: Handbook of*

Family Psychology and Systems Theory (pp. 141-158). Washington, DC: American Psychological Association.

25. Tehan T., Zale E., Rosand J, Vranceanu AM. Perceptions of needs and recommendations for implementation of a resiliency intervention for patients with stroke and their families; Nurses speak. Accepted for presentation at Brain Health Conference, Columbus, OH April 28-30, 2017
26. http://www.who.int/mental_health/media/en/545.pdf. Accessed March 3rd, 2017.
27. Collins PY, Patel V, Joestl SS, March D, Insel TR, Daar A, on behalf of the Grand Challenges in Global Mental Health Scientific Advisory Board and Executive Committee. Grand Challenges in Global Mental Health. *Nature.* 2011 July 7. 474(7354):27-30. PMID 21734685
28. Edmondson, D., Richardson, S., Fausett, J.K., Falzon, L., Howard, V.J., Kronish, I.M. (2013). Prevalence of PTSD in survivors of stroke and transient ischemic attack: a meta-analytic review. *PLoS One, 8,* e66435. PMCID: PMC3686746
29. Ayerbe, L., Ayis, S., Wolfe, C.D., Rudd, A.G. (2013). Natural history, predictors and outcomes of depression after stroke: systematic review and meta-analysis. *The British Journal of Psychiatry, 202,* 14-21. PMID:23284148
30. Carod-Artal, F.J., Egido, J.A. (2009). Quality of life after stroke: the importance of a good recovery. *Cerebrovascular Diseases, 27,* 204-214. PMID: 19342853
31. Bartoli, F., Lillia, N., Lax, A., Crocamo, C., Mantero, V., Carrà, G., Agostoni, E. Clerici, M. (2013). Depression after stroke and risk of mortality: a systematic review and meta-analysis. *Stroke Research and Treatment, 2013,* 862978. PMCID: PMC3606772
32. Denno, M.S., Gillard, P.J., Graham, G.D., DiBonaventura, M.D., Goren, A., Varon, S.F., Zorowitz, R. (2013). Anxiety and depression associated with caregiver burden in caregivers of stroke survivors with spasticity. *Archives of Physical Medicine and Rehabilitation, 94,*1731-1736. PMID: 23548544
33. Bakas, T., Burgener, S.C. (2002). Predictors of emotional distress, general health, and caregiving outcomes in family caregivers of stroke survivors. *Topics in Stroke Rehabilitation, 9,* 34-45. PMID: 14523721
34. Monin, J., Doyle, M., Levy, B., Schulz, R., Fried, T., Kershaw, T. (2016). Spousal associations between frailty and depressive symptoms: longitudinal findings from the cardiovascular health study. *Journal of the American Geriatrics Society, 64,* 824-830. PMID: 27100578
35. Lee, S,, Colditz, G.A., Berkman, L.F., Kawachi, I. (2003). Caregiving and risk of coronary heart disease in US women: a prospective study. *American Journal of Preventative Medicine, 24,*113-119. PMID: 12568816
36. Ji, J., Zöller, B., Sundquist, K., Sundquist, J. (2012). Increased risks of coronary heart disease and stroke among spousal caregivers of cancer patients. *Circulation, 125,* 1742-1747. PMID: 22415143
37. Schulz, R., Beach, S.R. (1999). Caregiving as a risk factor for mortality: the Caregiver Health Effects Study. *JAMA, 282,* 2215-2219. PMID: 10605972
38. Beach, S.R., Schulz, R., Williamson, G.M., Miller, L.S., Weiner, M.F., Lance, C.E. (2005). Risk factors for potentially harmful informal caregiver behavior. *Journal of the American Geriatrics Society, 53,* 255-261. PMID:15673349
39. Turner-Stokes, L., Hassan, N. (2002). Depression after stroke: a review of the evidence base to inform the development of an integrated care pathway. Part 1: Diagnosis, frequency and impact. *Clinical Rehabilitation, 16,* 231-247. PMID: 12017511
40. Schubart, J.R., Kinzie, M.B., & Farace, E. (2008). Caring for the brain tumor patient: family caregiver burden and unmet needs. *Neuro-oncology, 10,* 61-72. PMCID: PMC2600839

41. Palmer, S., & Glass, T.A. (2003). Family function and stroke recovery: a review. *Rehabilitation Psychology, 48,* 255-265.
42. Perrin, P.B., Heesacker, M., Hinojosa, M.S., Uthe, C.E., & Rittman, M.R. (2009). Identifying at-risk, ethnically diverse stroke caregivers for counseling: a longitudinal study of mental health. *Rehabilitation Psychology, 54,* 138-149. PMID: 19469603
43. Bienvenu, O.J., Colantuoni, E., Mendez-Tellez, P.A., Dinglas, V.D., Shanholtz, C., Husain, N., ... Needham,
D.M. (2012). Depressive symptoms and impaired physical function after acute lung injury. *American Journal of Respiratory and Critical Care Medicine, 185,* 517-524. PMCID: PMC3297105
44. Martin, L.R., Williams, S.L., Haskard, K.B., & DiMatteo, M.R. (2005). The challenge of patient adherence. *Therapeutics and Clinical Risk Management, 1,* 189-199. PMCID: PMC1661624
45. Rees, J., O'Boyle, C., & MacDonagh, R. (2001). Quality of life: impact of chronic illness on the partner. *Journal of the Royal Society of Medicine, 94,* 563-566. PMCID: PMC1282240
46. Azoulay, E., Pochard, F., Kentish-Barnes, N., Chevret, S., Aboab, J., Adrie, C., ... FAMIREA Study Group. (2005). Risk of post-traumatic stress symptoms in family members of intensive care unit patients. *American Journal of Respiratory and Critical Care Medicine, 171,* 987-994. PMID: 15665319
47. Jones, C., Skirrow, P., Griffiths, R.D., Humphris, G., Ingleby, S., Eddleston, J., ... Gager, M. (2004). Posttraumatic stress disorder-related symptoms in relatives of patients following intensive care. *Intensive Care Medicine, 30,* 456-460. PMID: 14767589
48. Jones, C., & Griffiths, R.D. (2007). Patient and caregiver counselling after the intensive care unit: what are the needs and how should they be met? *Current Opinion in Critical Care, 13,* 503-507. PMID: 17762226
49. Im, K., Belle, S.H., Shulz, R., Mendelsohn, A.B., Chelluri, L., & QOL-MV Investigators. (2004). Prevalence and outcomes of caregiving after prolonged (> or =48 hours) mechanical ventilation in the ICU. *Chest, 125,* 597-606. PMID: 14769744
50. Douglas, S.L., Daly, B.J., Kelley, C.G., O'Toole, E., & Montenegro, H. (2005). Impact of a disease management
program upon caregivers of chronically ill patients. *Chest, 128,* 3925-3936. PMID: 16354865
51. Martire LM, Lustig AP, Schulz R, Miller GE, Helgeson VS. Is it beneficial to involve a family member? A metaanalysis of psychosocial interventions for chronic illness. *Health Psychol* 2004; 23(6):599-611
52. Bakas, T., Clark, P.C., Kelly-Hayes, M., King, R.B., Lutz, B.J., Miller, E.L., & American Heart Association Council on Cardiovascular and Stroke Nursing and the Stroke Council. (2014). Evidence for stroke family caregiver and dyad interventions: a statement for healthcare professionals from the American Heart Association and American Stroke Association. *Stroke, 45,* 2836-2852. PMID: 25034718
53. Barclay-Goddard, R., King, J., Dubouloz, C.J., Schwartz, C.E., & Response Shift Think Tank Working Group. (2012). Building on transformative learning and response shift theory to investigate health-related quality of life changes over time in individuals with chronic health conditions and disability. *Archives of Physical Medicine and Rehabilitation, 93,* 214-220. PMID: 22289229
54. Cook, W.L. & Kenny, D.A. (2005). The Actor-Partner Interdependence Model: A model of bidirectional effects in developmental studies. *Journal of Behavioral Development, 29,* 102-109.
55. Zale EL, McCurley JL, Lin A, Funes C, Tehan T, Henhuis T, Rosand J., Vranceanu, A.M. Early psychological distress is crossectionally and prospectively interdependent between patients

admitted to the Neuro-ICU and their family caregivers. Submitted for presentation at Society of Behavioral Medicine, New Orleans, April 28-30, 2018.

56. Richards, K.C., Enderlin, C.A., Beck, C., McSweeney, J.C., Jones, T.C., & Roberson, P.K. (2007). Tailored biobehavioral interventions: a literature review and synthesis. *Research and Theory for Nursing Practices, 21*, 271-285. PMID: 18236771
57. Vranceanu AM, Ricklin E, Merker V, Park E, Plotkin SR. (2016) Mind body therapy for patients with
neurofibromatosis via live video; An RCT. Neurology 87 (8):806-14.
58. Zale EL, Piere-Louis C, Riklin E, Macklin E, Vranceanu AM. The impact of a mind body program on multiple dimensions of resiliency in geographically diverse patients with neurofibromatosis. JCCP accepted.
59. Bellg AJ, Borrelli B, Resnick B, et al. (2004). Enhancing treatment fidelity in health behavior change studies: Best practices and recommendations from the NIH Behavior Change Consortium. *Health Psychol.* 23:443-451. PMID:15367063.
60. Vranceanu AM**,** Merker VL, Plotkin SR, Park ER. The Relaxation Response Resiliency Program (3RP) in patients with neurofibromatosis 1, neurofibromatosis 2, and schwannomatosis: results from a pilot study. J Neurooncol. 2014; 120(1): 103-109.
61. Rounsavile, B.J., Carroll, K.M., & Onken, L.S. (2001). A stage model of behavioral therapies research: getting started and moving on from stage I. *Clinical Psychology: Science and Practice, 8*, 133-142.
62. Larsen, D.L., Attkisson, C.C., Hargreaves, W.A., & Nguyen, T.D. (1979). Assessment of client/patient satisfaction: development of a general scale. *Evaluation and Program Planning, 2*, 197-207. PMID: 10245370
63. Devilly, G.J., & Borkovec, T.D. (2000). Psychometric properties of the credibility/expectancy questionnaire. *Journal of Behavior Therapy and Experimental Psychiatry, 31,* 73-86. PMID: 11132119
64. Zigmond, A.S. & Snaith, R.P. (1983). The hospital anxiety and depression scale. *Acta Psychiatrica Scandinavica, 67,* 361-370. PMID: 6880820
65. Bhandari, N.J., Jain, T., Marolda, C., & ZuWallack, R.L. (2013). Comprehensive pulmonary rehabilitation results in clinically meaningful improvements in anxiety and depression in patients with chronic obstructive pulmonary disease. *Journal of Cardiopulmonary Rehabilitation and Prevention, 33,* 123-127. PMID: 23399845
66. Blanchard, E.B., Jones-Alexander, J., Buckley, T.C., & Forneris, C.A. (1996). Psychometric properties of the PTSD Checklist (PCL). *Behaviour Research and Therapy, 34,* 669-673. PMID: 8870294
67. Monson, C.M., Gradus, J.L., Young-Xu, Y., Schnurr, P.P., Price, J.L., & Schumm, J.A. (2008). Change in posttraumatic stress disorder symptoms: do clinicians and patients agree? *Psychological Assessment, 20,* 131-138. PMID: 18557690
68. Feldman, G., Hates, A., Kumar, S., Greeson, J., & Laurenceau, J.P. (2007). Mindfulness and emotion regulation: The development and initial validation of the Cognitive and Affective Mindfulness Scale Revised (CAMS-R). *Journal of Psychopathology and Behavioral Assessment, 29*, 177-190.
69. Schwarzer, R. & Jerusalem, M. (1995). Generalized Self-Efficacy scale. In J. Weinman, S. Wright, & M. Johnston (Eds.), *Measures in health psychology: A user's portfolio. Causal and control beliefs* (pp. 35-37). Windsor, UK: NFER-NELSON.

70. Cohen, S., Mermelstein, R., Kamarck, T., & Hoberman, H.M. (1985). Measuring the functional components of social support. In I.G. Sarason & B.R. Sarason (Eds.), *Social Support: Theory, Research and Applications* (pp.73-94). Netherlands: Springer.
71. Carver CS. (2006). Measure of Current Status. http://www.psy.miami.edu/faculty/ccarver/sclMOCS.html
72. Merz, EL, Roesch, SC, Malcarne, VL, Penedo, FJ, Llabre, MM, Weitzman, OB, ... Johnson, TP. (2013). Validation of interpersonal support evaluation list-12 (ISEL-12) scores among English- and Spanish-speaking Hispanics/Latinos from the HCHS/SOL sociocultural ancillary study. *Psychological Assessment* 26(2): 384-394.
73. Wilhelm, K. & Parker, G. (1988). The development of a measure of intimate bonds. *Psychological Medicine*, *18*, 225-234. PMID: 3363041
74. Guest, G., Bunce, A., & Johnson, L. (2006). How many interviews are enough? An experiment with data saturation and variability. *Field Methods, 18*(1), 24. doi: 10.1177/1525822X05279903
75. Browne RH. On the use of a pilot sample for sample size determination. *Stat Med*.1995;14: 1933-40. PMID:8532986.
76. Lancaster GA, Dodd S, Williamson PR. (2004). Design and analysis of pilot studies: recommendations for good practice. *J Eval Clin Pract.*;10:307-12. PMID: 15189396.
77. Rounsaville BJ, Caroll KM, Onken LS. (2001)A stage model of behavioral therapies research: getting started and moving on from stage I. *Clin Psychol Sci Pract*. 8:133–142
78. Shih WJ, Ohman-Strickland PA, Lin Y. (2004) Analysis of pilot and early phase studies with small sample sizes. *Stat Med.* 23:1827–1842 PMID: 15195318.
79. Whitehead AL, Julious SA, Cooper CL, Campbell MJ (2016). Estimating the sample size for a pilot randomized trial to minimise the overall trial sample size for the external pilot and main trial for a continuous outcome variable. *Stat Methods Med Res* 25:1057-1073. PMID: 26092476.
80. Miles, H.B., Huberman, A.M.(1994). *Qualitative Data Analysis.* Sage Publication, Thousand Oaks California.
81. Bowen DJ, Kreuter M, Spring B, et al. (2009) How we design feasibility studies. *Am J Prev Med.* May;36(5):452-7. PMID: 19362699.
82. Schafer JL, Graham JW. (2002). Missing data: our view of the state of the art. *Psychol Methods* 7(2):147-77. PMID: 12090408.
83. National Alliance for Caregiving (NAC). (2015). *Caregiving in the U.S. 2015.* Bethesda, MD: AARP Public Policy Institute.
84. Ruskin, P.E., Silver-Aylaian, M., Kling, M.A., Reed, S.A., Bradham, D.D., Hebel, J.R., ... Hauser, P. (2004). Treatment outcomes in depression: comparison of remote treatment through telepsychiatry to in-person treatment. *The American Journal of Psychiatry, 161,* 1471-1476. PMID: 15285975
85. Carlbring, P., & Andersson, G. (2006). Internet and psychological treatment. How well can they be combined? *Computers in Human Behavior, 22,* 545-553.
86. Clough, B.A., & Casey, L.M. (2011). Technological adjuncts to increase adherence to therapy: a review. *Clinical Psychology Review, 31,* 697-710. PMID: 21497153
87. Lin A, Jacobo M, Jacobs J, Tehan T, Salgueiro D, Rosand J, Vranceanu AM, Zale E. Gender differences in emotional distress among caregivers of patients admitted to the Neuroscience-Intensive Care. Submitted to the Society of Behavioral Medicine Annual Meeting, New Orleans, April 2018
88. Blake H, McKinney K, Treece E, Lee NB. (2002) An evaluation of screening measures for cognitive functioning after stroke. Age and Ageing, 31, pp.451-456.

Partners Human Research
Partners HealthCare
399 Revolution Drive, Suite 710
Somerville, MA 02145
Tel: 857-282-1900
Fax: 857-282-5693

Notification of IRB Review

Protocol #: 2018P002187

Date:	March 11, 2019
To:	Vranceanu, Ana-Maria,<br>MGH<br>Partners > MGH > Psychiatry
From:	Partners Human Research<br>399 Revolution Drive, Suite 710<br>Somerville, MA 02145
Title of Protocol:	Recovering Together: Building resiliency in dyads in patients admitted to the Neuroscience Intensive Care Unit (NICU) and their caregivers
Version/Number:	1
Version Date:	09/24/2018
Sponsor/Funding/Support:	Proposal Title: Recovering together: Building resiliency in dyads of patients admitted to the Neuroscience Intensive Care Unit (NICU) and their caregivers<br>Principal Investigator: Vranceanu, Ana-Maria<br>Immediate Sponsor: NIH<br>Award Number: 1R21NR017979-01A1<br>Fund #: 233585

IRB Amendment #:	9
IRB Review Type:	Expedited
IRB Approval Date:	03/10/2019
Approval/Activation Date:	03/11/2019
**IRB Expiration Date:**	**10/26/2019**

This project has been reviewed and approved by the **PHS IRB**. During the review of this project, the IRB specifically considered (i) the risks and anticipated benefits, if any, to subjects; (ii) the selection of subjects; (iii) the procedures for obtaining and documenting informed consent; (iv) the safety of subjects; and (v) the privacy of subjects and confidentiality of the data.

Please note that if an IRB member had a conflict of interest with regard to the review of this project, consistent with IRB policies and procedures, the member was required to recuse him/herself and, if applicable, leave the room during the discussion and vote on this project except to provide information requested by the IRB.

Partners Human Research
Partners HealthCare
399 Revolution Drive, Suite 710
Somerville, MA 02145
Tel: 857-282-1900
Fax: 857-282-5693

GENERAL REVIEW COMMENTS

This approval covers an amendment to update the consent form to reflect that the posttest assessments take 30- 45 minutes to complete and add that participants have the option to complete the post assessments over the phone with a study staff member or online via REDCap; and adding the Barthel Index and Modified Rankin Scale to be collected at all 3 time points by trained study staff.

As Principal Investigator, you are responsible for ensuring that this project is conducted in compliance with all applicable federal, state and local laws and regulations, institutional policies, and requirements of the IRB, which include, but are not limited to, the following:

1. Submission of any and all proposed changes to this project (e.g., protocol, recruitment materials, consent form, status of the study, etc.) to the IRB for review and approval prior to initiation of the change(s), except where necessary to eliminate apparent immediate hazards to the subject(s). Changes made to eliminate apparent immediate hazards to subjects must be reported to the IRB as an unanticipated problem.
2. Submission of a continuing review submission or institutional status report as required by the IRB and/or institution to continue the research, and submission of a final report when the project has been closed or completed.
3. Submission of any and all unanticipated problems, including adverse event(s) in accordance with the IRB's policy on reporting unanticipated problems including adverse events.
4. Obtaining informed consent from subjects or their legally authorized representative prior to initiation of research procedures when and as required by the IRB and, when applicable, documenting informed consent current IRB approved consent form(s) with the IRB-approval stamp in the document footer.
5. Informing all investigators and study staff listed on the project of changes and unanticipated problems, including adverse events, involving risks to subjects or others.
6. When investigator financial disclosure forms are required, submitting updated financial disclosure forms for yourself and for informing all site responsible investigators, co-investigators and any other members of the study staff identified by you as being responsible for the design, conduct, or reporting of this research study of their obligation to submit updated Investigator Financial Disclosure Forms for this protocol to the IRB if (a) they have acquired new financial interests related to the study and/or (b) any of their previously reported financial interests related to the study have changed.

IMPORTANT REMINDER: THE IRB HAS THE AUTHORITY TO TERMINATE PROJECTS THAT ARE NOT IN COMPLIANCE WITH THESE REQUIREMENTS.

Questions related to this project may be directed to **Ednice, Monteiro | Tel: 282-1916 | Email: EEMONTEIRO@PARTNERS.ORG**

cc:

Ana-Maria, Vranceanu, , Psychiatry, Psychiatry, Principal Investigator

Partners Human Research
Partners HealthCare
399 Revolution Drive, Suite 710
Somerville, MA 02145
Tel: 857-282-1900
Fax: 857-282-5693

Sofia, Distefano, BS, Neurology, Neurology, Research Coordinator/Manager

Ann, Lin, , Psychiatry, Psychiatry, Research Coordinator/Manager

Melissa, Gates, , Psychiatry, Psychiatry, Research Coordinator/Manager

Partners HealthCare System
Research Consent Form

Certificate of Confidentiality Template
Version Date: January 2018

Subject Identification

Protocol Title: Recovering Together: Building resiliency in dyads in patients admitted to the Neuroscience Intensive Care Unit (NICU) and their caregivers

Principal Investigator: Ana-Maria Vranceanu, PhD

Site Principal Investigator: N/A

Description of Subject Population: Acute neurological illnesses (ANIs) patient - caregiver dyads admitted to the NICU

About this consent form

Please read this form carefully. It tells you important information about a research study. A member of our research team will also talk to you about taking part in this research study. People who agree to take part in research studies are called "subjects." This term will be used throughout this consent form.

Partners HealthCare System is made up of Partners hospitals, health care providers, and researchers. In the rest of this consent form, we refer to the Partners system simply as "Partners."

If you have any questions about the research or about this form, please ask us. Taking part in this research study is up to you. If you decide to take part in this research study, you must sign this form to show that you want to take part. We will give you a signed copy of this form to keep.

A description of this clinical trial will be available on *https://www.ClinicalTrials.gov*, as required by the U.S. Law. This website will not include information that can identify you. At most, the website will include a summary of the results. You can search this website at any time.

Why is this research study being done?

This research study is being done to understand the experiences of patients admitted to the neuroscience intensive care unit (Neuro-ICU) and their primary caregivers. The purpose of this study is to compare two dyadic (patient and caregiver) programs to improve emotional distress in patients and caregivers.

We are asking you to take part in this research study because you are at least 18 years of age, an English-speaking patient with an acute neurological illness (ANI) admitted to the intensive care unit, or a caregiver of a patient with an ANI.

About 80 dyads (160 people) will take part in this research study.

This research study is being conducted by the Neuroscience Intensive Care Unit at Massachusetts General Hospital. The National Institute of Nursing Research is paying for this study to be done.

How long will I take part in this research study?

It will take you about 4-5 months to complete this research study.

What will happen in this research study?

The training program we are testing was developed based on information from the nursing care team and patients and caregivers like yourself. It has 6 sessions and it teaches behavioral, and psychosocial skills. Two sessions will take place during hospitalization and 4 sessions will take place through a secure, live-videoconferencing program called Vidyo.

If you choose to participate in this study, we will ask you to sign this consent form before we do any study procedures.

Once you sign this consent form and agree to participate in this study, you and your partner will be randomly assigned together to one of two groups. You will be assigned by chance (like flipping a coin) to one of the two dyadic training programs. There is no way to predict which of the two groups you and your partner you will be in. Both groups will participate in a dyadic program with a study therapist and a program manual. We do not know right now which intervention will be more effective. You will not know what the other intervention is.

Below is an outline of the study schedule:

Study Intake (1/2 hour)

During this portion of the study, you will fill out several questionnaires online or in person, through a secure system. The survey questions will ask you about your stress levels, mental health, behavior, medical symptoms, and quality of life. You can complete these questionnaires in-person during your intake visit, on paper at home, or on a personal computer at home.

For your safety, we will also ask you to provide contact information for a family member or friend that we may contact on your behalf in case of emergency.

Weekly Sessions 1-6

The intervention will have 6 sessions with 2 general sessions delivered in-person within the Neuro-ICU and 4 tailored specific sessions to be delivered via live video using Vidyo, when you return home or at rehab. Vidyo is a HIPAA approved, secure online videoconferencing software program. We will help you set up for Vidyo sessions. We will help you install Vidyo on your computer, tablet or smartphone and will teach you how to use it before we start the groups. Once you log in from a webcam-equipped computer, the Vidyo program will allow you to see and hear the entire group in real-time, while participating from your home or another independent location. Study staff will schedule one brief Vidyo meeting with you to ensure that you are comfortable with using the software.

Post Program Assessment (30 - 45 minutes)

This portion of the study will occur after you have completed the 6-week program. You will fill out several questionnaires. The survey questions will ask you about your stress levels, mental health, behavior, medical symptoms and quality of life. You will have the option to complete these on a personal computer at home or over the phone with a member of our study staff team.

Follow-Up Assessments (30 - 45 minutes)

This portion of the study will occur 3 months after you have completed the 6-week program. You will fill out several questionnaires. The survey questions will ask you about your stress levels, mental health, behavior, medical symptoms and quality of life. You will have the option to complete these questionnaires on a computer from home or over the phone with a member of our study staff team.

Place of Visits: You can attend the online group sessions from your home or any other private place with a personal computer. The personal computer must be equipped with a webcam and Vidyo videoconferencing software.

Confidentiality: Your research study information will remain confidential, stored without identifying information, and be accessible only to study staff. Confidentiality will only be suspended in the case of a psychological emergency. In the unlikely event that a participant is determined to be actively suicidal and at risk for self-harm during any study procedures, the research assistant will contact the Principal Investigator (Vranceanu) and appropriate clinical intervention will be executed. Dr. Vranceanu may start a psychiatric consult depending on the severity of the situation.

OPTION TO CHOOSE:

The study investigator may wish to re-contact you in the future about related research studies. Do you agree to let us contact you in the future?
YES: ________ NO: _________

Reminders via Text Message

With your permission, we would like to send you text messages to remind you of specific assignments related to the treatment and physical activity encouragements. The Partners standard is to send secure text messages. If you prefer, we can send you “unencrypted” texts that are not secure and could result in the unauthorized use or disclosure of your information. If you want to receive communication by unencrypted texts despite these risks, Partners Healthcare will not be held responsible. Text message and data rates may apply based on your cell phone service plan. Your preference to receive unencrypted texts will apply to text messages sent to you from research staff in this study. If you wish to communicate with other research staff at Partners regarding additional studies, your preference will have to be documented with each research study.
These texts will not contain any protected health information. You have the right to refuse these texting reminders.
Your decision will not impact your ability to participate in the study. You can opt out of these text messages at any time.

Please select one of the following options:

I consent to text message reminders (please use your initials to indicate your response):

Yes: _________ No: _________

What are the risks and possible discomforts from being in this research study?

There are no foreseeable physical risks from this research study. Responding to questions about your recent feelings, emotions, and thoughts may cause you to feel discomfort. If you experience any of these symptoms, you may choose not to answer any question that makes you feel uncomfortable. You may also find it time consuming to participate in the 6 visits.

What are the possible benefits from being in this research study?

You will not benefit from this study directly. You may enjoy the opportunity to talk about your ANI experience and to share your story. However, knowledge from this research study may benefit others by enhancing our understanding of the role of skills-based interventions in treating future ANI patients or caregivers of ANI patients.

What other treatments or procedures are available for my condition?

The program offered in this research study does not constitute individualized, personal care. These interventions are broad-based training methods that are not tailored to any individual. If you would like formal mental healthcare or personalized instruction in mind body methods, we can give you a referral for psychological treatment that is suitable for you. For example, you may seek psychotherapy or medications outside of this research study or you may participate in other research studies for which you may qualify.

Participation in this research study does not mean that you cannot seek other forms of treatment for psychological distress, including medications or other forms of psychotherapy. In fact, we ask that you continue your regular medical treatment with your physician in addition to taking part in this research study.

Can I still get medical care within Partners if I don’t take part in this research study, or if I stop taking part?

Yes. Your decision won’t change the medical care you get within Partners now or in the future. There will be no penalty, and you won’t lose any benefits you receive now or have a right to receive.

Taking part in this research study is up to you. You can decide not to take part. If you decide to take part now, you can change your mind and drop out later. We will tell you if we learn new information that could make you change your mind about taking part in this research study.

What should I do if I want to stop taking part in the study?

If you take part in this research study, and want to drop out, you should tell us. We will make sure that you stop the study safely. We will also talk to you about follow-up care, if needed.

Also, it is possible that we will have to ask you to drop out of the study before you finish it. If this happens, we will tell you why. We will also help arrange other care for you, if needed.

Will I be paid to take part in this research study?

All participants will receive $20 for the completion of each of the 3 assessment points. Participants can receive up to $60 for study participation.

What will I have to pay for if I take part in this research study?

There will be no cost to you for any study visits. All of the group sessions and study assessments will be paid for by study funds. However, you will be required to have access to a personal computer equipped with a webcam or other video/audio capture device. The study will not provide you with such a computer. In addition, you will be required to download and install Vidyo videoconferencing software in order to participate in the online group sessions. This software is available for a free download, and study staff will give you specific instruction on how to locate and install it onto your computer.

What happens if I am injured as a result of taking part in this research study?

We will offer you the care needed to treat any injury that directly results from taking part in this research study. We reserve the right to bill your insurance company or other third parties, if appropriate, for the care you get for the injury. We will try to have these costs paid for, but you may be responsible for some of them. For example, if the care is billed to your insurer, you will be responsible for payment of any deductibles and co-payments required by your insurer.

Injuries sometimes happen in research even when no one is at fault. There are no plans to pay you or give you other compensation for an injury, should one occur. However, you are not giving up any of your legal rights by signing this form.

If you think you have been injured or have experienced a medical problem as a result of taking part in this research study, tell the person in charge of this study as soon as possible. The researcher's name and phone number are listed in the next section of this consent form.

If I have questions or concerns about this research study, whom can I call?

You can call us with your questions or concerns. Our telephone numbers are listed below. Ask questions as often as you want.

Ana-Maria Vranceanu, PhD, is the person in charge of this research study. You can call her at 617-724-4977, Monday through Friday, 9:00 am to 5:00 pm. You can also call Dr. Jonathan Rosand, MD, at 617-724-2698, Melissa Gates, at 617-643-9406, or Ann Lin, at 617-724-0051, Monday through Friday, 9:00 am to 5:00 pm, with questions about this research study.

If you have questions about the scheduling of appointments or study visits, please call Melissa Gates, at 617-643-9406, or Ann Lin, at 617-724-0051.

If you want to speak with someone **not** directly involved in this research study, please contact the Partners Human Research Committee office. You can call them at 857-282-1900.

You can talk to them about:

- Your rights as a research subject
- Your concerns about the research
- A complaint about the research

Also, if you feel pressured to take part in this research study, or to continue with it, they want to know and can help.

If I take part in this research study, how will you protect my privacy?

Federal law requires Partners to protect the privacy of health information and related information that identifies you. We refer to this information as “identifiable information.”

In this study, we may collect identifiable information about you from:

- Past, present, and future medical records
- Research procedures, including research office visits, tests, interviews, and questionnaires

Who may see, use, and share your identifiable information and why:

- Partners researchers and staff involved in this study
- The sponsor(s) of the study, and people or groups it hires to help perform this research or to audit the research
- Other researchers and medical centers that are part of this study
- The Partners ethics board or an ethics board outside Partners that oversees the research
- A group that oversees the data (study information) and safety of this study
- Non-research staff within Partners who need identifiable information to do their jobs, such as for treatment, payment (billing), or hospital operations (such as assessing the quality of care or research)
- People or groups that we hire to do certain work for us, such as data storage companies, accreditors, insurers, and lawyers
- Federal agencies (such as the U.S. Department of Health and Human Services (DHHS) and agencies within DHHS like the Food and Drug Administration, the National Institutes of Health, and the Office for Human Research Protections) state agencies, and foreign government bodies that oversee, evaluate, and audit research, which may include inspection of your records
- Public health and safety authorities, if we learn information that could mean harm to you or others (such as to make required reports about communicable diseases or about child or elder abuse)
- Other researchers within or outside Partners, for use in other research as allowed by law.

Certificate of Confidentiality

A federal Certificate of Confidentiality (Certificate) has been issued for this research to add special protection for information and specimens that may identify you. With a Certificate, unless you give permission (such as in this form) and except as described above, the researchers are not allowed to share your identifiable information or identifiable specimens, including for a court order or subpoena.

Certain information from the research will be put into your medical record and will not be covered by the Certificate. This includes records of medical tests or procedures done at the hospitals and clinics, and information that treating health care providers may need to care for you. Please ask your study doctor if you have any questions about what information will be included in your medical record. Other researchers receiving your identifiable information or specimens are expected to comply with the privacy protections of the Certificate. The Certificate does not stop you from voluntarily releasing information about yourself or your participation in this study.

Even with these measures to protect your privacy, once your identifiable information is shared outside Partners, we cannot control all the ways that others use or share it and cannot promise that it will remain completely private.

Because research is an ongoing process, we cannot give you an exact date when we will either destroy or stop using or sharing your identifiable information. Your permission to use and share your information does not expire.

The results of this research may be published in a medical book or journal, or used to teach others. However, your name or other identifiable information **will not** be used for these purposes without your specific permission.

Your Privacy Rights

You have the right **not** to sign this form that allows us to use and share your identifiable information for research; however, if you don't sign it, you can't take part in this research study.

You have the right to withdraw your permission for us to use or share your identifiable information for this research study. If you want to withdraw your permission, you must notify the person in charge of this research study in writing. Once permission is withdrawn, you cannot continue to take part in the study.

If you withdraw your permission, we will not be able to take back information that has already been used or shared with others, and such information may continue to be used for certain purposes, such as to comply with law or maintain the reliability of the study.

You have the right to see and get a copy of your identifiable information that is used or shared for treatment or for payment. To ask for this information, please contact the person in charge of this research study. You may only get such information after the research is finished.

Subject Identification

Informed Consent and Authorization

Statement of Person Giving Informed Consent and Authorization

- I have read this consent form.
- This research study has been explained to me, including risks and possible benefits (if any), other possible treatments or procedures, and other important things about the study.
- I have had the opportunity to ask questions.
- I understand the information given to me.

Signature of Subject:

I give my consent to take part in this research study and agree to allow my identifiable information to be used and shared as described above.

_______________________	_______________	_______________
Subject	Date	Time (optional)

Signature of Study Doctor or Person Obtaining Consent:

Statement of Study Doctor or Person Obtaining Consent

- I have explained the research to the study subject.
- I have answered all questions about this research study to the best of my ability.

_______________________	_______________	_______________
Study Doctor or Person Obtaining Consent	Date	Time (optional)

Consent Form Version Date: 2/26/2019

PARTNERS HUMAN RESEARCH COMMITTEE DETAILED PROTOCOL

Principal Investigator: Ana-Maria Vranceanu, PhD

Protocol Title: Recovering Together: Building resiliency in dyads in patients admitted to the Neuroscience Intensive Care Unit (NICU) and their caregivers

Funding: National Institute of Nursing Research

Version Date: 2/26/2019

I. BACKGROUND AND SIGNIFICANCE

Acute neurological illnesses (ANIs) are common, costly and often lead to long-term disability. ANIs are biologically distinct injuries that disrupt the normal function of the brain. The most common ANIs in Neuroscience Intensive Care Units (NICU) include cerebrovascular (stroke/hemorrhage and brain aneurysm), structural (tumors and lesions/brain masses), and traumatic (TBI) brain injuries. NICU admissions for ANIs are prevalent (e.g., 795,000 acute stroke/year; 275,000 acute TBI/year) and costly[1,2]; post NICU prolonged rehabilitation is common[3].

ANIs are associated with chronic emotional distress in both patients (pts) and caregivers (cgs). Although biologically heterogeneous, ANIs are unified by sudden onset, and substantial emotional distress in both pts (e.g., 12-43% anxiety[4-7]; 10-58% depression[4,7,8]; 20-29% post-traumatic stress PTS[5,9]) and family cgs (27-60% depression, anxiety or PTS [4,10,11]).
These symptoms often become chronic and treatment resistant[26,27].

Pt and cg factors interact and influence physical and emotional outcomes in both pts and cgs. Post ANI emotional distress is associated with pts' poor medical adherence[28], slower recovery[28-30], higher mortality[29-31], and need of more caregiving assistance[32], which further increase cgs' distress[30,33,34] and own risk for morbidity[35,36] and mortality[37]; in turn cgs' emotional distress interferes with ability to provide high-quality care to pts[38,39] and negatively impacts pts' outcomes.

Current management of ANIs does not meet the psychological needs of pts and cgs for 3 reasons[40-51]. First, although recognition of the emotional burden associated with NICU admission has increased, and some NICUs have social workers available to assist pts and cgs, there are no formal screening methods for emotional distress routinely integrated in practice during hospitalization, when the primary focus is on medical care and survival; further, there are no formal evidence-based treatments integrated within the medical care. When social workers are included to help pts and cgs, the care is brief and occurs only during hospitalization. When referrals to mental health services are provided to families at discharge, few will access additional treatment due to burden associated with traveling outside of home. Second, psychosocial interventions available for ANI pts or cgs are limited in that they are delivered when symptoms are already chronic, address only one emotional illness (e.g., depression or anxiety or PTS), and/or are focused on a *single member* of the pt-cg dyad. Even interventions labeled as "dyadic," which include pts and cgs, typically address only the pts' needs and do not

focus on cg outcomes or on the dyad's interpersonal communication and bond/relationship[12,52]. These interventions are not consistent with the *dyadic framework*[22] which specifies that dyadic interventions should account for the interdependence between pt and cg psychosocial factors including their interpersonal bond by ensuring that both pts and cgs attend each session together, and by targeting improvement in outcomes for both pts and cgs. Third, most interventions are delivered using uniform protocols. However, the needs of ANI dyads are heterogeneous due to varying levels of post ANI impairment, identity of the cg, context and stage of life. A recent systematic review[52] urged for the development of dyadic interventions that address the needs of *both* pts and cgs and are tailored to the specific needs of each ANI dyad.

We developed the first dyadic skills-based intervention – Recovering Together - to prevent chronic heightened emotional distress in at risk ANI pt-cg dyads. The "Recovering Together" program is informed by the theoretical response-shift framework of adaptation to acute illness[53] (successful adaptation implies recalibration of values and life goals), the family strength vulnerability model[24] (within dyads relational systems have strengths and weaknesses in how they cope with life events), the dyadic longitudinal model[22] (distress travel from one member of the dyad to the other across time), the APIM model[54], and the resiliency framework[17]**.** The program is in line with recent recommendations for skill-based interventions for critical care patients, and uses preliminary data collected by our team for the past 3 years[4, 13-15]. The intervention teaches pts and cgs resiliency factors that are associated with well-being after trauma for both pts and cgs: *mindfulness* – the ability to stay present and defer judgment in the face of adversity[18]; *coping*– the arsenal and application of one's behavioral, cognitive, and emotional strategies to manage stress[19]; *social support* –empathetic interpersonal interactions that meet one's emotional and functional needs[20]; *self-efficacy* – perceived ability to adapt under adversity[21] and *positive dyadic interpersonal communication to increase interpersonal bond*[12]. Informed by the aforementioned theoretical models, our conceptual model hypothesizes that by teaching both members of the dyad resiliency and interpersonal communication skills (e.g., Recovering Together) we will be able to sustainably decrease emotional distress in both members of the dyad

In addition, our team has an established record of collaboration on published or ongoing investigations. **Emotional distress is prevalent in dyads, interdependent between pt and cg, and negatively associated with resiliency factors[4].** Our team conducted a cross-sectional study of pt-cg dyads in the NICU (40% stroke, 30% tumor). 75% pts and 84% cgs approached agreed to participate. 74% pts had been intubated at one time during NICU admission, and 2/3 were discharged home. Rates of clinically significant symptoms of depression, anxiety and PTS did not differ between pts (24%, 43%, 21%) and cgs (24%, 46%, 17%), or by any demographic or medical characteristic. Dyadic modeling showed that for both pts and cgs, mindfulness and coping impacted both self and partner's emotional distress symptoms. We showed: 1) feasibility of recruitment; 2) high emotional distress in dyads; and that 3) modifiable resiliency factors (mindfulness and coping) are intervention targets interdependently associated with distress in pts and cgs, regardless of the identity of cg (e.g., spouse, friend, etc).

Resiliency factors are associated with lower emotional distress in NICU dyads[13]. Our team found that resiliency factors of mindfulness, coping, self-efficacy and patient-caregiver interactions were associated with decreased emotional distress in dyads of ANI. This study confirms mindfulness, coping as intervention targets and provides novel evidence on self-efficacy and patient-caregiver interaction as additional important intervention targets.

ANI pts have greater anxiety than cancer patients at early diagnoses[14]. We led the first cross-comparison study of emotional distress among dyads with ANI and cancer. This study supports the priority of addressing emotional distress in ANI dyads as has been emphasized for cancer dyads.

Clinically significant emotional distress in one member of the dyad at hospitalization predicts chronic emotional distress in at least 1 of the dyad members 3 and 6 months later[55]. Our team has an ongoing prospective study of dyads with ANI. Retention rates for dyads due for assessments at 3 and 6 months thus far are 84% and 91% for pts and 87.7% and 95.7% for cgs, confirming our ability to retain post-ANI participants. Within each dyad, if one member screens in for clinically significant symptoms for any diagnosis (i.e., depression, anxiety or PTSD) at hospitalization there is good sensitivity and specificity that one member of the dyad will endorse clinically significant symptoms 3 months later. This study shows a reliable method for identifying dyads of patients at risk for chronic heightened emotional distress by identifying dyads in which either the pt or cg screens in for heightened emotional distress (symptoms of depression, anxiety or PTSD).

Caregiver gender moderates the prospective association of resiliency factors to emotional distress[87]. This study found that at the time of admission resiliency factors have main effects on emotional distress, with no differences by cg gender. However, significant interaction effects emerged prospectively such that male cgs with high mindfulness at baseline demonstrated lower levels of emotional distress at 3 and 6 months later than did males with low mindfulness ($p = 0.026$ and $p <0.013$). Similarly, women cgs with high intimate bond at baseline reported the lowest levels of depression symptoms 3 and 6 months later ($p < 0.020$). This study confirmed the need to assess and address resiliency factors early in the recovery process, and identified important gender differences to be accounted for in intervention development.

Recovering Together; Developing a novel dyadic resiliency skills program for ANI pt-cg dyads at risk for chronic emotional distress[15]. With funding from American Heart Association, we conducted 20 qualitative interviews with pt-cg stroke dyads at risk for chronic emotional distress during NICU hospitalization. We also conducted additional clinical interviews with 10 pt-cg dyads representative of other ANI diagnoses. 83% dyads approached agreed to participate. Pts (23) and cgs (25) were mostly women. Dyads were mostly spouses and mothers-daughter. Data was analyzed with Nvivo10. Main themes did not differ by medical diagnoses: 1) most challenging and distressing experiences: uncertainty about future, anxiety, depression, sleep difficulties, worries about the future, guilt, managing job with caretaking, making treatment decisions, lack of predictability; 2) concerns about interpersonal relationships (self-image, role changes, role fulfillment); 3) fear of recurrence; 4) adjusting to sequelae. Dyads noted interest in a resiliency program (30/30) and preferred a combination of in person and live video sessions (30/30). Dyads learned about resiliency skills (e.g., name, description and goal) and agreed they would be helpful to them. They also noted interest in learning about: survivorship plans, adaptation to deficits (present or anticipated), and return to normal living. We found no thematic differences between stroke dyads and other ANI dyads. Challenges associated with embracing the caregiver role emerged as a theme while differences by the identity of caregiver (e.g., spouse vs. friend, vs parent) did not. Themes associated with the gender of the cg emerged and have been incorporated in the intervention.

Nurses perception of the needs for and feasibility of "Recovering Together for ANI families[25]. We conducted 2 focus groups (N =15) with NICU nurses who provided feedback on the qualitative findings from our 30 ANI dyads and shared own experiences and opinions on

implementation and scalability of the intervention including nurse involvement. Nurses concurred with pts experiences, and suggested strategies to recruit and retain dyads for the study, which are now included in the methodology section of the current grant proposal. Studies 3.6 and 3.7 represent the building blocks for the development of our Recovering Together program and manual. Nurses provided edits and contributed to the iterative development of the manual. These studies also confirm feasibility of conducting the pilot RCT proposed through this R21.

II. SPECIFIC AIMS

The current study has the following objectives:

Aim 1: **To determine the feasibility of recruitment, feasibility of program delivery, program credibility, and program satisfaction using evidence-based benchmarks.**

Hypothesis 1: We hypothesize that > 75% of the dyads approached will agree to participate.
Hypothesis 2: We hypothesize that > 75% of dyads who start the intervention will complete at least 4 sessions.
Hypothesis 3: We hypothesize that > 75% of participants will report average credibility (Credibility and Expectancy Questionnaire) scores greater than the scale's midpoint.
Hypothesis 4: We hypothesize that > 75% participants will report average satisfaction (Client Satisfaction Scale) scores greater than the scale's midpoint.

Aim 2: **To demonstrate a proof of concept that the Recovering Together program can sustainably improve emotional distress [Hospital Anxiety and Depression Scale; HADS], Post Traumatic Symptoms (PTS) [PCL-S], resiliency variables (mindfulness, coping, social support and self-efficacy) and interpersonal factors (interpersonal bond).**

Hypothesis 1: We hypothesize that participation in the Recovering Together Program will be associated with a more potent decrease in emotional distress and PTS compared to participation in the educational program (control), and that these improvements will maintain at 3 month follow up.
Hypothesis 2: We hypothesize that Recovering Together Program will be associated with a more potent increase in resiliency variables (mindfulness, coping, social support, self-efficacy) and interpersonal factors (interpersonal bond) compared to participation in the educational program, and that these improvements will maintain at 3 month follow up.

For this feasibility pilot RCT, our primary outcomes (feasibility, credibility and satisfaction) will be assessed in Aim 1. Our secondary outcomes in this trial are: emotional distress, PTS, mindfulness, coping, social support, self-efficacy, and interpersonal bond, and will be assessed in Aim 2.

III. SUBJECT SELECTION

All participants will be recruited from the Massachusetts General Hospital Neuroscience ICU, using IRB approved recruitment materials.

Inclusion/Exclusion Criteria

Eligible dyads (Pts and Cgs) must meet the following inclusion criteria:

1) Age 18 or older
2) English fluency and literacy
3) Access to high speed internet for video sessions
4) Pt with an informal cg (family or friend who provides unpaid care) available and willing to participate
5) Hospitalized with an ANI within 1-2 weeks (pt) OR primary cg of a pt currently admitted with an ANI
6) Either pt <u>or</u> cg have clinically significant symptoms of depression, anxiety, and/or PTS

One or more of the following exclusion criteria will render a pt ineligible:

1) Permanent and severe cognitive impairment severe enough to impede participation – This will be determined by trained study staff through an assessment conducted as part of usual care and that includes the MMSE (score of <23) and GCS (score of <10). Nurses and study staff are trained and use these measures as part of NICU care.
2) Dyads where the pt is anticipated to die or to never be able to participate due to medical sequelae. This will be determined by nurses.

All adult patients and family caregivers, satisfying all inclusion criteria, are eligible for enrollment in this study regardless of sex, race, or ethnicity. Vulnerable populations will not be recruited.

Recruitment

Patients will be recruited from the NICU at MGH from the medical team (nurses). Recruitment will be facilitated by the nursing team who will introduce the study to eligible dyads and who will also assess whether pts are able to consent, consistent with medical presentation, and cognitive status. Trained study staff will be administering the MMSE and nurses will administer the GSC, to all patients and will refer only dyads where the patients scores are higher than the established cut off scores on these measures. Consistent with NICU practice, nurses will administer the GSC. They will refer participants to the study only if they are cleared medically and cognitively. The team will page the RA using the secure Voalte system used by our team and nursing staff, when both the pt and cg are present and able to hear more about the study. The RA will only approach pts identified and cleared by the medical team. The RA will ensure eligibility of both pts and cgs based on the additional inclusionary and exclusionary criteria depicted above. The RA will finalize the screening and conduct informed consent**.** All participants will receive a physical copy of the informed consent form which has the contact information for the PI. If cg~~s~~ cannot be reached in person in the hospital, they will be contacted via phone for screening and completion of informed consent. Cgs will be given a copy of the consent form to review while discussing the study over the telephone. We will fax or securely e-mail the consent form prior to obtaining consent. Cg will return a signed copy of the consent form.

Patients and caregivers will be considered enrolled when they sign the consent form with the study staff. Please see details on the informed consent process in the next section, Subject Enrollment (Section IV). These procedures will be completed in a private medical space.

Participants will be explicitly informed that this intervention is a research study that does not constitute individualized, personal care. The intervention is a broad-based method of training that is not tailored to any particular individual. Should any participant seek formal mental healthcare, study staff will refer them to either MGH Psychiatry, as appropriate.

We will recruit pts with any type of ANI and their cgs, ensuring representation of all NICU diagnoses. All cgs will be recruited within the first week of the pts' hospitalization. Pt's medical team will also alert the research assistant whether they anticipate that the patient might be able to participate in the study at a future point during the hospital stay. In situations where patients are unable to consent due to the severity of the ANI, we will enroll the informal cg and return to enroll the pt as soon as their mental capacity improves. The research assistant will not approach patients who are not mentally or physically capable to participate. This study does not include participants with impaired decision making.

We will not include patients who do not have a caregiver. Informal caregivers will be designated by the health care proxy and verbally confirmed by the patient. Nurses will assist the study team with identifying the patient's health care proxy.

Eligible cases may also be identified by daily screening of Epic admission reports.

The study team will keep track of all pts and cgs approached who refuse to participate (along with reasons for refusal), as well as those who were not approached and reasons why.

When the RA approaches a patient, patients and caregivers will be given the choice of watching a short recruitment video that contains testimonials from previous patients.

The recruitment process was developed and refined through prior research for the past 2 years.

IV. SUBJECT ENROLLMENT

Participants will be referred to the study by the nursing team who will ensure that patients are medically and cognitively able to participate. Eligible dyads will next be screened, consented, and enrolled by the research assistant.

After determining eligibility, study staff will meet with potential dyads to review the informed consent document. If cgs cannot be reached in person in the hospital, they will be contacted via phone to complete informed consent. Cgs will be given a copy of the consent form to review while discussing the study over the telephone. We will fax or securely e-mail the consent form prior to obtaining consent. Cgs will return a signed copy of the consent form. After the document has been reviewed, study staff will answer any and all questions the pt or cg may have. Once all questions have been addressed, each member of the dyad will sign the consent form, which will

include a description of all study procedures, the option to receive text message reminders, information about potential risks and benefits of participation, and study contact information (including that of the IRB) in case questions arise at a later time. The consent form will also explicitly state that study participation is voluntary, and that participants may refuse to answer any questions that make them uncomfortable, and may discontinue participation at any time. In addition, participants will be assured that withdrawal from the study will not compromise their medical care in any way.

As informed consent is a continuous process, participants will be given a copy of the signed informed consent document, and will be invited to ask questions about their participation at any point over the course of the study.

Following a study enrollment and baseline assessment, dyads will be randomly assigned to either the newly developed psychosocial intervention or to the educational program (control) using a random number sequence generator to ensure comparability between groups. We will document time between assessment and intervention initiation, and analyze as a predictor of study outcomes as needed. Randomization will be developed by the statistician, without any input from the rest of the team.

V. STUDY PROCEDURES

After enrollment, participants will complete study assessments. All subjects will be given baseline psychological and behavioral assessments that will assess depression, anxiety, PTS symptoms, and other psychological constructs. Assessments will be administered online, using the REDCap system. Subjects may choose to fill out these questionnaires on-site, or to fill them out at home on a personal computer or other Internet-equipped device. This assessment includes demographic information and a battery of psychological questionnaires. We will also collect information about important clinical variables including duration of acute hospitalization. All questionnaires are itemized below:

Administered at Baseline Only:
Demographics
Prior Mental Health History questions
Credibility Questionnaire

Administered at Baseline, Post-Intervention, and Follow-up:
Medical history information from LMR (i.e., prior ANI status, current psychotropic meds, comorbid medical conditions, etc.)
Post-Traumatic Stress Disorder Checklist (PCL-S)[29]
Hospital Depression and Anxiety Scale (HADS)[30]
Measure of Current Status Part A (MOCS-A)[31]
The Cognitive and Affective Mindfulness Scale (CAMS)[32]
World Health Organization Quality of Life (WHOQOL-BREF)[33]
Dyadic Relationship Scale (DRS)
Experience in Close Relationships – Relationship Structure (ECR-RS)

Administered Post-Intervention and Follow-up Only:
Client Satisfaction Questionnaire (CSQ-3)[34]

The study staff will complete the following questionnaires to assess symptom severity for each patient at all 3 timepoints (baseline, post intervention and follow up):
Modified Rankin Scale (mRS)[35] [Pts only]
Barthel Index[36] [Pts only]

After completing the baseline assessments, dyads will be randomized to either the newly developed psychosocial intervention or to treatment as usual.

The psychosocial intervention entails 6 sessions, each 30 minutes. Both the pt and cg participate in each session. The intervention is manualized, and teaches dyads resiliency (mindfulness, social support, self-efficacy, coping skills) and interpersonal communications (interpersonal bond) skills. The first 2 sessions will occur in person, during hospitalization. The next 4 sessions are chosen from 5 available, depending on each dyad's preference, and are delivered after discharge via secure live video. For the video sessions, dyads can participate from the same or different locations. During the in hospital sessions, dyads learn diaphragmatic breathing, mindfulness, self-care, and dialectics. During the video sessions, dyads learn how to identify negative thinking patterns and replace them with adaptive thoughts, how to communicate effectively and openly, how to engage in self-care, and how to accept things that can't be changed. Participants in the intervention group will also receive treatment as usual. This may include meeting with nurses, physical therapist**s**, medical doctors, and other members of the pt's medical team. Treatment as usual may also involve administration of SSRI to those patients with motor problems.

Those in the educational program (control) will receive general health information that mimics the Recovering Together Program, but without teaching any of the resiliency or interpersonal communication skills that are hypothesized to be responsible for improvement in emotional distress. The educational program will also have 6 sessions, 2 in-person dyadic visits in the NICU and 4 dyadic virtual visits following discharge. It controls for dose and support from clinician. Both members of the dyads participate in all sessions. The topics of each session include: education about the stress of the ANI on patient and caregiver; education on the importance of self-care; education on stress associated with discharge and home adjustment; education on the importance of following up with medical recommendations; education on interpersonal stress as part of adjustment to ANI; education on self-care. The educational program condition will ensure that patients will remain blind to intervention or control and increase confidence that improvement in outcomes are due to the active ingredients of the intervention and not confounds. Participants in the educational program will continue with their current care. This may include meeting with nurses, physical therapist**s**, medical doctors, and other members of the pt's medical team. Treatment as usual may also involve administration of SSRI to those patients with motor problems.

Participants in both groups will be given post-treatment psychological and behavioral assessments identical to those administered at baseline, in addition to the CSQ-3. As a baseline, participants will be given the option to complete post-treatment questionnaires on site or at home. Participants will be asked to complete questionnaires immediately after completion of the group intervention (T2), and 3 months (T3) after completion of the group intervention, in order to measure long term outcomes. Study staff will email these questionnaires to the participants via the REDCap system or give participants the option to complete them over the phone with a study staff member.

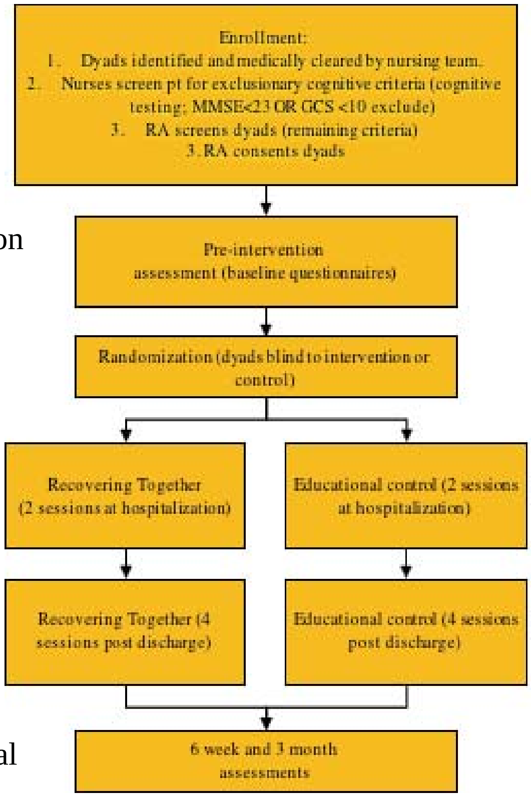

VI. BIOSTATISTIC ANALYSIS

We plan to conduct a RCT with patients from the Neuro-ICU. The main intervention goal is to provide dyads with resiliency and interpersonal communication skills necessary to optimize recovery. Dyads will be medically cleared by a member of the nursing staff. Nurses will then screen dyads for exclusionary cognitive criteria. Dyads will complete additional screening and baseline questionnaires with a trained research assistant. Dyads will be randomized to one of two groups 1) Psychosocial skills based intervention (Recovering Together), or 2) Educational Control.

We chose our study measures based on our theoretical frameworks, strong psychometric properties in studies of patients with ANI, and feasibility in our prior work with ANI dyads. Measures will be collected at baseline, post-intervention, and at 3 month follow up. Data collection and management will be conducted with Research Electronic Data Capture. Dyads will be given the option to complete measures electronically, by paper and pencil, or telephone.

1) Is the psychosocial skills-based intervention feasible, credible usable, and accepted by pts and cgs in the ICU?

Our primary aim focuses on trial feasibility, acceptability, credibility and preliminary effect. In pilot studies, ≥30 participants are recommended per group to establish feasibility and detect larger effect sizes for ≥1 outcome. We plan to recruit 80 dyads (160 participants), 40 dyads (80 participants) per arm to establish feasibility, acceptability, credibility and estimate effect size for emotional distress variables (primary quantitative outcomes). Assuming attrition of over 25% (in excess of what we experienced in our preliminary studies), we will have the necessary 30 dyads (60 participants) per arm. This size is considered to yield stable estimates of M/SDs based on prior behavioral trial recommendations. Effect sizes from this study may overestimate power in future sample calculations. We chose study measures based on our theoretical frameworks, strong psychometric properties in studies of patients with ANI, and feasibility work in our prior

work with ANI dyads. Feasibility will be reported as the percentage of patients enrolled in the study who complete at least 75% of the intervention sessions. Demonstration of feasibility will be assessed by the number of individuals who drop out of the study prior to completing the post- intervention assessment and the rate of missed sessions. If drop-out rate or missed-session rate exceeds 25%, revisions to the intervention may be needed. We will also report number of patients approached, enrolled, randomized and who completed time 2 and time 3 to determine feasibility.

2) Is the psychosocial skills-based intervention effective for pts and cgs in the ICU?

Dr. Vranceanu developed the proposed study design in collaboration with the NICU study team (nurses, physicians, clinical interns) and an MGH psychologist who specializes in using mindfulness and emotion regulation in both chronic illness and medically healthy populations. With funding from the American Heart Association, we conducted 20 qualitative interviews with pt-cg stroke dyads at risk for chronic emotional distress during NICU hospitalization. We also conducted additional clinical interviews with 10 pt-cg dyads representative of other ANI diagnoses. 83% of dyads approached agreed to participate. Pts (23) and cgs (25) were mostly women. Dyads were mostly spouses and mothers-daughter. Dyads noted interest in a resiliency program (30/30) and preferred a combination of in person and live video sessions (30/30). Dyads learned about resiliency skills (name, description, and goal) and agreed they would be helpful to them. They also noted interest in learning about: survivorship plans, adaptation to deficits (present or anticipated), and return to normal living. This led to the development and subsequent refinement (through feedback from the nursing team) of the Recovering Together Program active intervention.

3) Is the effect of the skills-based intervention for pts and cgs in the ICU durable?

We will assess feasibility, usability, and acceptability by the enrollment numbers, participants completion in at least 4 out of 6 sessions, and the questionnaires, The Credibility and Expectancy and Client Satisfaction Scale. These will be primary outcomes. The PCL-S, HADS, and resiliency measures will serve as the secondary outcomes.

We will use student’s t-test to assess within-group differences in long-term outcomes. We will compare measures at the 3 month follow-up assessment to measures at the post-intervention assessment for both study arms.

We will also use t tests and chi squared tests to assess differences at the 3 month follow up.

VII. RISKS AND DISCOMFORTS

There is a risk that some participants may feel uncomfortable completing various psychological questionnaires or parts of the skills-based on intervention. Participants are free to withdraw from the study at any time, as the study is completely voluntary.

As in any research study, there is a small risk that confidentiality may be breached; all efforts to minimize this risk will be taken. In the unlikely event that participants will become suicidal during the duration of the study, the research assistant will contact the PI and the appropriate clinical intervention will be executed.

VIII. POTENTIAL BENEFITS

Participants in this study may observe a reduction in depression, anxiety, and/or psychological and physiological markers of stress, as well as an improvement in perceived qualify of life. It is hoped that the intervention will result in improvements across these domains. Participants may learn new techniques for managing distress and lifestyle factors that may enhance wellbeing, both in disease-specific domains as well as in their general lives. In addition, all participants will receive $20 for the completion of each of the 3 assessment points.

IX. MONITORING AND QUALITY ASSURANCE

Electronic information will be stored in REDCap (Research Electronic Data Capture), a free, secure, and HIPAA-compliant web-based application hosted by the Partners HealthCare Research Computing Enterprise Research Infrastructure & Services (ERIS) group (based at the PHS Needham corporate datacenter). Data will be stored on password protected computers that will be stored in secure locations at all times. Paper data files (with coded subject identification) will be stored in a locked filing cabinet. Only research staff will have access to these data locations.

A unique anonymous identifier will be assigned to each subject; subsequently, all data collected will be associated exclusively with this identifier. This includes all questionnaires administered over the course of the study, as well as home practice logs.

Data from this study will be stored for three years after the publication of all study results, at which time all paper data files will be shredded, and computer files will be deleted.

Data Management and Quality Control Procedures

To maximize accuracy and security, all survey data will be collected and stored on REDCap. Research staff will ensure that proper consent has been obtained before sending the REDCap survey to each participant.

REDCap (Research Electronic Data Capture) is a free, secure, HIPAA compliant web-based application hosted by the Partners HealthCare Research Computing Enterprise Research Infrastructure & Services (ERIS) group. Vanderbilt University, with collaboration from a consortium of academic and non-profit institutional partners, has developed this software toolset and workflow methodology for electronic collection and management of research and clinical study data. Data collection projects rely on a study-specific data dictionary defined by members of the research team with planning assistance from Harvard Catalyst, The Harvard Clinical and

Translational Science Center EDC Support Staff. This iterative development and testing process results in a well-planned data collection strategy for individual studies. Using REDCap, the research team can also design web-based surveys and engage potential respondents using a variety of notification methods. REDCap provides flexible features that can be used for a variety of research projects and provides an intuitive interface to enter data with real time validation (automated data type and range checks). The system offers easy data manipulation with audit trails, reports for monitoring and querying participant records, and an automated export mechanism to common statistical packages (SPSS, SAS, Stata, R/S-Plus).

Since consistency of application of the study protocol is critical to acquiring high quality data, all research personnel have undergone or will undergo a competency-based training program prior to enrolling subjects.

Data and Safety Monitoring Plan

Adverse Event Monitoring: Throughout the study subjects will be monitored for the occurrence of events defined as any undesirable experience or unanticipated risk. Lack of effect of treatment is not considered an event. All adverse events will be reported on an adverse event form. The Principle Investigator has the responsibility of reporting serious adverse events (death, life threatening illness or injury, serious injury, or permanent disability) to PHRC within 24-72 hours of notification.

X. REFERENCES

1. Center for Disease Control and Prevention. (2015). Stroke Facts: Stroke in the United States. *CDC.* Retrieved from http://www.cdc.gov/stroke/facts.htm.
2. Center for Disease Control and Prevention. (2015). *Report to Congress on Traumatic Brain Injury in the United States: Epidemiology and Rehabilitation.* National Center for Injury Prevention and Control; Division of Unintentional Injury Prevention. Atlanta, GA.
3. The Society of Critical Care Medicine. (2015). What is critical care? *SCCM*. Retrieved from www.myicucare.org.
4. Shaffer KM, Riklin E, Stagl JM, Rosand J, Vranceanu AM. (2016). Mindfulness and coping are inversely related to psychiatric symptoms in patients and informal caregivers in the Neuroscience ICU: Implications for Clinical Care. Critical *Care Medicine*, 44 2036-2038. PMID: 27513536
5. Jackson, J.C., Mitchell, N., & Hopkins, R.O. (2011). Cognitive functioning, mental health, and quality of life in ICU survivors: an overview. *Anesthesiology Clinics, 29,* 751-764. PMID: 22078921
6. Kress, J.P., Gehlbach, B., Lacy, M., Pliskin, N., Pohlman, A.S., & Hall, J.B. (2003). The long-term psychological effects of daily sedative interruption on critically ill patients. *American Journal of Respiratory and Critical Care Medicine*, *168,* 1457-1461. PMID: 14525802
7. Cheung, A.M., Tansey, C.M., Tomlinson, G., Diaz-Granados, N., Matté, A., Barr, A., ... Herridge, M.S. (2006). Two-year outcomes, health care use, and costs of survivors of acute respiratory distress syndrome. *American Journal of Respiratory and Critical Care Medicine, 174,* 538-544. PMID: 16763220
8. Daydow, D.S., Gifford, J.M., Desai, S.V., Bienvenu, O.J., & Needham, D.M. (2009). Depression in general intensive care unit survivors: a systematic review. *Intensive Care Medicine, 35,* 796-809. PMID: 19165464

9. Jackson, J.C., Hart, R.P., Gordon, S.M., Hopkins, R.O., Girard, T.D., & Ely, W.E. (2007). Post-traumatic stress disorder and post-traumatic stress symptoms following critical illness in medical intensive care unit patients: assessing the magnitude of the problem. *Critical Care, 11,* R27. PMCID: PMC2151890
10. Choi, J., Hoffman, J.A., Schulz, R., Ren, D., Donahoe, M.P., Given, B., & Sherwood, P.R. (2013). Health risk behaviors in family caregivers during patients' stay in intensive care units: a pilot analysis. *American Journal of Critical Care, 22,* 41-45. PMCID: PMC4109809
11. McAdam, J.L., Fontaine, D.K., White, D.B., Dracup, K.A., & Puntillo, K.A. (2012). Psychological symptoms of family members of high-risk intensive care unit patients. *American Journal of Critical Care, 21,* 386-393. PMID:23117902
12. McCarthy, M.J., Lyons, S.L., Powers, L.E. (2012). Relational factors associated with depressive symptoms among stroke survivor-spouse dyads. *Journal of Family Social Work* 15: 303-320.
13. Shaffer KM, Riklin E, Stagl JM, Rosand J, Vranceanu AM. (2016) Psychosocial resiliency is associated with lower psychological distress among dyads of patients and their informal caregivers in the neuroscience intensive care unit. J Critical Care 2016 Jul 16; 36:154-159. PMID: 27546765
14. Schaffer KM, Jacobs JM, Coleman JN, Rosand J, Temel, J, Greer JA. Vranceanu AM**.** Anxiety and depressive symptoms among two seriously ill medical populations and their family caregivers. Neurocritical Care 2016; ahead of print.
15. Zale EL, Piere-Louis C, Tehan T, Henhuis T, Rosand J., Vranceanu, A.M. Improving resiliency and brain health after acute neurological illness; Perceptions of patients and families. Accepted for presentation at Brain Health Conference, Columbus, OH April 28-30, 2017.
16. Martire LM, Lustig AP, Schulz R, Miller GE, Helgeson VS. Is it beneficial to involve a family member? A meta-analysis of psychosocial interventions for chronic illness. *Health Psychol* 2004; 23(6):599-611
17. Bonanno, G. A., Galea, S., Bucciarelli, A., & Vlahov, D. (2007). What predicts psychological resilience after disaster? The role of demographics, resources, and life stress. *Journal of Consulting and Clinical Psychology*, *75*,671–682. http://dx.doi.org/10.1037/0022-006X.75.5.671
18. Brown KW, Ryan RM (2003) The benefits of being present: mindfulness and its role in psychological well-being.J Pers Soc Psychol 84(4):822
19. Donnellan C, Hevey D, Hickey A, O'Neil D (2006) Defining and quantifying coping strategies after stroke: a review. J Neurol Neurosurg Psychiatry 77:1208-1218
20. Southwick SM, Vythilingam M, Charney DS (2005) The psychobiology of depression and resilience to stress:implications for prevention and treatment. Annu Rev Clin Psychol 1:255-291
21. Korpershoek C, van der Bijl J, Hafsteinsdóttir TB (2011) Self-efficacy and its influence on recovery of patients with stroke: a systematic review. J Adv Nurs 67(9):1876-1894
22. Savini, S., Buck, H.G., Dickson, V.V., Simeone, S., Pucciarelli, G., Fida, R., ... Vellone, E. (2015). Quality of life in stroke survivor-caregiver dyads: a new conceptual framework and longitudinal study protocol. *Journal of Advanced Nursing, 71*(3), 676-687. PMID: 25186274
23. Barclay-Goddard, R., King, J., Dubouloz, C.J., Schwartz, C.E., & Response Shift Think Tank Working Group. (2012). Building on transformative learning and response shift theory to investigate health-related quality of life changes over time in individuals with chronic health conditions and disability. *Archives of Physical Medicine and Rehabilitation, 93*, 214-220. PMID: 22289229
24. Shields, C.G., King, D.A., & Wynne, L.C. (1995). Interventions with later life families. In R.H. Mikesell, D.D. Lustermann, & S.H. McDaniel (Eds.), *Integrating Family Therapy: Handbook of*

Family Psychology and Systems Theory (pp. 141-158). Washington, DC: American Psychological Association.

25. Tehan T., Zale E., Rosand J, Vranceanu AM. Perceptions of needs and recommendations for implementation of a resiliency intervention for patients with stroke and their families; Nurses speak. Accepted for presentation at Brain Health Conference, Columbus, OH April 28-30, 2017
26. http://www.who.int/mental_health/media/en/545.pdf. Accessed March 3rd, 2017.
27. Collins PY, Patel V, Joestl SS, March D, Insel TR, Daar A, on behalf of the Grand Challenges in Global Mental Health Scientific Advisory Board and Executive Committee. Grand Challenges in Global Mental Health. *Nature.* 2011 July 7. 474(7354):27-30. PMID 21734685
28. Edmondson, D., Richardson, S., Fausett, J.K., Falzon, L., Howard, V.J., Kronish, I.M. (2013). Prevalence of PTSD in survivors of stroke and transient ischemic attack: a meta-analytic review. *PLoS One, 8,* e66435. PMCID: PMC3686746
29. Ayerbe, L., Ayis, S., Wolfe, C.D., Rudd, A.G. (2013). Natural history, predictors and outcomes of depression after stroke: systematic review and meta-analysis. *The British Journal of Psychiatry, 202,* 14-21. PMID:23284148
30. Carod-Artal, F.J., Egido, J.A. (2009). Quality of life after stroke: the importance of a good recovery. *Cerebrovascular Diseases, 27,* 204-214. PMID: 19342853
31. Bartoli, F., Lillia, N., Lax, A., Crocamo, C., Mantero, V., Carrà, G., Agostoni, E. Clerici, M. (2013). Depression after stroke and risk of mortality: a systematic review and meta-analysis. *Stroke Research and Treatment, 2013,* 862978. PMCID: PMC3606772
32. Denno, M.S., Gillard, P.J., Graham, G.D., DiBonaventura, M.D., Goren, A., Varon, S.F., Zorowitz, R. (2013). Anxiety and depression associated with caregiver burden in caregivers of stroke survivors with spasticity. *Archives of Physical Medicine and Rehabilitation, 94,*1731-1736. PMID: 23548544
33. Bakas, T., Burgener, S.C. (2002). Predictors of emotional distress, general health, and caregiving outcomes in family caregivers of stroke survivors. *Topics in Stroke Rehabilitation, 9,* 34-45. PMID: 14523721
34. Monin, J., Doyle, M., Levy, B., Schulz, R., Fried, T., Kershaw, T. (2016). Spousal associations between frailty and depressive symptoms: longitudinal findings from the cardiovascular health study. *Journal of the American Geriatrics Society, 64,* 824-830. PMID: 27100578
35. Lee, S,, Colditz, G.A., Berkman, L.F., Kawachi, I. (2003). Caregiving and risk of coronary heart disease in US women: a prospective study. *American Journal of Preventative Medicine, 24,*113-119. PMID: 12568816
36. Ji, J., Zöller, B., Sundquist, K., Sundquist, J. (2012). Increased risks of coronary heart disease and stroke among spousal caregivers of cancer patients. *Circulation, 125,* 1742-1747. PMID: 22415143
37. Schulz, R., Beach, S.R. (1999). Caregiving as a risk factor for mortality: the Caregiver Health Effects Study. *JAMA, 282,* 2215-2219. PMID: 10605972
38. Beach, S.R., Schulz, R., Williamson, G.M., Miller, L.S., Weiner, M.F., Lance, C.E. (2005). Risk factors for potentially harmful informal caregiver behavior. *Journal of the American Geriatrics Society, 53,* 255-261. PMID:15673349
39. Turner-Stokes, L., Hassan, N. (2002). Depression after stroke: a review of the evidence base to inform the development of an integrated care pathway. Part 1: Diagnosis, frequency and impact. *Clinical Rehabilitation, 16,* 231-247. PMID: 12017511
40. Schubart, J.R., Kinzie, M.B., & Farace, E. (2008). Caring for the brain tumor patient: family caregiver burden and unmet needs. *Neuro-oncology, 10,* 61-72. PMCID: PMC2600839

41. Palmer, S., & Glass, T.A. (2003). Family function and stroke recovery: a review. *Rehabilitation Psychology, 48,* 255-265.
42. Perrin, P.B., Heesacker, M., Hinojosa, M.S., Uthe, C.E., & Rittman, M.R. (2009). Identifying at-risk, ethnically diverse stroke caregivers for counseling: a longitudinal study of mental health. *Rehabilitation Psychology, 54,* 138-149. PMID: 19469603
43. Bienvenu, O.J., Colantuoni, E., Mendez-Tellez, P.A., Dinglas, V.D., Shanholtz, C., Husain, N., ... Needham,
D.M. (2012). Depressive symptoms and impaired physical function after acute lung injury. *American Journal of Respiratory and Critical Care Medicine, 185,* 517-524. PMCID: PMC3297105
44. Martin, L.R., Williams, S.L., Haskard, K.B., & DiMatteo, M.R. (2005). The challenge of patient adherence. *Therapeutics and Clinical Risk Management, 1,* 189-199. PMCID: PMC1661624
45. Rees, J., O'Boyle, C., & MacDonagh, R. (2001). Quality of life: impact of chronic illness on the partner. *Journal of the Royal Society of Medicine, 94,* 563-566. PMCID: PMC1282240
46. Azoulay, E., Pochard, F., Kentish-Barnes, N., Chevret, S., Aboab, J., Adrie, C., ... FAMIREA Study Group. (2005). Risk of post-traumatic stress symptoms in family members of intensive care unit patients. *American Journal of Respiratory and Critical Care Medicine, 171,* 987-994. PMID: 15665319
47. Jones, C., Skirrow, P., Griffiths, R.D., Humphris, G., Ingleby, S., Eddleston, J., ... Gager, M. (2004). Posttraumatic stress disorder-related symptoms in relatives of patients following intensive care. *Intensive Care Medicine, 30,* 456-460. PMID: 14767589
48. Jones, C., & Griffiths, R.D. (2007). Patient and caregiver counselling after the intensive care unit: what are the needs and how should they be met? *Current Opinion in Critical Care, 13,* 503-507. PMID: 17762226
49. Im, K., Belle, S.H., Shulz, R., Mendelsohn, A.B., Chelluri, L., & QOL-MV Investigators. (2004). Prevalence and outcomes of caregiving after prolonged (> or =48 hours) mechanical ventilation in the ICU. *Chest, 125,* 597-606. PMID: 14769744
50. Douglas, S.L., Daly, B.J., Kelley, C.G., O'Toole, E., & Montenegro, H. (2005). Impact of a disease management
program upon caregivers of chronically ill patients. *Chest, 128,* 3925-3936. PMID: 16354865
51. Martire LM, Lustig AP, Schulz R, Miller GE, Helgeson VS. Is it beneficial to involve a family member? A metaanalysis of psychosocial interventions for chronic illness. *Health Psychol* 2004; 23(6):599-611
52. Bakas, T., Clark, P.C., Kelly-Hayes, M., King, R.B., Lutz, B.J., Miller, E.L., & American Heart Association Council on Cardiovascular and Stroke Nursing and the Stroke Council. (2014). Evidence for stroke family caregiver and dyad interventions: a statement for healthcare professionals from the American Heart Association and American Stroke Association. *Stroke, 45,* 2836-2852. PMID: 25034718
53. Barclay-Goddard, R., King, J., Dubouloz, C.J., Schwartz, C.E., & Response Shift Think Tank Working Group. (2012). Building on transformative learning and response shift theory to investigate health-related quality of life changes over time in individuals with chronic health conditions and disability. *Archives of Physical Medicine and Rehabilitation, 93,* 214-220. PMID: 22289229
54. Cook, W.L. & Kenny, D.A. (2005). The Actor-Partner Interdependence Model: A model of bidirectional effects in developmental studies. *Journal of Behavioral Development, 29,* 102-109.
55. Zale EL, McCurley JL, Lin A, Funes C, Tehan T, Henhuis T, Rosand J., Vranceanu, A.M. Early psychological distress is crossectionally and prospectively interdependent between patients

admitted to the Neuro-ICU and their family caregivers. Submitted for presentation at Society of Behavioral Medicine, New Orleans, April 28-30, 2018.

56. Richards, K.C., Enderlin, C.A., Beck, C., McSweeney, J.C., Jones, T.C., & Roberson, P.K. (2007). Tailored biobehavioral interventions: a literature review and synthesis. *Research and Theory for Nursing Practices, 21*, 271-285. PMID: 18236771
57. Vranceanu AM, Ricklin E, Merker V, Park E, Plotkin SR. (2016) Mind body therapy for patients with
neurofibromatosis via live video; An RCT. Neurology 87 (8):806-14.
58. Zale EL, Piere-Louis C, Riklin E, Macklin E, Vranceanu AM. The impact of a mind body program on multiple dimensions of resiliency in geographically diverse patients with neurofibromatosis. JCCP accepted.
59. Bellg AJ, Borrelli B, Resnick B, et al. (2004). Enhancing treatment fidelity in health behavior change studies: Best practices and recommendations from the NIH Behavior Change Consortium. *Health Psychol.* 23:443-451. PMID:15367063.
60. Vranceanu AM**,** Merker VL, Plotkin SR, Park ER. The Relaxation Response Resiliency Program (3RP) in patients with neurofibromatosis 1, neurofibromatosis 2, and schwannomatosis: results from a pilot study. J Neurooncol. 2014; 120(1): 103-109.
61. Rounsavile, B.J., Carroll, K.M., & Onken, L.S. (2001). A stage model of behavioral therapies research: getting started and moving on from stage I. *Clinical Psychology: Science and Practice, 8*, 133-142.
62. Larsen, D.L., Attkisson, C.C., Hargreaves, W.A., & Nguyen, T.D. (1979). Assessment of client/patient satisfaction: development of a general scale. *Evaluation and Program Planning, 2*, 197-207. PMID: 10245370
63. Devilly, G.J., & Borkovec, T.D. (2000). Psychometric properties of the credibility/expectancy questionnaire. *Journal of Behavior Therapy and Experimental Psychiatry, 31,* 73-86. PMID: 11132119
64. Zigmond, A.S. & Snaith, R.P. (1983). The hospital anxiety and depression scale. *Acta Psychiatrica Scandinavica, 67,* 361-370. PMID: 6880820
65. Bhandari, N.J., Jain, T., Marolda, C., & ZuWallack, R.L. (2013). Comprehensive pulmonary rehabilitation results in clinically meaningful improvements in anxiety and depression in patients with chronic obstructive pulmonary disease. *Journal of Cardiopulmonary Rehabilitation and Prevention, 33,* 123-127. PMID: 23399845
66. Blanchard, E.B., Jones-Alexander, J., Buckley, T.C., & Forneris, C.A. (1996). Psychometric properties of the PTSD Checklist (PCL). *Behaviour Research and Therapy, 34,* 669-673. PMID: 8870294
67. Monson, C.M., Gradus, J.L., Young-Xu, Y., Schnurr, P.P., Price, J.L., & Schumm, J.A. (2008). Change in posttraumatic stress disorder symptoms: do clinicians and patients agree? *Psychological Assessment, 20,* 131-138. PMID: 18557690
68. Feldman, G., Hates, A., Kumar, S., Greeson, J., & Laurenceau, J.P. (2007). Mindfulness and emotion regulation: The development and initial validation of the Cognitive and Affective Mindfulness Scale Revised (CAMS-R). *Journal of Psychopathology and Behavioral Assessment, 29*, 177-190.
69. Schwarzer, R. & Jerusalem, M. (1995). Generalized Self-Efficacy scale. In J. Weinman, S. Wright, & M. Johnston (Eds.), *Measures in health psychology: A user's portfolio. Causal and control beliefs* (pp. 35-37). Windsor, UK: NFER-NELSON.

70. Cohen, S., Mermelstein, R., Kamarck, T., & Hoberman, H.M. (1985). Measuring the functional components of social support. In I.G. Sarason & B.R. Sarason (Eds.), *Social Support: Theory, Research and Applications* (pp.73-94). Netherlands: Springer.
71. Carver CS. (2006). Measure of Current Status. http://www.psy.miami.edu/faculty/ccarver/sclMOCS.html
72. Merz, EL, Roesch, SC, Malcarne, VL, Penedo, FJ, Llabre, MM, Weitzman, OB, ... Johnson, TP. (2013). Validation of interpersonal support evaluation list-12 (ISEL-12) scores among English- and Spanish-speaking Hispanics/Latinos from the HCHS/SOL sociocultural ancillary study. *Psychological Assessment* 26(2): 384-394.
73. Wilhelm, K. & Parker, G. (1988). The development of a measure of intimate bonds. *Psychological Medicine*, *18*, 225-234. PMID: 3363041
74. Guest, G., Bunce, A., & Johnson, L. (2006). How many interviews are enough? An experiment with data saturation and variability. *Field Methods, 18*(1), 24. doi: 10.1177/1525822X05279903
75. Browne RH. On the use of a pilot sample for sample size determination. *Stat Med*.1995;14: 1933-40. PMID:8532986.
76. Lancaster GA, Dodd S, Williamson PR. (2004). Design and analysis of pilot studies: recommendations for good practice. *J Eval Clin Pract*.;10:307-12. PMID: 15189396.
77. Rounsaville BJ, Caroll KM, Onken LS. (2001)A stage model of behavioral therapies research: getting started and moving on from stage I. *Clin Psychol Sci Pract*. 8:133–142
78. Shih WJ, Ohman-Strickland PA, Lin Y. (2004) Analysis of pilot and early phase studies with small sample sizes. *Stat Med.* 23:1827–1842 PMID: 15195318.
79. Whitehead AL, Julious SA, Cooper CL, Campbell MJ (2016). Estimating the sample size for a pilot randomized trial to minimise the overall trial sample size for the external pilot and main trial for a continuous outcome variable. *Stat Methods Med Res* 25:1057-1073. PMID: 26092476.
80. Miles, H.B., Huberman, A.M.(1994). *Qualitative Data Analysis.* Sage Publication, Thousand Oaks California.
81. Bowen DJ, Kreuter M, Spring B, et al. (2009) How we design feasibility studies. *Am J Prev Med.* May;36(5):452-7. PMID: 19362699.
82. Schafer JL, Graham JW. (2002). Missing data: our view of the state of the art. *Psychol Methods* 7(2):147-77. PMID: 12090408.
83. National Alliance for Caregiving (NAC). (2015). *Caregiving in the U.S. 2015.* Bethesda, MD: AARP Public Policy Institute.
84. Ruskin, P.E., Silver-Aylaian, M., Kling, M.A., Reed, S.A., Bradham, D.D., Hebel, J.R., ... Hauser, P. (2004). Treatment outcomes in depression: comparison of remote treatment through telepsychiatry to in-person treatment. *The American Journal of Psychiatry, 161,* 1471-1476. PMID: 15285975
85. Carlbring, P., & Andersson, G. (2006). Internet and psychological treatment. How well can they be combined? *Computers in Human Behavior, 22,* 545-553.
86. Clough, B.A., & Casey, L.M. (2011). Technological adjuncts to increase adherence to therapy: a review. *Clinical Psychology Review, 31,* 697-710. PMID: 21497153
87. Lin A, Jacobo M, Jacobs J, Tehan T, Salgueiro D, Rosand J, Vranceanu AM, Zale E. Gender differences in emotional distress among caregivers of patients admitted to the Neuroscience-Intensive Care. Submitted to the Society of Behavioral Medicine Annual Meeting, New Orleans, April 2018
88. Blake H, McKinney K, Treece E, Lee NB. (2002) An evaluation of screening measures for cognitive functioning after stroke. Age and Ageing, 31, pp.451-456.

Partners Human Research
Partners HealthCare
399 Revolution Drive, Suite 710
Somerville, MA 02145
Tel: 857-282-1900
Fax: 857-282-5693

Notification of IRB Review

Protocol #: 2018P002187

Date:	Aubust 14, 2019		
To:	Vranceanu, Ana-Maria,<br>MGH<br>Partners > MGH > Psychiatry		
From:	Partners Human Research<br>399 Revolution Drive, Suite 710<br>Somerville, MA 02145		
**Title of Protocol:**	**Recovering Together: Building resiliency in dyads in patients admitted to the Neuroscience Intensive Care Unit (NICU) and their caregivers**		
Version/g umBer:	4		
Version Date:	08/06/2019		
Sponsor/Fundinb/Support:	Proposal Title:	Recoverinb tobether: Nuildinb resiliency in dyads of patients admitted to the g euroscience Intensive Care Unit (g ICU) and their carebivers	
	Principal Investibator:	Vranceanu, Ana-Maria	
	Immediate Sponsor:	g IH	
	A# ard g umBer:	5R21g R017979-02	
	Fund w.	233585	
**IRB Amendment #:**	**16**		
IRN Revie# Type:	Expedited		
IRN Approval Date:	08/12/2019		
Approval/Activation Date:	08/14/2019		
**IRB Expiration Date:**	**10/26/2019**		

This project has Been revie# ed and approved By the **PHS IRB**. Durinb the revie# of this project, the IRN specifically considered (i) the risks and anticipated Benefits, if any, to suBjects; (ii) the selection of suBjects; (iii) the procedures for oBtaininb and documentinb informed consent; (iv) the safety of suBjects; and (v) the privacy of suBjects and confidentiality of the data.

Please note that if an IRN memBer had a conflict of interest # ith rebard to the revie# of this project, consistent # ith IRN policies and procedures, the memBer # as required to recuse him/herself and, if applicaBle, leave the room durinb the discussion and vote on this project except to provide information requested By the IRN.

Partners Human Research
Partners HealthCare
399 Revolution Drive, Suite 710
Somerville, MA 02145
Tel: 857-282-1900
Fax: 857-282-5693

- **The following has been approved by the IRB:**

  ✓ **Added the option to obtain verbal consent to our *Detailed Protocol* and *Protocol Summary.***

- **The following documents were reviewed and approved by the IRB:**

  Protocol Summary (8 6/ 2019)
  Detailed Protocol (8/6/2019)
  Consent Form

As Principal Investibator, you are responsiBle for ensurinb that this project is conducted in compliance # ith all applicaBle federal, state and local la# s and rebulations, institutional policies, and requirements of the IRN, # hich include, But are not limited to, the follo# inb:

1. SuBmission of any and all proposed chanbes to this project (e.b., protocol, recruitment materials, consent form, status of the study, etc.) to the IRN for revie# and approval prior to initiation of the chanbe(s), <u>except</u> # here necessary to eliminate apparent immediate hazards to the suBject(s). Chanbes made to eliminate apparent immediate hazards to suBjects must Be reported to the IRN as an unanticipated proBlem.
2. SuBmission of a continuinb revie# suBmission or institutional status report as required By the IRN and/or institution to continue the research, and suBmission of a final report # hen the project has Been closed or completed.
3. SuBmission of any and all unanticipated proBlems, includinb adverse event(s) in accordance # ith the IRN's policy on reportinb unanticipated proBlems includinb adverse events.
4. OBtaininb informed consent from suBjects or their lebally authorized representative prior to initiation of research procedures # hen and as required By the IRN and, # hen applicaBle, documentinb informed consent current IRN approved consent form(s) # ith the IRN-approval stamp in the document footer.
5. Informinb all investibators and study staff listed on the project of chanbes and unanticipated proBlems, includinb adverse events, involvinb risks to suBjects or others.
6. When investibator financial disclosure forms are required, suBmittinb updated financial disclosure forms for yourself and for informinb all site responsiBle investibators, co-investibators and any other memBers of the study staff identified By you as Beinb responsiBle for the desibn, conduct, or reportinb of this research study of their oBlibation to suBmit updated Investibator Financial Disclosure Forms for this protocol to the IRN if (a) they have acquired ne# financial interests related to the study and/or (B) any of their previously reported financial interests related to the study have chanbed.

IMPORTANT REMINDER: THE IRB HAS THE AUTHORITY TO TERMINATE PROJECTS THAT ARE NOT IN COMPLIANCE WITH THESE REQUIREMENTS.

Questions related to this project may Be directed to **Virginia, Rodriguez | Tel: 857-282-1899 | Email: VGRODRIGUEZ@PARTNERS.ORG**

Partners Human Research
Partners HealthCare
399 Revolution Drive, Suite 710
Somerville, MA 02145
Tel: 857-282-1900
Fax: 857-282-5693

cc:

Ana-Maria, Vranceanu, Psychiatry, Principal Investigator

Sofia, Distefano, BS, Neurology, Neurology, Research Coordinator/Manager

Melissa, Gates, Psychiatry, Research Coordinator/Manager

PARTNERS HUMAN RESEARCH COMMITTEE
PROTOCOL SUMMARY

Answer all questions accurately and completely in order to provide the PHRC with the relevant information to assess the risk-benefit ratio for the study. Do not leave sections blank.

PRINCIPAL/OVERALL INVESTIGATOR
Ana-Maria Vranceanu, Ph.D.

PROTOCOL TITLE
Recovering Together: Building resiliency in dyads in patients admitted to the Neuroscience Intensive Care Unit (NICU) and their caregivers

FUNDING
National Institute of Nursing Research

VERSION DATE
August 6, 2019

SPECIFIC AIMS

Concisely state the objectives of the study and the hypothesis being tested.

The current study has the following specific aims:

1) To determine the feasibility of recruitment, feasibility of program delivery, program credibility, and program satisfaction using evidence-based benchmarks.

   Hypothesis 1: We hypothesize that > 75% of the dyads approached will agree to participate.
   Hypothesis 2: We hypothesize that > 75% of dyads who start the intervention will complete at least 4 sessions.
   Hypothesis 3: We hypothesize that > 75% of participants will report average credibility (Credibility and Expectancy Questionnaire) scores greater than the scale's midpoint.
   Hypothesis 4: We hypothesize that > 75% participants will report average satisfaction (Client Satisfaction Scale) scores greater than the scale's midpoint.

2) To demonstrate a proof of concept that the Recovering Together program can sustainably improve emotional distress [Hospital Anxiety and Depression Scale; HADS], Post-Traumatic Symptoms (PTS) [PCL-S], resiliency variables (mindfulness, coping, social support and self-efficacy) and interpersonal factors (interpersonal bond).

   Hypothesis 1: We hypothesize that participation in the Recovering Together Program will be associated with a more potent decrease in emotional distress and PTS compared to participation in the educational program (control), and that these improvements will maintain at 3 month follow up.
   Hypothesis 2: We hypothesize that Recovering Together Program will be associated with a more potent increase in resiliency variables (mindfulness, coping, social support, self-efficacy) and interpersonal factors (interpersonal bond) compared to participation in the educational program, and that these improvements will maintain at 3 month follow up.

BACKGROUND AND SIGNIFICANCE

Provide a brief paragraph summarizing prior experience important for understanding the proposed study and procedures.

Acute neurological illnesses (ANIs) are common, costly and often lead to long-term disability. ANIs are biologically distinct injuries that disrupt the normal function of the brain. The most common ANIs in Neuroscience Intensive Care Units (NICU) include cerebrovascular (stroke/hemorrhage and brain aneurysm), structural (tumors and lesions/brain masses), and traumatic (TBI) brain injuries. NICU admissions for ANIs are prevalent (e.g., 795,000 acute stroke/year; 275,000 acute TBI/year) and costly; post NICU prolonged rehabilitation is common.
ANIs are associated with chronic emotional distress in both patients (pts) and caregivers (cgs). Although biologically heterogeneous, ANIs are unified by sudden onset, and substantial emotional distress in both pts (e.g., 12-43% anxiety; 10-58% depression; 20-29% post-traumatic stress PTS) and family cgs (27-60% depression, anxiety or PTS). These symptoms often become chronic and treatment resistant.
Pt and cg factors interact and influence physical and emotional outcomes in both pts and cgs. Post ANI emotional distress is associated with pts' poor medical adherence, slower recovery, higher mortality, and need of more caregiving assistance, which further increase cgs' distress and own risk for morbidity and mortality; in turn cgs' emotional distress interferes with ability to provide high-quality care to pts and negatively impacts pts' outcomes.
Current management of ANIs does not meet the psychological needs of pts and cgs for 3 reasons. First, although recognition of the emotional burden associated with NICU admission has increased, and some NICUs have social workers available to assist pts and cgs, there are no formal screening methods for emotional distress routinely integrated in practice during hospitalization, when the primary focus is on medical care and survival; further, there are no formal evidence-based treatments integrated within the medical care. When social workers are included to help pts and cgs, the care is brief and occurs only during hospitalization. When referrals to mental health services are provided to families at discharge, few will access additional treatment due to burden associated with traveling outside of home. Second, psychosocial interventions available for ANI pts or cgs are limited in that they are delivered when symptoms are already chronic, address only one emotional illness (e.g., depression or anxiety or PTS), and/or are focused on a *single member* of the pt-cg dyad. Even interventions labeled as "dyadic," which include pts and cgs, typically address only the pts' needs and do not focus on cg outcomes or on the dyad's interpersonal communication and bond/relationship. These interventions are not consistent with the *dyadic framework* which specifies that dyadic interventions should account for the interdependence between pt and cg psychosocial factors including their interpersonal bond by ensuring that both pts and cgs attend each session together, and by targeting improvement in outcomes for both pts and cgs. Third, most interventions are delivered using uniform protocols. However, the needs of ANI dyads are heterogeneous due to varying levels of post ANI impairment, identity of the cg, context and stage of life. A recent systematic review urged for the development of dyadic interventions that address the needs of *both* pts and cgs and are tailored to the specific needs of each ANI dyad.
We developed the first dyadic skills-based intervention – Recovering Together - to prevent chronic heightened emotional distress in at risk ANI pt-cg dyads. The "Recovering

Together" program is informed by the theoretical response-shift framework of adaptation to acute illness (successful adaptation implies recalibration of values and life goals), the family strength vulnerability model (within dyads relational systems have strengths and weaknesses in how they cope with life events), the dyadic longitudinal model (distress travel from one member of the dyad to the other across time), the APIM model, and the resiliency framework**.** The program is in line with recent recommendations for skill-based interventions for critical care patients, and uses preliminary data collected by our team for the past 3 years. The intervention teaches pts and cgs resiliency factors that are associated with well-being after trauma for both pts and cgs: *mindfulness* – the ability to stay present and defer judgment in the face of adversity; *coping*– the arsenal and application of one's behavioral, cognitive, and emotional strategies to manage stress; *social support* –empathetic interpersonal interactions that meet one's emotional and functional needs; *self-efficacy* – perceived ability to adapt under adversity and *positive dyadic interpersonal communication to increase interpersonal bond*. Informed by the aforementioned theoretical models, our conceptual model hypothesizes that by teaching both members of the dyad resiliency and interpersonal communication skills (e.g., Recovering Together) we will be able to sustainably decrease emotional distress in both members of the dyad.

RESEARCH DESIGN AND METHODS

Briefly describe study design and anticipated enrollment, i.e., number of subjects to be enrolled by researchers study-wide and by Partners researchers. Provide a brief summary of the eligibility criteria (for example, age range, gender, medical condition). Include any local site restrictions, for example, "Enrollment at Partners will be limited to adults although the sponsor's protocol is open to both children and adults."

The current study will be a two arm, feasibility pilot RCT.

We will randomize a total of 80 dyads (40 dyads in each arm) to either a psychosocial intervention or to an educational program in the Neuro ICU.

Eligible dyads (pts and cgs) must meet the following inclusion criteria:

1) Age 18 or older
2) English fluency and literacy
3) Access to high speed internet for video sessions
4) Pt with an informal cg (family or friend who provides unpaid care) available and willing to participate
5) Hospitalized with an ANI within 1-2 weeks (pt) OR primary cg of a pt currently admitted with an ANI
6) Either pt <u>or</u> cg have clinically significant symptoms of depression, anxiety, and/or PTS

One or more of the following exclusion criteria will render a pt ineligible:

1) Permanent or severe cognitive impairment severe enough to impede participation – This will be determined by nurses through an assessment conducted as part of usual care and that includes the MMSE (score of <23) and GCS (score of <10). Nurses are trained and use these measures as part of NICU care.

2) Dyads where the pt is anticipated to die or to never be able to participate due to medical sequelae. This will be determined by nurses.

   This exclusionary criterion is already used in our IRB approved prospective study with this population.

Briefly describe study procedures. Include any local site restrictions, for example, “Subjects enrolled at Partners will not participate in the pharmacokinetic portion of the study.” Describe study endpoints.

Study procedures:

Participants will be referred to the study by the nursing team who will ensure that patients are medically and cognitively able to participate (see above). Eligible dyads will next be screened, consented, and enrolled by the research assistant.

After enrollment, subjects will be randomized to either the newly developed psychosocial intervention or to the educational program (control). Dyads will be randomly assigned by using a random number sequence generator to ensure comparability between groups. Randomization will be developed by the statistician, without any input from the rest of the team. All subjects will be given baseline psychological and behavioral assessments that will assess depression, anxiety, PTSD symptoms, and other psychological constructs. All assessments will be administered via computer using the REDCap secure data collection system.

In the skills-based intervention group, sessions focus on developing skills to cope and manage ANI related stressors. The intervention will be tailored consistent with AHA recommendations for ANI skills-based interventions and will include 2 general and 4 specific modules. It is anticipated that the intervention will have 6 sessions with 2 general sessions delivered, in person if possible, within the NICU, or through live video using Vidyo if patients leave the hospital before sessions occur, and 4 tailored specific sessions (chosen from by the dyads from 6 available modules) to be delivered via live video using Vidyo.

Those in the educational program will receive general health information that mimics the Recovering Together Program, but without teaching any of the resiliency or interpersonal communication skills that are hypothesized to be responsible for improvement in emotional distress. There will also be 6 sessions, 2 in-person dyadic visits in the NICU and 4 dyadic virtual visits following discharge. The educational program group will not have the opportunity to specify which modules they would like to take; the modules will be predetermined. All participants will receive medical care as determined by their medical team.

Both groups will continue with their current care with addition to the educational programs provided.

Both groups will complete post-treatment psychological and behavioral assessments (identical to those administered at baseline) to be administered immediately after completion of the course and again at three months after completion of the course in order to measure long term outcomes. They will also be asked questions about the perceived efficacy of the intervention.

Study Endpoints:

1) Is the psychosocial skills-based intervention feasible, usable, and accepted by pts and cgs in the ICU?

2) Is the intervention associated with improvement in depression, anxiety and PTSD?

3) Is the effect of the skills-based intervention for pts and cgs in the ICU durable at 3 month follow up?

We will assess feasibility, usability, and acceptability by the enrolment numbers, participant completion in at least 4 out of 6 sessions, and the questionnaires, the Credibility and Expectancy and Client Satisfaction Scale. These will be the primary outcomes. The PCL-S, HADS, and the resiliency measures will serve as the secondary outcomes.

For studies involving treatment or diagnosis, provide information about standard of care at Partners (e.g., BWH, MGH) and indicate how the study procedures differ from standard care. Provide information on available alternative treatments, procedures, or methods of diagnosis.

NA

Describe how risks to subjects are minimized, for example, by using procedures which are consistent with sound research design and which do not unnecessarily expose subjects to risk or by using procedures already being performed on the subject for diagnostic or treatment purposes.

All study staff will complete required Partners human subjects trainings prior to the start of study procedures. In order to preserve confidentiality of participants, study data will not be linked to any identifying information; rather, study ID numbers will be assigned and used to identify participants. All study forms will be stored in locked storage spaces, to which only study staff will have access. All interventionists and assessors will have advanced training in clinical interviewing and assessment. Participants will be informed that they may refuse to answer questions that make them feel uncomfortable.

Describe explicitly the methods for ensuring the safety of subjects. Provide objective criteria for removing a subject from the study, for example, objective criteria for worsening disease/lack of improvement and/or unacceptable adverse events. The inclusion of objective drop criteria is especially important in studies designed with placebo control groups.

There is no risk of physical injury to participants. If a participant is judged to be suicidal at any time during participation, the interventionist will refer him or her to appropriate services, including the Acute Psychiatry Service at MGH if deemed appropriate.

FORESEEABLE RISKS AND DISCOMFORTS

Provide a brief description of any foreseeable risks and discomforts to subjects. Include those related to drugs/devices/procedures being studied and/or administered/performed solely for research purposes. In addition, include psychosocial risks, and risks related to privacy and confidentiality. When applicable, describe risks to a developing fetus or nursing infant.

Participants may feel uncomfortable completing various psychological questionnaires. As in any research study, there is a small risk that confidentiality may be breached; all efforts to minimize this risk will be taken, as outlined above. In the unlikely event that participants will become suicidal during the duration of the study, the research assistant will contact the PI and appropriate clinical intervention will be executed.

EXPECTED BENEFITS

Describe both the expected benefits to individual subjects participating in the research and the importance of the knowledge that may reasonably be expected to result from the study. Provide a brief, realistic summary of potential benefits to subjects, for example, "It is hoped that the treatment will result in a partial reduction in tumor size in at least 25% of the enrolled subjects." Indicate how the results of the study will benefit future patients with the disease/condition being studied and/or society, e.g., through increased knowledge of human physiology or behavior, improved safety, or technological advances.

Participants may not benefit from the study directly. However, knowledge from this research study may benefit others by enhancing our understanding of the role of psychosocial skills-based interventions in treating future pts and cgs within the NICU. All participants will receive $20 for the completion of each of the 3 assessment points.

EQUITABLE SELECTION OF SUBJECTS

The risks and benefits of the research must be fairly distributed among the populations that stand to benefit from it. No group of persons, for example, men, women, pregnant women, children, and minorities, should be categorically excluded from the research without a good scientific or ethical reason to do so. Please provide the basis for concluding that the study population is representative of the population that stands to potentially benefit from this research.

All subjects who satisfy the inclusion/exclusion criteria are eligible for enrollment in this study regardless of sex, race or ethnicity.

When people who do not speak English are excluded from participation in the research, provide the scientific rationale for doing so. Individuals who do not speak English should not be denied participation in research simply because it is inconvenient to translate the consent form in different languages and to have an interpreter present.

Only participants who can read and speak English will be included in the current study as not all assessment measures have been validated for use in non-English speaking populations.

For guidance, refer to the following Partners policy:

Obtaining and Documenting Informed Consent of Subjects who do not Speak English
https://partnershealthcare-public.sharepoint.com/ClinicalResearch/Non-English_Speaking_Subjects.1.10.pdf

RECRUITMENT PROCEDURES

Explain in detail the specific methodology that will be used to recruit subjects. Specifically address how, when, where and by whom subjects will be identified and approached about participation. Include any specific recruitment methods used to enhance recruitment of women and minorities.

Potential participants will be referred for study participation by their medical teams (nurses). The medical team will assess whether the ANI patients are able to consent, consistent with medical presentation. Trained study staff will be administering the MMSE and nurses will be administering the GSC to all patients and will refer only dyads where the patients' scores are higher than the established cut off scores on these measures. Pts and their respective cgs who are able to consent and who express interest in the study will speak with a study team member to learn more about the study and be screened for eligibility; those who wish to participate will complete consent. If cgs cannot be reached in person in the hospital, they will be contacted via phone for screening and completed of informed consent. Cgs will be given a copy of the consent form to review while discussing the study over the telephone. We will fax or securely e-mail the consent form prior to obtaining consent. Cgs will return a signed copy of the consent form.
If a patient who is interested in enrolling in the study is unable to provide a full signature for written consent due symptoms from their ANI (unable to move or use their arm to write), then we will ask them to make their mark on the signature line in the consent form. People who cannot make their mark on the consent form can indicate consent by other means, e.g., orally, nodding their head, etc. The means by which consent was given by the subject will be documented in the consent form and research record.

Patients and caregivers will be considered enrolled when they sign the consent form with the study staff. These procedures will be completed in a private medical space.

Participants will be explicitly informed that this intervention is a research study that does not constitute individualized, personal care. The intervention is a broad-based method of training that is not tailored to any particular individual. Should any participant seek formal mental healthcare, study staff will refer them to either MGH Psychiatry, as appropriate.

All dyads will be recruited within 1-2 weeks of pt's hospitalization. The pt's medical team will also alert the research assistant whether they anticipate that the patient might be able to participate in the study at a future point during the hospital stay. In situations where patients are unable to consent due to the severity of the stroke, we will enroll the informal cg and return to enroll the cg as soon as their mental capacity improves. The research assistant will not approach patients who are not mentally or physically capable to participate.

Eligible cases may also be identified by daily screening of Epic admission reports.

We will not include patients who do not have a caregiver. Informal caregivers will be designated by the health care proxy and verbally confirmed by the patient. Nurses will assist the study team with identifying the patient's health care proxy.

Provide details of remuneration, when applicable. Even when subjects may derive medical benefit from participation, it is often the case that extra hospital visits, meals at the hospital, parking fees or other inconveniences will result in additional out-of-pocket expenses related to study participation. Investigators may wish to consider providing reimbursement for such expenses when funding is available

All participants will receive $20 compensation for completing each of the 3 assessment points.

For guidance, refer to the following Partners policies:<br>Recruitment of Research Subjects<br>**https://partnershealthcare-public.sharepoint.com/ClinicalResearch/Recruitment_Of_Research_Subjects.pdf**<br><br>Guidelines for Advertisements for Recruiting Subjects<br>**https://partnershealthcare-public.sharepoint.com/ClinicalResearch/Guidelines_For_Advertisements.1.11.pdf**<br><br>Remuneration for Research Subjects<br>**https://partnershealthcare-public.sharepoint.com/ClinicalResearch/Remuneration_for_Research_Subjects.pdf**

CONSENT PROCEDURES

Explain in detail how, when, where, and by whom consent is obtained, and the timing of consent (i.e., how long subjects will be given to consider participation). For most studies involving more than minimal risk and all studies involving investigational drugs/devices, a licensed physician investigator must obtain informed consent. When subjects are to be enrolled from among the investigators' own patients, describe how the potential for coercion will be avoided.

After determining eligibility study staff will meet with potential dyads to review the informed consent document. After the document has been reviewed, study staff will answer any and all questions the pt or cg may have. Once all questions have been addressed, each member of the dyad will sign informed consent form, which will include a description of all study procedures, information about potential risks and benefits of participation, the option to receive text message reminders and study contact information (including that of the IRB) in case questions arise at a later time. The consent form will also explicitly state that study participation is voluntary, and that participants may refuse to answer any questions that make them uncomfortable, and may discontinue participation at any time. In addition, participants will be assured that withdrawal from the study will not compromise their medical care in any way.

As informed consent is a continuous process, participants will be given a copy of the signed informed consent document, and will be invited to ask questions about their participation at any point over the course of the study.

NOTE: When subjects are unable to give consent due to age (minors) or impaired decision-making capacity, complete the forms for Research Involving Children as Subjects of Research and/or Research Involving Individuals with Impaired Decision-making Capacity, available on the New Submissions page on the PHRC website:

https://partnershealthcare.sharepoint.com/sites/phrmApply/aieipa/irb

For guidance, refer to the following Partners policy:

Informed Consent of Research Subjects:

https://partnershealthcare-public.sharepoint.com/ClinicalResearch/Informed_Consent_of_Research_Subjects.pdf

DATA AND SAFETY MONITORING

Describe the plan for monitoring the data to ensure the safety of subjects. The plan should include a brief description of (1) the safety and/or efficacy data that will be reviewed; (2) the planned frequency of review; and (3) who will be responsible for this review and for determining whether the research should be altered or stopped. Include a brief description of any stopping rules for the study, when appropriate. Depending upon the risk, size and complexity of the study, the investigator, an expert group, an independent Data and Safety Monitoring Board (DSMB) or others might be assigned primary responsibility for this monitoring activity.

NOTE: Regardless of data and safety monitoring plans by the sponsor or others, the principal investigator is ultimately responsible for protecting the rights, safety, and welfare of subjects under his/her care.

Risks to participants are minimal. In the unlikely event that a participant is determined to be actively suicidal and at risk for self-harm during any study procedures, the research assistant will contact the PI (Vranceanu) and appropriate clinical intervention will be executed. Dr. Vranceanu may start a psychiatric consult depending on the severity of the situation. All study staff have been trained in responsible research conduct through a CITI course at MGH. The research assistant has also been trained on the importance of maintaining confidentiality, and the assignment of ID numbers. All data will be kept confidential, under lock-and-key, accessible only to trained study staff. Participants' data will be identified by ID number only, and a link between names and ID numbers will be kept separately under lock and key.

Describe the plan to be followed by the Principal Investigator/study staff for review of adverse events experienced by subjects under his/her care, and when applicable, for review of sponsor safety reports and DSMB reports. Describe the plan for reporting adverse events to the sponsor and the Partners' IRB and, when applicable, for submitting sponsor safety reports and DSMB reports to the Partners' IRBs. When the investigator is also the sponsor of the IND/IDE, include the plan for reporting of adverse events to the FDA and, when applicable, to investigators at other sites.

NOTE: In addition to the adverse event reporting requirements of the sponsor, the principal investigator must follow the Partners Human Research Committee guidelines for Adverse Event Reporting

Adverse events may be discovered in the event that a patient spontaneously reports an adverse event, or an adverse event is discovered during the assessment process. All adverse events will be reported by the PI to the Office of Research Compliance within 24 hours.

MONITORING AND QUALITY ASSURANCE

Describe the plan to be followed by the principal investigator/study staff to monitor and assure the validity and integrity of the data and adherence to the IRB-approved protocol. Specify who will be responsible for monitoring, and the planned frequency of monitoring. For example, specify who will review the accuracy and completeness of case report form entries, source documents, and informed consent.

NOTE: Regardless of monitoring plans by the sponsor or others, the principal investigator is ultimately responsible for ensuring that the study is conducted at his/her investigative site in accordance with the IRB-approved protocol, and applicable regulations and requirements of the IRB.

Once completed, a member of study staff will verify that all items on all questionnaires have been addressed. Data will be checked for out of range values using frequency distributions prior to analyzing the data. The Principal Investigator will be responsible for ensuring compliance with IRB procedures.

For guidance, refer to the following Partners policies:

Data and Safety Monitoring Plans and Quality Assurance
https://partnershealthcare-public.sharepoint.com/ClinicalResearch/DSMP_in_Human_Subjects_Research.pdf

Reporting Unanticipated Problems (including Adverse Events)
https://partnershealthcare-public.sharepoint.com/ClinicalResearch/Reporting_Unanticipated_Problems_including_Adverse_Events.pdf

PRIVACY AND CONFIDENTIALITY

Describe methods used to protect the privacy of subjects and maintain confidentiality of data collected. This typically includes such practices as substituting codes for names and/or medical record numbers; removing face sheets or other identifiers from completed surveys/questionnaires; proper disposal of printed computer data; limited access to study data; use of password-protected computer databases; training for research staff on the importance of confidentiality of data, and storing research records in a secure location.

NOTE: Additional measures, such as obtaining a Certificate of Confidentiality, should be considered and are strongly encouraged when the research involves the collection of sensitive data, such as sexual, criminal or illegal behaviors.

As noted above, study data will not be linked to any identifying information; rather, study ID numbers will be assigned and used to identify participants. All study forms will be stored in

locked storage spaces, to which only study staff will have access. All study staff will complete required Partners human subjects trainings prior to the start of study procedures.

SENDING SPECIMENS/DATA TO RESEARCH COLLABORATORS OUTSIDE PARTNERS

Specimens or data collected by Partners investigators will be sent to research collaborators outside Partners, indicate to whom specimens/data will be sent, what information will be sent, and whether the specimens/data will contain identifiers that could be used by the outside collaborators to link the specimens/data to individual subjects.

Data collected from the current study will not be sent to research collaborators outside of Partners.

Specifically address whether specimens/data will be stored at collaborating sites outside Partners for future use not described in the protocol. Include whether subjects can withdraw their specimens/data, and how they would do so. When appropriate, submit documentation of IRB approval from the recipient institution.

Specimens / data will not be stored at collaborating sites outside of Partners for future use not described in this protocol.

RECEIVING SPECIMENS/DATA FROM RESEARCH COLLABORATORS OUTSIDE PARTNERS

When specimens or data collected by research collaborators outside Partners will be sent to Partners investigators, indicate from where the specimens/data will be obtained and whether the specimens/data will contain identifiers that could be used by Partners investigators to link the specimens/data to individual subjects. When appropriate, submit documentation of IRB approval and a copy of the IRB-approved consent form from the institution where the specimens/data were collected.

Specimens and data will not be collected by research collaborators outside of Partners.

PARTNERS HUMAN RESEARCH COMMITTEE DETAILED PROTOCOL

Principal Investigator: Ana-Maria Vranceanu, PhD

Protocol Title: Recovering Together: Building resiliency in dyads in patients admitted to the Neuroscience Intensive Care Unit (NICU) and their caregivers

Funding: National Institute of Nursing Research

Version Date: 8/6/2019

I. BACKGROUND AND SIGNIFICANCE

Acute neurological illnesses (ANIs) are common, costly and often lead to long-term disability. ANIs are biologically distinct injuries that disrupt the normal function of the brain. The most common ANIs in Neuroscience Intensive Care Units (NICU) include cerebrovascular (stroke/hemorrhage and brain aneurysm), structural (tumors and lesions/brain masses), and traumatic (TBI) brain injuries. NICU admissions for ANIs are prevalent (e.g., 795,000 acute stroke/year; 275,000 acute TBI/year) and costly[1,2]; post NICU prolonged rehabilitation is common[3].

ANIs are associated with chronic emotional distress in both patients (pts) and caregivers (cgs). Although biologically heterogeneous, ANIs are unified by sudden onset, and substantial emotional distress in both pts (e.g., 12-43% anxiety[4-7]; 10-58% depression[4,7,8]; 20-29% post-traumatic stress PTS[5,9]) and family cgs (27-60% depression, anxiety or PTS [4,10,11]). These symptoms often become chronic and treatment resistant[26,27].

Pt and cg factors interact and influence physical and emotional outcomes in both pts and cgs. Post ANI emotional distress is associated with pts' poor medical adherence[28], slower recovery[28-30], higher mortality[29-31], and need of more caregiving assistance[32], which further increase cgs' distress[30,33,34] and own risk for morbidity[35,36] and mortality[37]; in turn cgs' emotional distress interferes with ability to provide high-quality care to pts[38,39] and negatively impacts pts' outcomes.

Current management of ANIs does not meet the psychological needs of pts and cgs for 3 reasons[40-51]. First, although recognition of the emotional burden associated with NICU admission has increased, and some NICUs have social workers available to assist pts and cgs, there are no formal screening methods for emotional distress routinely integrated in practice during hospitalization, when the primary focus is on medical care and survival; further, there are no formal evidence-based treatments integrated within the medical care. When social workers are included to help pts and cgs, the care is brief and occurs only during hospitalization. When referrals to mental health services are provided to families at discharge, few will access additional treatment due to burden associated with traveling outside of home. Second, psychosocial interventions available for ANI pts or cgs are limited in that they are delivered when symptoms are already chronic, address only one emotional illness (e.g., depression or anxiety or PTS), and/or are focused on a *single member* of the pt-cg dyad. Even interventions labeled as "dyadic," which include pts and cgs, typically address only the pts' needs and do not

focus on cg outcomes or on the dyad's interpersonal communication and bond/relationship[12,52]. These interventions are not consistent with the *dyadic framework*[22] which specifies that dyadic interventions should account for the interdependence between pt and cg psychosocial factors including their interpersonal bond by ensuring that both pts and cgs attend each session together, and by targeting improvement in outcomes for both pts and cgs. Third, most interventions are delivered using uniform protocols. However, the needs of ANI dyads are heterogeneous due to varying levels of post ANI impairment, identity of the cg, context and stage of life. A recent systematic review[52] urged for the development of dyadic interventions that address the needs of *both* pts and cgs and are tailored to the specific needs of each ANI dyad.

We developed the first dyadic skills-based intervention – Recovering Together - to prevent chronic heightened emotional distress in at risk ANI pt-cg dyads. The "Recovering Together" program is informed by the theoretical response-shift framework of adaptation to acute illness[53] (successful adaptation implies recalibration of values and life goals), the family strength vulnerability model[24] (within dyads relational systems have strengths and weaknesses in how they cope with life events), the dyadic longitudinal model[22] (distress travel from one member of the dyad to the other across time), the APIM model[54], and the resiliency framework[17]**.** The program is in line with recent recommendations for skill-based interventions for critical care patients, and uses preliminary data collected by our team for the past 3 years[4, 13-15]. The intervention teaches pts and cgs resiliency factors that are associated with well-being after trauma for both pts and cgs: *mindfulness* – the ability to stay present and defer judgment in the face of adversity[18]; *coping*– the arsenal and application of one's behavioral, cognitive, and emotional strategies to manage stress[19]; *social support* –empathetic interpersonal interactions that meet one's emotional and functional needs[20]; *self-efficacy* – perceived ability to adapt under adversity[21] and *positive dyadic interpersonal communication to increase interpersonal bond*[12]. Informed by the aforementioned theoretical models, our conceptual model hypothesizes that by teaching both members of the dyad resiliency and interpersonal communication skills (e.g., Recovering Together) we will be able to sustainably decrease emotional distress in both members of the dyad

In addition, our team has an established record of collaboration on published or ongoing investigations. **Emotional distress is prevalent in dyads, interdependent between pt and cg, and negatively associated with resiliency factors[4].** Our team conducted a cross-sectional study of pt-cg dyads in the NICU (40% stroke, 30% tumor). 75% pts and 84% cgs approached agreed to participate. 74% pts had been intubated at one time during NICU admission, and 2/3 were discharged home. Rates of clinically significant symptoms of depression, anxiety and PTS did not differ between pts (24%, 43%, 21%) and cgs (24%, 46%, 17%), or by any demographic or medical characteristic. Dyadic modeling showed that for both pts and cgs, mindfulness and coping impacted both self and partner's emotional distress symptoms. We showed: 1) feasibility of recruitment; 2) high emotional distress in dyads; and that 3) modifiable resiliency factors (mindfulness and coping) are intervention targets interdependently associated with distress in pts and cgs, regardless of the identity of cg (e.g., spouse, friend, etc).

Resiliency factors are associated with lower emotional distress in NICU dyads[13]. Our team found that resiliency factors of mindfulness, coping, self-efficacy and patient-caregiver interactions were associated with decreased emotional distress in dyads of ANI. This study confirms mindfulness, coping as intervention targets and provides novel evidence on self-efficacy and patient-caregiver interaction as additional important intervention targets.

ANI pts have greater anxiety than cancer patients at early diagnoses[14]. We led the first cross-comparison study of emotional distress among dyads with ANI and cancer. This study supports the priority of addressing emotional distress in ANI dyads as has been emphasized for cancer dyads.

Clinically significant emotional distress in one member of the dyad at hospitalization predicts chronic emotional distress in at least 1 of the dyad members 3 and 6 months later[55]. Our team has an ongoing prospective study of dyads with ANI. Retention rates for dyads due for assessments at 3 and 6 months thus far are 84% and 91% for pts and 87.7% and 95.7% for cgs, confirming our ability to retain post-ANI participants. Within each dyad, if one member screens in for clinically significant symptoms for any diagnosis (i.e., depression, anxiety or PTSD) at hospitalization there is good sensitivity and specificity that one member of the dyad will endorse clinically significant symptoms 3 months later. This study shows a reliable method for identifying dyads of patients at risk for chronic heightened emotional distress by identifying dyads in which either the pt or cg screens in for heightened emotional distress (symptoms of depression, anxiety or PTSD).

Caregiver gender moderates the prospective association of resiliency factors to emotional distress[87]. This study found that at the time of admission resiliency factors have main effects on emotional distress, with no differences by cg gender. However, significant interaction effects emerged prospectively such that male cgs with high mindfulness at baseline demonstrated lower levels of emotional distress at 3 and 6 months later than did males with low mindfulness ($p = 0.026$ and $p <0.013$). Similarly, women cgs with high intimate bond at baseline reported the lowest levels of depression symptoms 3 and 6 months later ($p < 0.020$). This study confirmed the need to assess and address resiliency factors early in the recovery process, and identified important gender differences to be accounted for in intervention development.

Recovering Together; Developing a novel dyadic resiliency skills program for ANI pt-cg dyads at risk for chronic emotional distress[15]. With funding from American Heart Association, we conducted 20 qualitative interviews with pt-cg stroke dyads at risk for chronic emotional distress during NICU hospitalization. We also conducted additional clinical interviews with 10 pt-cg dyads representative of other ANI diagnoses. 83% dyads approached agreed to participate. Pts (23) and cgs (25) were mostly women. Dyads were mostly spouses and mothers-daughter. Data was analyzed with Nvivo10. Main themes did not differ by medical diagnoses: 1) most challenging and distressing experiences: uncertainty about future, anxiety, depression, sleep difficulties, worries about the future, guilt, managing job with caretaking, making treatment decisions, lack of predictability; 2) concerns about interpersonal relationships (self-image, role changes, role fulfillment); 3) fear of recurrence; 4) adjusting to sequelae. Dyads noted interest in a resiliency program (30/30) and preferred a combination of in person and live video sessions (30/30). Dyads learned about resiliency skills (e.g., name, description and goal) and agreed they would be helpful to them. They also noted interest in learning about: survivorship plans, adaptation to deficits (present or anticipated), and return to normal living. We found no thematic differences between stroke dyads and other ANI dyads. Challenges associated with embracing the caregiver role emerged as a theme while differences by the identity of caregiver (e.g., spouse vs. friend, vs parent) did not. Themes associated with the gender of the cg emerged and have been incorporated in the intervention.

Nurses perception of the needs for and feasibility of "Recovering Together for ANI families[25]. We conducted 2 focus groups (N =15) with NICU nurses who provided feedback on the qualitative findings from our 30 ANI dyads and shared own experiences and opinions on

implementation and scalability of the intervention including nurse involvement. Nurses concurred with pts experiences, and suggested strategies to recruit and retain dyads for the study, which are now included in the methodology section of the current grant proposal. Studies 3.6 and 3.7 represent the building blocks for the development of our Recovering Together program and manual. Nurses provided edits and contributed to the iterative development of the manual. These studies also confirm feasibility of conducting the pilot RCT proposed through this R21.

II. SPECIFIC AIMS

The current study has the following objectives:

Aim 1: **To determine the feasibility of recruitment, feasibility of program delivery, program credibility, and program satisfaction using evidence-based benchmarks.**

Hypothesis 1: We hypothesize that > 75% of the dyads approached will agree to participate.
Hypothesis 2: We hypothesize that > 75% of dyads who start the intervention will complete at least 4 sessions.
Hypothesis 3: We hypothesize that > 75% of participants will report average credibility (Credibility and Expectancy Questionnaire) scores greater than the scale's midpoint.
Hypothesis 4: We hypothesize that > 75% participants will report average satisfaction (Client Satisfaction Scale) scores greater than the scale's midpoint.

Aim 2: **To demonstrate a proof of concept that the Recovering Together program can sustainably improve emotional distress [Hospital Anxiety and Depression Scale; HADS], Post Traumatic Symptoms (PTS) [PCL-S], resiliency variables (mindfulness, coping, social support and self-efficacy) and interpersonal factors (interpersonal bond).**

Hypothesis 1: We hypothesize that participation in the Recovering Together Program will be associated with a more potent decrease in emotional distress and PTS compared to participation in the educational program (control), and that these improvements will maintain at 3 month follow up.
Hypothesis 2: We hypothesize that Recovering Together Program will be associated with a more potent increase in resiliency variables (mindfulness, coping, social support, self-efficacy) and interpersonal factors (interpersonal bond) compared to participation in the educational program, and that these improvements will maintain at 3 month follow up.

For this feasibility pilot RCT, our primary outcomes (feasibility, credibility and satisfaction) will be assessed in Aim 1. Our secondary outcomes in this trial are: emotional distress, PTS, mindfulness, coping, social support, self-efficacy, and interpersonal bond, and will be assessed in Aim 2.

III. SUBJECT SELECTION

All participants will be recruited from the Massachusetts General Hospital Neuroscience ICU, using IRB approved recruitment materials.

Inclusion/Exclusion Criteria

Eligible dyads (Pts and Cgs) must meet the following inclusion criteria:

1) Age 18 or older
2) English fluency and literacy
3) Access to high speed internet for video sessions
4) Pt with an informal cg (family or friend who provides unpaid care) available and willing to participate
5) Hospitalized with an ANI within 1-2 weeks (pt) OR primary cg of a pt currently admitted with an ANI
6) Either pt or cg have clinically significant symptoms of depression, anxiety, and/or PTS

One or more of the following exclusion criteria will render a pt ineligible:

1) Permanent and severe cognitive impairment severe enough to impede participation – This will be determined by trained study staff through an assessment conducted as part of usual care and that includes the MMSE (score of <23) and GCS (score of <10). Nurses and study staff are trained and use these measures as part of NICU care.
2) Dyads where the pt is anticipated to die or to never be able to participate due to medical sequelae. This will be determined by nurses.

All adult patients and family caregivers, satisfying all inclusion criteria, are eligible for enrollment in this study regardless of sex, race, or ethnicity. Vulnerable populations will not be recruited.

Recruitment

Patients will be recruited from the NICU at MGH from the medical team (nurses). Recruitment will be facilitated by the nursing team who will introduce the study to eligible dyads and who will also assess whether pts are able to consent, consistent with medical presentation, and cognitive status. Trained study staff will be administering the MMSE and nurses will administer the GSC, to all patients and will refer only dyads where the patients scores are higher than the established cut off scores on these measures. Consistent with NICU practice, nurses will administer the GSC. They will refer participants to the study only if they are cleared medically and cognitively. The team will page the RA using the secure Voalte system used by our team and nursing staff, when both the pt and cg are present and able to hear more about the study. The RA will only approach pts identified and cleared by the medical team. The RA will ensure eligibility of both pts and cgs based on the additional inclusionary and exclusionary criteria depicted above. The RA will finalize the screening and conduct informed consent**.** All participants will receive a physical copy of the informed consent form which has the contact information for the PI. If cg~~s~~ cannot be reached in person in the hospital, they will be contacted via phone for screening and completion of informed consent. Cgs will be given a copy of the consent form to review while discussing the study over the telephone. We will fax or securely e-mail the consent form prior to obtaining consent. Cg will return a signed copy of the consent form. If a patient who is interested in enrolling in the study is unable to provide a full written signature due symptoms from their

ANI (unable to move or use their arm to write), then we will ask them to make their mark on the signature line in the consent form. People who cannot make their mark on the consent form can indicate consent by other means, e.g., orally, nodding their head, etc. The means by which consent was given by the subject will be documented in the consent form and research record. Patients and caregivers will be considered enrolled when they sign the consent form with the study staff. Please see details on the informed consent process in the next section, Subject Enrollment (Section IV). These procedures will be completed in a private medical space.

Participants will be explicitly informed that this intervention is a research study that does not constitute individualized, personal care. The intervention is a broad-based method of training that is not tailored to any particular individual. Should any participant seek formal mental healthcare, study staff will refer them to either MGH Psychiatry, as appropriate.

We will recruit pts with any type of ANI and their cgs, ensuring representation of all NICU diagnoses. All cgs will be recruited within the first week of the pts' hospitalization. Pt's medical team will also alert the research assistant whether they anticipate that the patient might be able to participate in the study at a future point during the hospital stay. In situations where patients are unable to consent due to the severity of the ANI, we will enroll the informal cg and return to enroll the pt as soon as their mental capacity improves. The research assistant will not approach patients who are not mentally or physically capable to participate. This study does not include participants with impaired decision making.

We will not include patients who do not have a caregiver. Informal caregivers will be designated by the health care proxy and verbally confirmed by the patient. Nurses will assist the study team with identifying the patient's health care proxy.

Eligible cases may also be identified by daily screening of Epic admission reports.

The study team will keep track of all pts and cgs approached who refuse to participate (along with reasons for refusal), as well as those who were not approached and reasons why.

When the RA approaches a patient, patients and caregivers will be given the choice of watching a short recruitment video that contains testimonials from previous patients.

The recruitment process was developed and refined through prior research for the past 2 years.

IV. SUBJECT ENROLLMENT

Participants will be referred to the study by the nursing team who will ensure that patients are medically and cognitively able to participate. Eligible dyads will next be screened, consented, and enrolled by the research assistant.

After determining eligibility, study staff will meet with potential dyads to review the informed consent document. Patients who cannot make their mark on the consent form because of lingering symptoms from the ANI can indicate consent by other means, e.g., orally, nodding

their head, etc. The means by which consent was given by the subject will be documented in the consent form and research record. If cgs cannot be reached in person in the hospital, they will be contacted via phone to complete informed consent. Cgs will be given a copy of the consent form to review while discussing the study over the telephone. We will fax or securely e-mail the consent form prior to obtaining consent. Cgs will return a signed copy of the consent form. After the document has been reviewed, study staff will answer any and all questions the pt or cg may have. Once all questions have been addressed, each member of the dyad will sign the consent form, which will include a description of all study procedures, the option to receive text message reminders, information about potential risks and benefits of participation, and study contact information (including that of the IRB) in case questions arise at a later time. The consent form will also explicitly state that study participation is voluntary, and that participants may refuse to answer any questions that make them uncomfortable, and may discontinue participation at any time. In addition, participants will be assured that withdrawal from the study will not compromise their medical care in any way.

As informed consent is a continuous process, participants will be given a copy of the signed informed consent document, and will be invited to ask questions about their participation at any point over the course of the study.

Following a study enrollment and baseline assessment, dyads will be randomly assigned to either the newly developed psychosocial intervention or to the educational program (control) using a random number sequence generator to ensure comparability between groups. We will document time between assessment and intervention initiation, and analyze as a predictor of study outcomes as needed. Randomization will be developed by the statistician, without any input from the rest of the team.

V. STUDY PROCEDURES

After enrollment, participants will complete study assessments. All subjects will be given baseline psychological and behavioral assessments that will assess depression, anxiety, PTS symptoms, and other psychological constructs. Assessments will be administered online, using the REDCap system. Subjects may choose to fill out these questionnaires on-site, or to fill them out at home on a personal computer or other Internet-equipped device. This assessment includes demographic information and a battery of psychological questionnaires. We will also collect information about important clinical variables including duration of acute hospitalization. All questionnaires are itemized below:

Administered at Baseline Only:
Demographics
Prior Mental Health History questions
Credibility Questionnaire

Administered at Baseline, Post-Intervention, and Follow-up:
Medical history information from LMR (i.e., prior ANI status, current psychotropic meds, comorbid medical conditions, etc.)
Post-Traumatic Stress Disorder Checklist (PCL-S)[29]

Hospital Depression and Anxiety Scale (HADS)[30]
Measure of Current Status Part A (MOCS-A)[31]
The Cognitive and Affective Mindfulness Scale (CAMS)[32]
World Health Organization Quality of Life (WHOQOL-BREF)[33]
Dyadic Relationship Scale (DRS)
Experience in Close Relationships – Relationship Structure (ECR-RS)

Administered Post-Intervention and Follow-up Only:
Client Satisfaction Questionnaire (CSQ-3)[34]

The study staff will complete the following questionnaires to assess symptom severity for each patient at all 3 timepoints (baseline, post intervention and follow up):
Modified Rankin Scale (mRS)[35] [Pts only]
Barthel Index[36] [Pts only]

After completing the baseline assessments, dyads will be randomized to either the newly developed psychosocial intervention or to treatment as usual.

The psychosocial intervention entails 6 sessions, each 30 minutes. Both the pt and cg participate in each session. The intervention is manualized, and teaches dyads resiliency (mindfulness, social support, self-efficacy, coping skills) and interpersonal communications (interpersonal bond) skills. The first 2 sessions will occur in person, during hospitalization. The next 4 sessions are chosen from 5 available, depending on each dyad's preference, and are delivered after discharge via secure live video. For the video sessions, dyads can participate from the same or different locations. During the in hospital sessions, dyads learn diaphragmatic breathing, mindfulness, self-care, and dialectics. During the video sessions, dyads learn how to identify negative thinking patterns and replace them with adaptive thoughts, how to communicate effectively and openly, how to engage in self-care, and how to accept things that can't be changed. Participants in the intervention group will also receive treatment as usual. This may include meeting with nurses, physical therapist**s**, medical doctors, and other members of the pt's medical team. Treatment as usual may also involve administration of SSRI to those patients with motor problems.

Those in the educational program (control) will receive general health information that mimics the Recovering Together Program, but without teaching any of the resiliency or interpersonal communication skills that are hypothesized to be responsible for improvement in emotional distress. The educational program will also have 6 sessions, 2 in-person dyadic visits in the NICU and 4 dyadic virtual visits following discharge. It controls for dose and support from clinician. Both members of the dyads participate in all sessions. The topics of each session include: education about the stress of the ANI on patient and caregiver; education on the importance of self-care; education on stress associated with discharge and home adjustment; education on the importance of following up with medical recommendations; education on interpersonal stress as part of adjustment to ANI; education on self-care. The educational program condition will ensure that patients will remain blind to intervention or control and increase confidence that improvement in outcomes are due to the active ingredients of the intervention and not confounds. Participants in the educational program will continue with their

current care. This may include meeting with nurses, physical therapists, medical doctors, and other members of the pt's medical team. Treatment as usual may also involve administration of SSRI to those patients with motor problems.

Participants in both groups will be given post-treatment psychological and behavioral assessments identical to those administered at baseline, in addition to the CSQ-3. As a baseline, participants will be given the option to complete post-treatment questionnaires on site or at home. Participants will be asked to complete questionnaires immediately after completion of the group intervention (T2), and 3 months (T3) after completion of the group intervention, in order to measure long term outcomes. Study staff will email these questionnaires to the participants via the REDCap system or give participants the option to complete them over the phone with a study staff member.

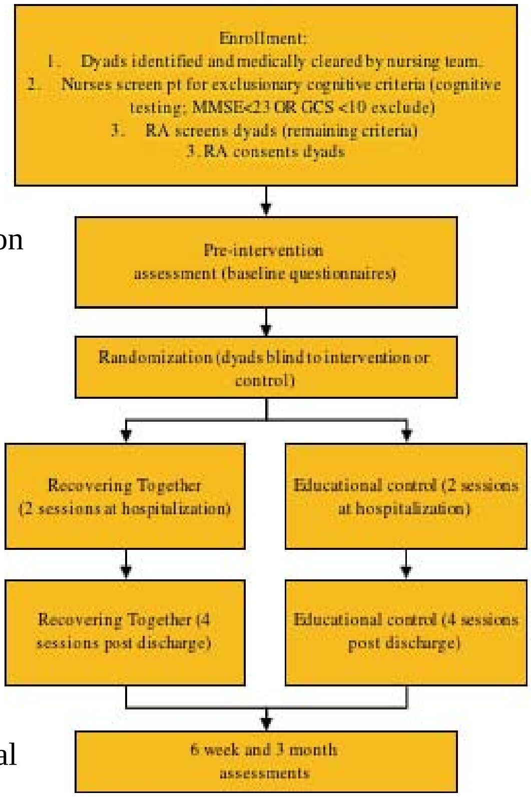

VI. BIOSTATISTIC ANALYSIS

We plan to conduct a RCT with patients from the Neuro-ICU. The main intervention goal is to provide dyads with resiliency and interpersonal communication skills necessary to optimize recovery. Dyads will be medically cleared by a member of the nursing staff. Nurses will then screen dyads for exclusionary cognitive criteria. Dyads will complete additional screening and baseline questionnaires with a trained research assistant. Dyads will be randomized to one of two groups 1) Psychosocial skills based intervention (Recovering Together), or 2) Educational Control.

We chose our study measures based on our theoretical frameworks, strong psychometric properties in studies of patients with ANI, and feasibility in our prior work with ANI dyads. Measures will be collected at baseline, post-intervention, and at 3 month follow up. Data collection and management will be conducted with Research Electronic Data Capture. Dyads will be given the option to complete measures electronically, by paper and pencil, or telephone.

1) Is the psychosocial skills-based intervention feasible, credible usable, and accepted by pts and cgs in the ICU?

Our primary aim focuses on trial feasibility, acceptability, credibility and preliminary effect. In pilot studies, ≥30 participants are recommended per group to establish feasibility and detect larger effect sizes for ≥1 outcome. We plan to recruit 80 dyads (160 participants), 40 dyads (80 participants) per arm to establish feasibility, acceptability, credibility and estimate effect size for emotional distress variables (primary quantitative outcomes). Assuming attrition of over 25% (in excess of what we experienced in our preliminary studies), we will have the necessary 30 dyads (60 participants) per arm. This

size is considered to yield stable estimates of M/SDs based on prior behavioral trial recommendations. Effect sizes from this study may overestimate power in future sample calculations. We chose study measures based on our theoretical frameworks, strong psychometric properties in studies of patients with ANI, and feasibility work in our prior work with ANI dyads. Feasibility will be reported as the percentage of patients enrolled in the study who complete at least 75% of the intervention sessions. Demonstration of feasibility will be assessed by the number of individuals who drop out of the study prior to completing the post- intervention assessment and the rate of missed sessions. If drop-out rate or missed-session rate exceeds 25%, revisions to the intervention may be needed. We will also report number of patients approached, enrolled, randomized and who completed time 2 and time 3 to determine feasibility.

2) Is the psychosocial skills-based intervention effective for pts and cgs in the ICU?

Dr. Vranceanu developed the proposed study design in collaboration with the NICU study team (nurses, physicians, clinical interns) and an MGH psychologist who specializes in using mindfulness and emotion regulation in both chronic illness and medically healthy populations. With funding from the American Heart Association, we conducted 20 qualitative interviews with pt-cg stroke dyads at risk for chronic emotional distress during NICU hospitalization. We also conducted additional clinical interviews with 10 pt-cg dyads representative of other ANI diagnoses. 83% of dyads approached agreed to participate. Pts (23) and cgs (25) were mostly women. Dyads were mostly spouses and mothers-daughter. Dyads noted interest in a resiliency program (30/30) and preferred a combination of in person and live video sessions (30/30). Dyads learned about resiliency skills (name, description, and goal) and agreed they would be helpful to them. They also noted interest in learning about: survivorship plans, adaptation to deficits (present or anticipated), and return to normal living. This led to the development and subsequent refinement (through feedback from the nursing team) of the Recovering Together Program active intervention.

3) Is the effect of the skills-based intervention for pts and cgs in the ICU durable?

We will assess feasibility, usability, and acceptability by the enrollment numbers, participants completion in at least 4 out of 6 sessions, and the questionnaires, The Credibility and Expectancy and Client Satisfaction Scale. These will be primary outcomes. The PCL-S, HADS, and resiliency measures will serve as the secondary outcomes.

We will use student’s t-test to assess within-group differences in long-term outcomes. We will compare measures at the 3 month follow-up assessment to measures at the post-intervention assessment for both study arms.

We will also use t tests and chi squared tests to assess differences at the 3 month follow up.

VII. RISKS AND DISCOMFORTS

There is a risk that some participants may feel uncomfortable completing various psychological questionnaires or parts of the skills-based on intervention. Participants are free to withdraw from the study at any time, as the study is completely voluntary.

As in any research study, there is a small risk that confidentiality may be breached; all efforts to minimize this risk will be taken. In the unlikely event that participants will become suicidal during the duration of the study, the research assistant will contact the PI and the appropriate clinical intervention will be executed.

VIII. POTENTIAL BENEFITS

Participants in this study may observe a reduction in depression, anxiety, and/or psychological and physiological markers of stress, as well as an improvement in perceived qualify of life. It is hoped that the intervention will result in improvements across these domains. Participants may learn new techniques for managing distress and lifestyle factors that may enhance wellbeing, both in disease-specific domains as well as in their general lives. In addition, all participants will receive $20 for the completion of each of the 3 assessment points.

IX. MONITORING AND QUALITY ASSURANCE

Electronic information will be stored in REDCap (Research Electronic Data Capture), a free, secure, and HIPAA-compliant web-based application hosted by the Partners HealthCare Research Computing Enterprise Research Infrastructure & Services (ERIS) group (based at the PHS Needham corporate datacenter). Data will be stored on password protected computers that will be stored in secure locations at all times. Paper data files (with coded subject identification) will be stored in a locked filing cabinet. Only research staff will have access to these data locations.

A unique anonymous identifier will be assigned to each subject; subsequently, all data collected will be associated exclusively with this identifier. This includes all questionnaires administered over the course of the study, as well as home practice logs.

Data from this study will be stored for three years after the publication of all study results, at which time all paper data files will be shredded, and computer files will be deleted.

Data Management and Quality Control Procedures

To maximize accuracy and security, all survey data will be collected and stored on REDCap. Research staff will ensure that proper consent has been obtained before sending the REDCap survey to each participant.

REDCap (Research Electronic Data Capture) is a free, secure, HIPAA compliant web-based application hosted by the Partners HealthCare Research Computing Enterprise Research Infrastructure & Services (ERIS) group. Vanderbilt University, with collaboration from a

consortium of academic and non-profit institutional partners, has developed this software toolset and workflow methodology for electronic collection and management of research and clinical study data. Data collection projects rely on a study-specific data dictionary defined by members of the research team with planning assistance from Harvard Catalyst, The Harvard Clinical and Translational Science Center EDC Support Staff. This iterative development and testing process results in a well-planned data collection strategy for individual studies. Using REDCap, the research team can also design web-based surveys and engage potential respondents using a variety of notification methods. REDCap provides flexible features that can be used for a variety of research projects and provides an intuitive interface to enter data with real time validation (automated data type and range checks). The system offers easy data manipulation with audit trails, reports for monitoring and querying participant records, and an automated export mechanism to common statistical packages (SPSS, SAS, Stata, R/S-Plus).

Since consistency of application of the study protocol is critical to acquiring high quality data, all research personnel have undergone or will undergo a competency-based training program prior to enrolling subjects.

Data and Safety Monitoring Plan

Adverse Event Monitoring: Throughout the study subjects will be monitored for the occurrence of events defined as any undesirable experience or unanticipated risk. Lack of effect of treatment is not considered an event. All adverse events will be reported on an adverse event form. The Principle Investigator has the responsibility of reporting serious adverse events (death, life threatening illness or injury, serious injury, or permanent disability) to PHRC within 24-72 hours of notification.

X. REFERENCES

1. Center for Disease Control and Prevention. (2015). Stroke Facts: Stroke in the United States. *CDC*. Retrieved from http://www.cdc.gov/stroke/facts.htm.
2. Center for Disease Control and Prevention. (2015). *Report to Congress on Traumatic Brain Injury in the United States: Epidemiology and Rehabilitation.* National Center for Injury Prevention and Control; Division of Unintentional Injury Prevention. Atlanta, GA.
3. The Society of Critical Care Medicine. (2015). What is critical care? *SCCM*. Retrieved from www.myicucare.org.
4. Shaffer KM, Riklin E, Stagl JM, Rosand J, Vranceanu AM. (2016). Mindfulness and coping are inversely related to psychiatric symptoms in patients and informal caregivers in the Neuroscience ICU: Implications for Clinical Care. Critical *Care Medicine*, 44 2036-2038. PMID: 27513536
5. Jackson, J.C., Mitchell, N., & Hopkins, R.O. (2011). Cognitive functioning, mental health, and quality of life in ICU survivors: an overview. *Anesthesiology Clinics, 29,* 751-764. PMID: 22078921
6. Kress, J.P., Gehlbach, B., Lacy, M., Pliskin, N., Pohlman, A.S., & Hall, J.B. (2003). The long-term psychological effects of daily sedative interruption on critically ill patients. *American Journal of Respiratory and Critical Care Medicine, 168*, 1457-1461. PMID: 14525802
7. Cheung, A.M., Tansey, C.M., Tomlinson, G., Diaz-Granados, N., Matté, A., Barr, A., ... Herridge, M.S. (2006). Two-year outcomes, health care use, and costs of survivors of acute respiratory

distress syndrome. *American Journal of Respiratory and Critical Care Medicine, 174,* 538-544. PMID: 16763220

8. Daydow, D.S., Gifford, J.M., Desai, S.V., Bienvenu, O.J., & Needham, D.M. (2009). Depression in general intensive care unit survivors: a systematic review. *Intensive Care Medicine, 35,* 796-809. PMID: 19165464
9. Jackson, J.C., Hart, R.P., Gordon, S.M., Hopkins, R.O., Girard, T.D., & Ely, W.E. (2007). Post-traumatic stress disorder and post-traumatic stress symptoms following critical illness in medical intensive care unit patients: assessing the magnitude of the problem. *Critical Care, 11,* R27. PMCID: PMC2151890
10. Choi, J., Hoffman, J.A., Schulz, R., Ren, D., Donahoe, M.P., Given, B., & Sherwood, P.R. (2013). Health risk behaviors in family caregivers during patients' stay in intensive care units: a pilot analysis. *American Journal of Critical Care, 22,* 41-45. PMCID: PMC4109809
11. McAdam, J.L., Fontaine, D.K., White, D.B., Dracup, K.A., & Puntillo, K.A. (2012). Psychological symptoms of family members of high-risk intensive care unit patients. *American Journal of Critical Care, 21,* 386-393. PMID:23117902
12. McCarthy, M.J., Lyons, S.L., Powers, L.E. (2012). Relational factors associated with depressive symptoms among stroke survivor-spouse dyads. *Journal of Family Social Work* 15: 303-320.
13. Shaffer KM, Riklin E, Stagl JM, Rosand J, Vranceanu AM. (2016) Psychosocial resiliency is associated with lower psychological distress among dyads of patients and their informal caregivers in the neuroscience intensive care unit. J Critical Care 2016 Jul 16; 36:154-159. PMID: 27546765
14. Schaffer KM, Jacobs JM, Coleman JN, Rosand J, Temel, J, Greer JA. Vranceanu AM**.** Anxiety and depressive symptoms among two seriously ill medical populations and their family caregivers. Neurocritical Care 2016; ahead of print.
15. Zale EL, Piere-Louis C, Tehan T, Henhuis T, Rosand J., Vranceanu, A.M. Improving resiliency and brain health after acute neurological illness; Perceptions of patients and families. Accepted for presentation at Brain Health Conference, Columbus, OH April 28-30, 2017.
16. Martire LM, Lustig AP, Schulz R, Miller GE, Helgeson VS. Is it beneficial to involve a family member? A meta-analysis of psychosocial interventions for chronic illness. *Health Psychol* 2004; 23(6):599-611
17. Bonanno, G. A., Galea, S., Bucciarelli, A., & Vlahov, D. (2007). What predicts psychological resilience after disaster? The role of demographics, resources, and life stress. *Journal of Consulting and Clinical Psychology, 75,*671–682. http://dx.doi.org/10.1037/0022-006X.75.5.671
18. Brown KW, Ryan RM (2003) The benefits of being present: mindfulness and its role in psychological well-being.J Pers Soc Psychol 84(4):822
19. Donnellan C, Hevey D, Hickey A, O'Neil D (2006) Defining and quantifying coping strategies after stroke: a review. J Neurol Neurosurg Psychiatry 77:1208-1218
20. Southwick SM, Vythilingam M, Charney DS (2005) The psychobiology of depression and resilience to stress:implications for prevention and treatment. Annu Rev Clin Psychol 1:255-291
21. Korpershoek C, van der Bijl J, Hafsteinsdóttir TB (2011) Self-efficacy and its influence on recovery of patients with stroke: a systematic review. J Adv Nurs 67(9):1876-1894
22. Savini, S., Buck, H.G., Dickson, V.V., Simeone, S., Pucciarelli, G., Fida, R., ... Vellone, E. (2015). Quality of life in stroke survivor-caregiver dyads: a new conceptual framework and longitudinal study protocol. *Journal of Advanced Nursing, 71*(3), 676-687. PMID: 25186274
23. Barclay-Goddard, R., King, J., Dubouloz, C.J., Schwartz, C.E., & Response Shift Think Tank Working Group. (2012). Building on transformative learning and response shift theory to

investigate health-related quality of life changes over time in individuals with chronic health conditions and disability. *Archives of Physical Medicine and Rehabilitation, 93,* 214-220. PMID: 22289229

24. Shields, C.G., King, D.A., & Wynne, L.C. (1995). Interventions with later life families. In R.H. Mikesell, D.D. Lustermann, & S.H. McDaniel (Eds.), *Integrating Family Therapy: Handbook of Family Psychology and Systems Theory* (pp. 141-158). Washington, DC: American Psychological Association.
25. Tehan T., Zale E., Rosand J, Vranceanu AM. Perceptions of needs and recommendations for implementation of a resiliency intervention for patients with stroke and their families; Nurses speak. Accepted for presentation at Brain Health Conference, Columbus, OH April 28-30, 2017
26. http://www.who.int/mental_health/media/en/545.pdf. Accessed March 3rd, 2017.
27. Collins PY, Patel V, Joestl SS, March D, Insel TR, Daar A, on behalf of the Grand Challenges in Global Mental Health Scientific Advisory Board and Executive Committee. Grand Challenges in Global Mental Health. *Nature.* 2011 July 7. 474(7354):27-30. PMID 21734685
28. Edmondson, D., Richardson, S., Fausett, J.K., Falzon, L., Howard, V.J., Kronish, I.M. (2013). Prevalence of PTSD in survivors of stroke and transient ischemic attack: a meta-analytic review. *PLoS One, 8,* e66435. PMCID: PMC3686746
29. Ayerbe, L., Ayis, S., Wolfe, C.D., Rudd, A.G. (2013). Natural history, predictors and outcomes of depression after stroke: systematic review and meta-analysis. *The British Journal of Psychiatry, 202,* 14-21. PMID:23284148
30. Carod-Artal, F.J., Egido, J.A. (2009). Quality of life after stroke: the importance of a good recovery. *Cerebrovascular Diseases, 27,* 204-214. PMID: 19342853
31. Bartoli, F., Lillia, N., Lax, A., Crocamo, C., Mantero, V., Carrà, G., Agostoni, E. Clerici, M. (2013). Depression after stroke and risk of mortality: a systematic review and meta-analysis. *Stroke Research and Treatment, 2013,* 862978. PMCID: PMC3606772
32. Denno, M.S., Gillard, P.J., Graham, G.D., DiBonaventura, M.D., Goren, A., Varon, S.F., Zorowitz, R. (2013). Anxiety and depression associated with caregiver burden in caregivers of stroke survivors with spasticity. *Archives of Physical Medicine and Rehabilitation, 94,*1731-1736. PMID: 23548544
33. Bakas, T., Burgener, S.C. (2002). Predictors of emotional distress, general health, and caregiving outcomes in family caregivers of stroke survivors. *Topics in Stroke Rehabilitation, 9,* 34-45. PMID: 14523721
34. Monin, J., Doyle, M., Levy, B., Schulz, R., Fried, T., Kershaw, T. (2016). Spousal associations between frailty and depressive symptoms: longitudinal findings from the cardiovascular health study. *Journal of the American Geriatrics Society, 64,* 824-830. PMID: 27100578
35. Lee, S,, Colditz, G.A., Berkman, L.F., Kawachi, I. (2003). Caregiving and risk of coronary heart disease in US women: a prospective study. *American Journal of Preventative Medicine, 24,*113-119. PMID: 12568816
36. Ji, J., Zöller, B., Sundquist, K., Sundquist, J. (2012). Increased risks of coronary heart disease and stroke among spousal caregivers of cancer patients. *Circulation, 125,* 1742-1747. PMID: 22415143
37. Schulz, R., Beach, S.R. (1999). Caregiving as a risk factor for mortality: the Caregiver Health Effects Study. *JAMA, 282,* 2215-2219. PMID: 10605972
38. Beach, S.R., Schulz, R., Williamson, G.M., Miller, L.S., Weiner, M.F., Lance, C.E. (2005). Risk factors for potentially harmful informal caregiver behavior. *Journal of the American Geriatrics Society, 53,* 255-261. PMID:15673349

39. Turner-Stokes, L., Hassan, N. (2002). Depression after stroke: a review of the evidence base to inform the development of an integrated care pathway. Part 1: Diagnosis, frequency and impact. *Clinical Rehabilitation, 16,* 231-247. PMID: 12017511
40. Schubart, J.R., Kinzie, M.B., & Farace, E. (2008). Caring for the brain tumor patient: family caregiver burden and unmet needs. *Neuro-oncology, 10,* 61-72. PMCID: PMC2600839
41. Palmer, S., & Glass, T.A. (2003). Family function and stroke recovery: a review. *Rehabilitation Psychology, 48,* 255-265.
42. Perrin, P.B., Heesacker, M., Hinojosa, M.S., Uthe, C.E., & Rittman, M.R. (2009). Identifying at-risk, ethnically diverse stroke caregivers for counseling: a longitudinal study of mental health. *Rehabilitation Psychology, 54,* 138-149. PMID: 19469603
43. Bienvenu, O.J., Colantuoni, E., Mendez-Tellez, P.A., Dinglas, V.D., Shanholtz, C., Husain, N., ... Needham,
D.M. (2012). Depressive symptoms and impaired physical function after acute lung injury. *American Journal of Respiratory and Critical Care Medicine, 185,* 517-524. PMCID: PMC3297105
44. Martin, L.R., Williams, S.L., Haskard, K.B., & DiMatteo, M.R. (2005). The challenge of patient adherence. *Therapeutics and Clinical Risk Management, 1,* 189-199. PMCID: PMC1661624
45. Rees, J., O'Boyle, C., & MacDonagh, R. (2001). Quality of life: impact of chronic illness on the partner. *Journal of the Royal Society of Medicine, 94,* 563-566. PMCID: PMC1282240
46. Azoulay, E., Pochard, F., Kentish-Barnes, N., Chevret, S., Aboab, J., Adrie, C., ... FAMIREA Study Group. (2005). Risk of post-traumatic stress symptoms in family members of intensive care unit patients. *American Journal of Respiratory and Critical Care Medicine, 171,* 987-994. PMID: 15665319
47. Jones, C., Skirrow, P., Griffiths, R.D., Humphris, G., Ingleby, S., Eddleston, J., ... Gager, M. (2004). Posttraumatic stress disorder-related symptoms in relatives of patients following intensive care. *Intensive Care Medicine, 30,* 456-460. PMID: 14767589
48. Jones, C., & Griffiths, R.D. (2007). Patient and caregiver counselling after the intensive care unit: what are the needs and how should they be met? *Current Opinion in Critical Care, 13,* 503-507. PMID: 17762226
49. Im, K., Belle, S.H., Shulz, R., Mendelsohn, A.B., Chelluri, L., & QOL-MV Investigators. (2004). Prevalence and outcomes of caregiving after prolonged (> or =48 hours) mechanical ventilation in the ICU. *Chest, 125,* 597-606. PMID: 14769744
50. Douglas, S.L., Daly, B.J., Kelley, C.G., O'Toole, E., & Montenegro, H. (2005). Impact of a disease management
program upon caregivers of chronically ill patients. *Chest, 128,* 3925-3936. PMID: 16354865
51. Martire LM, Lustig AP, Schulz R, Miller GE, Helgeson VS. Is it beneficial to involve a family member? A metaanalysis of psychosocial interventions for chronic illness. *Health Psychol* 2004; 23(6):599-611
52. Bakas, T., Clark, P.C., Kelly-Hayes, M., King, R.B., Lutz, B.J., Miller, E.L., & American Heart Association Council on Cardiovascular and Stroke Nursing and the Stroke Council. (2014). Evidence for stroke family caregiver and dyad interventions: a statement for healthcare professionals from the American Heart Association and American Stroke Association. *Stroke, 45,* 2836-2852. PMID: 25034718
53. Barclay-Goddard, R., King, J., Dubouloz, C.J., Schwartz, C.E., & Response Shift Think Tank Working Group. (2012). Building on transformative learning and response shift theory to investigate health-related quality of life changes over time in individuals with chronic health

conditions and disability. *Archives of Physical Medicine and Rehabilitation, 93,* 214-220. PMID: 22289229
54. Cook, W.L. & Kenny, D.A. (2005). The Actor-Partner Interdependence Model: A model of bidirectional effects in developmental studies. *Journal of Behavioral Development, 29*, 102-109.
55. Zale EL, McCurley JL, Lin A, Funes C, Tehan T, Henhuis T, Rosand J., Vranceanu, A.M. Early psychological distress is crossectionally and prospectively interdependent between patients admitted to the Neuro-ICU and their family caregivers. Submitted for presentation at Society of Behavioral Medicine, New Orleans, April 28-30, 2018.
56. Richards, K.C., Enderlin, C.A., Beck, C., McSweeney, J.C., Jones, T.C., & Roberson, P.K. (2007). Tailored biobehavioral interventions: a literature review and synthesis. *Research and Theory for Nursing Practices, 21*, 271-285. PMID: 18236771
57. Vranceanu AM, Ricklin E, Merker V, Park E, Plotkin SR. (2016) Mind body therapy for patients with
neurofibromatosis via live video; An RCT. Neurology 87 (8):806-14.
58. Zale EL, Piere-Louis C, Riklin E, Macklin E, Vranceanu AM. The impact of a mind body program on multiple dimensions of resiliency in geographically diverse patients with neurofibromatosis. JCCP accepted.
59. Bellg AJ, Borrelli B, Resnick B, et al. (2004). Enhancing treatment fidelity in health behavior change studies: Best practices and recommendations from the NIH Behavior Change Consortium. *Health Psychol.* 23:443-451. PMID:15367063.
60. Vranceanu AM**,** Merker VL, Plotkin SR, Park ER. The Relaxation Response Resiliency Program (3RP) in patients with neurofibromatosis 1, neurofibromatosis 2, and schwannomatosis: results from a pilot study. J Neurooncol. 2014; 120(1): 103-109.
61. Rounsavile, B.J., Carroll, K.M., & Onken, L.S. (2001). A stage model of behavioral therapies research: getting started and moving on from stage I. *Clinical Psychology: Science and Practice, 8*, 133-142.
62. Larsen, D.L., Attkisson, C.C., Hargreaves, W.A., & Nguyen, T.D. (1979). Assessment of client/patient satisfaction: development of a general scale. *Evaluation and Program Planning, 2,* 197-207. PMID: 10245370
63. Devilly, G.J., & Borkovec, T.D. (2000). Psychometric properties of the credibility/expectancy questionnaire. *Journal of Behavior Therapy and Experimental Psychiatry, 31,* 73-86. PMID: 11132119
64. Zigmond, A.S. & Snaith, R.P. (1983). The hospital anxiety and depression scale. *Acta Psychiatrica Scandinavica, 67*, 361-370. PMID: 6880820
65. Bhandari, N.J., Jain, T., Marolda, C., & ZuWallack, R.L. (2013). Comprehensive pulmonary rehabilitation results in clinically meaningful improvements in anxiety and depression in patients with chronic obstructive pulmonary disease. *Journal of Cardiopulmonary Rehabilitation and Prevention, 33,* 123-127. PMID: 23399845
66. Blanchard, E.B., Jones-Alexander, J., Buckley, T.C., & Forneris, C.A. (1996). Psychometric properties of the PTSD Checklist (PCL). *Behaviour Research and Therapy, 34,* 669-673. PMID: 8870294
67. Monson, C.M., Gradus, J.L., Young-Xu, Y., Schnurr, P.P., Price, J.L., & Schumm, J.A. (2008). Change in posttraumatic stress disorder symptoms: do clinicians and patients agree? *Psychological Assessment, 20,* 131-138. PMID: 18557690
68. Feldman, G., Hates, A., Kumar, S., Greeson, J., & Laurenceau, J.P. (2007). Mindfulness and emotion regulation: The development and initial validation of the Cognitive and Affective

Mindfulness Scale Revised (CAMS-R). *Journal of Psychopathology and Behavioral Assessment, 29*, 177-190.

69. Schwarzer, R. & Jerusalem, M. (1995). Generalized Self-Efficacy scale. In J. Weinman, S. Wright, & M. Johnston (Eds.), *Measures in health psychology: A user's portfolio. Causal and control beliefs* (pp. 35-37). Windsor, UK: NFER-NELSON.
70. Cohen, S., Mermelstein, R., Kamarck, T., & Hoberman, H.M. (1985). Measuring the functional components of social support. In I.G. Sarason & B.R. Sarason (Eds.), *Social Support: Theory, Research and Applications* (pp.73-94). Netherlands: Springer.
71. Carver CS. (2006). Measure of Current Status. http://www.psy.miami.edu/faculty/ccarver/sclMOCS.html
72. Merz, EL, Roesch, SC, Malcarne, VL, Penedo, FJ, Llabre, MM, Weitzman, OB, ... Johnson, TP. (2013). Validation of interpersonal support evaluation list-12 (ISEL-12) scores among English- and Spanish-speaking Hispanics/Latinos from the HCHS/SOL sociocultural ancillary study. *Psychological Assessment* 26(2): 384-394.
73. Wilhelm, K. & Parker, G. (1988). The development of a measure of intimate bonds. *Psychological Medicine, 18,* 225-234. PMID: 3363041
74. Guest, G., Bunce, A., & Johnson, L. (2006). How many interviews are enough? An experiment with data saturation and variability. *Field Methods*, *18*(1), 24. doi: 10.1177/1525822X05279903
75. Browne RH. On the use of a pilot sample for sample size determination. *Stat Med*.1995;14: 1933-40. PMID:8532986.
76. Lancaster GA, Dodd S, Williamson PR. (2004). Design and analysis of pilot studies: recommendations for good practice. *J Eval Clin Pract*.;10:307-12. PMID: 15189396.
77. Rounsaville BJ, Caroll KM, Onken LS. (2001)A stage model of behavioral therapies research: getting started and moving on from stage I. *Clin Psychol Sci Pract*. 8:133–142
78. Shih WJ, Ohman-Strickland PA, Lin Y. (2004) Analysis of pilot and early phase studies with small sample sizes. *Stat Med.* 23:1827–1842 PMID: 15195318.
79. Whitehead AL, Julious SA, Cooper CL, Campbell MJ (2016). Estimating the sample size for a pilot randomized trial to minimise the overall trial sample size for the external pilot and main trial for a continuous outcome variable. *Stat Methods Med Res* 25:1057-1073. PMID: 26092476.
80. Miles, H.B., Huberman, A.M.(1994). *Qualitative Data Analysis.* Sage Publication, Thousand Oaks California.
81. Bowen DJ, Kreuter M, Spring B, et al. (2009) How we design feasibility studies. *Am J Prev Med.* May;36(5):452-7. PMID: 19362699.
82. Schafer JL, Graham JW. (2002). Missing data: our view of the state of the art. *Psychol Methods* 7(2):147-77. PMID: 12090408.
83. National Alliance for Caregiving (NAC). (2015). *Caregiving in the U.S. 2015.* Bethesda, MD: AARP Public Policy Institute.
84. Ruskin, P.E., Silver-Aylaian, M., Kling, M.A., Reed, S.A., Bradham, D.D., Hebel, J.R., ... Hauser, P. (2004). Treatment outcomes in depression: comparison of remote treatment through telepsychiatry to in-person treatment. *The American Journal of Psychiatry, 161*, 1471-1476. PMID: 15285975
85. Carlbring, P., & Andersson, G. (2006). Internet and psychological treatment. How well can they be combined? *Computers in Human Behavior, 22*, 545-553.
86. Clough, B.A., & Casey, L.M. (2011). Technological adjuncts to increase adherence to therapy: a review. *Clinical Psychology Review, 31*, 697-710. PMID: 21497153

87. Lin A, Jacobo M, Jacobs J, Tehan T, Salgueiro D, Rosand J, Vranceanu AM, Zale E. Gender differences in emotional distress among caregivers of patients admitted to the Neuroscience-Intensive Care. Submitted to the Society of Behavioral Medicine Annual Meeting, New Orleans, April 2018
88. Blake H, McKinney K, Treece E, Lee NB. (2002) An evaluation of screening measures for cognitive functioning after stroke. Age and Ageing, 31, pp.451-456.

Partners HealthCare System
Research Consent Form

Certificate of Confidentiality Template
Version Date: January 2018

Subject Identification

Protocol Title: Recovering Together: Building resiliency in dyads in patients admitted to the Neuroscience Intensive Care Unit (NICU) and their caregivers

Principal Investigator: Ana-Maria Vranceanu, PhD

Site Principal Investigator: N/A

Description of Subject Population: Acute neurological illnesses (ANIs) patient - caregiver dyads admitted to the NICU

About this consent form

Please read this form carefully. It tells you important information about a research study. A member of our research team will also talk to you about taking part in this research study. People who agree to take part in research studies are called “subjects.” This term will be used throughout this consent form.

Partners HealthCare System is made up of Partners hospitals, health care providers, and researchers. In the rest of this consent form, we refer to the Partners system simply as “Partners.”

If you have any questions about the research or about this form, please ask us. Taking part in this research study is up to you. If you decide to take part in this research study, you must sign this form to show that you want to take part. We will give you a signed copy of this form to keep.

A description of this clinical trial will be available on *https://www.ClinicalTrials.gov*, as required by the U.S. Law. This website will not include information that can identify you. At most, the website will include a summary of the results. You can search this website at any time.

Why is this research study being done?

This research study is being done to understand the experiences of patients admitted to the neuroscience intensive care unit (Neuro-ICU) and their primary caregivers. The purpose of this study is to compare two dyadic (patient and caregiver) programs to improve emotional distress in patients and caregivers.

We are asking you to take part in this research study because you are at least 18 years of age, an English-speaking patient with an acute neurological illness (ANI) admitted to the intensive care unit, or a caregiver of a patient with an ANI.

About 80 dyads (160 people) will take part in this research study.

This research study is being conducted by the Neuroscience Intensive Care Unit at Massachusetts General Hospital. The National Institute of Nursing Research is paying for this study to be done.

How long will I take part in this research study?

It will take you about 4-5 months to complete this research study.

What will happen in this research study?

The training program we are testing was developed based on information from the nursing care team and patients and caregivers like yourself. It has 6 sessions and it teaches behavioral, and psychosocial skills. Two sessions will take place during hospitalization and 4 sessions will take place through a secure, live-videoconferencing program called Vidyo.

If you choose to participate in this study, we will ask you to sign this consent form before we do any study procedures.

Once you sign this consent form and agree to participate in this study, you and your partner will be randomly assigned together to one of two groups. You will be assigned by chance (like flipping a coin) to one of the two dyadic training programs. There is no way to predict which of the two groups you and your partner you will be in. Both groups will participate in a dyadic program with a study therapist and a program manual. We do not know right now which intervention will be more effective. You will not know what the other intervention is.

Below is an outline of the study schedule:

Subject Identification

Study Intake (1/2 hour)

During this portion of the study, you will fill out several questionnaires online or in person, through a secure system. The survey questions will ask you about your stress levels, mental health, behavior, medical symptoms, and quality of life. You can complete these questionnaires in-person during your intake visit, on paper at home, or on a personal computer at home.

For your safety, we will also ask you to provide contact information for a family member or friend that we may contact on your behalf in case of emergency.

Weekly Sessions 1-6

The intervention will have 6 sessions with 2 general sessions delivered in-person within the Neuro-ICU and 4 tailored specific sessions to be delivered via live video using Vidyo, when you return home or at rehab. Vidyo is a HIPAA approved, secure online videoconferencing software program. We will help you set up for Vidyo sessions. We will help you install Vidyo on your computer, tablet or smartphone and will teach you how to use it before we start the groups. Once you log in from a webcam-equipped computer, the Vidyo program will allow you to see and hear the entire group in real-time, while participating from your home or another independent location. Study staff will schedule one brief Vidyo meeting with you to ensure that you are comfortable with using the software.

Post Program Assessment (30 - 45 minutes)

This portion of the study will occur after you have completed the 6-week program. You will fill out several questionnaires. The survey questions will ask you about your stress levels, mental health, behavior, medical symptoms and quality of life. You will have the option to complete these on a personal computer at home or over the phone with a member of our study staff team.

Follow-Up Assessments (30 - 45 minutes)

This portion of the study will occur 3 months after you have completed the 6-week program. You will fill out several questionnaires. The survey questions will ask you about your stress levels, mental health, behavior, medical symptoms and quality of life. You will have the option to complete these questionnaires on a computer from home or over the phone with a member of our study staff team.

Place of Visits: You can attend the online group sessions from your home or any other private place with a personal computer. The personal computer must be equipped with a webcam and Vidyo videoconferencing software.

Confidentiality: Your research study information will remain confidential, stored without identifying information, and be accessible only to study staff. Confidentiality will only be suspended in the case of a psychological emergency. In the unlikely event that a participant is determined to be actively suicidal and at risk for self-harm during any study procedures, the research assistant will contact the Principal Investigator (Vranceanu) and appropriate clinical intervention will be executed. Dr. Vranceanu may start a psychiatric consult depending on the severity of the situation.

OPTION TO CHOOSE:

The study investigator may wish to re-contact you in the future about related research studies. Do you agree to let us contact you in the future?
YES: ________ NO: _________

Reminders via Text Message

With your permission, we would like to send you text messages to remind you of specific assignments related to the treatment and physical activity encouragements. The Partners standard is to send secure text messages. If you prefer, we can send you "unencrypted" texts that are not secure and could result in the unauthorized use or disclosure of your information. If you want to receive communication by unencrypted texts despite these risks, Partners Healthcare will not be held responsible. Text message and data rates may apply based on your cell phone service plan. Your preference to receive unencrypted texts will apply to text messages sent to you from research staff in this study. If you wish to communicate with other research staff at Partners regarding additional studies, your preference will have to be documented with each research study.
These texts will not contain any protected health information. You have the right to refuse these texting reminders.
Your decision will not impact your ability to participate in the study. You can opt out of these text messages at any time.

Please select one of the following options:

I consent to text message reminders (please use your initials to indicate your response):

Yes: _________ No: _________

What are the risks and possible discomforts from being in this research study?

There are no foreseeable physical risks from this research study. Responding to questions about your recent feelings, emotions, and thoughts may cause you to feel discomfort. If you experience any of these symptoms, you may choose not to answer any question that makes you feel uncomfortable. You may also find it time consuming to participate in the 6 visits.

What are the possible benefits from being in this research study?

You will not benefit from this study directly. You may enjoy the opportunity to talk about your ANI experience and to share your story. However, knowledge from this research study may benefit others by enhancing our understanding of the role of skills-based interventions in treating future ANI patients or caregivers of ANI patients.

What other treatments or procedures are available for my condition?

The program offered in this research study does not constitute individualized, personal care. These interventions are broad-based training methods that are not tailored to any individual. If you would like formal mental healthcare or personalized instruction in mind body methods, we can give you a referral for psychological treatment that is suitable for you. For example, you may seek psychotherapy or medications outside of this research study or you may participate in other research studies for which you may qualify.

Participation in this research study does not mean that you cannot seek other forms of treatment for psychological distress, including medications or other forms of psychotherapy. In fact, we ask that you continue your regular medical treatment with your physician in addition to taking part in this research study.

Can I still get medical care within Partners if I don’t take part in this research study, or if I stop taking part?

Yes. Your decision won’t change the medical care you get within Partners now or in the future. There will be no penalty, and you won’t lose any benefits you receive now or have a right to receive.

Taking part in this research study is up to you. You can decide not to take part. If you decide to take part now, you can change your mind and drop out later. We will tell you if we learn new information that could make you change your mind about taking part in this research study.

What should I do if I want to stop taking part in the study?

If you take part in this research study, and want to drop out, you should tell us. We will make sure that you stop the study safely. We will also talk to you about follow-up care, if needed.

Also, it is possible that we will have to ask you to drop out of the study before you finish it. If this happens, we will tell you why. We will also help arrange other care for you, if needed.

Will I be paid to take part in this research study?

All participants will receive $20 for the completion of each of the 3 assessment points. Participants can receive up to $60 for study participation.

What will I have to pay for if I take part in this research study?

There will be no cost to you for any study visits. All of the group sessions and study assessments will be paid for by study funds. However, you will be required to have access to a personal computer equipped with a webcam or other video/audio capture device. The study will not provide you with such a computer. In addition, you will be required to download and install Vidyo videoconferencing software in order to participate in the online group sessions. This software is available for a free download, and study staff will give you specific instruction on how to locate and install it onto your computer.

What happens if I am injured as a result of taking part in this research study?

We will offer you the care needed to treat any injury that directly results from taking part in this research study. We reserve the right to bill your insurance company or other third parties, if appropriate, for the care you get for the injury. We will try to have these costs paid for, but you may be responsible for some of them. For example, if the care is billed to your insurer, you will be responsible for payment of any deductibles and co-payments required by your insurer.

Injuries sometimes happen in research even when no one is at fault. There are no plans to pay you or give you other compensation for an injury, should one occur. However, you are not giving up any of your legal rights by signing this form.

If you think you have been injured or have experienced a medical problem as a result of taking part in this research study, tell the person in charge of this study as soon as possible. The researcher's name and phone number are listed in the next section of this consent form.

If I have questions or concerns about this research study, whom can I call?

You can call us with your questions or concerns. Our telephone numbers are listed below. Ask questions as often as you want.

Ana-Maria Vranceanu, PhD, is the person in charge of this research study. You can call her at 617-724-4977, Monday through Friday, 9:00 am to 5:00 pm. You can also call Dr. Jonathan Rosand, MD, at 617-724-2698, or Melissa Gates, at 617-643-9406, Monday through Friday, 9:00 am to 5:00 pm, with questions about this research study.

If you have questions about the scheduling of appointments or study visits, please call Melissa Gates, at 617-643-9406.

If you want to speak with someone **not** directly involved in this research study, please contact the Partners Human Research Committee office. You can call them at 857-282-1900.

You can talk to them about:

- Your rights as a research subject
- Your concerns about the research
- A complaint about the research

Also, if you feel pressured to take part in this research study, or to continue with it, they want to know and can help.

If I take part in this research study, how will you protect my privacy?

Federal law requires Partners to protect the privacy of health information and related information that identifies you. We refer to this information as “identifiable information.”

Subject Identification

In this study, we may collect identifiable information about you from:

- Past, present, and future medical records
- Research procedures, including research office visits, tests, interviews, and questionnaires

Who may see, use, and share your identifiable information and why:

- Partners researchers and staff involved in this study
- The sponsor(s) of the study, and people or groups it hires to help perform this research or to audit the research
- Other researchers and medical centers that are part of this study
- The Partners ethics board or an ethics board outside Partners that oversees the research
- A group that oversees the data (study information) and safety of this study
- Non-research staff within Partners who need identifiable information to do their jobs, such as for treatment, payment (billing), or hospital operations (such as assessing the quality of care or research)
- People or groups that we hire to do certain work for us, such as data storage companies, accreditors, insurers, and lawyers
- Federal agencies (such as the U.S. Department of Health and Human Services (DHHS) and agencies within DHHS like the Food and Drug Administration, the National Institutes of Health, and the Office for Human Research Protections) state agencies, and foreign government bodies that oversee, evaluate, and audit research, which may include inspection of your records
- Public health and safety authorities, if we learn information that could mean harm to you or others (such as to make required reports about communicable diseases or about child or elder abuse)
- Other researchers within or outside Partners, for use in other research as allowed by law.

Certificate of Confidentiality

A federal Certificate of Confidentiality (Certificate) has been issued for this research to add special protection for information and specimens that may identify you. With a Certificate, unless you give permission (such as in this form) and except as described above, the researchers are not allowed to share your identifiable information or identifiable specimens, including for a court order or subpoena.

Certain information from the research will be put into your medical record and will not be covered by the Certificate. This includes records of medical tests or procedures done at the hospitals and clinics, and information that treating health care providers may need to care for you. Please ask your study doctor if you have any questions about what information will be included in your medical record. Other researchers receiving your identifiable information or specimens are expected to comply with the privacy protections of the Certificate. The Certificate does not stop you from voluntarily releasing information about yourself or your participation in this study.

Even with these measures to protect your privacy, once your identifiable information is shared outside Partners, we cannot control all the ways that others use or share it and cannot promise that it will remain completely private.

Because research is an ongoing process, we cannot give you an exact date when we will either destroy or stop using or sharing your identifiable information. Your permission to use and share your information does not expire.

The results of this research may be published in a medical book or journal, or used to teach others. However, your name or other identifiable information **will not** be used for these purposes without your specific permission.

Your Privacy Rights

You have the right **not** to sign this form that allows us to use and share your identifiable information for research; however, if you don't sign it, you can't take part in this research study.

You have the right to withdraw your permission for us to use or share your identifiable information for this research study. If you want to withdraw your permission, you must notify the person in charge of this research study in writing. Once permission is withdrawn, you cannot continue to take part in the study.

If you withdraw your permission, we will not be able to take back information that has already been used or shared with others, and such information may continue to be used for certain purposes, such as to comply with law or maintain the reliability of the study.

You have the right to see and get a copy of your identifiable information that is used or shared for treatment or for payment. To ask for this information, please contact the person in charge of this research study. You may only get such information after the research is finished.

Subject Identification

Informed Consent and Authorization

Statement of Person Giving Informed Consent and Authorization

- I have read this consent form.
- This research study has been explained to me, including risks and possible benefits (if any), other possible treatments or procedures, and other important things about the study.
- I have had the opportunity to ask questions.
- I understand the information given to me.

Signature of Subject:

I give my consent to take part in this research study and agree to allow my identifiable information to be used and shared as described above.

_______________________	_______________	_______________
Subject	Date	Time (optional)

Signature of Study Doctor or Person Obtaining Consent:

Statement of Study Doctor or Person Obtaining Consent

- I have explained the research to the study subject.
- I have answered all questions about this research study to the best of my ability.

_______________________	_______________	_______________
Study Doctor or Person Obtaining Consent	Date	Time (optional)

Consent of Subjects Who Cannot Read or Write or are Physically Unable to Talk or Write

The consent form was presented orally to the subject in the subject's own language, the subject was given the opportunity to ask questions, and the subject has indicated his/her consent and authorization for participation by (check one box as applicable):

Subject Identification

- Making his/her mark above
- Other means____
  (fill in above)

Consent Form Version Date: 8/6/2019
