# Supplementary material for: Feasibility and Efficacy of a Resiliency Intervention for the Prevention of Chronic Emotional Distress Among Survivor-Caregiver Dyads Admitted to the Neuroscience Intensive Care Unit: A Randomized Clinical Trial
Source: JAMA Netw Open. 2020 Oct 14;3(10):e2020807. doi: 10.1001/jamanetworkopen.2020.20807 (PMC7557506; doi:10.1001/jamanetworkopen.2020.20807)
Supplement: Supplement 2. — eFigure. Adjusted Mean Scores for Depression (Hospital Anxiety and Depression Scale), Anxiety (Hospital Anxiety and Depression Scale), and PTS (PTSD Checklist–Civilian Version) by Randomization from the Shared Baseline [file jamanetwopen-e2020807-s002.pdf]

Supplemental Online Content

Vranceanu AM, Bannon S, Mace R, et al. Feasibility and efficacy of a resiliency intervention for the prevention of chronic emotional distress among survivor-caregiver dyads admitted to the neuroscience intensive care unit: a randomized clinical trial. *JAMA Netw Open*. 2020;3(10):e2020807. doi:10.1001/jamanetworkopen.2020.20807

eFigure. Adjusted Mean Scores for Depression (Hospital Anxiety and Depression Scale), Anxiety (Hospital Anxiety and Depression Scale), and PTS (PTSD Checklist–Civilian Version) by Randomization from the Shared Baseline

This supplemental material has been provided by the authors to give readers additional information about their work.

© 2020 Vranceanu A-M et al. *JAMA Network Open.*

eFigure. Adjusted Mean Scores for Depression (Hospital Anxiety and Depression Scale), Anxiety (Hospital Anxiety and Depression Scale), and PTS (PTSD Checklist–Civilian Version) by Randomization from the Shared Baseline

Mixed-model repeated-measure ANOVAs for patients and caregivers.

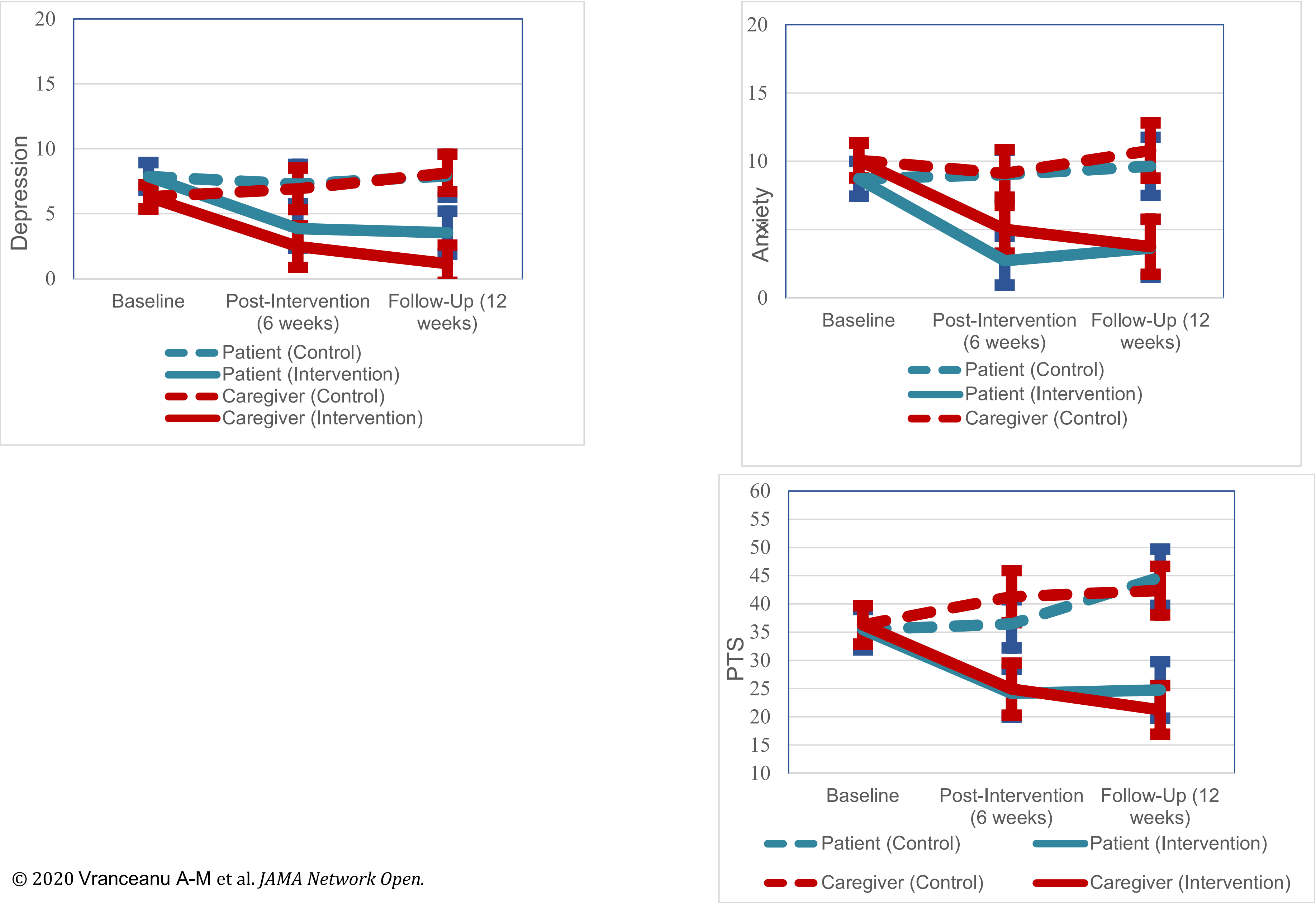

© 2020 Vranceanu A-M et al. *JAMA Network Open.*
