# Supplementary material for: Feasibility and Efficacy of a Resiliency Intervention for the Prevention of Chronic Emotional Distress Among Survivor-Caregiver Dyads Admitted to the Neuroscience Intensive Care Unit: A Randomized Clinical Trial
Source: JAMA Netw Open. 2020 Oct 14;3(10):e2020807. doi: 10.1001/jamanetworkopen.2020.20807 (PMC7557506; doi:10.1001/jamanetworkopen.2020.20807)
Supplement: Supplement 3. — Data Sharing Statement [file jamanetwopen-e2020807-s003.pdf]

Data Sharing Statement

Vranceanu. Feasibility and Efficacy of a Resiliency Intervention for the Prevention of Chronic Emotional Distress Among Survivor-Caregiver Dyads Admitted to the Neuroscience Intensive Care Unit. *JAMA Netw Open*. Published October 14, 2020.
10.1001/jamanetworkopen.2020.20807

Data
Data available: No

Additional Information
Explanation for why data not available: Authors have full control of all primary data, and the journal is welcome to review these data should they request to do so.
